# Transcriptional Profiling of *Coxiella burnetii* Reveals Extensive Cell Wall Remodeling in the Small Cell Variant Developmental Form

**DOI:** 10.1371/journal.pone.0149957

**Published:** 2016-02-24

**Authors:** Kelsi M. Sandoz, David L. Popham, Paul A. Beare, Daniel E. Sturdevant, Bryan Hansen, Vinod Nair, Robert A. Heinzen

**Affiliations:** 1 *Coxiella* Pathogenesis Section, Laboratory of Bacteriology, Rocky Mountain Laboratories, National Institute of Allergy and Infectious Diseases, National Institutes of Health, Hamilton, Montana, United States of America; 2 Department of Biological Sciences, Virginia Tech, Blacksburg, Virginia, United States of America; 3 Genomics Unit, Research Technologies Branch, Rocky Mountain Laboratories, National Institute of Allergy and Infectious Diseases, National Institutes of Health, Hamilton, Montana, United States of America; 4 Electron Microscopy Unit, Research Technologies Branch, Rocky Mountain Laboratories, National Institute of Allergy and Infectious Diseases, National Institutes of Health, Hamilton, Montana, United States of America; Kansas State University, UNITED STATES

## Abstract

A hallmark of *Coxiella burnetii*, the bacterial cause of human Q fever, is a biphasic developmental cycle that generates biologically, ultrastructurally, and compositionally distinct large cell variant (LCV) and small cell variant (SCV) forms. LCVs are replicating, exponential phase forms while SCVs are non-replicating, stationary phase forms. The SCV has several properties, such as a condensed nucleoid and an unusual cell envelope, suspected of conferring enhanced environmental stability. To identify genetic determinants of the LCV to SCV transition, we profiled the *C*. *burnetii* transcriptome at 3 (early LCV), 5 (late LCV), 7 (intermediate forms), 14 (early SCV), and 21 days (late SCV) post-infection of Vero epithelial cells. Relative to early LCV, genes downregulated in the SCV were primarily involved in intermediary metabolism. Upregulated SCV genes included those involved in oxidative stress responses, arginine acquisition, and cell wall remodeling. A striking transcriptional signature of the SCV was induction (>7-fold) of five genes encoding predicted L,D transpeptidases that catalyze nonclassical 3–3 peptide cross-links in peptidoglycan (PG), a modification that can influence several biological traits in bacteria. Accordingly, of cross-links identified, muropeptide analysis showed PG of SCV with 46% 3–3 cross-links as opposed to 16% 3–3 cross-links for LCV. Moreover, electron microscopy revealed SCV with an unusually dense cell wall/outer membrane complex as compared to LCV with its clearly distinguishable periplasm and inner and outer membranes. Collectively, these results indicate the SCV produces a unique transcriptome with a major component directed towards remodeling a PG layer that likely contributes to *Coxiella*’s environmental resistance.

## Introduction

*Coxiella burnetii* is a wide-ranging bacterial pathogen that causes the zoonosis Q fever. Humans are generally infected by inhalation of contaminated aerosols generated by domestic livestock, with sheep, goats, and dairy cattle being the primary animal reservoirs. Symptomatic acute infection normally manifest as a severe influenza-like illness with complications including pneumonia and hepatitis. Endocarditis is a rare but serious consequence of chronic infection [1].

During natural infection, *C*. *burnetii* preferentially infects mononuclear phagocytes, such as alveolar macrophages [2, 3]. *In vitro* studies using primary and immortalized phagocytes indicate that *C*. *burnetii* traffics completely through the endocytic pathway to ultimately reside in a vacuole that resembles a functional phagolysosome [4]. Secretion of proteins with effector functions directly into the host cell cytosol by a Dot/Icm type 4B secretion system (T4BSS) is required for modifications of the *Coxiella*-containing vacuole that allow pathogen growth [5]. In pregnant females, extensive colonization of trophoblasts can also occur leading to reproductive problems, such as preterm birth [6, 7]. Parturition by infected animals can deposit tremendous numbers of *C*. *burnetii* into the environment, with placental tissue containing upwards of 10^9^ organisms per gram [6, 8]. The human aerosol infectious dose of *C*. *burnetii* is less than 10 bacteria [9]; consequently, windborne distribution of desiccated placental material from a single point source can cause large Q fever outbreaks [10–14].

Transmission of *C*. *burnetii* from environmental sources is aided by the pathogen’s noted resistance properties [15]. In the late 1950’s, thermal resistance by *C*. *burnetii* resulted in adoption of a higher temperature (71.7°C) for continuous process pasteurization [16]. *C*. *burnetii* remains viable in dried guinea pig blood for greater than 6 months and within random soil samples [15, 17]. Infectious organisms persist after sonication in distilled water for 45 min [18].

Experimental evidence shows that resistance to physical disruption clearly resides with a specialized small cell variant (SCV) morphological form of *C*. *burnetii* that is generated during a biphasic developmental cycle [18–21]. Distinguishing ultrastructural characteristics of SCVs (~0.2 to 0.5 μm in size) include condensed chromatin, a thick cell envelope, and a system of unusual internal membranes. SCVs are stationary phase forms that arise following morphologic differentiation of the large cell variant (LCV), which is considered the replicative form of *C*. *burnetii* [22, 23]. LCVs (>0.5 μm in size) have dispersed chromatin and a typical looking Gram-negative cell envelope. Nearly homogeneous SCV are observed if axenic or infected host cultures are incubated an additional 1 to 2 weeks after *C*. *burnetii* enters the stationary phase of its growth cycle [22, 23]. Interestingly, unlike *E*. *coli*, *C*. *burnetii* can persist in stationary phase stasis for several weeks without losing viability [22, 23].

The SCV is clearly resistant to osmotic and mechanical insult, and physical attributes support the notion that SCV are more environmentally stable than LCV. Unfortunately, data substantiating enhanced SCV resistance to stressors typically encountered in the environment, such as desiccation and heat, are missing as these studies were conducted with mixed cell populations [15]. Moreover, the biological properties of SCV and LCV and the relevance of differentiation in Q fever pathogenesis and disease transmission are poorly defined. The SCV is commonly referenced as the extracellular infectious form of *C*. *burnetii* [24, 25], but laboratory data actually show that SCV and LCV infect cultured cells with roughly equal efficiency [22, 23]. Thus, the *C*. *burnetii* developmental cycle is not designed to generate forms with differential infectivity, such as chlamydial elementary bodies and reticulates bodies [26]. Rather, the cycle appears to ensure that stable SCVs are generated such that, when deposited into the environment by animal reservoirs, serve as long-lived contaminants that can perpetuate the zoonotic cycle by infection of naïve hosts through the aerosol route.

There is limited knowledge of the transcriptome and proteome of *C*. *burnetii* morphological forms that confer their unique biological properties. A handful of proteins have been identified that are differentially synthesized by LCV and SCV, including the small basic DNA binding proteins Hq1 and ScvA that are enriched in the SCV [27]. A recent study employing RNA sequencing identified 15 developmentally regulated small RNAs (sRNA) generated by LCV and SCV cultivated in Vero cells or the synthetic medium, ACCM-2 [28, 29]. Eight sRNAs are expressed at higher levels by organisms grown in host cells versus ACCM-2 [28], suggesting they regulate processes important for intracellular growth. Although ACCM-2 supports *C*. *burnetii* morphological differentiation similar to host cell grown organisms, subtle changes in media composition can dramatically alter this process [23], indicating biphasic development is finely tuned to the biochemical environment.

To improve our understanding of *C*. *burnetii* morphological development, we characterized the program of gene expression associated with LCV to SCV transition in Vero cells. Transcriptional microarray analysis revealed temporal up-regulation of specific subsets of genes in the SCV, including those associated with stress responses, arginine acquisition, and cell wall remodeling. Cell remodeling genes comprised those encoding predicted L,D transpeptidases that catalyze nonclassical 3–3 peptide cross-links in cell wall peptidoglycan (PG) that impart unique structural properties and resistance to β-lactam antibiotics [30, 31]. Consistent with this observation, PG analysis showed a substantially higher percentage of 3–3 cross-links in SCV than LCV of the total cross-linked muropeptides identified. Moreover, cryo-electron microscopy (CEM) revealed a dense layer of unusual thickness, presumably comprised of PG, underlying the outer membrane of SCV, but not LCV. The distinctive gene expression program of SCV described herein provides important clues concerning factors that contribute to *Coxiella*’s environmental resistance and pathogenesis.

## Materials and Methods

### C. burnetii

The *C*. *burnetii* Nine Mile phase II, clone 4 (RSA439) strain was used in this study. SCVs of *C*. *burnetii* were used as inocula in all experiments and were generated by extended incubation of infected African green monkey kidney (Vero) fibroblasts (CCL-81; American Type Culture Collection) [22]. Briefly, Vero cells grown in RPMI 1640 medium (Invitrogen, Carlsbad, CA) supplemented with 2% fetal bovine serum (FBS) were infected with *C*. *burnetii* and bacteria were purified from host cells at 21 to 28 days post-infection as previously described [32]. Purified stocks were titered by enumerating *C*. *burnetii* genome equivalents (GE) and stored at -80°C as previously described [22, 33].

### Infection of Vero cells for microarray

Six-well tissue culture plates were seeded with Vero cells (0.6 x 10^6^ cells/well) in RPMI 1640 medium supplemented with 10% FBS and incubated overnight at 37°C in 5% CO_2_. Cells were washed once with phosphate buffered saline (PBS; 1 mM KH_2_PO_4_, 155 mM NaCl, 3 mM Na_2_HPO_4_, pH 7.4), then infected with *C*. *burnetii* SCV at an multiplicity of infection of 50 based on GE. SCVs were suspended in RPMI without FBS and added in 2 ml volumes to each well. Plates were centrifuged at 400 X *g* for 30 min, the inoculum aspirated, then cells washed 1X with Hank’s buffered saline solution. Three ml of fresh RPMI + 10% FBS was added to each well and the plates incubated at 37°C in 5% CO_2_. Infected cell cultures were washed with PBS, then lysed in situ with 1 ml of Trizol reagent (Invitrogen) at 3, 5, 7, 14, and 21 days post-infection. Samples were frozen at -80°C and RNA was extracted as previously described [34]. Two wells of a 6-well tray represented a single replicate sample.

### Transcription microarray analysis

A MicrobEnrich kit (Applied Biosystems) was used to increase the relative level of *C*. *burnetii* RNA from infected Vero cells. Enriched RNA (~1 μg) was amplified using a MessageAmp II Bacteria kit (Applied Biosystem). Briefly, double stranded cDNA was synthesized and purified using a QiaQuick 96-well system. Biotin-labeled cRNA was then *in vitro* transcribed using cDNA as a template. Ten μg of labeled cRNA was fragmented using Ambion 10X fragmentation buffer (Invitrogen) and incubated at 75°C for 15 min. Quantitative RT-PCR of *C*. *burnetii* FtsI cRNA was conducted to estimate the amount of *C*. *burnetii* cRNA in Vero cell samples. cRNA from uninfected Vero cells was used to determine host cRNA targets that cross react with Affymetrix GeneChip oligos. Cross-reacting species were not considered in the final analysis. Samples were hybridized to a custom Affymetrix GeneChip (RMLchip3a520351F) that contain probes sets corresponding to >96% of the open reading frames encoded by Nine Mile RSA493 reference strain [35, 36].

Hybridization, fluidics, scanning and data analysis were performed according to standard Affymetrix protocols (http://www.affymetrix.com) and as previously described [37]. Briefly, Command Console (CC v3.1, http://www.Affymetrix.com) software was used to convert the image files to cell intensity data (cel files). All cel files, representing individual samples, were normalized by using the trimmed mean scaling method within expression console (EC v1.2, http://www.Affymetrix.com) to produce the analyzed cel files (chp files) along with the report files. An ANOVA was performed within Partek (Partek, Inc. St. Louis, Mo., v6.6 6.12.0420) to obtain multiple test corrected *p*-values using the false discovery rate method [38]. Significance at the 0.01 level and a two-fold expression level difference was used to filter the probe-set list. These data were combined with fold change values, signal confidence (above background), cross hybridization to mock, and call consistency (as a percent) as calculated using custom Excel templates for each comparison.

### Verification of microarray transcriptional data

The expression level of selected genes was confirmed using the QuantiGene Assay system (Affymetrix) and custom probes according to the manufacturer's directions (Panomics). Validation was conducted using RNA from *C*. *burnetii* cultured in Vero cells or in the synthetic medium ACCM-2 [29]. Infected cells in 6-well tissue culture plates or *C*. *burnetii* harvested from ACCM-2 were lysed directly in QuantiGene lysis buffer, which was combined with blocking buffer and probes. RNA solutions were loaded into a 96-well capture plate and incubated for 24 h at 55°C. RNA was detected by luminescence over 1000 ms with a Tecan Safire^2^ microplate reader (Mannedorg). Background luminescence associated with RNA derived from uninfected Vero cells was subtracted from luminescence associated with RNA derived from *C*. *burnetii*-infected Vero cells to determine gene-specific expression levels. Similarly, deionized water (dH_2_O), was used as a background control for *C*. *burnetii* grown in ACCM-2. Transcriptional signals were normalized to *C*. *burnetii* GE.

### Microcapillary reverse-phase HPLC nano-electrospray tandem mass spectrometry (LC-MS/MS)

*C*. *burnetii* was cultivated in ACCM-2 for 3, 14, or 21 days, then equal numbers of bacteria (based on GE) were washed 3 times with PBS and suspended in 2X Laemmli sample buffer. Samples were boiled prior to one-dimensional SDS-PAGE conducted by the Research Technologies Branch of the National Institute of Allergy and Infectious Diseases. Whole bacterial cell mass spectrometry of separated proteins was performed as previously described [33].

Computer controlled data dependent automated switching to MS/MS by Xcalibur 2.1 software was used for data acquisition and provided the peptide sequence information for protein identification. Databank was performed with Mascot software (Matrix Science). The data was searched against a sequence file containing *C*. *burnetii* proteins found in the UniprotKB TrEMBL database and human, pig, and cow proteins found in UniprotKB SwissProt (03/2014). The data was searched with one allowed missed cleavage, and mass tolerances of 10 ppm and 0.8 Da for the precursor and fragment ions, respectively. Carbamidomethylation of cysteine was set as a fixed modification, while oxidation of methionine, deamidation of asparagine and glutamine, and protein N-terminal acetylation were searched as dynamic modifications.

The resulting search files were re-clustered against the same sequence database for further analysis using ProteoIQ Software v. 2.7 (PREMIER Biosoft), and results were preferentially parsed to *C*. *burnetii* Nine Mile RSA493. Assignments were filtered using the Protein Prophet algorithm as implemented within ProteoIQ, with cutoff filters set to 95% for peptides, and 99% for proteins. Protein assignments were only considered if they met both the probability thresholds and 2 spectra per peptide sequence/2 peptides per protein minimums. Two forms of sample normalization were performed, a normalized spectral abundance factor approach and a total sampling normalization. The normalization factors applied to the data were 1.00, 1.01, and 1.06 for 3, 14, and 21 day samples, respectively.

### PG isolation

*C*. *burnetii* LCV and SCV were purified from infected Vero cells in T-150 tissue culture flasks after 5 (24 flasks) and 14 days (12 flasks) of growth, respectively. Bacterial pellets were suspended in 4 ml of dH_2_O, and the suspension kept on ice while being added dropwise to 50 ml of boiling 4% sodium dodecyl sulfate (SDS). Lysed bacteria were boiled with stirring for 30 min, and water added throughout the procedure to maintain a 50 ml volume. Lysates were cooled to room temperature, then centrifuged at 110,000 X *g* for 60 min at room temperature. The supernatants were discarded and the pellets suspended in 20 ml of dH_2_O at 50°C. Samples were centrifuged again, supernatants removed, and pellets suspended in 20 ml of dH_2_O that was heated to 100°C for 2 min to dissolve any precipitated SDS. This procedure was repeated until SDS was no longer detected [39]. SDS-free pellets were suspended in 1 ml of 100 mM Tris HCl (pH 7.5) containing 100 μg of α-amylase (Sigma-Aldrich) and the samples incubated at 37°C for 1 h. DNase I (10 μg; Sigma-Aldrich), RNase (40 μg; Sigma-Aldrich), and MgSO_4_ (final concentration = 20 mM) were added to samples that were incubated at 37°C for 2 h. Trypsin (40 μg; Worthington Biochemical Corp.) and CaCl_2_ (final concentration = 10 mM) were added to samples that were incubated at 37°C overnight. Samples were centrifuged at 110,000 X *g* for 1 h and the pellets suspended in 4 ml of 1 N NaOH. Samples were incubated at room temperature for 20 h, then centrifuged at 110,000 X *g* for 1 h. Pellets were washed with dH_2_O and the sample centrifuged again. The supernatant was discarded and the final pellet comprised of PG suspended in 150 μl of dH_2_O. Samples were stored at -80°C.

### PG analysis

Purified PG was digested with 250 units Mutanolysin (Sigma) in 12.5 mM NaPO_4_ (pH 5.5) for 16 h at 37°C [40]. Solubilized muropeptides were separated from insoluble material by centrifugation at 110,000 X *g* for 1 h. Equivalent samples of the supernatant and pellet samples were subjected to amino acid analysis [41] to quantify PG digestion. Solubilized muropeptides were dried under vacuum and resuspended in 0.25 M Na_2_B_4_O_7_ (pH 9.0). Terminal sugars were reduced by addition of NaBH_4_ to 5 mg/ml and incubation for 10 minutes. Reduction was terminated by addition of H_3_PO_4_ to bring the pH to ~2. Muropeptides were separated and purified by reverse-phase HPLC essentially as described [40]. Structural information on purified muropeptides was obtained using MALDI-TOF-TOF mass spectrometry [40, 42]

### Transmission and cryo-electron microscopy

For transmission electron microscopy (TEM), *C*. *burnetti* infected Vero cells were grown on 13 mm Thermanox coverslips (Nunc) for the indicated time, then fixed overnight at 4°C with 2.5% glutaraldehyde in 100 mM sodium cacodylate buffer (pH 7.2). Cells were post-fixed with 1% osmium tetroxide-0.8% potassium ferricyanide in 100 mM sodium cacodylate buffer. Samples were then stained with 1% tannic acid in distilled water followed by 2.5% samarium acetate. Secondary fixation and staining steps were conducted in a Pelco Biowave for 2 min on 2 min off 2 min on at 250 W under vacuum. Sample were dehydrated with a graded ethanol series, then embedded in epon/araldite resin. Thin sections were cut with a Leica UC 6 (Leica) ultramicrotome and viewed on a 80 kV Hitachi 7500 (Hitachi) electron microscope. Digital images were acquired with a Hammamatsu XR-100 bottom mount CCD system (Advanced Microscopy Techniques).

For CEM, 3.5 μl of a *C*. *burnetii* suspension was frozen on a glow discharged Quantifoil^™^ R 2/2 grid mounted in an autogrid assembly using Vitrobot Mark IV (FEI). Relative humidity was set at 85% with a blot force of 5, drain time of 1 sec, and blot time of 3.5 sec. Images were collected using an FEI Titan Krios 300kV FEG-TEM equipped with an FEI Falcon II CMOS direct electron detection camera. Images were captured in microprobe mode at a nominal magnification of 29,000 X, which corresponds to a pixel size of 2.87 Å. For added contrast, images were collected using a FEI Volta phase plate at 5 μ defocus at a dose rate of ~20–23 e^-^/Å^2^. Non-phase plate images were captured using a 70 μ objective aperture under similar conditions.

## Microarray Data

The microarray data have been deposited in the NCBI GEO public database under accession number GSE74489.

## Results and Discussion

### Kinetics of *C*. *burnetii* morphological differentiation

**Fig 1:**
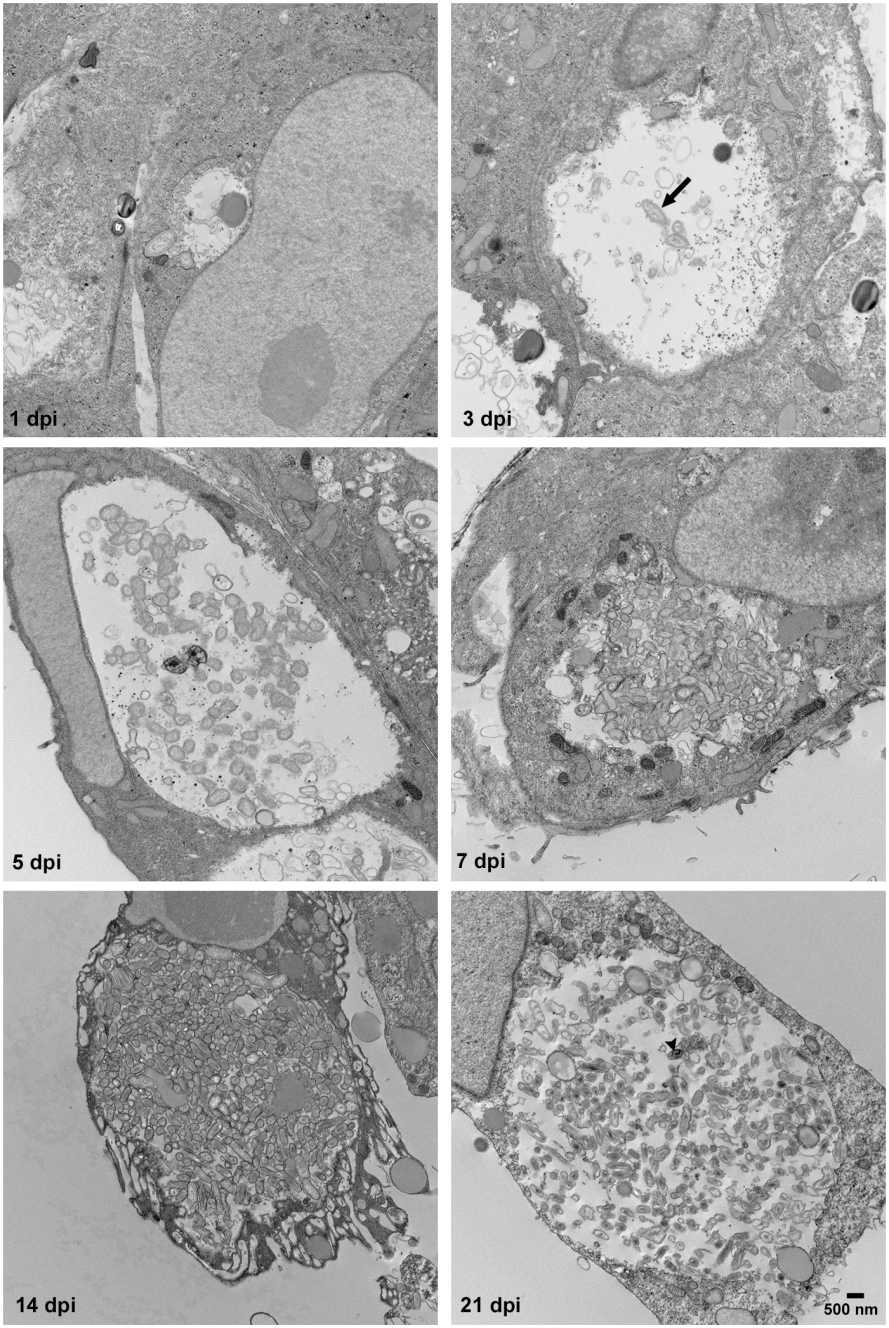
*C*. *burnetii* morphological development in Vero cells. Vero cell monolayers were incubated with purified SCV for 1 h to allow for adherence and internalization. Extracellular organisms were then washed from cell monolayers and fresh media added. Infected cells were fixed and processed for transmission electron microscopy at 1, 3, 5, and 7, 14, and 21 days post-infection (dpi). Prototypic LCV and SCV are designated in selected panels with an arrow and arrowhead, respectively. Bar, 500 nm.

We have previously established *C*. *burnetii* growth kinetics and corresponding morphological differentiations to the onset of stationary phase using a Vero cell model of infection [22, 43, 44]. Following infection with SCV, *C*. *burnetii* lag phase lasts approximately 2 days and consists of SCV to LCV morphogenesis. Exponential growth of LCV occurs over the next 4 days, culminating with the onset of stationary phase and the appearance of SCV and intermediate forms. If infected cultures are incubated an additional 1 to 3 weeks, parasitophorous vacuoles harbor nearly homogeneous SCV [22]. To more carefully document the timing of *C*. *burnetii* morphological differentiation, infected Vero cells were processed for electron microscopy at 3, 5, 7, 14, and 21 days post-infection (Fig 1). LCV and SCV were scored based on size and on the presence of dispersed or compacted chromatin, respectively. At 3 and 5 days post-infection, LCV were observed in large and spacious PV. At 7 days post-infection, replicating bacteria had nearly filled the PV lumen and the population contained mixture of LCV, SCV and intermediate forms. This time point correlates with the onset of the growth cycle stationary phase in various *C*. *burnetii* culture systems [4, 22, 23]. At 14 and 21 days post-infection, the cell populations were highly enriched for SCV.

### Transcriptional microarray detects gene expression associated with LCV to SCV developmental transition

Temporal orchestration of gene expression is required for bacteria undergoing developmental changes [45]. To gain insight into the program of gene expression governing differentiation of LCV to SCV, we profiled by microarray the transcriptome of *C*. *burnetii* grown in Vero cells for 3 (early LCV), 5 (late LCV), 7 (intermediate forms), 14 (early SCV), and 21 (late SCV) days.

**Fig 2:**
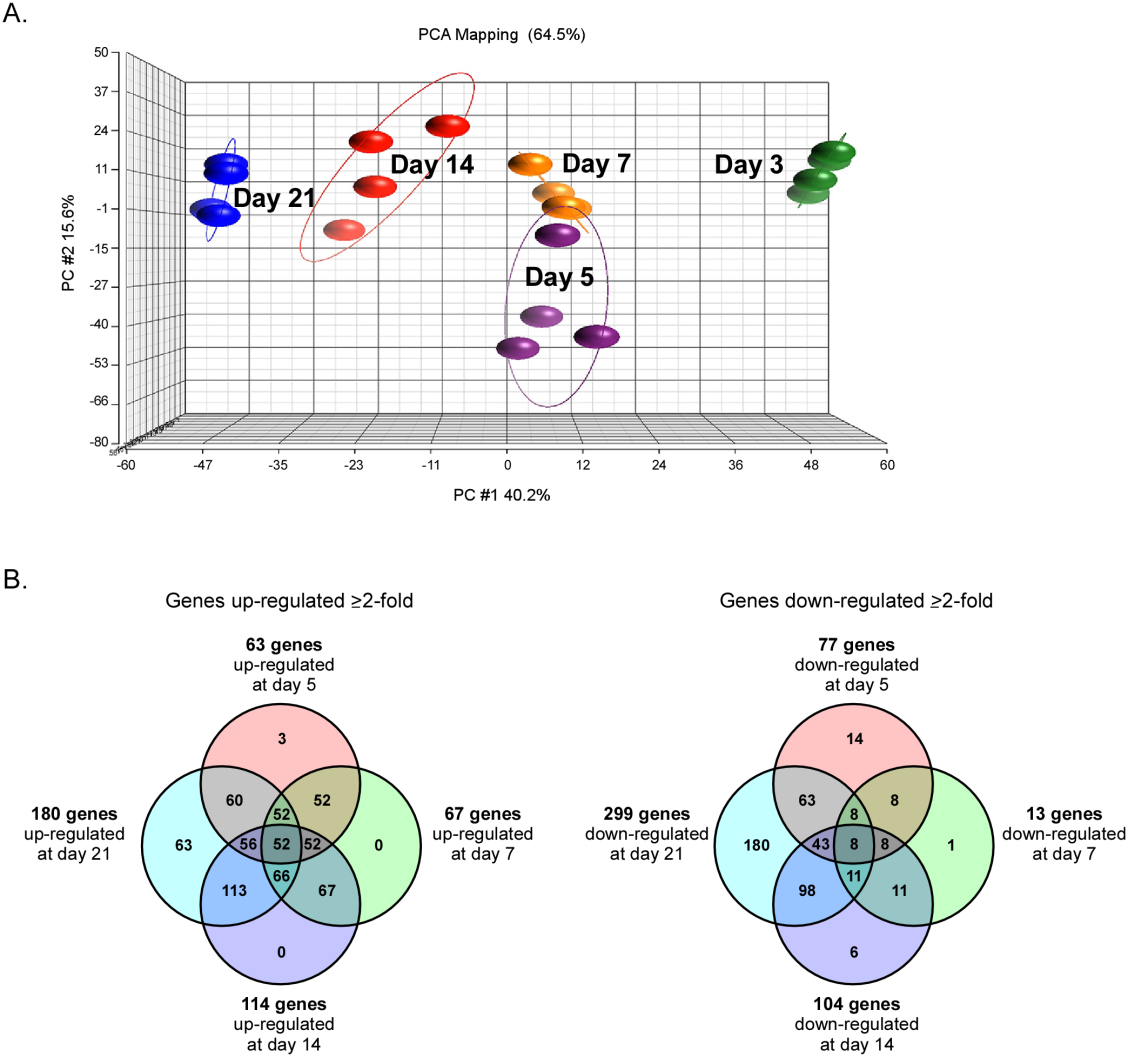
Transcriptional profiling of *C*. *burnetii* LCV to SCV morphological transition during intracellular growth in Vero cells. (A) Principal-component analysis showing distinct grouping of microarray expression data at each time point (4 biological replicate samples). (B) Venn diagrams showing the number of genes up or downregulated ≥ 2 fold at 5, 7, 14, and 21 days post-infection of Vero cells relative to 3 days post-infection.

Principal components analysis revealed greater separation between time points than between replicates within a time point, indicating developmental cycle-specific gene expression changes were occurring (Fig 2A). To identify the genes differentially regulated during LCV to SCV transition, we compared the microarray signals associated with each gene at 5, 7, 14, and 21 days post-infection to signals generated with the 3 days post-infection sample. Using a ≥ 2-fold cutoff, statistically significant up-regulation of 63, 67, 114, and 180 genes at 5, 7, 14, and 21 days post-infection, respectively, was observed. Conversely, 77, 13, 104, and 299 genes were expressed ≥ 2-fold less at 5, 7, 14, and 21 days post-infection, respectively (Fig 2B and S1 Table). Fifty-two and 8 genes showed up and downregulated expression levels, respectively, of ≥ 2-fold at all time points relative to the 3 day time point (Fig 2B and S1 Table). Unless otherwise noted, the following discussion of expression levels of individual genes refers to levels observed between early LCV (3 days post-infection) and late SCV (21 days post-infection).

### Conversion to the SCV results in downregulation of metabolic genes

The overall expression profile of downregulated genes in SCV is consistent with stringent response physiology in response to amino acid depletion [46]. Consequently, many genes involved in protein biosynthesis, such as those encoding rRNAs, ribosomal proteins, and tRNA synthetases, were downregulated at least 2-fold during LCV to SCV transition (S1 Table). Nutrient limitation is predicted as the PV becomes densely packed with bacteria during the late exponential phase of growth. CBU1931, encoding a hypothetical protein, showed the greatest downregulation (9.7-fold).

### Transition to the SCV induces multiple stress response pathways

**Fig 3:**
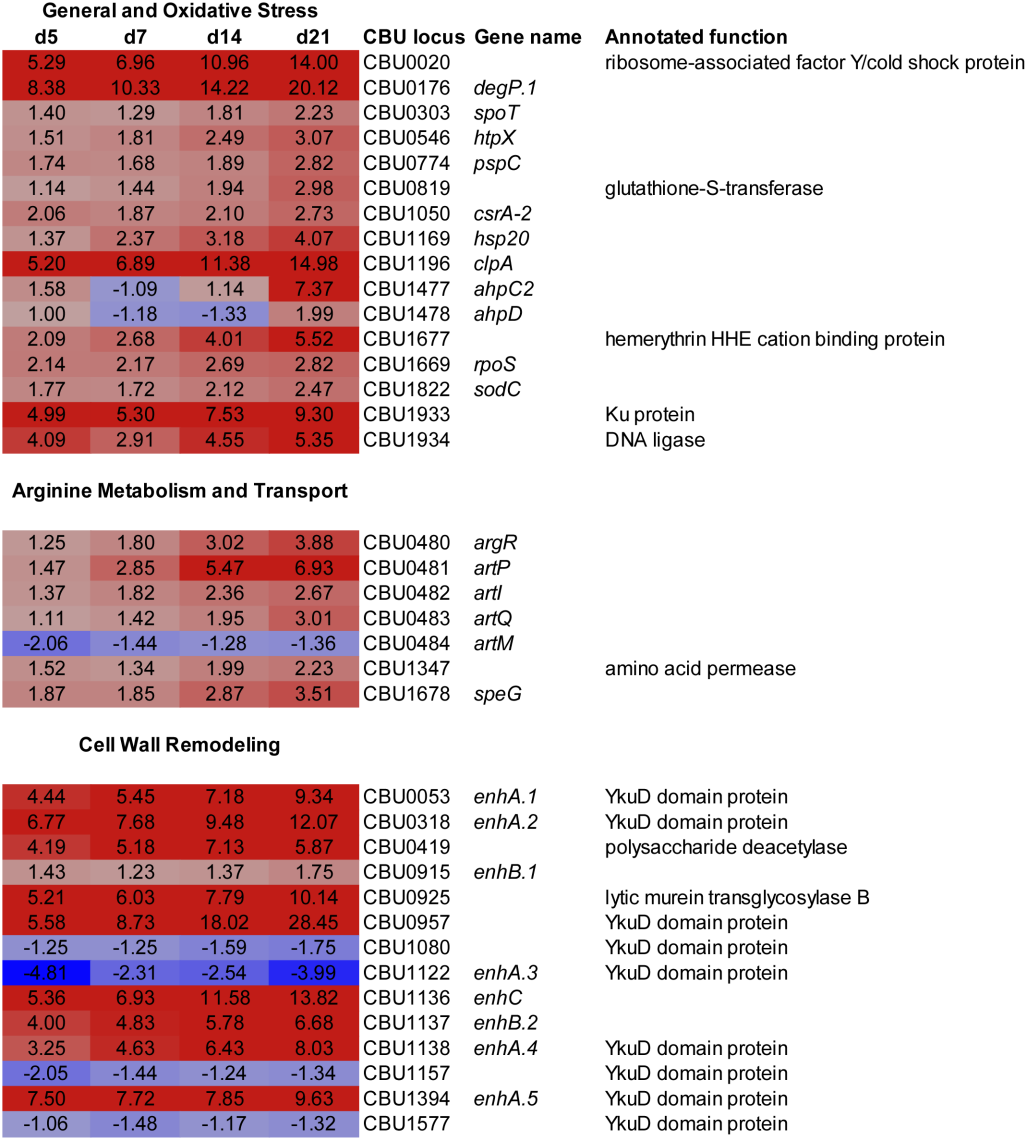
Expression levels of *C*. *burnetii* gene subsets that are upregulated during transition of LCV to SCV. Fold-increase in mRNA levels at 5, 7, 14, and 21 days post-infection relative to 3 days post-infection with a corresponding color code is depicted. Gene subsets shown are those involved in general and oxidative stress, arginine metabolism and transport, and cell wall remodeling.

Nutrient limitation upon bacterial entry into stationary phase triggers multiple stress responses that enable survival in a growth-arrested state [47]. Several genes encoding proteins with regulatory roles in stationary phase survival were upregulated in SCV (Fig 3). A critical positive regulator of genes conferring bacterial resistance during stationary phase is the sigma factor RpoS (σ^S^) [47]. Accordingly, *C*. *burnetii rpoS* was induced 2.8-fold during late stationary phase. Moreover, *spoT*, which produces the alarmone ppGpp that positively regulates expression of *rpoS* [48], was induced 2.2-fold. Up-regulation of *pspC* (2.8-fold) encoding a predicted transcriptional regulator involved in management of membrane stress was also observed [49]. *C*. *burnetii* encodes two mRNA-binding Csr (carbon storage regulator) proteins. Global post-transcriptional regulation by Csr proteins is complex, but is generally associated with activation and repression of genes needed for growth and stasis, respectively [50]. Interestingly, *csrA-2* was upregulated (2.7-fold) while *csrA-1* (*cbu0024*) was downregulated (2.5-fold) in SCV, suggesting specific roles in regulating *C*. *burnetii* developmental transitions (Fig 3 and S1 Table). Of note, *C*. *burnetii* lacks predicted CsrA-regulating sRNAs [51]. *cbu0960*, encoding a predicted transcriptional regulator with a helix-turn-helix domain (Pfam1381), was also induced 7.9-fold (S1 Table).

Increased oxidative stress is a hallmark of growth-arrested bacteria in stationary phase. Reactive oxygen species damage several macromolecules, including proteins where carbonylation of amino acid side chains and oxidation of metal cofactors can occur [47]. Accordingly, several oxidative stress response-related genes were induced during differentiation to the SCV including *ahpC2* (7.4-fold) and *ahpD* (2.0-fold), encoding a peroxiredoxin and peroxiredoxin reductase, respectively (Fig 3). These proteins form an alkylhydroperoxide reductase complex that detoxifies organic peroxides, hydroperoxides, and peroxynitrite. Alkylhydroperoxide reductases are considered the primary scavenger of low dose hydrogen peroxide generated by endogenous metabolism [52]. AhpC of *Brucella abortus* plays a major role in maintaining stationary phase viability in nutrient-limited media [53]. *sodC*, encoding a Cu,Zn superoxide dismutase (SOD) was induced 2.5-fold. A periplasmic Cu,Zn SOD aids survival of *Legionella pneumophila* in stationary phase [54]. Interestingly, *C*. *burnetii* secretes this enzyme from a periplasmic location via membrane blebbing [55, 56]. A gene (*cbu1677*) encoding a hemerythrin-like protein, associated with sensing and detoxification of oxygen [57] was also induced 5.5-fold. Glutathione S-transferase (*cbu0819*) was induced 3.0-fold. This class of enzyme protects bacteria against oxidative and chemical stress by conjugating toxic components to the non-protein thiol, glutathione [58].

A consequence of amino acid limitation during stationary phase is mistranslation of proteins that are more prone to oxidation [47]. The transcriptional profile of *C*. *burnetii* transitioning to SCV suggest deployment of several mechanisms involved in quality control and *de novo* synthesis of proteins required for extended stationary phase survival (Fig 3). The gene encoding ribosome associated factor Y (*cbu0020*), a protein thought to improve translational fidelity under amino acid-limiting conditions [59], was induced 10.5-fold. The gene encoding the small heat shock protein Hsp20 (*cbu1169*), predicted to serve as chaperone in maintaining correct protein structure [60], was induced 4.1-fold. Conversely, induction of *htpX* (3.1-fold), *degP*.*1* (20.1-fold), and *clpA* (15.0-fold) that encode proteins involved in degradation of misfolded or damaged proteins [61–63], was also observed. *De novo* protein synthesis is required for stationary phase survival, and protease-dependent autophagy is thought to contribute necessary amino acids [47, 64]. The Clp family of proteases is particularly important in survival of bacteria in stationary phase [63].

Extended cell-cycle exit during stationary phase exposes DNA to multiple insults that can result in double stranded breaks. Because the lower chromosome copy number of stationary phase cells limits repair by homologous recombination, the non-homologous end-joining (NHEJ) pathway of repair is critical for maintaining DNA integrity and bacterial viability [65]. The NHEJ pathway employs Ku protein and an ATP-dependent DNA ligase, usually encoded by an operon [65]. *C*. *burnetii* SCV showed 9.3-fold induction of *cbu1933*, encoding Ku, and 5.3-fold induction of adjacent *cbu1934*, encoding an ATP-dependent DNA ligase (Fig 3). Of note, condensed chromatin, as observed in the SCV, is proposed to facilitate repair of double stranded breaks by limiting diffusion of break termini [65].

### Genes encoding transporters of essential amino acids are induced in the SCV

The *C*. *burnetii* Nine Mile genome contains 122 genes annotated as encoding transporters [66]. Within this cohort are 30 genes that direct synthesis of predicted transporters of amino acids or peptides, predicted to be preferred carbon and energy sources of *C*. *burnetii* [67] (S2 Table). Four ATP-binding cassette (ABC) superfamily transport systems account for fourteen proteins [68]. There are eight amino acid-polyamine-organocation (APC) family permeases [69], four major facilitator superfamily (MFS) transporters [70], two proton-dependent oligopeptide transport (POT) family transporters, one oligopeptide (OPT) family transporter [66], and one hydroxy/aromatic amino acid permease (HAAAP) family transporter [66]. Only 6 amino acid transporters were upregulated in SCV with no transporters of peptides upregulated (Fig 3 and S1 Table). Four of 6 induced transporters are predicted to acquire the essential amino acids L-arginine (ABC transporter *artPIQ* and the APC permease CBU1347) [66, 71], L-valine (MSF transporter CBU0515), and L-proline (MSF transporter CBU2058) [66]. The substrate specificities of CBU1347 and CBU0515 are based on homology to defined transporters of *Franscisella novicida* (*ArgP*; 40% aa identity) [71] and *L*. *pneumophila* (*PhtJ*; 36% aa identity) [70]. The substrate specificity of induced APC transporters CBU0426 and CBU0354 are unknown [66]. Interestingly, *argR* encoding an L-arginine repressor protein and immediately upstream of *artPIQ*, was induced 3.9-fold. ArgR acts as a repressor or activator of gene expression when bound to L-arginine [72], with repression of genes associated with L-arginine biosynthesis or acquisition [72, 73]. The ArgR regulon of *E*. *coli* is comprised of 140 genes, with a complex regulon also described for *L*. *pneumophila* that includes genes encoding the Dot/Icm T4BSS and translocated substrates, as well as stress response proteins [74, 75]. ArgR-mediated regulation is required for adaptive responses that enable *L*. *pneumophila* growth in amoeba, the pathogen’s natural host [75, 76], and virulence gene expression by *Streptococcus pneumoniae* [73]. Expression of *L*. *pneumophila* ArgR is regulated by RpoS [76], which was also upregulated in the SCV. L-arginine also plays important roles in signal transduction originating from the lysosome [77, 78] and killing functions of macrophages [79]. Further elucidation of *C*. *burnetii* metabolism of arginine should improve our understanding of SCV biogenesis and *C*. *burnetii* survival within a phagolysosome-like vacuole of macrophages.

An additional upregulated gene related to arginine metabolism was *speG* (*cbu1678*; 3.5-fold) (Fig 3) that encodes a diamine acetyltransferase capable of acetylating polyamines such as putrescine and spermidine. This modification inactivates cationic polyamines that play diverse roles in bacterial physiology including stabilization of nucleic acids and the cell envelope, and protection against oxidative stress [80]. *C*. *burnetii* appears cable of generating putrescine from arginine by the activity of an arginine decarboxylase (CBU0722) and agmatinase (CBU0720). *C*. *burnetii* may also acquire additional polyamines from the host cell based on the property of basic amines to accumulate in acidic vesicles. Indeed, a polyamine auxotroph of *Leishmania major* can replicate in an acidic parasitophorous vacuole superficially similar to *Coxiella*’s [81, 82]. As opposed to *speG*, *cbu0722* and *cbu0720* were downregulated in SCV (2.3- and 4.6-fold, respectively) (S1 Table). Thus, it is possible that the SCV is limiting accumulation of physiologically active putrescine. It is interesting to speculate on the potential roles of polyamines in *C*. *burnetii* physiology. In Gram-negative bacteria like *C*. *burnetii* that lack Braun’s lipoprotein, polyamines are covalently linked to peptidoglycan to help anchor the cell wall to the outer membrane [83]. Perhaps this modification is important for envelope stability of LCV but not SCV. The nucleic acid stabilizing properties of polyamines may be supplanted in the SCV by chromatin-associated small basic proteins [27]. Indeed, 21 of 42 genes showing the greatest induction (>5.0-fold) in SCV encoded hypothetical proteins with an average pI of 9.3 and M_*r*_ of 16.3 kDa (S3 Table).

### Cell wall remodeling genes are upregulated in the SCV

A striking transcriptional signature of the SCV was induction (>7.6-fold) of 5 of 9 genes encoding YkuD family proteins (Pfam03734) that harbor a domain associated with L,D transpeptidase catalytic activity [30, 84] (Fig 3). L,D transpeptidases catalyze nonclassical 3–3 cross-links between diaminopimelate molecules in PG peptide stems while D,D transpeptidases (also known as penicillin-binding proteins) catalyze 4–3 peptide cross-links between D-alanine and diaminopimelate [85]. In *E*. *coli*, L,D transpeptidases also attach Braun lipoprotein to PG [86]. Bacteria primarily synthesize 4–3 cross-links [85]; however, *E*. *coli* increases the percentage of 3–3 cross-links from 5.8 to 10.6% as it transitions from exponential to stationary phase growth [87], while the cell wall of *Mycobacterium tuberculosis* contains 60% 3–3 cross-links irrespective of growth state [88]. Although the nature of peptide cross-links determines the efficacy of antibiotics that target cell wall transpeptidation [31, 88–91], the structural and physiological benefits of 3–3 cross-links remain obscure. There is a shorter distance between glycan strands with 3–3 cross-links that may increase PG rigidity [92, 93]. Unlike D,D transpeptidases, L,D transpeptidases can also catalyze cross-links without *de novo* synthesis of muropeptide precursors [94], an energy saving strategy for stationary phase bacteria undergoing morphological change [94]. Nonetheless, L,D transpeptidase mutants of *M*. *tuberculosis* show attenuated virulence [31, 91], defects in several physiological processes [31], and aberrant cell shape [31, 93].

**Fig 4:**
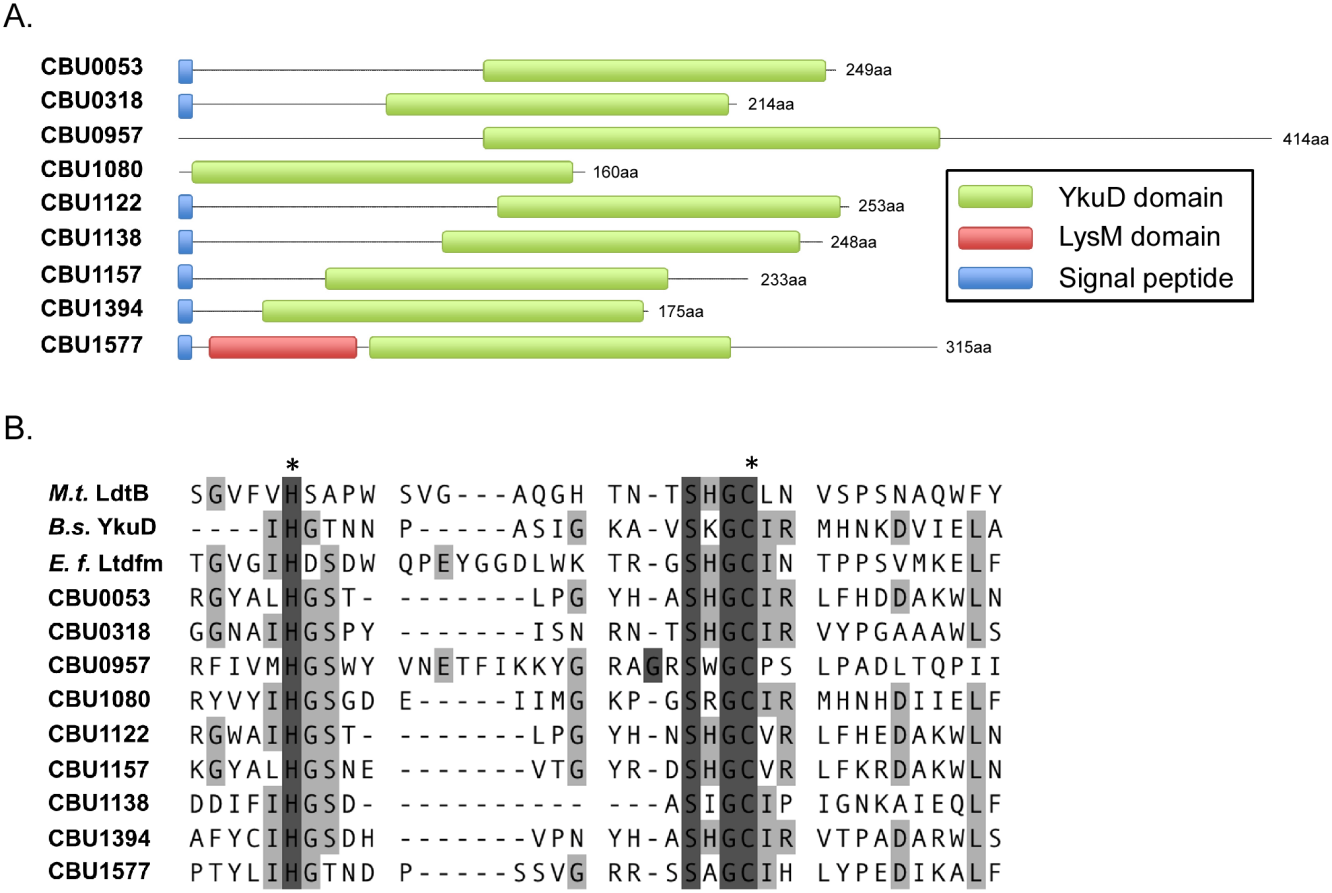
YkuD family proteins of *C*. *burnetii*. (A) Schematic showing location of YkuD domains. Location of signal peptides, predicted by SignalP 4.1, and the LysM domain of CBU1577 are also depicted. (B) Amino acid sequence lineup of the predicted catalytic domains of *C*. *burnetii* YkuD family proteins with *M*. *tuberculosis* LdtB, *E*. *faecium* Ltd_fm_, and *B*. *subtilis* YkuD. Invariant histidine and cysteine proposed as catalytic residues are indicated with asterisks [97].

Four of five induced genes encoding YkuD family proteins were previously annotated as *enh* genes: *enhA*.*1* (*cbu0053*), *enhA*.*2* (*cbu0318*), *enhA*.*4* (*cbu1138*), and *enhA*.*5* (*cbu1394*) The *enh* family of genes was named based on their association with enhanced entry of *L*. *pneumophila* into mammalian cells when overexpressed [95]. An additional YkuD family paralog not annotated as an *enh* gene, *cbu0957*, was also induced. YkuD family *cbu1080*, *cbu1122* (*enhA*.*3*), *cbu1157*, and *cbu1577* were not induced, or were moderately downregulated in SCV (Fig 3). CBU1577 also contains a predicted LysM domain involved in glycan (including PG) binding [96] (Fig 4A). YkuD family proteins contain a highly conserved catalytic domain with invariant histidine and cysteine residues (His336 and Cys354 in *M*. *tuberculosis* L,D-transpeptidase type 2 (LtdB) [84, 97, 98]. Accordingly, this domain was highly conserved in *C*. *burnetii* YkuD family proteins (Fig 4B). The nine *C*. *burnetii* genes encoding YkuD family proteins are conserved as full copies in all sequenced strains, suggesting they serve critical functions in *Coxiella* biology. *Enterococcus faecium* produces a serine/threonine protein phosphatase StpA required for optimal L,D transpeptidase activity [99]. *cbu0488*, annotated as encoding a serine/threonine protein phosphatase, was induced 6.3-fold, suggesting the protein may play a similar role in regulating *C*. *burnetii* L,D transpeptidase activity (S1 Table).

*C*. *burnetii* also contains the paralogs *enhB*.*1* (*cbu0915*) and *enhB*.*2* (*cbu1137*), with *enhB*.*2* upregulated 6.7-fold in SCV. The function of these genes is unknown, but *enhB*.*2* resides in a predicted operon with *enhA*.*4* and *enhC* (*cbu1136*) [100], which was induced 13.9-fold. EnhC of *L*. *pneumophila* interferes with the function of a soluble lytic murein transglycosylase, an enzyme responsible for cleaving the glycosidic bond between *N*-acetylmuramic acid and *N*-acetylglucosamine of PG. In growing and replicating bacteria, the activities of lytic transglycosylases are required for insertion of new cell wall material, and to form localized gaps in PG that facilitate assembly of complex structures that transverse the cell envelope, such as type III and type IV secretions systems, and flagella [101]. EnhC is thought to benefit intracellular growth of *L*. *pneumophila* by inhibiting release of cell wall disaccharide-peptide fragments that can be recognized by the cytosolic innate immune sensor Nod1 [102–104]. The function of *C*. *burnetii enhC* in SCV biology is unknown; however, *cbu0925*, which encodes a membrane-bound lytic murein transglycosylase B, was also induced 10.1-fold. Thus, it is intriguing to speculate that *C*. *burnetii* EnhC regulates the activity of CBU0925.

A final upregulated gene associated with cell wall structure was *cbu0419* (up 5.9-fold) that encodes a carbohydrate esterase superfamily protein with a predicted polysaccharide deacetylase domain (Pfam1522) [105] (Fig 3). PG N-deacetylases (*pdg* genes) deacetylate N-acetylglucosamine of PG at the C-2 position [106, 107]. This modification renders PG resistant to lysozyme digestion, thereby lowering release of PG muropeptide fragments that can be recognized by Nod proteins and enabling survival and replication of pathogenic bacteria in host cells [107–109]. Like PdgA (HP0310) from *Helicobacter pylori*, CBU0419 shares significant amino acid identity with only the catalytic domain of characterized Pdg’s with conservation of two zinc-binding histidine residues (His99 and His103) required for enzyme activity [109–111] (data not shown). Thus, *C*. *burnetii* EnhC and CBU0419 may work in concert to minimize release of PG fragments that engage NOD receptors and induce pro-inflammitory responses. How PG fragments might translocate from the *Coxiella*-containing vacuole into the cytosol is unclear; however, the process is known to occur via type IV secretion [112] and the activity of endolysosomal peptide transporters [113]. *C*. *burnetii* is resistant to lysozyme [114] and proliferates in phagolysosome-like vacuole that degrades *E*. *coli* in 15 min [4]. Thus, cell wall modifications such as deacetylation may protect against lysosomal degradation and promote long-term intracellular survival of stationary phase SCV.

### Validation of microarray results for cell wall remodeling genes

**Fig 5:**
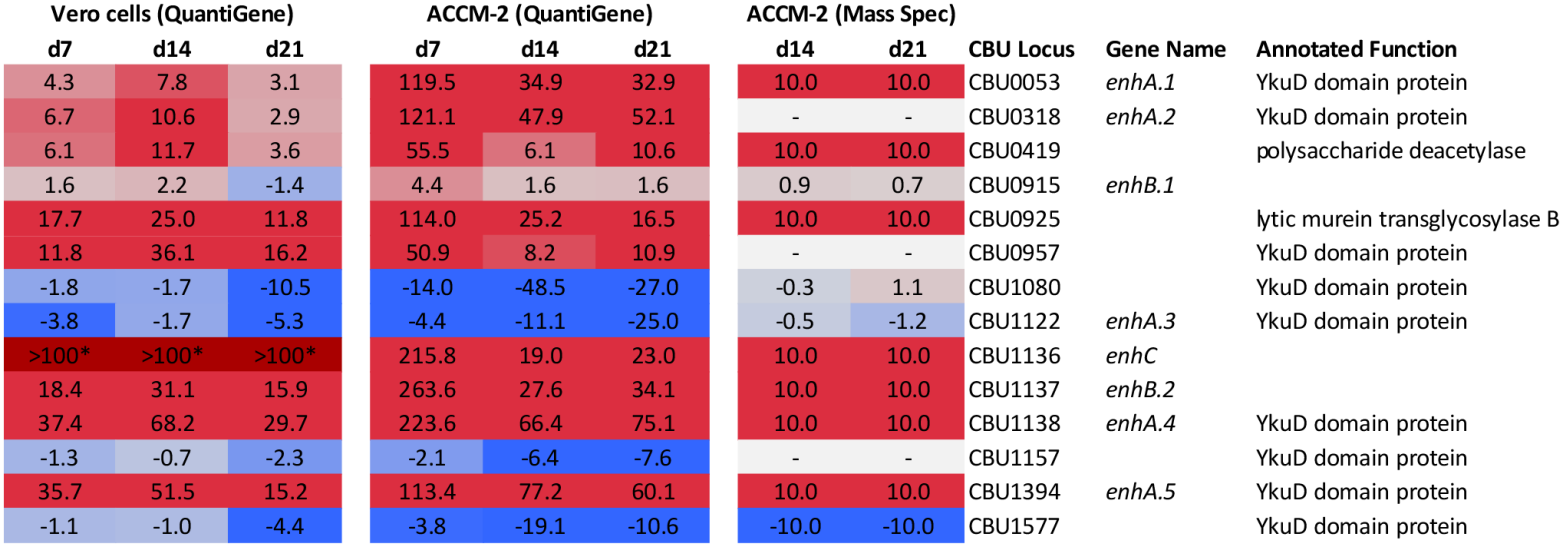
QuantiGene and mass spectometry verification of gene expression detected by microarray for cell wall remodeling genes. RNA from *Coxiella*-infected Vero cells or bacteria grown in ACCM-2 was subjected to QuantiGene analysis to determine expression levels. Fold-increase in mRNA levels at 5, 7, 14, and 21 days post-infection relative to 3 days post-infection with a corresponding color code is depicted. A value of >100 indicates signal was not detected with the day 3 RNA samples. Mass spectrometry of *C*. *burnetii* grown in ACCM-2 for 3, 14, and 21 days. Depicted are normalized peptide scores. Relative expression of protein at 14 and 21 days post-infection is shown as log2 ratio compared to 3 days post-infection. A value of 10 indicates no peptide was detected in day 3 bacteria. A value of -10 indicates no peptide was detected in day 14 and day 21 bacteria. A minus sign indicates no peptide was detected at any time point.

Based on the remarkable upregulation of cell remodeling genes in the SCV microarray, we chose to validate this transcriptional behavior using independent methods. QuantiGene analysis was conducted on fresh RNA samples derived from Vero cells infected with *C*. *burnetii* for 3, 7, 14, and 21 days. Because developmental changes in media-grown bacteria mimic those of host cell-grown organisms [23], we also analyzed RNA samples from *C*. *burnetii* cultured in the axenic medium ACCM-2 over the same time course. Additionally, whole bacterial cell LC-MS/MS was conducted on *C*. *burnetii* cultured in ACCM-2 for 3 (early LCV), 14 (early SCV), and 21 (late LCV) days [33]. Cell wall remodeling genes of host cell and axenically-grown *Coxiella* exhibited a similar transcriptional profile during SCV development as shown by microarray (Fig 5). Differences in the degree of differential expression between the two data sets likely reflect dynamic range differences between microarray and QuantiGene signals, and the variability inherent in using independent samples. Transcription strongly correlated with protein synthesis (Fig 5 and S4 Table). Notable exceptions were CBU0318 and CBU0957, which were undetectable in axenically cultured SCV, despite abundant transcript. Synthesis of these two enzymes may be post-transcriptionally regulated to result in PG modifications that occur only during intracellular growth. Indeed, this cell wall remodeling behavior has been demonstrated in *Salmonella enterica* serovar Typhimurium which specifically alters its PG structure during growth in epithelial cells to increase the percentage of dimeric cross-linked muropeptides missing one of the two *N*-acetylglucosamine-β-(1,4)-*N*-acetylmuramic acid moieties [115].

### SCV have abundant 3–3 muropeptide crosslinks and an unusually thick cell wall-outer membrane complex

**Fig 6:**
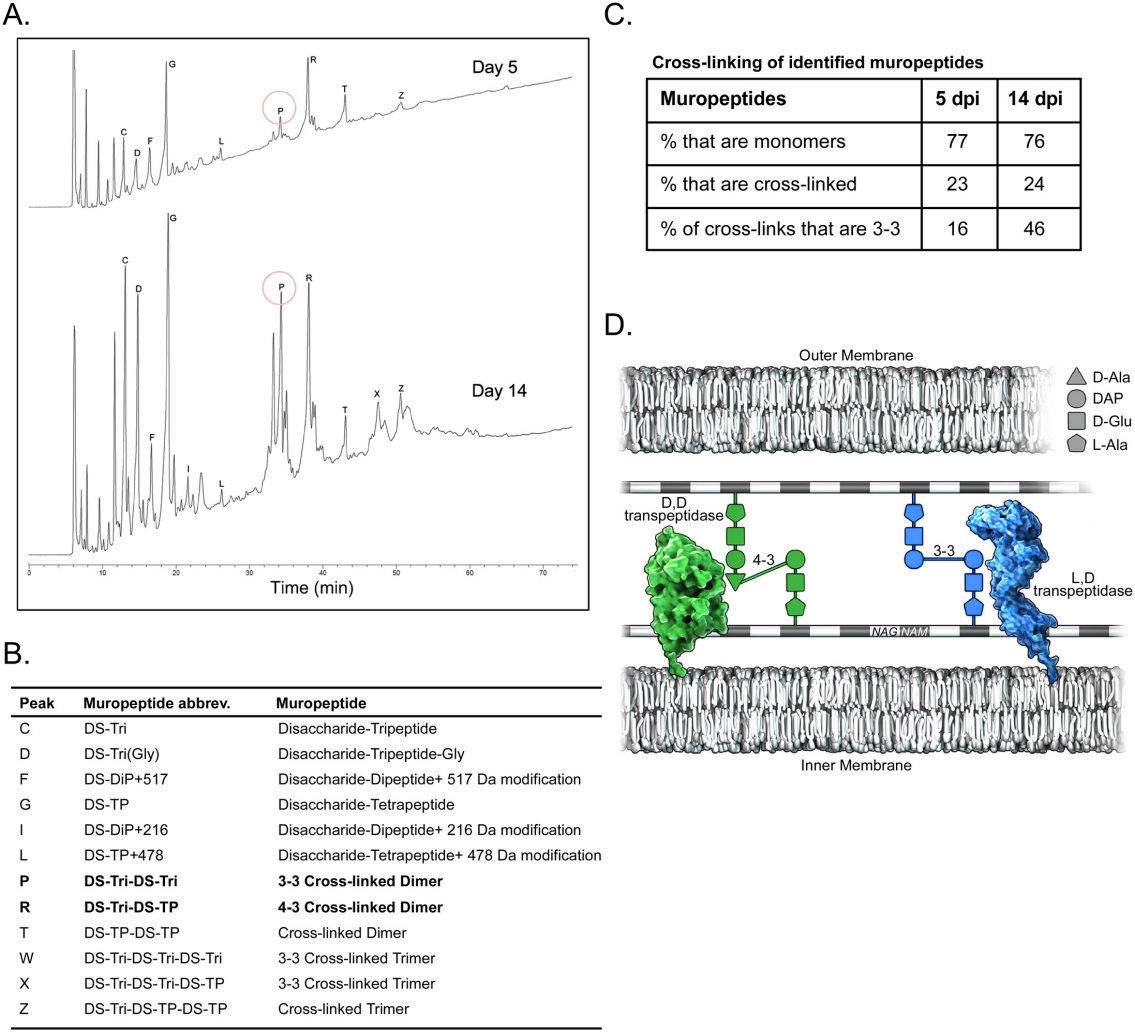
Muropeptide analysis of *C*. *burnetii* peptidoglycan. (A) HPLC elution profiles of muropeptides of LCV and SCV purified from Vero cells at 5 and 14 days post-infection (dpi), respectively (B) Muropeptides identified by MS and corresponding HPLC peaks in panel (A). (C) Molar percentage of muropeptides in monomer or cross-linked forms. The percentage of 3–3 cross-linked forms is also shown. (D) Schematic of peptidoglycan containing classical 4–3 and non-classical 3–3 cross-links that correspond to peaks R and P, respectively in panel (A). Unlinked stem peptides that are anchored to NAM residues have been removed for clarity. Abbreviations: NAG, *N*-acetylglucosamine; NAM, *N*-acetylmuramic acid; DAP, diaminopimelate.

The marked up-regulation of genes encoding YkuD family proteins with predicted L,D transpeptidase activity prompted us to characterize the PG muropeptide composition of LCV and SCV harvested from Vero cells after growth for 5 and 14 days, respectively. Overall, identified muropeptides of LCV and SCV showed roughly the same degree of cross-linking (23 versus 24%). This percentage is on the low end for Gram-negative bacteria, which display considerable structural variability in PG, and resembles that of *Helicobactor pylori* [116]. Cross-linking results contrast with an early study of the *C*. *burnetii* Ohio strain where chemical analysis of bacteria purified from embryonated eggs suggested 40% muropeptide cross-linking [117]. Of total cross-linked muropeptides, 46% of SCV were 3–3 cross-linked whereas only 16% of LCV were 3–3 cross-linked (Fig 6). Thus, L,D transpeptidase up-regulation by the SCV correlates with substantially increased PG 3–3 cross-linking. Cross-linked trimeric muropetides, suggestive of a multi-layered cell wall [116], were also more prevalent in SCV (9%) than LCV (3%). Additional muropeptide structures distinguished SCV from LCV, such as a disaccharide with an unknown modification of 216 Da (peak I) (Fig 6).

**Fig 7:**
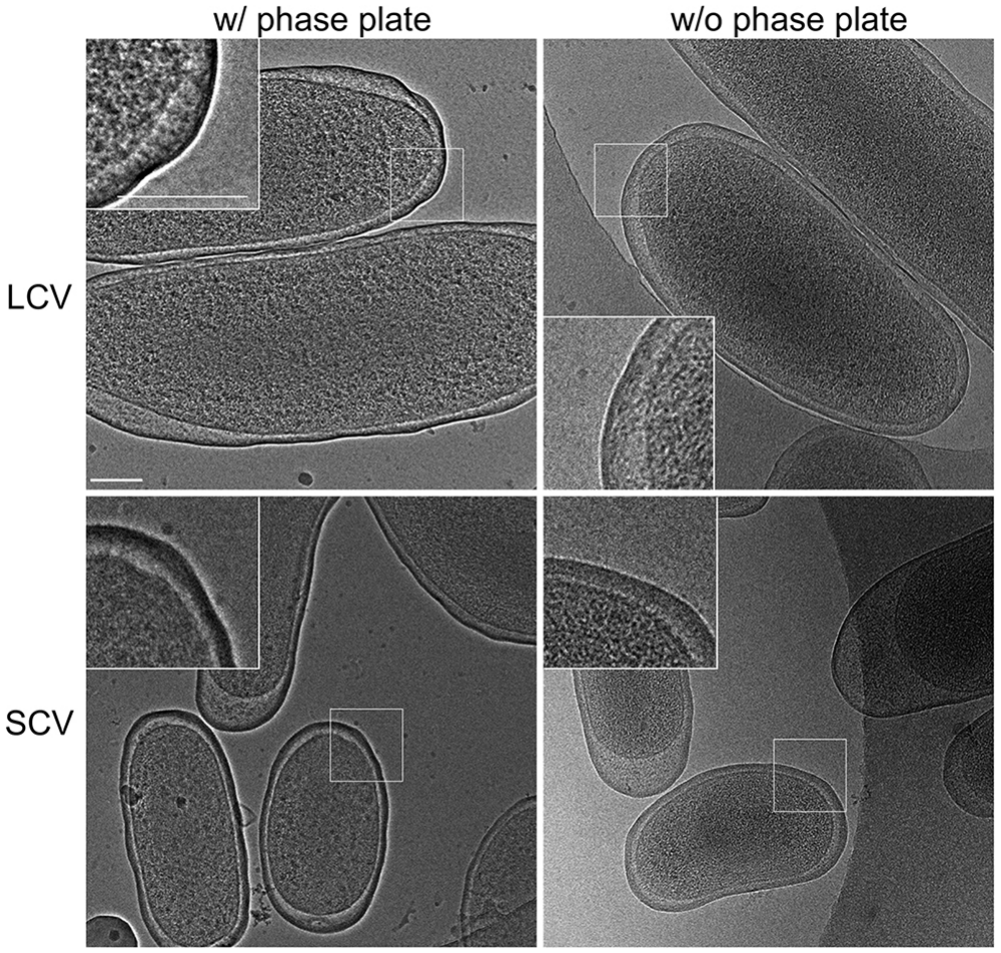
Ultrastucture of unfixed LCV and SCV imaged by cryo-electron microscopy. Representative images of LCV and SCV after 4 and 28 days of growth, respectively. A thicker dense cell wall/outer membrane complex distinguishes SCV from LCV. Scale bar, 100 nm.

Previous chemical analyses of cell envelope fractions indicate SCV contain 2.7-fold more PG than LCV on a dry weight basis [19, 118, 119]. Furthermore, conventional EM using fixed bacteria has demonstrated SCV with a prominent dense layer beneath the outer membrane proposed to be PG [19, 118, 119]. Chemical fixatives and heavy metal stains used in conventional TEM can induce artifacts in PG appearance [120]. Therefore, we employed CEM on LCV and SCV after 4 and 28 days of growth, respectively, to gain a high-resolution image of the native *C*. *burnetii* cell envelope [121]. Consistent with earlier studies, CEM revealed a dense layer of unusual thickness, presumably comprised of PG, underlying the outer membrane of SCV, but not LCV (Fig 7). Collectively, mass spectrometry and EM data indicate SCV synthesize an unusually structured cell wall with resistance properties atypical of Gram-negative bacteria.

## Summary

As a stationary phase SCV, *C*. *burnetii* has the extraordinary ability to persist for several weeks in a phagolysosome-like vacuole without losing viability. Remarkable hardiness is also witnessed in the extracellular environment where *C*. *burnetii* survival has been referred to as spore-like [1], a property also speculated to reside with the SCV developmental form. By profiling the transcriptome of *C*. *burnetii* during biphasic development, we revealed molecular determinants of SCV differentiation. SCV development clearly involves responses to protect against oxidative and nutritional stress. Moreover, developmentally regulated remodeling of the PG is a signature of the SCV predicted to promote both intracellular and extracellular survival. Polysaccharide deacetylase activity of CBU0419 would render PG resistant to lysozyme, and in concert with the predicted activity of EnhC, lower release of PG fragments that might be recognized by the cytosolic innate immune system. Generation of PG 3–3 cross-links by L,D transpeptidases, along with construction of the unique cell wall structure visualized by CEM, are alterations predicted to increase the physical resistance of SCV. Several detected but uncharacterized PG modifications of SCV may also play important biological and structural roles. Technical constraints precluded generation of sufficient early LCV (3 days post-infection) for PG and CEM analysis, which may have revealed more dramatic differences between cell walls of LCV and SCV. Nonetheless, the developmentally regulated PG remodeling and stress response genes identified here are attractive candidates for inactivation studies to more precisely define the unique biology of the *C*. *burnetii* SCV.

## Supporting Information

S1 Table Expression levels of *C*. *burnetii* genes as determined by microarray. The microarray signal associated with each gene at 5, 7, 14, and 21 days post-infection was normalized to the signal generated with the 3 days post-infection sample. Bold and italicized signals are statistically significant (*P* <0.05) with respect the 3 days post-infection signal.
(PDF)

S2 Table Predicted amino acid transporter proteins in *C*. *burnetii* RSA493. (PDF)

S3 Table Molecular weights of hypothetical proteins encoded by genes upregulated >5-fold in 21 day SCV. (PDF)

S4 Table Mass spectrometry of *C*. *burnetii* RSA493. (PDF)
