# Supplementary material for: Transcriptional Profiling of Coxiella burnetii Reveals Extensive Cell Wall Remodeling in the Small Cell Variant Developmental Form
Source: PLoS One. 2016 Feb 24;11(2):e0149957. doi: 10.1371/journal.pone.0149957 (PMC4766238; doi:10.1371/journal.pone.0149957)
Supplement: S1 Table — The microarray signal associated with each gene at 5, 7, 14, and 21 days post-infection was normalized to the signal generated with the 3 days post-infection sample. Bold and italicized signals are statistically significant (P <0.05) with respect the 3 days post-infection signal. (PDF) [file pone.0149957.s001.pdf]

NCBI RSA 493 mapping	NCBI syn	Description	5	7	14	21
CBU_0001	dnaA	chromosome replication initiator DnaA	-1.03	-1.15	-1.15	-1.23
CBU_0002	dnaN	DNA polymerase III subunit beta	1.17	1.26	1.13	1.13
CBU_0003	recF	DNA replication and repair protein RecF	1.20	-1.16	-1.20	-1.34
CBU_0004	gyrB	DNA gyrase subunit B	***1.30***	***1.52***	***1.68***	***2.64***
CBU_0006	-	IS1111A transposase	-1.23	-1.26	-1.61	-1.39
CBU_0007	-	hypothetical protein	-1.12	***1.34***	***1.89***	***2.72***
CBU_0007a	-	hypothetical protein	-1.16	1.46	***2.69***	***4.44***
CBU_0008	-	hypothetical protein	1.52	1.01	-1.11	-1.36
CBU_0008a	-	hypothetical protein	1.09	-1.11	-1.36	***-1.86***
CBU_0009	dacB	D-alanyl-meso-diaminopimelate endopeptidase	-1.06	1.04	-1.29	-1.35
CBU_0010	aroE	shikimate 5-dehydrogenase	-1.83	-1.82	***-2.32***	***-3.55***
CBU_0011	-	hypothetical protein	-1.19	-1.79	-1.52	-1.41
CBU_0014	-	low-affinity inorganic phosphate transporter	-1.09	-1.20	1.00	-1.05
CBU_0015	-	hypothetical protein	-1.20	-1.15	***-1.30***	***-1.66***
CBU_0016	xapA	purine nucleoside phosphorylase	1.01	-1.13	-1.22	-1.43
CBU_0017	deoC	deoxyribose-phosphate aldolase	1.04	-1.17	-1.04	-1.07
CBU_0018	-	hypothetical protein	***-2.38***	***-1.77***	***-1.38***	***-1.42***
CBU_0019	-	hypothetical protein	***5.29***	***6.96***	***10.96***	***14.00***
CBU_0020	-	ribosomal subunit interface protein domain/'cold-shock' DNA-binding domain protein	***3.94***	***6.32***	***8.24***	***10.51***
CBU_0021	-	hypothetical protein	***1.44***	***1.55***	***1.79***	***2.22***
CBU_0022	-	hypothetical protein	***1.66***	1.22	1.29	1.44
CBU_0023	-	hypothetical protein	***1.44***	1.19	1.25	***1.68***
CBU_0024	csrA-1	carbon storage regulator	***-2.16***	***-1.65***	***-2.19***	***-2.47***
CBU_0025	-	hypothetical protein	***-2.18***	***-1.54***	***-2.37***	***-3.04***
CBU_0026	rpiA	ribose-5-phosphate isomerase A	-1.04	1.09	1.12	-1.02
CBU_0027	-	acyltransferase	1.26	-1.20	-1.29	-1.27
CBU_0029	-	1-acyl-sn-glycerol-3-phosphate acyltransferase	-1.35	-1.34	***-1.81***	***-2.42***
CBU_0030	-	hydroxymethylglutaryl-coenzyme A reductase	-1.46	-1.31	***-2.01***	***-3.00***
CBU_0031	-	short chain dehydrogenase/reductase oxidoreductase	-1.19	-1.06	-1.07	-1.18
CBU_0032	-	hypothetical protein	1.31	1.13	1.43	-1.07
CBU_0033	-	4'-phosphopantetheinyl transferase	1.02	-1.09	-1.24	-1.36
CBU_0034	-	acyl carrier protein	-1.04	-1.05	-1.15	-1.03
CBU_0035	-	beta-ketoacyl-ACP synthase	1.25	-1.14	-1.21	-1.25
CBU_0036	-	beta-hydroxyacyl-ACP dehydratase	1.01	-1.41	-1.35	***-1.52***
CBU_0037	fabA	3-hydroxydecanoyl-ACP dehydratase	1.21	-1.25	-1.38	***-1.70***
CBU_0037a	-	hypothetical protein	1.09	2.12	1.28	1.84
CBU_0038	-	3-oxoacyl-ACP synthase	1.03	-1.17	-1.43	***-1.94***
CBU_0039	prlC	oligopeptidase A	-1.24	-1.16	-1.23	***-1.65***
CBU_0041	-	hypothetical protein	1.44	1.29	1.11	1.13
CBU_0042	hemH	ferrochelatase	***-2.55***	***-1.89***	***-2.80***	***-4.55***
CBU_0043	-	deoxyribonucleotide triphosphate pyrophosphatase	***1.40***	1.05	1.06	1.14
CBU_0044	-	hypothetical protein	***1.35***	***1.42***	***1.63***	***1.93***
CBU_0045	-	lipoprotein	1.03	***1.37***	1.29	1.18
CBU_0048	-	auxiliary transport protein, membrane fusion protein family	1.73	1.83	***2.32***	***2.30***
CBU_0049	-	hypothetical protein	***-2.00***	***-1.46***	***-1.87***	***-2.00***
CBU_0050	-	hypothetical protein	-1.17	-1.10	-1.28	***-1.85***
CBU_0051	-	hypothetical protein	***-1.79***	***-1.45***	***-1.40***	***-1.66***
CBU_0053	enhA.1	enhanced entry protein	***4.44***	***5.45***	***7.18***	***9.34***
CBU_0054	-	chorismate--pyruvate lyase	-1.24	-1.11	-1.06	-1.15
CBU_0055	ubiA	4-hydroxybenzoate polyprenyltransferase	***-1.53***	-1.44	-1.31	***-1.69***
CBU_0056	-	TolC family type I secretion outer membrane protein	***1.50***	***1.51***	***1.72***	***1.85***
CBU_0057	-	hypothetical protein	1.59	1.19	-1.02	1.07
CBU_0058	-	hypothetical protein	2.05	1.43	1.50	2.20
CBU_0062	-	DnaJ domain-containing protein	1.43	1.57	***1.62***	***1.94***
CBU_0063	kdtA	3-deoxy-D-manno-octulosonic-acid transferase	***-1.76***	-1.25	***-1.61***	***-2.33***
CBU_0064	parE	DNA topoisomerase IV subunit B	-1.02	-1.09	-1.19	***-1.74***
CBU_0065	-	rhodanese domain-containing protein	-1.04	-1.13	***-1.44***	***-1.65***
CBU_0065a	-	hypothetical protein	1.22	1.01	1.05	1.02
CBU_0066	-	5,10-methenyltetrahydrofolate synthetase	***1.46***	***1.33***	***1.83***	***2.64***
CBU_0067	-	hypothetical protein	-1.26	-1.19	-1.11	***-1.50***
CBU_0068	-	hypothetical protein	***-1.50***	-1.29	***-1.71***	***-2.40***
CBU_0068a	-	hypothetical protein	1.11	-1.75	***-2.10***	-1.80
CBU_0072	-	ankyrin repeat-containing protein	1.61	1.21	1.38	***1.53***
CBU_0073	-	peptidase, M24 family	-1.09	-1.03	1.12	-1.08
CBU_0074	-	hypothetical protein	1.09	-1.07	1.18	1.02
CBU_0075	ubiH	2-polyprenyl-6-methoxyphenol 4-hydroxylase	-1.06	-1.30	***-1.31***	***-1.53***
CBU_0076	visC	ubiquinone biosynthesis hydroxylase, UbiH/UbiF/VisC/COQ6 family	-1.24	-1.41	-1.32	***-1.50***
CBU_0077	-	hypothetical protein	-1.27	1.04	-1.05	-1.28
CBU_0081	proS	prolyl-tRNA synthetase	-1.03	1.06	-1.03	-1.08
CBU_0083	-	hypothetical protein	1.72	-1.01	-1.09	1.22

6
5
4
3
2
1
-1
-2
-3
-4
-5
-6

NCBI RSA 493 mapping	NCBI syn	Description	5	7	14	21
CBU_0084	-	sulfatase	***-2.12***	-1.21	1.15	-1.01
CBU_0085	-	entericidin B	***-1.48***	1.05	1.09	-1.02
CBU_0086	-	amidohydrolase	-1.09	-1.04	1.13	1.10
CBU_0087	-	phosphoglycerol transferase MdoB-like protein	***2.25***	***2.89***	***3.93***	***4.67***
CBU_0089	-	hypothetical protein	1.17	-1.04	1.08	1.30
CBU_0090	tolB	TolB	1.12	***1.63***	***2.46***	***3.88***
CBU_0091	-	peptidoglycan-associated lipoprotein	-1.08	1.27	1.30	***1.65***
CBU_0092	ybgF	tol-pal system protein YbgF	1.16	1.24	***1.56***	***1.86***
CBU_0093	queE	radical SAM domain-containing protein	1.07	-1.11	1.02	1.05
CBU_0094	clpB	ATP-dependent chaperone ClpB	***-1.63***	1.17	1.31	1.37
CBU_0095	-	sodium-calcium exchanger	-1.10	-1.21	-1.36	***-1.59***
CBU_0095a	-	acetyltransferase	1.04	1.21	***1.48***	***1.82***
CBU_0096	clS	cardiolipin synthetase	***1.44***	1.08	1.18	1.15
CBU_0097	pssA	CDP-diacylglycerol--serine O-phosphatidyltransferase	1.13	-1.04	***-1.56***	***-1.79***
CBU_0098	nadC	carboxylating nicotinate-nucleotide pyrophosphorylase	-1.27	1.21	1.42	1.11
CBU_0098a	-	hypothetical protein	-1.32	***-1.74***	***-1.73***	***-1.84***
CBU_0098b	-	hypothetical protein	1.62	1.22	1.24	1.51
CBU_0099	sixA	phosphohistidine phosphatase SixA	***-1.87***	-1.31	***-1.56***	***-2.13***
CBU_0100	-	lipoprotein	-1.32	-1.23	-1.27	***-1.55***
CBU_0101	nadB	L-aspartate oxidase	-1.14	-1.20	-1.17	***-1.53***
CBU_0102	-	hypothetical protein	-1.20	-1.20	-1.14	***-1.57***
CBU_0103	-	M20/M25/M40 family peptidase	-1.42	-1.19	-1.54	***-2.53***
CBU_0106	-	hypothetical protein	***-2.42***	-1.53	-1.04	-1.09
CBU_0107	-	D-methionine ABC transporter ATP-binding protein	-1.22	-1.14	***-1.53***	-1.35
CBU_0108	-	D-methionine ABC transporter permease	1.03	-1.03	-1.02	-1.01
CBU_0109	-	D-methionine ABC transporter periplasmic D-methionine-binding protein	-1.03	-1.27	***-2.11***	-1.48
CBU_0110	-	hypothetical protein	1.44	2.34	1.99	1.88
CBU_0110a	-	hypothetical protein	1.35	-1.45	-1.40	-1.35
CBU_0111	kbl	2-amino-3-ketobutyrate CoA ligase	-1.24	-1.04	-1.18	-1.62
CBU_0112	tdh	L-threonine 3-dehydrogenase	1.11	-1.08	-1.10	-1.18
CBU_0113	-	hypothetical protein	1.19	1.07	1.07	1.20
CBU_0114	-	nucleotide-binding protein	***-1.50***	-1.08	-1.25	***-1.64***
CBU_0115	mraZ	cell division protein MraZ	***1.55***	***1.35***	***1.57***	***2.04***
CBU_0116	mraW	S-adenosyl-methyltransferase MraW	***1.43***	***1.30***	***1.54***	***2.08***
CBU_0117	ftsL	cell division protein	***1.36***	1.23	***1.31***	***1.55***
CBU_0118	pbpB	penicillin-binding protein	1.04	-1.18	-1.18	-1.08
CBU_0119	-	hypothetical protein	-1.28	-1.14	-1.01	-1.59
CBU_0121	-	hypothetical protein	1.49	1.10	-1.06	1.13
CBU_0122	-	hypothetical protein	1.08	1.31	1.19	1.18
CBU_0123	murE	UDP-N-acetylmuramoylalanyl-D-glutamate--2,6-diaminopimelate ligase	1.04	-1.10	-1.27	***-1.49***
CBU_0124	murF	UDP-N-acetylmuramoyl-tripeptide--D-alanyl-D-alanine ligase	***-1.38***	-1.27	***-1.54***	***-2.02***
CBU_0125	mraY	phospho-N-acetylmuramoyl-pentapeptide-transferase	-1.22	-1.39	-1.88	***-3.02***
CBU_0127	-	hypothetical protein	-2.46	-2.90	-1.44	-1.57
CBU_0130	-	hypothetical protein	1.02	-1.42	-1.57	-1.99
CBU_0131	murD	UDP-N-acetylmuramoyl-L-alanyl-D-glutamate synthetase	***-1.42***	-1.09	-1.14	***-1.45***
CBU_0132	ftsW	cell division protein	-1.06	-1.12	-1.01	-1.26
CBU_0135	murG	undecaprenyldiphospho-muramoylpentapeptide beta-N- acetylglucosaminyltransferase	1.27	1.21	1.06	-1.10
CBU_0136	murC	UDP-N-acetylmuramate--L-alanine ligase	1.05	-1.06	1.00	-1.04
CBU_0137	murB	UDP-N-acetylenolpyruvoylglucosamine reductase	1.25	-1.01	-1.02	-1.17
CBU_0138	ftsQ	cell division protein	1.58	-1.16	-1.00	-1.15
CBU_0139	-	carbonate dehydratase	-1.35	-1.18	-1.22	-1.48
CBU_0140	ftsA	cell division protein	***1.30***	1.04	1.07	1.08
CBU_0141	ftsZ	cell division protein FtsZ	1.35	1.14	1.17	1.11
CBU_0142	lpxC	UDP-3-O-[3-hydroxymyristoyl] N-acetylglucosamine deacetylase	1.03	1.07	1.06	1.00
CBU_0143	-	hypothetical protein	-1.42	-1.24	-1.08	-1.24
CBU_0146	-	hypothetical protein	***-1.40***	***-1.44***	***-1.89***	***-2.36***
CBU_0147	secA	preprotein translocase subunit SecA	-1.40	-1.32	-1.44	***-2.30***
CBU_0148	mutT	7,8-dihydro-8-oxoguanine-triphosphatase	-1.34	***-1.53***	***-1.98***	***-2.69***
CBU_0149	-	hypothetical protein	1.18	-1.04	1.03	1.00
CBU_0150	-	hypothetical protein	1.29	1.19	-1.04	1.01
CBU_0151	queF	7-cyano-7-deazaguanine reductase	1.35	1.35	1.19	1.25
CBU_0152	coaE	dephospho-CoA kinase	-1.13	-1.21	-1.18	-1.36
CBU_0153	pilD	type 4 prepilin peptidase	-1.06	-1.03	1.03	-1.14
CBU_0154	pilC	type 4 pili biogenesis protein (plasma membrane protein)	-2.02	-1.60	-1.57	-1.21
CBU_0155	pilB	type 4 pili biogenesis protein (nuleotide-binding protein)	-1.02	-1.34	-1.88	***-3.10***
CBU_0156	pilA	type IV-A pilin protein PilA	-1.61	-1.38	-1.53	***-1.69***
CBU_0156a	-	phage integrase	-1.23	-1.56	***-1.73***	***-2.55***
CBU_0157	-	hypothetical protein	-1.31	1.17	1.14	1.07
CBU_0158	-	CRISPR-associated Csy4 family protein	-1.40	-1.34	-1.23	-1.22
CBU_0159	-	CRISPR-associated Cas5e family protein	-1.56	-1.46	-1.22	1.18

NCBI RSA 493 mapping	NCBI syn	Description	5	7	14	21
CBU_0165	-	hypothetical protein	-1.02	-1.30	-1.08	1.03
CBU_0166	-	hypothetical protein	1.07	-1.22	1.10	1.86
CBU_0167	pksH	enoyl-CoA hydratase	1.39	1.38	1.43	***2.04***
CBU_0168	-	acyl carrier protein	2.14	1.56	1.77	***1.95***
CBU_0173	-	hypothetical protein	1.46	1.94	***2.10***	***2.05***
CBU_0175	-	serine/threonine kinase	-1.29	-1.30	***-1.37***	***-1.56***
CBU_0176	degP.1	serine protease domain-containing protein	***8.38***	***10.33***	***14.22***	***20.12***
CBU_0177	-	quaternary amine ABC transporter permease/periplasmic amine-binding protein	***1.59***	1.12	1.27	1.21
CBU_0178	-	quaternary amine ABC transporter ATP-binding protein	1.32	1.03	1.08	1.24
CBU_0179	anmK	anhydro-N-acetylmuramic acid kinase	1.31	-1.20	-1.25	-1.48
CBU_0180	-	peptidase M23/M37 domain-containing protein	1.24	1.12	1.11	1.03
CBU_0181	tyrS	tyrosyl-tRNA synthetase	-1.13	-1.13	1.02	1.06
CBU_0181a	-	hypothetical protein	***5.69***	***5.14***	***4.18***	***5.93***
CBU_0182	-	homing endonuclease, 23S rRNA intron	1.18	-1.08	-1.08	1.22
CBU_0183	-	hypothetical protein	-1.44	-1.34	-1.26	-1.26
CBU_0184	-	hypothetical protein	-1.68	-1.62	-1.43	-1.24
CBU_0193	-	hypothetical protein	***3.82***	***4.71***	***7.31***	***9.56***
CBU_0194	sda	L-serine ammonia-lyase	1.28	1.17	***1.34***	***1.44***
CBU_0195	ampG	AmpG	-1.07	-1.45	1.05	1.04
CBU_0196	-	hypothetical protein	1.01	1.14	1.42	***1.43***
CBU_0197	-	hypothetical protein	1.31	1.08	1.07	1.08
CBU_0198	-	hypothetical protein	1.20	-1.05	1.19	1.15
CBU_0199	coaA	pantothenate kinase	-1.22	-1.15	***-1.43***	***-1.44***
CBU_0200	-	aldose 1-epimerase	1.09	-1.09	-1.24	***-1.38***
CBU_0201	-	ankyrin repeat-containing protein	-1.01	-1.05	***-1.28***	***-1.82***
CBU_0205	gltX	glutamyl-tRNA synthetase	***-1.69***	***-1.61***	***-1.93***	***-2.48***
CBU_0206	-	hypothetical protein	***2.34***	***2.07***	***2.34***	***2.67***
CBU_0207	-	hypothetical protein	1.03	1.13	***1.51***	***1.87***
CBU_0209	-	hypothetical protein	-1.35	-1.20	***-2.06***	-1.90
CBU_0210	-	hypothetical protein	1.37	1.02	-1.05	-1.09
CBU_0213	-	hypothetical protein	-1.12	1.19	1.46	1.16
CBU_0214	-	hypothetical protein	-1.13	1.08	-1.01	***-1.46***
CBU_0215	-	NlpC-P60 family protein	1.09	1.01	-1.06	-1.22
CBU_0217a	-	hypothetical protein	1.25	1.06	2.54	2.17
CBU_0218	cydA-1	cytochrome d ubiquinol oxidase subunit I	1.60	1.32	1.24	1.01
CBU_0220	-	hypothetical protein	-1.50	-1.20	-1.17	***-1.46***
CBU_0221	-	phosphopantethiene-protein transferase domain-contain protein	-1.46	-1.17	-1.23	-1.36
CBU_0221b	-	elongation factor Tu	1.00	1.07	-1.10	-1.04
CBU_0224	secE	protein translocase subunit	1.04	1.13	-1.15	-1.02
CBU_0225	nusG	transcription termination/antitermination factor NusG	1.01	-1.01	-1.20	-1.16
CBU_0226	rplK	50S ribosomal protein L11	-1.04	1.04	-1.01	1.03
CBU_0227	rplA	50S ribosomal protein L1	-1.09	-1.08	-1.29	-1.30
CBU_0228	rplJ	50S ribosomal protein L10	-1.17	-1.21	-1.42	***-1.65***
CBU_0229	rplL	50S ribosomal protein L7/L12	-1.18	-1.09	-1.19	-1.10
CBU_0230	-	hypothetical protein	-1.06	1.03	-1.39	-1.14
CBU_0231	rpoB	DNA-directed RNA polymerase subunit beta	-1.08	-1.02	-1.14	-1.23
CBU_0232	rpoC	DNA-directed RNA polymerase subunit beta'	1.02	-1.01	-1.04	-1.07
CBU_0233	rpsL	30S ribosomal protein S12	-1.16	-1.09	***-1.56***	***-1.93***
CBU_0234	rpsG	30S ribosomal protein S7	-1.17	1.01	-1.19	-1.28
CBU_0235	fusA	elongation factor G	-1.17	-1.17	***-1.52***	***-1.63***
CBU_0237	rpsJ	30S ribosomal protein S10	-1.12	-1.14	***-1.49***	***-1.45***
CBU_0238	rplC	50S ribosomal protein L3	1.26	1.18	1.02	-1.02
CBU_0239	rplD	50S ribosomal protein L4	-1.07	-1.11	***-1.46***	***-1.59***
CBU_0240	rplW	50S ribosomal protein L23	-1.08	-1.10	-1.37	-1.35
CBU_0241	rplB	50S ribosomal protein L2	-1.27	-1.16	-1.38	***-1.56***
CBU_0242	rpsS	30S ribosomal protein S19	-1.05	-1.14	-1.30	-1.24
CBU_0243	rplV	50S ribosomal protein L22	-1.26	-1.10	-1.51	***-1.64***
CBU_0244	rpsC	30S ribosomal protein S3	-1.14	-1.25	-1.35	-1.33
CBU_0245	rplP	50S ribosomal protein L16	-1.10	-1.16	-1.20	-1.26
CBU_0246	rpmC	50S ribosomal protein L29	-1.21	-1.25	-1.30	-1.34
CBU_0247	rpsQ	30S ribosomal protein S17	-1.09	-1.08	***-1.38***	***-1.48***
CBU_0248	rplN	50S ribosomal protein L14	-1.04	-1.15	-1.31	-1.29
CBU_0249	rplX	50S ribosomal protein L24	-1.11	-1.17	-1.30	-1.27
CBU_0250	rplE	50S ribosomal protein L5	-1.00	1.01	-1.20	***-1.30***
CBU_0251	rpsN	30S ribosomal protein S14	-1.03	-1.02	-1.02	1.05
CBU_0252	rpsH	30S ribosomal protein S8	-1.04	-1.09	-1.28	-1.21
CBU_0253	rplF	50S ribosomal protein L6	-1.04	-1.05	-1.08	-1.14
CBU_0254	rplR	50S ribosomal protein L18	1.12	1.01	-1.10	-1.12
CBU_0255	rpsE	30S ribosomal protein S5	1.17	1.02	-1.19	-1.36
CBU_0256	rpmD	50S ribosomal protein L30	-1.00	-1.06	-1.14	-1.08

NCBI RSA 493 mapping	NCBI syn	Description	5	7	14	21
CBU_0257	rplO	50S ribosomal protein L15	1.07	-1.05	-1.10	-1.24
CBU_0258	secY	preprotein translocase subunit SecY	-1.12	-1.26	-1.18	-1.36
CBU_0259	-	hypothetical protein	-1.25	-1.30	-1.76	***-1.81***
CBU_0260	rpsM	30S ribosomal protein S13	-1.14	-1.03	-1.26	-1.26
CBU_0261	rpsK	30S ribosomal protein S11	-1.25	-1.05	-1.31	***-1.46***
CBU_0262	rpsD	30S ribosomal protein S4	-1.38	-1.20	-1.39	***-1.50***
CBU_0263	rpoA	DNA-directed RNA polymerase subunit alpha	-1.01	-1.09	-1.30	-1.37
CBU_0264	rplQ	50S ribosomal protein L17	-1.09	-1.26	-1.35	-1.33
CBU_0265	-	glucose/galactose transporter	-1.00	-1.22	-1.31	-1.61
CBU_0270	-	trans-2-enoyl-CoA reductase	***-1.73***	-1.23	***-1.76***	***-3.19***
CBU_0271	ssb	single-stranded DNA-binding protein	1.07	-1.05	-1.00	1.08
CBU_0272	-	MFS superfamily transporter	1.05	1.07	1.11	1.04
CBU_0273	-	hypothetical protein	-1.29	1.15	1.16	1.07
CBU_0274	uvrA	excinuclease ABC subunit A	1.49	1.31	***1.78***	***2.44***
CBU_0274a	-	hypothetical protein	1.33	1.13	1.42	***2.26***
CBU_0275	hemE	uroporphyrinogen decarboxylase	***2.35***	***2.12***	***2.80***	***3.51***
CBU_0276	-	rubredoxin-NAD(+) reductase	1.23	1.05	***1.53***	***1.96***
CBU_0277	-	hypothetical protein	1.37	1.10	1.18	1.24
CBU_0278	-	multiple antibiotic resistance protein MarC	-1.01	-1.11	-1.20	-1.41
CBU_0279	-	amidinotransferase	-1.10	1.02	1.06	***-1.41***
CBU_0280	dinP	DNA polymerase IV	-1.33	1.09	-1.12	-1.60
CBU_0282	-	transcriptional regulator	-1.18	-1.23	-1.39	-1.41
CBU_0283	-	hypothetical protein	1.31	1.21	1.16	1.30
CBU_0284	-	prophage protein gp49	***1.34***	1.00	-1.07	***1.19***
CBU_0285	-	hypothetical protein	1.15	1.09	-1.15	-1.04
CBU_0286	pcnB	poly(A) polymerase	***-1.87***	***-1.83***	***-2.24***	***-3.15***
CBU_0287	folK-2	2-amino-4-hydroxy-6-hydroxymethyldihydropteridine pyrophosphokinase	-1.81	***-2.10***	***-2.41***	***-2.95***
CBU_0288	coaD	phosphopantetheine adenylyltransferase	-1.37	-1.14	-1.37	***-1.67***
CBU_0289	-	ferredoxin	-1.03	***-1.31***	***-1.37***	***-1.32***
CBU_0290	rpmG	50S ribosomal protein L33	***-1.93***	-1.29	***-1.78***	***-2.54***
CBU_0291	rpmB	50S ribosomal protein L28	***-1.71***	-1.17	***-1.49***	***-2.30***
CBU_0291b	-	hypothetical protein	-1.53	1.01	1.61	1.21
CBU_0293	dut	deoxyuridine 5'-triphosphate nucleotidohydrolase	-1.18	1.02	***-1.55***	***-2.10***
CBU_0294	-	phosphoglucomutase	-1.49	-1.17	***-1.90***	***-2.59***
CBU_0295	-	hypothetical protein	1.41	-1.04	1.07	1.31
CBU_0296	pyrE	orotate phosphoribosyltransferase	***1.44***	1.20	***1.56***	***1.96***
CBU_0297	xth	exodeoxyribonuclease III	***-1.27***	-1.12	-1.10	***-1.37***
CBU_0298	murI	glutamate racemase	-1.15	1.06	-1.01	-1.28
CBU_0299	rph	ribonuclease PH	***-1.78***	-1.23	***-1.44***	***-1.74***
CBU_0300	-	hypothetical protein	-1.07	-1.07	-1.18	-1.58
CBU_0301	gmk	guanylate kinase	-1.17	-1.19	-1.38	***-1.78***
CBU_0302	rpoZ	DNA-directed RNA polymerase subunit omega	***1.77***	***1.44***	***1.70***	***2.24***
CBU_0303	spoT	GTP pyrophosphokinase	***1.40***	1.29	***1.81***	***2.23***
CBU_0304	-	endoribonuclease L-PSP	-1.09	1.04	-1.06	1.04
CBU_0305	recG	ATP-dependent DNA helicase RecG	1.05	1.06	***1.46***	***1.79***
CBU_0306	-	hypothetical protein	1.23	1.01	-1.02	1.24
CBU_0307	-	OmpA-like transmembrane domain-containing protein	1.23	1.03	1.04	1.23
CBU_0307a	-	hypothetical protein	-1.16	-1.45	-1.17	-1.40
CBU_0309	htpG	heat shock protein 90	***-2.20***	-1.02	1.10	1.11
CBU_0311	-	hypothetical protein	1.47	-1.03	1.03	***2.06***
CBU_0312	folD	bifunctional 5,10-methylene-tetrahydrofolate dehydrogenase/ 5,10-methylene-tetrahydrofolate	-1.34	-1.37	***-1.58***	***-2.26***
CBU_0312a	-	hypothetical protein	1.45	1.19	1.44	***1.73***
CBU_0313	-	Slt family transglycosylase	***-1.54***	-1.07	1.01	-1.16
CBU_0314	-	hydroxyacylglutathione hydrolase	1.58	***1.66***	***1.93***	***1.66***
CBU_0315	-	methyltransferase	-1.07	-1.23	-1.25	-1.53
CBU_0316	rnhA	ribonuclease H	-1.03	-1.02	-1.04	-1.09
CBU_0316a	-	hypothetical protein	1.37	1.07	***1.48***	***2.77***
CBU_0317	dnaQ	DNA polymerase III subunit epsilon	-1.21	-1.85	-1.74	***-1.88***
CBU_0318	enhA.2	ErfK/YbiS/YcfS/YnhG family protein	***6.77***	***7.68***	***9.48***	***12.07***
CBU_0319	-	hypothetical protein	1.68	1.51	1.64	1.87
CBU_0321	icmH	IcmH	-1.02	-1.01	1.04	-1.07
CBU_0322	-	hypothetical protein	1.06	-1.26	-1.33	***-1.74***
CBU_0323	-	acyl carrier protein	1.02	-1.22	-1.26	-1.37
CBU_0324	-	acyl-CoA synthetase	1.48	-1.07	1.20	1.41
CBU_0326	purD	phosphoribosylamine--glycine ligase	1.44	1.12	***1.75***	***1.85***
CBU_0330	thiC	thiamine biosynthesis protein ThiC	1.01	1.31	1.00	1.01
CBU_0331	-	glycine oxidase ThiO	-1.60	-1.00	-1.16	-1.25
CBU_0332	thiS	sulfur carrier protein ThiS	-1.26	-1.31	-1.50	-1.40
CBU_0333	thiG	thiazole synthase	1.10	-1.16	-1.18	-1.03
CBU_0334	thiDE	thiamine-phosphate pyrophosphorylase/thiamine-phosphate pyrophosphorylase	1.03	-1.06	1.01	-1.12

NCBI RSA 493 mapping	NCBI syn	Description	5	7	14	21
CBU_0335	-	acid phosphatase	-1.33	-1.15	-1.24	***-1.62***
CBU_0336	purH	bifunctional phosphoribosylaminoimidazolecarboxamide formyltransferase/IMP cyclohydrola	-1.21	***-1.58***	***-1.95***	***-2.56***
CBU_0337	fis	Fis family transcriptional regulator	-1.13	-1.13	-1.29	-1.35
CBU_0338	pepN	aminopeptidase	-2.01	-1.40	-1.62	-2.44
CBU_0339	-	hypothetical protein	1.04	1.03	1.04	-1.01
CBU_0340	-	hypothetical protein	-1.08	-1.73	-1.80	-1.78
CBU_0341	pfkA	6-phosphofructokinase	-1.04	-1.04	-1.20	***-1.58***
CBU_0342	-	hypothetical protein	-1.12	-1.25	-1.81	-1.42
CBU_0343	-	hypothetical protein	-1.31	-1.07	-1.09	-1.12
CBU_0346	xylB	xylulose kinase	1.56	-1.50	-1.15	-1.40
CBU_0347	-	D-xylose-proton symporter	-1.05	-1.17	***-1.53***	***-2.05***
CBU_0349	gph	phosphoglycolate phosphatase	-1.02	-1.34	-1.46	***-1.89***
CBU_0350	ubiG	3-demethylubiquinone-9 3-methyltransferase	-1.16	-1.14	***-1.27***	***-1.26***
CBU_0351	-	Hpt domain-containing protein	***-1.42***	-1.14	-1.28	***-1.43***
CBU_0352	czcD.1	cation efflux system protein CzcD	1.36	***1.47***	***1.49***	***1.39***
CBU_0353	-	periplasmic protease	1.12	-1.05	-1.06	-1.36
CBU_0354	-	amino acid permease	***1.74***	***1.79***	***2.30***	***2.91***
CBU_0357	-	hypothetical protein	-1.02	-1.48	1.01	1.28
CBU_0362	-	amidohydrolase	1.20	1.22	1.29	1.02
CBU_0364	-	phosphate transporter	1.53	1.17	-1.66	***-3.06***
CBU_0365	-	hypothetical protein	1.03	1.09	-1.21	-1.55
CBU_0366	phoR	hypothetical protein	1.01	1.36	1.05	-1.08
CBU_0367	phoB	PhoB family transcriptional regulator	-1.05	1.15	1.11	-1.20
CBU_0368	-	glucose/sorbosone dehydrogenase	-1.02	1.11	-1.10	***-1.41***
CBU_0369	-	hypothetical protein	***2.01***	1.64	1.73	***1.84***
CBU_0370	-	surface antigen	1.44	1.14	1.33	1.46
CBU_0371	-	multidrug resistance transporter, Bcr family	1.51	1.14	-1.03	-1.21
CBU_0372	-	Fic family protein	1.70	1.38	1.46	1.75
CBU_0373	-	acyl dehydratase MaoC	***1.72***	1.06	1.40	***1.61***
CBU_0377	-	hypothetical protein	1.02	1.10	-1.02	-1.18
CBU_0378	-	lipoprotein	***-2.12***	-1.21	***-2.12***	***-2.42***
CBU_0379	ampD	anhydro-N-acetylmuramyl-tripeptide amidase	-1.23	-1.06	1.32	1.18
CBU_0380	-	hypothetical protein	1.03	-1.04	1.10	1.17
CBU_0381	grpB	glutamate-rich protein	-1.07	-1.10	1.24	1.29
CBU_0382	ispB	polyprenyl synthetase	***-1.96***	***-1.84***	***-2.26***	***-3.76***
CBU_0383	tag	DNA-3-methyladenine glycosylase I	***-1.66***	-1.29	***-1.50***	***-2.19***
CBU_0384b	-	hypothetical protein	***1.52***	-1.14	-1.32	***-1.84***
CBU_0385	rplU	50S ribosomal protein L21	***-1.71***	-1.12	***-1.49***	***-1.91***
CBU_0386	rpmA	50S ribosomal protein L27	-1.20	-1.02	-1.20	***-1.40***
CBU_0387	obgE	GTPase ObgE	-1.23	-1.07	***-1.26***	***-1.31***
CBU_0388	-	hypothetical protein	1.03	1.04	1.23	***1.34***
CBU_0389	rpsT	30S ribosomal protein S20	***-1.43***	-1.24	***-1.79***	***-2.37***
CBU_0390	mviN	virulence factor	-1.35	1.10	1.31	1.10
CBU_0391	ribF	FMN adenylyltransferase	***-1.74***	-1.21	-1.28	***-1.69***
CBU_0394	-	hypothetical protein	1.80	1.22	***2.03***	***2.86***
CBU_0395	-	lipoprotein	-1.21	1.08	-1.08	-1.42
CBU_0396	ileS	isoleucyl-tRNA synthetase	-1.25	-1.40	***-1.95***	***-2.49***
CBU_0397	lspA	lipoprotein signal peptidase	1.05	-1.13	-1.45	***-1.58***
CBU_0398	-	hypothetical protein	1.24	1.23	***1.35***	1.25
CBU_0400	-	hypothetical protein	***1.54***	***1.63***	***2.05***	***2.24***
CBU_0400a	-	hypothetical protein	-1.32	-1.26	1.07	-1.06
CBU_0402	-	acetyltransferase	-1.32	-1.28	-1.05	-1.00
CBU_0403	-	acetyltransferase	-1.07	-1.16	-1.32	***-1.45***
CBU_0407	-	hypothetical protein	-1.14	-1.53	-1.09	1.02
CBU_0409	-	hypothetical protein	1.11	1.47	1.99	***2.96***
CBU_0410	-	hypothetical protein	1.13	1.61	***1.69***	***1.74***
CBU_0411	-	hypothetical protein	1.06	-1.29	1.17	1.04
CBU_0412	pilE	type IV pilin	-1.12	-1.20	-1.18	-1.05
CBU_0415	-	guanine deaminase	1.70	1.68	1.73	1.30
CBU_0416	-	hypothetical protein	-1.08	-1.21	-1.38	***-1.48***
CBU_0417	-	hypothetical protein	1.34	1.61	1.06	1.27
CBU_0418	-	branched-chain amino acid aminotransferase	1.08	***1.97***	***2.62***	***2.32***
CBU_0419	-	polysaccharide deacetylase	***4.19***	***5.18***	***7.13***	***5.87***
CBU_0422	panD	aspartate alpha-decarboxylase	***-1.66***	-1.25	***-1.42***	***-2.00***
CBU_0423	panC	pantoate--beta-alanine ligase	***-3.04***	-1.92	***-2.44***	***-3.63***
CBU_0424	panB	3-methyl-2-oxobutanoate hydroxymethyltransferase	***-1.46***	-1.42	***-1.89***	***-2.22***
CBU_0425	-	hypothetical protein	1.43	1.49	1.70	***2.37***
CBU_0426	-	amino acid permease	1.48	1.10	-1.42	***-2.39***
CBU_0429	-	peptidyl-tRNA hydrolase domain-containing protein	1.06	1.03	-1.26	-1.85
CBU_0430	lysS	lysyl-tRNA synthetase	***-1.64***	-1.33	***-1.60***	***-2.07***

NCBI RSA 493 mapping	NCBI syn	Description	5	7	14	21
CBU_0431	-	glycine cleavage T-protein (aminomethyl transferase) domain protein	-1.26	-1.18	-1.30	***-1.98***
CBU_0432	-	MFS superfamily transporter	***-1.79***	-1.26	***-1.66***	***-1.72***
CBU_0433	-	transporter, small conductance mechanosensitive ion channel (MscS) family	-1.33	-1.07	-1.09	***-1.53***
CBU_0434	-	hypothetical protein	1.34	-1.07	-1.05	1.19
CBU_0434a	-	hypothetical protein	1.29	-1.08	-1.16	1.11
CBU_0436	-	hypothetical protein	-2.67	-2.65	-2.28	***-3.00***
CBU_0439	-	hypothetical protein	-1.33	-1.54	***-2.98***	***-2.41***
CBU_0440	-	hypothetical protein	-1.25	-1.13	1.03	-1.07
CBU_0441	ogt	methylated-DNA-[protein]-cysteine S-methyltransferase	***-1.72***	-1.50	***-2.64***	***-3.37***
CBU_0442	rplS	50S ribosomal protein L19	***-2.16***	-1.56	***-2.22***	***-3.50***
CBU_0443	trmD	tRNA (guanine-N(1)-)-methyltransferase	-1.15	-1.75	-1.37	-1.62
CBU_0444	rimM	16S rRNA-processing protein RimM	-1.33	-1.03	***-1.42***	***-1.56***
CBU_0445	rpsP	30S ribosomal protein S16	***-1.66***	-1.24	***-1.69***	***-2.14***
CBU_0446	-	hypothetical protein	-1.39	-1.39	-1.33	***-1.69***
CBU_0447	-	ankyrin repeat-containing protein	***-1.81***	-1.21	-1.29	***-2.17***
CBU_0448	-	hypothetical protein	1.68	***2.14***	***2.54***	1.86
CBU_0450	ffh	signal recognition particle subunit FFH/SRP54	***-2.05***	***-1.53***	***-2.20***	***-2.51***
CBU_0451	-	cytochrome C assembly protein	***1.53***	1.16	1.12	1.06
CBU_0452	corB	transporter, HlyC/CorC (HCC) family	1.12	-1.13	-1.01	-1.31
CBU_0453	fimT	fimT protein	-1.14	-1.39	-1.52	***-1.95***
CBU_0454	adk	adenylate kinase	-1.19	-1.04	-1.07	-1.25
CBU_0455	-	thioredoxin	-1.00	1.14	1.14	1.09
CBU_0456	-	DNA-binding protein	1.37	1.11	1.11	***1.44***
CBU_0457	-	hypothetical protein	1.79	-1.51	-1.20	-1.55
CBU_0458	-	hypothetical protein	***-1.53***	-1.15	-1.21	***-1.50***
CBU_0459	rosB	transporter, monovalent cation:proton antiporter-2 (CPA2) family	***-1.58***	-1.36	-1.31	***-2.16***
CBU_0460	ampE	beta-lactamase induction protein	***-1.60***	-1.19	-1.36	***-1.93***
CBU_0461	aceE	pyruvate dehydrogenase subunit E1	***-1.93***	***-1.52***	***-1.69***	***-2.09***
CBU_0462	pdhC	dihydrolipoyllysine-residue acetyltransferase E2 component of pyruvate dehydrogenase comp	-1.19	-1.18	-1.21	-1.26
CBU_0463	lpdA	dihydrolipoyl dehydrogenase, E3 component of pyruvate dehydrogenases complex	-1.25	-1.21	-1.29	-1.35
CBU_0465	-	hypothetical protein	-1.50	***-1.75***	***-2.03***	***-2.40***
CBU_0467	bioC.1	methyltransferase	***-1.88***	-1.37	-1.45	***-1.94***
CBU_0468	xseB	exodeoxyribonuclease VII small subunit	***1.27***	1.18	***1.27***	***1.63***
CBU_0469	-	hypothetical protein	***3.40***	***3.12***	***3.84***	***5.52***
CBU_0470	-	phosphoribosyl transferase	***3.99***	***5.45***	***7.26***	***8.94***
CBU_0472	recQ	ATP-dependent DNA helicase	-1.11	-1.07	-1.11	-1.25
CBU_0473	-	DNA-binding protein BpH2	1.33	1.12	1.16	***1.31***
CBU_0474	-	hypothetical protein	1.34	1.18	1.42	***1.62***
CBU_0476	-	hypothetical protein	***1.68***	1.20	1.18	1.19
CBU_0477	-	hypothetical protein	***2.21***	***3.86***	***4.57***	***5.87***
CBU_0478	-	hypothetical protein	1.05	-1.26	-1.13	-1.13
CBU_0478a	-	hypothetical protein	-1.26	-1.01	-1.25	***-1.73***
CBU_0479	kdsB	3-deoxy-manno-octulosonate cytidylyltransferase	***-1.38***	-1.25	***-1.63***	***-2.01***
CBU_0480	argR	arginine repressor ArgR	1.25	***1.80***	***3.02***	***3.88***
CBU_0481	artP	arginine ABC transporter ATP-binding protein	***1.47***	***2.85***	***5.47***	***6.93***
CBU_0482	-	arginine ABC transporter substrate-binding protein	1.37	***1.82***	***2.36***	***2.67***
CBU_0483	artQ	arginine ABC transporter permease	1.11	1.42	***1.95***	***3.01***
CBU_0484	artM	arginine transport system permease	***-2.06***	-1.44	-1.28	-1.36
CBU_0485	-	hypothetical protein	-1.22	-1.20	-1.23	-1.11
CBU_0486	-	ribonuclease	***-1.94***	***-1.71***	***-2.04***	***-2.40***
CBU_0487	rluC	ribosomal large subunit pseudouridine synthase C	-1.17	-1.29	***-1.57***	***-1.96***
CBU_0488	-	Ser/Thr protein phosphatase	***2.72***	***2.99***	***4.63***	***6.32***
CBU_0489	-	phospholipase A1	-1.47	-1.36	***-1.62***	***-2.71***
CBU_0490	-	DNA polymerase X family/PHP domain-containing protein	***1.62***	***1.69***	***2.25***	***2.65***
CBU_0491	rpmF	50S ribosomal protein L32	***-1.72***	-1.32	***-1.82***	***-2.70***
CBU_0492	plsX	glycerol-3-phosphate acyltransferase PlsX	***-1.90***	-1.38	***-1.71***	***-2.61***
CBU_0493	fabH	3-oxoacyl-ACP synthase	***-1.89***	***-1.74***	***-1.70***	***-2.13***
CBU_0494	fabD	malonyl CoA-ACP transacylase	-1.08	-1.37	***-1.76***	***-1.80***
CBU_0495	fabG	3-oxoacyl-ACP reductase	1.05	-1.25	***-1.40***	***-1.48***
CBU_0496	acpP	acyl carrier protein	1.04	-1.10	-1.08	-1.08
CBU_0497	fabF	3-oxoacyl-ACP synthase	-1.06	-1.07	-1.22	***-1.44***
CBU_0498	-	hypothetical protein	***1.36***	***1.48***	***2.16***	***2.55***
CBU_0499	tmk	thymidylate kinase	1.18	1.13	***1.27***	***1.23***
CBU_0500	holB	DNA polymerase III subunit delta'	-1.01	-1.02	***1.48***	1.38
CBU_0501	-	hypothetical protein	-1.41	***-1.68***	-1.33	-1.26
CBU_0502	-	TatD family hydrolase	-1.19	-1.17	-1.28	***-1.61***
CBU_0503	glnA	glutamine synthetase	-1.14	-1.00	-1.03	-1.18
CBU_0504	-	di-/tripeptide transporter	***-1.60***	-1.24	-1.34	***-1.85***
CBU_0505	-	acetyltransferase	1.27	1.42	***1.96***	***2.63***
CBU_0506	recJ	single-stranded-DNA-specific exonuclease	1.03	1.05	1.07	-1.10

NCBI RSA 493 mapping	NCBI syn	Description	5	7	14	21
CBU_0507	-	hypothetical protein	-1.35	-1.40	-1.86	-1.89
CBU_0508	-	hypothetical protein	1.52	1.73	***2.43***	***3.74***
CBU_0509	trpR	Trp operon repressor	-1.10	-1.14	-1.07	1.13
CBU_0510	-	hypothetical protein	-1.24	-1.06	1.02	1.06
CBU_0513	-	fructose-1,6-bisphosphatase	1.25	1.11	-1.18	***-1.56***
CBU_0514	-	hypothetical protein	-1.05	-1.23	-1.17	-1.15
CBU_0514a	-	hypothetical protein	1.31	***1.87***	***2.65***	***3.12***
CBU_0515	-	MFS superfamily transporter	1.26	1.21	***2.23***	***2.23***
CBU_0516	-	hypothetical protein	1.31	1.02	1.48	***2.45***
CBU_0517	aspB	aspartate aminotransferase	***-2.29***	***-1.61***	***-2.50***	***-3.60***
CBU_0518	uvrB	excinuclease ABC subunit B	***-1.75***	-1.34	***-1.62***	***-1.95***
CBU_0519	-	DedA family protein	***-1.97***	-1.46	***-1.70***	***-2.15***
CBU_0520	leuA	hydroxymethylglutaryl-CoA lyase	***-1.52***	-1.36	***-1.98***	***-2.90***
CBU_0521	-	N-ethylammeline chlorohydrolase	***-1.75***	-1.16	***-1.76***	***-3.06***
CBU_0522	-	hypothetical protein	-1.99	-2.87	-1.24	-1.71
CBU_0524	gyrA	DNA gyrase subunit A	1.07	***1.43***	***1.47***	***1.37***
CBU_0525	serC	phosphoserine aminotransferase	-1.09	-1.19	-1.20	***-1.37***
CBU_0526	aroA	3-phosphoshikimate 1-carboxyvinyltransferase	-1.08	-1.15	-1.21	-1.30
CBU_0527	cmk	cytidylate kinase	-1.21	***-1.53***	***-1.67***	***-1.85***
CBU_0528	rpsA	30S ribosomal protein S1	-1.23	-1.34	***-1.42***	***-1.44***
CBU_0529	-	hypothetical protein	-1.22	-1.21	-1.12	***-1.44***
CBU_0530	-	hypothetical protein	-1.18	-1.47	1.08	-1.18
CBU_0531	pyrF	orotidine 5'-phosphate decarboxylase	***-1.63***	***-1.65***	***-1.71***	***-1.81***
CBU_0532	-	competence protein ComEA, helix-hairpin-helix repeat region	***-1.55***	***-1.53***	***-1.56***	***-2.41***
CBU_0533	rfe	undecaprenyl-phosphate alpha-N-acetylglucosaminyltransferase	-1.20	-1.11	-1.06	-1.22
CBU_0534	-	hypothetical protein	-1.28	-1.02	1.04	-1.24
CBU_0535	-	sporulation and cell division repeat protein	-1.05	1.08	1.04	-1.32
CBU_0536	-	hypothetical protein	2.15	***3.70***	***9.75***	***16.22***
CBU_0537	-	hypothetical protein	1.74	1.38	-1.01	2.21
CBU_0538	-	hypothetical protein	***2.48***	***3.68***	***6.22***	***8.78***
CBU_0539	-	tripeptide permease TppB	1.26	1.22	1.32	1.32
CBU_0540	smc	chromosome segregation protein SMC	-1.15	-1.33	-1.19	-1.37
CBU_0541	zipA	cell division protein ZipA	1.07	-1.04	-1.19	-1.18
CBU_0542	ligA	NAD-dependent DNA ligase	-1.21	-1.43	***-1.46***	***-1.82***
CBU_0543	-	hypothetical protein	-2.15	-1.47	-1.27	-1.98
CBU_0544	-	hypothetical protein	-1.26	-1.23	-1.15	-1.28
CBU_0545	lemA	LemA family protein	***1.47***	***1.72***	***2.31***	***2.78***
CBU_0546	htpX	heat shock protein HtpX	1.51	1.81	***2.49***	***3.07***
CBU_0547	-	hypothetical protein	1.21	1.03	1.12	1.28
CBU_0548	-	lytic murein transglycosylase B	-1.06	-1.12	-1.27	-1.30
CBU_0549	rodA	rod shape-determining protein	-1.30	-1.12	1.22	-1.06
CBU_0550	pbpA	penicillin-binding protein	1.48	1.14	1.73	1.92
CBU_0552	-	iojap family protein	-1.30	-1.18	***-1.59***	***-1.96***
CBU_0555	-	hypothetical protein	***3.25***	***3.50***	***4.58***	***5.18***
CBU_0556	nadD	nicotinate (nicotinamide) nucleotide adenylyltransferase	-1.06	-1.58	-1.71	***-1.93***
CBU_0557	holA	DNA polymerase III subunit delta	-1.43	-1.15	-1.46	-1.64
CBU_0558	-	rare lipoprotein B	-1.29	-1.56	-1.60	***-1.89***
CBU_0559	leuS	leucyl-tRNA synthetase	-1.51	***-1.68***	***-2.21***	***-2.64***
CBU_0560	-	hypothetical protein	-1.20	1.09	1.09	-1.21
CBU_0562	-	ATP-binding protein	1.03	-1.16	1.05	1.02
CBU_0562a	-	hypothetical protein	***1.75***	***1.51***	***1.63***	***2.00***
CBU_0563	-	hypothetical protein	-1.01	1.02	1.27	-1.04
CBU_0564	cutE	apolipoprotein N-acyltransferase	1.23	-1.24	-1.28	-1.37
CBU_0565	corC	hypothetical protein	1.23	-1.03	-1.16	***-1.49***
CBU_0566	-	MFS superfamily transporter	-1.21	-1.67	-1.33	-1.16
CBU_0567	-	hypothetical protein	-1.12	-1.57	-1.62	-1.63
CBU_0568	-	PhoH-like protein	-1.03	-1.14	-1.12	-1.20
CBU_0569	-	(dimethylallyl)adenosine tRNA methylthiotransferase	-1.39	-1.26	-1.57	-1.47
CBU_0570	-	amino acid permease	***-1.71***	-1.23	***-1.46***	***-2.11***
CBU_0571	-	hypothetical protein	1.52	***2.54***	***3.90***	***4.91***
CBU_0572	-	cytosol aminopeptidase	1.14	1.03	1.13	1.10
CBU_0573	fadE	acyl-CoA dehydrogenase	1.04	-1.20	-1.21	-1.42
CBU_0574	-	acetyl-CoA acetyltransferase	1.06	-1.03	-1.18	-1.45
CBU_0576	yfcX	fatty oxidation complex subunit alpha	-1.22	-1.07	1.18	-1.09
CBU_0577	-	hypothetical protein	-1.10	-1.17	-1.05	-1.27
CBU_0578	-	polyprenyl-phosphate beta-D-glucosyltransferase	1.02	-1.14	1.19	1.13
CBU_0579	-	undecaprenyl-phosphomannose:protein mannosyltransferase	-1.05	-1.07	1.49	1.34
CBU_0580	-	hypothetical protein	1.07	-1.16	-1.13	-1.14
CBU_0581	-	ferredoxin	-1.22	-1.22	-1.29	***-1.51***
CBU_0582	bolA	BolA family protein	1.05	1.16	1.19	1.21

NCBI RSA 493 mapping	NCBI syn	Description	5	7	14	21
CBU_0583	-	glutaredoxin family protein	1.14	1.38	***1.45***	***1.49***
CBU_0584	-	hypothetical protein	1.22	1.24	1.22	***1.41***
CBU_0585	-	hypothetical protein	1.07	-1.05	1.10	1.18
CBU_0586	-	pyridine nucleotide-disulfide oxidoreductase	1.53	1.28	1.31	1.28
CBU_0587	glpE	thiosulfate sulfurtransferase	1.10	-1.07	-1.10	-1.11
CBU_0588	nadA	quinolinate synthetase	1.03	-1.14	1.12	1.15
CBU_0589	-	(2Fe-2S) ferredoxin	1.01	-1.17	-1.16	-1.12
CBU_0590	-	hypothetical protein	1.11	-1.04	-1.08	-1.01
CBU_0591	-	hypothetical protein	1.08	-1.35	-1.07	-1.18
CBU_0592	-	hypothetical protein	-1.12	-1.21	1.03	1.04
CBU_0596	-	metallo-beta-lactamase	1.04	-1.08	-1.24	***-1.42***
CBU_0596a	-	hypothetical protein	1.12	-1.32	-1.03	1.27
CBU_0597	-	oxygen-independent coproporphyrinogen III oxidase	-1.14	-1.20	-1.32	***-1.66***
CBU_0598	nudE	ADP-ribose diphosphatase NudE	1.28	1.13	1.16	1.21
CBU_0599	cysQ-1	3'(2'),5'-bisphosphate nucleotidase	***1.54***	1.02	1.12	1.32
CBU_0607	mvaD	diphosphomevalonate decarboxylase/isopentenyl-diphosphate delta-isomerase	-1.42	-1.33	-2.03	***-2.09***
CBU_0608	-	mevalonate kinase	-1.33	-1.28	-1.35	***-1.83***
CBU_0609	-	phopshomevalonate kinase	-1.33	-1.26	-1.50	***-2.22***
CBU_0610	-	hydroxymethylglutaryl-CoA reductase (NADPH)	-1.59	-1.12	***-2.01***	***-4.26***
CBU_0611	yaeT	hypothetical protein	-1.36	-1.24	-1.58	***-2.23***
CBU_0612	ompH	outer membrane chaperone protein Skp	1.02	-1.03	-1.13	-1.16
CBU_0613	lpxD	UDP-3-O-[3-hydroxymyristoyl] glucosamine N-acyltransferase	-1.04	-1.01	-1.04	-1.16
CBU_0614	fabZ	(3R)-hydroxymyristoyl-ACP dehydratase	1.07	-1.12	-1.16	-1.24
CBU_0615	lpxA	UDP-N-acetylglucosamine acyltransferase	1.02	-1.18	-1.32	***-1.53***
CBU_0616	-	hydrolase	-1.27	-1.65	-1.78	***-2.25***
CBU_0617	-	hypothetical protein	-1.10	-1.41	-2.87	***-5.87***
CBU_0618	-	acyltransferase	1.11	-1.04	-1.25	-1.19
CBU_0619	-	hypothetical protein	-1.70	-1.41	-1.41	***-1.70***
CBU_0620	lpxB	lipid-A-disaccharide synthase	-1.08	-1.24	-1.18	-1.31
CBU_0621	-	oxidoreductase, NAD-binding	1.04	-1.04	1.10	1.02
CBU_0622	-	zinc-binding domain-containing protein	-1.09	-1.47	***-1.66***	***-1.65***
CBU_0623	-	hypothetical protein	1.04	-1.17	-1.29	-1.18
CBU_0624	-	hypothetical protein	-1.11	-1.29	-1.22	-1.22
CBU_0625	-	hypothetical protein	***-1.57***	***-1.41***	***-1.34***	***-1.59***
CBU_0626	-	hypothetical protein	-1.01	1.11	1.34	1.29
CBU_0627	-	hypothetical protein	-1.31	1.59	1.01	1.74
CBU_0628	ppa	inorganic diphosphatase	1.15	1.27	1.23	1.09
CBU_0629	putA	bifunctional proline dehydrogenase/pyrroline-5-carboxylate dehydrogenase	1.18	1.29	1.01	-1.18
CBU_0630	mip	peptidyl-prolyl cis-trans isomerase Mip	1.17	1.18	1.25	1.22
CBU_0631	purL	phosphoribosylformylglycinamidine synthase	1.17	1.10	-1.02	-1.10
CBU_0632	-	hypothetical protein	1.13	-1.06	-1.03	1.09
CBU_0634	-	aerobic respiration control sensor protein arcB	-1.24	-1.48	-1.49	***-1.80***
CBU_0635	-	hypothetical protein	1.07	-1.14	-1.11	-1.26
CBU_0636	-	transporter, UAA family	-1.00	-1.17	-1.09	-1.38
CBU_0637	-	coenzyme PQQ synthesis protein C	-1.07	-1.20	-1.22	-1.43
CBU_0638	-	branched-chain alpha-keto acid dehydrogenase subunit E2	1.10	-1.15	-1.59	***-1.83***
CBU_0639	-	pyruvate dehydrogenase (acetyl-transferring) E1 component subunit beta	-1.04	-1.31	***-1.67***	***-1.96***
CBU_0640	-	pyruvate dehydrogenase (acetyl-transferring) E1 component subunit alpha	-1.59	-1.50	***-1.92***	***-3.11***
CBU_0641	-	leucine dehydrogenase	***-1.88***	-1.46	***-2.23***	***-4.52***
CBU_0642	-	glycerophosphoryl diester phosphodiesterase	-1.04	-1.11	-1.22	-1.35
CBU_0643	ribD	5-amino-6-(5-phosphoribosylamino)uracil reductase	-1.03	1.03	-1.09	-1.31
CBU_0644	-	plasmid stabilization system toxin protein	1.26	1.16	1.58	***2.51***
CBU_0645	-	prevent-host-death family protein	1.22	1.34	***1.53***	***2.40***
CBU_0646	ribE	riboflavin synthase subunit alpha	***-1.61***	1.02	***-1.48***	***-1.51***
CBU_0647	ribA	3,4-dihydroxy-2-butanone 4-phosphate synthase	***-1.55***	1.09	-1.14	-1.18
CBU_0648	ribH	6,7-dimethyl-8-ribityllumazine synthase	-1.23	1.04	-1.29	***-1.66***
CBU_0649	-	riboflavin transporter	***-1.54***	-1.09	-1.27	***-1.47***
CBU_0656	-	hypothetical protein	1.08	1.24	1.17	1.24
CBU_0657	recR	recombination protein RecR	1.06	1.10	1.34	***1.44***
CBU_0658	-	hypothetical protein	1.00	1.08	1.17	1.23
CBU_0659	dnaZX	DNA polymerase III subunits gamma and tau	***-1.45***	-1.38	***-1.42***	***-1.66***
CBU_0660	-	hypothetical protein	-1.16	-1.26	-1.03	1.12
CBU_0661	-	hypothetical protein	-1.09	-1.36	-1.50	-1.34
CBU_0662	-	hypothetical protein	1.42	1.01	-1.19	-1.19
CBU_0663	-	hypothetical protein	1.00	1.02	-1.14	1.11
CBU_0664	-	ISAs1 family transposase	***-1.45***	-1.23	***-1.46***	***-1.62***
CBU_0665	-	hypothetical protein	-1.31	-1.08	1.17	1.15
CBU_0666	dapE	succinyl-diaminopimelate desuccinylase	***1.44***	1.33	***1.45***	***1.38***
CBU_0667	dapD	2,3,4,5-tetrahydropyridine-2,6-carboxylate N-succinyltransferase	***1.70***	1.35	***1.65***	***1.65***
CBU_0668	-	hypothetical protein	***1.40***	1.07	1.09	***1.35***

**NCBI RSA 493 mapping**	**NCBI syn**	**Description**	**5**	**7**	**14**	**21**
CBU_0669	-	hypothetical protein	-1.40	-1.14	-1.38	-1.33
CBU_0670	rhlE	ATP-dependent RNA helicase rhlE	1.58	-1.64	-1.91	-1.53
CBU_0671	rfbA	mannose-1-phosphate guanylyltransferase	-1.63	-1.57	***-1.89***	***-2.78***
CBU_0672	-	hypothetical protein	1.05	-1.20	-1.06	-1.24
CBU_0673	-	histidinol-phosphate phosphatase	-1.51	***-1.74***	***-2.07***	***-2.28***
CBU_0674	-	SIS domain-containing protein	-1.15	-1.31	-1.31	***-1.70***
CBU_0675	-	translaldolase	***-1.95***	***-1.54***	***-1.99***	***-3.53***
CBU_0676	-	UDP-glucose 4-epimerase	-1.76	-1.45	***-2.00***	***-2.24***
CBU_0677	-	NAD dependent epimerase/dehydratase	-1.32	-1.35	-1.59	***-2.10***
CBU_0678	-	ADP-heptose synthase	-1.24	-1.26	-1.79	***-1.97***
CBU_0679	-	Gfo/Idh/MocA family oxidoreductase	1.16	1.01	-1.84	-1.05
CBU_0680	-	UDP-glucose 6-dehydrogenase	-1.01	-1.58	-1.13	-1.16
CBU_0681	-	NAD-dependent epimerase/dehydratase	1.39	1.13	2.64	2.97
CBU_0682	-	methyltransferase	1.03	-1.16	-1.07	-1.48
CBU_0683	-	FkbM family methyltransferase	1.75	1.13	1.97	2.16
CBU_0684	-	sulfotransferase	1.48	1.21	1.67	1.36
CBU_0685	-	hypothetical protein	1.28	1.36	1.04	-1.20
CBU_0686	-	pyruvate dehydrogenase (acetyl-transferring) E1 component, alpha/beta fusion protein	-1.10	1.28	-1.01	-1.27
CBU_0687	-	hypothetical protein	1.83	1.16	1.32	1.20
CBU_0688	wcaG	GDP-L-fucose synthase	1.53	1.23	1.25	1.52
CBU_0689	gmd	GDP-mannose 4,6-dehydratase	1.41	1.43	1.14	-1.25
CBU_0690	-	polyprenyl-phosphate beta-D-mannosyltransferase	1.37	-1.01	1.25	2.30
CBU_0691	-	methyltransferase	1.59	-1.30	1.75	-1.41
CBU_0692	-	pyruvate dehydrogenase (acetyl-transferring) E1 component subunit beta	1.66	1.69	1.97	3.37
CBU_0693	-	pyruvate dehydrogenase (acetyl-transferring) E1 component subunit alpha	2.12	2.85	3.39	3.18
CBU_0694	-	glycosyltransferase	-2.67	1.28	2.33	2.40
CBU_0695	-	hypothetical protein	-1.49	-1.56	-2.14	-2.96
CBU_0696	-	DegT/DnrJ/EryC1/StrS aminotransferase	2.57	3.15	1.34	3.95
CBU_0697	-	DegT/DnrJ/EryC1/StrS aminotransferase	1.59	-1.01	1.83	1.84
CBU_0698	-	hypothetical protein	1.93	1.18	1.10	-1.17
CBU_0699	-	sulfotransferase	-1.36	***-1.52***	***-1.39***	***-1.59***
CBU_0700	-	bifunctional sulfate adenylyltransferase subunit 1/adenylylsulfate kinase	-1.23	-1.29	-1.36	***-1.74***
CBU_0701	cysQ-2	3'(2'),5'-bisphosphate nucleotidase	-1.15	-1.32	-1.64	***-2.03***
CBU_0702	-	short chain dehydrogenase/reductase oxidoreductase	-1.07	-1.26	-1.43	***-1.87***
CBU_0703	-	lipopolysaccharide/O-antigen ABC transporter permease	-1.39	-1.40	***-1.71***	***-2.55***
CBU_0704	rfbI	polysaccharide export ATP-binding protein	-1.37	-1.38	-1.38	-1.77
CBU_0705	-	hypothetical protein	1.17	1.03	1.26	***1.66***
CBU_0706	-	hypothetical protein	1.44	-1.02	1.30	***1.47***
CBU_0707	-	permease	1.73	1.49	1.75	***2.61***
CBU_0711	-	hypothetical protein	***4.03***	***4.00***	***5.74***	***9.46***
CBU_0712	gacA.1	GacA family DNA-binding response regulator	1.24	1.12	1.02	1.11
CBU_0713	-	phosphate transporter	-1.18	-1.24	-1.17	-1.32
CBU_0714	-	hypothetical protein	-1.02	-1.10	***-1.33***	***-1.51***
CBU_0715	-	IS30 family transposase	-1.11	1.10	-1.14	-1.21
CBU_0716	-	thioredoxin reductase	1.40	1.48	1.29	1.14
CBU_0718	-	hypothetical protein	***2.44***	2.11	***2.14***	***2.70***
CBU_0719	-	hypothetical protein	***2.30***	2.01	2.05	***2.58***
CBU_0720	-	agmatinase	***-1.49***	***-1.43***	***-2.12***	***-2.33***
CBU_0721	-	deoxyhypusine synthase-like protein	***-1.49***	-1.11	***-1.60***	***-2.19***
CBU_0722	-	decarboxylase, pyridoxal-dependent	***-2.30***	***-1.44***	***-2.27***	***-4.58***
CBU_0724c	-	hypothetical protein	***-2.87***	-1.94	***-2.72***	***-3.66***
CBU_0727	-	ABC transporter permease	1.15	1.03	1.10	1.01
CBU_0728	-	ABC transporter ATP-binding protein	1.01	-1.04	1.06	-1.04
CBU_0729	-	ABC transporter substrate-binding protein	1.60	1.40	1.54	1.56
CBU_0730	-	hypothetical protein	1.13	-1.15	-1.06	-1.08
CBU_0731	-	hypothetical protein	***4.04***	***3.71***	***4.50***	***5.17***
CBU_0734	-	hypothetical protein	1.07	-1.78	***-2.15***	-1.86
CBU_0735	-	uridine phosphorylase	-1.24	-1.18	-1.21	***-1.75***
CBU_0736	-	hypothetical protein	1.06	***1.56***	***1.54***	***1.47***
CBU_0737	tig	trigger factor	***-1.40***	-1.26	-1.33	***-1.35***
CBU_0738	clpP	ATP-dependent Clp protease proteolytic subunit	1.27	1.22	***1.36***	***1.46***
CBU_0739	clpX	ATP-dependent protease ATP-binding subunit ClpX	1.14	1.17	***1.59***	***1.57***
CBU_0740	lon	ATP-dependent endopeptidase	1.03	1.06	***1.30***	***1.43***
CBU_0741	pmbA	TldD/PmbA family protein	-1.02	-1.05	1.22	1.35
CBU_0742	-	hypothetical protein	1.13	1.29	1.60	***2.31***
CBU_0743	ptsH	phosphocarrier protein HPr	***2.29***	1.16	***1.71***	***2.65***
CBU_0744	-	HPr kinase/phosphorylase	1.19	-1.15	1.19	***1.62***
CBU_0745	-	ribosome-associated factor Y	1.13	1.36	***1.96***	***2.59***
CBU_0746	-	LPS ABC transporter ATP-binding protein	1.35	1.16	1.39	***1.64***
CBU_0747	-	cell envelope biogenesis protein YhbN	-1.41	-1.54	-2.04	***-2.04***

NCBI RSA 493 mapping	NCBI syn	Description	5	7	14	21
CBU_0748	-	hypothetical protein	1.05	-1.13	-1.18	-1.06
CBU_0749	-	HAD superfamily hydrolase	-1.26	-1.36	-1.54	***-2.12***
CBU_0750	-	arabinose-5-phosphate isomerase	***-1.35***	-1.23	***-1.38***	***-1.91***
CBU_0751	murA	UDP-N-acetylglucosamine 1-carboxyvinyltransferase	***1.84***	***1.70***	***2.32***	***2.70***
CBU_0752	-	esterase	1.46	1.11	1.44	1.16
CBU_0752a	-	hypothetical protein	1.22	1.02	1.28	***1.57***
CBU_0753	-	AcrB/AcrD/AcrF family transporter	1.22	-1.66	-1.22	-1.05
CBU_0754	-	RND family efflux transporter MFP subunit	-1.09	-1.29	1.27	***1.43***
CBU_0755	degP.2	endopeptidase	***1.45***	1.22	***1.34***	***1.43***
CBU_0756	-	hypothetical protein	1.03	-1.06	-1.11	-1.45
CBU_0757	rluD	ribosomal large subunit pseudouridine synthase D	-1.28	-1.10	-1.07	-1.33
CBU_0758	-	competence lipoprotein ComL	***-1.52***	-1.40	***-1.41***	***-2.17***
CBU_0760	gacS	response regulator receiver domain-containing protein	1.10	1.03	-1.03	1.05
CBU_0761	-	hypothetical protein	1.41	1.06	1.17	1.42
CBU_0762	-	hypothetical protein	-1.06	-1.14	-1.10	-1.18
CBU_0763	-	hypothetical protein	1.03	-1.13	-1.11	-1.03
CBU_0766	-	acetoacetyl-CoA synthetase	1.17	1.05	1.16	1.13
CBU_0767	-	hypothetical protein	***-1.48***	***-1.62***	***-2.01***	***-2.56***
CBU_0768	-	multidrug resistance protein B	1.07	1.07	-1.12	-1.53
CBU_0769	-	GMP synthase	-1.06	1.03	-1.36	-1.07
CBU_0770	-	ATPase	-1.31	-1.28	-1.21	***-1.54***
CBU_0771	prpB	2-methylisocitrate lyase	-1.11	1.18	1.27	1.16
CBU_0772	prpC	2-methylcitrate synthase	-1.27	1.07	1.01	-1.04
CBU_0773	phnB	glyoxalase	1.01	1.15	1.06	1.07
CBU_0774	pspC	PspC domain-containing protein	***1.74***	***1.68***	***1.89***	***2.82***
CBU_0775	-	GntR family transcriptional regulator	***1.54***	1.34	***1.42***	***2.07***
CBU_0776	-	ABC transporter ATP-binding protein	1.03	1.03	1.56	***2.07***
CBU_0777	-	ABC transporter permease	1.40	1.43	***1.72***	***1.76***
CBU_0777c	-	hypothetical protein	1.11	-1.08	-1.37	***-1.51***
CBU_0780	gacA.2	LuxR family transcriptional regulator	-1.16	-1.26	***-1.44***	***-1.78***
CBU_0781	-	ankyrin repeat-containing protein	-1.16	-1.17	-1.32	***-1.83***
CBU_0782	-	hypothetical protein	-1.03	-1.13	***-1.58***	***-1.68***
CBU_0784	-	hypothetical protein	1.11	-1.07	2.32	***3.52***
CBU_0786	-	coA-transferase III protein	-1.12	-1.15	1.02	1.21
CBU_0787	-	AMP-binding protein	-1.31	-1.23	-1.09	-1.11
CBU_0788	-	polyketide synthase	-1.12	-1.35	-1.10	-1.03
CBU_0789	-	two component system histidine kinase	1.68	1.26	***1.93***	***2.58***
CBU_0792	-	multidrug resistance protein B	1.07	1.00	-1.07	-1.06
CBU_0793	-	hypothetical protein	-1.22	-1.22	***-1.61***	***-1.60***
CBU_0794	-	hypothetical protein	-1.53	-1.24	-1.32	-1.30
CBU_0795	folE	GTP cyclohydrolase I	-1.08	-1.25	-1.56	***-1.89***
CBU_0796	-	histidine triad domain protein	-1.18	-1.25	-1.41	***-2.01***
CBU_0797	-	EmrB/QacA family drug resistance transporter	1.14	-1.33	-1.02	-1.00
CBU_0798	-	multidrug resistance protein A	1.02	-1.06	1.10	-1.14
CBU_0799	-	hypothetical protein	***-1.69***	-1.50	***-2.83***	***-3.98***
CBU_0800	-	hypothetical protein	-1.24	-1.29	***-1.75***	***-2.26***
CBU_0801	rimI	ribosomal-protein-S18-alanine acetyltransferase	-1.39	-1.45	***-1.76***	***-2.24***
CBU_0802	-	hypothetical protein	1.05	1.32	1.30	1.41
CBU_0803	-	RND family efflux transporter MFP subunit	1.25	1.40	1.07	-1.12
CBU_0804	-	AcrB/AcrD/AcrF family transporter	-1.29	-1.47	-1.10	1.03
CBU_0805	-	hypothetical protein	1.90	1.27	1.55	1.67
CBU_0806	-	hypothetical protein	1.60	1.23	1.09	1.16
CBU_0807	-	beta-lactamase	-1.03	***-1.45***	-1.31	***-1.55***
CBU_0808	valS	valyl-tRNA synthetase	***-1.80***	-1.31	***-1.78***	***-3.00***
CBU_0809	-	permease	-1.01	-1.17	-1.39	-1.47
CBU_0810	-	permease	-1.23	-1.22	-1.22	***-1.60***
CBU_0811	prfC	peptide chain release factor 3	***-1.68***	***-1.78***	***-1.59***	***-1.55***
CBU_0812	-	Na+ driven multidrug efflux pump	-1.79	***-2.31***	***-2.71***	***-2.54***
CBU_0813	-	hypothetical protein	-1.49	***-1.57***	-1.42	***-1.72***
CBU_0817	aacA4	GNAT family acetyltransferase	-1.53	-1.00	***-1.82***	***-1.82***
CBU_0818	-	TetR family transcriptional regulator	-1.16	1.15	1.30	1.33
CBU_0819	-	glutathione S-transferase	1.14	1.44	***1.94***	***2.98***
CBU_0822	-	Fic family protein	1.29	1.16	1.17	-1.03
CBU_0823	sfcA	malate dehydrogenase	1.05	-1.15	-1.07	-1.24
CBU_0824	purB	adenylosuccinate lyase	-1.07	-1.09	-1.41	***-1.97***
CBU_0825	-	DegT/DnrJ/EryC1/StrS aminotransferase	***-2.78***	***-1.54***	***-2.37***	***-3.75***
CBU_0826	rfaH	transcriptional activator RfaH	-1.80	-1.57	***-2.48***	***-3.47***
CBU_0827	-	hypothetical protein	***-1.67***	***-1.58***	***-2.51***	***-2.78***
CBU_0828	-	hexapeptide repeat-containing oxidoreductase	-1.32	-1.36	***-2.16***	***-2.30***
CBU_0829	-	NAD dependent epimerase/dehydratase	-1.08	-1.07	***-1.49***	***-1.50***

NCBI RSA 493 mapping	NCBI syn	Description	5	7	14	21
CBU_0830	-	perosamine synthetase	1.02	-1.09	-1.33	***-1.40***
CBU_0831	asnB-1	asparagine synthetase	-1.36	-1.41	***-2.11***	***-1.81***
CBU_0832	-	acetyltransferase	-1.21	-1.36	***-1.58***	***-1.58***
CBU_0833	-	ABC transporter permease/ATP-binding protein	***-1.53***	***-1.76***	***-1.93***	***-2.09***
CBU_0834	-	methyltransferase	-1.15	-1.16	-1.10	1.06
CBU_0835	-	hypothetical protein	-1.05	-1.56	-1.24	-1.06
CBU_0836	-	radical SAM domain-containing protein	-1.02	-1.31	-1.15	-1.02
CBU_0837	-	hypothetical protein	1.04	-1.16	***-1.36***	***-1.58***
CBU_0838	-	lipopolysaccharide N-acetylglucosaminyltransferase	-1.09	-1.29	-1.47	-1.62
CBU_0839	-	glycosyltransferase	-1.00	-1.30	-1.49	***-1.62***
CBU_0840	asnB-2	asparagine synthase	-1.04	-1.27	-1.33	***-1.52***
CBU_0841	-	alpha-D-QuiNAc alpha-1,3-galactosyltransferase	1.08	-1.39	***-1.81***	***-2.02***
CBU_0842	wecB	UDP-N-acetylglucosamine 2-epimerase	-1.04	-1.13	-1.48	-1.54
CBU_0843	-	glycosyltransferase	-1.23	-1.41	-1.66	***-1.80***
CBU_0844	-	capsular polysaccharide biosynthesis protein	***-2.02***	-1.72	***-2.44***	***-2.89***
CBU_0845	-	UDP-glucose/GDP-mannose dehydrogenase	***-1.72***	-1.43	***-2.02***	***-3.47***
CBU_0846	ugd	UDP-glucose 6-dehydrogenase	-1.39	-1.38	***-1.72***	***-1.99***
CBU_0847	-	oxidoreductase, short chain dehydrogenase/reductase	-1.48	-1.47	***-1.72***	***-2.39***
CBU_0848	pgi	glucose-6 phosphate 1-epimerase	-1.31	-1.15	-1.47	-1.66
CBU_0849	galU	UTP-glucose-1-phosphate uridylyltransferase	-1.18	-1.30	-1.29	-1.41
CBU_0850	-	hypothetical protein	***1.66***	1.59	***1.88***	***1.53***
CBU_0851	rpsO	30S ribosomal protein S15	***-1.54***	-1.24	***-1.75***	***-2.31***
CBU_0852	pnp	polynucleotide phosphorylase	-1.11	-1.25	-1.37	-1.42
CBU_0853	mgsA	methylglyoxal synthase	1.10	-1.12	1.24	1.24
CBU_0854	-	hypothetical protein	-1.33	-1.99	-1.15	-1.05
CBU_0856	-	lipid ABC transporter permease/ATP-binding protein	-1.01	-1.16	-1.21	-1.43
CBU_0857	lpxK	tetraacyldisaccharide 4'-kinase	1.12	-1.36	-1.13	-1.16
CBU_0858	nadE	glutamine-dependent NAD(+) synthetase	-1.08	1.03	1.02	1.03
CBU_0859	-	hypothetical protein	***1.32***	1.06	1.17	***1.28***
CBU_0860	-	hypothetical protein	1.01	1.20	-1.11	1.61
CBU_0863	-	hypothetical protein	-1.17	-1.30	-1.65	***-1.98***
CBU_0864	rpsF	30S ribosomal protein S6	***-1.94***	***-1.38***	***-1.81***	***-2.62***
CBU_0865	rpsR	30S ribosomal protein S18	***-1.85***	***-1.37***	***-1.61***	***-2.07***
CBU_0866	-	hypothetical protein	***-1.61***	-1.14	***-1.36***	***-1.82***
CBU_0867	rplI	50S ribosomal protein L9	***-1.94***	***-1.79***	***-2.31***	***-2.73***
CBU_0868	dnaB	replicative DNA helicase	-1.07	-1.07	-1.17	***-1.61***
CBU_0869	alr	alanine racemase	-1.00	-1.21	-1.22	***-1.52***
CBU_0870	-	hypothetical protein	-1.22	-1.40	***-1.58***	***-1.80***
CBU_0872	udk	uridine kinase	-1.14	-1.31	-1.44	***-2.09***
CBU_0873	-	Smr domain-containing protein	***-1.95***	***-1.60***	***-2.14***	***-3.23***
CBU_0874	aroC	chorismate synthase	-1.11	-1.15	-1.25	***-1.44***
CBU_0875	asd	aspartate-semialdehyde dehydrogenase	1.02	-1.23	***-1.42***	***-1.54***
CBU_0876	-	thiazole biosynthesis adenylyltransferase ThiF	-1.12	-1.09	-1.25	***-1.53***
CBU_0877	-	hypothetical protein	***2.42***	***2.69***	***3.77***	***5.55***
CBU_0880	-	hypothetical protein	1.65	-1.25	-1.12	1.85
CBU_0881	-	hypothetical protein	-1.04	-1.06	1.02	-1.01
CBU_0883	-	hypothetical protein	1.13	1.02	1.11	1.12
CBU_0884	bipA	GTP-binding protein TypA	***-2.30***	-1.52	***-1.79***	***-3.07***
CBU_0885	-	hypothetical protein	***-1.50***	-1.41	***-1.82***	***-2.16***
CBU_0886	coaBC	phosphopantothenate--cysteine ligase	-1.16	-1.27	***-2.05***	***-2.04***
CBU_0888	-	DsbB family disulfide bond formation protein	-1.01	-1.25	-1.42	***-2.07***
CBU_0889	dsbA	disulfide bond formation protein D	1.01	-1.10	-1.24	***-1.64***
CBU_0890	-	Zn-dependent hydrolase, glyoxalase II family	-1.43	***-1.53***	***-1.68***	***-2.56***
CBU_0891	-	hypothetical protein	1.36	1.40	1.17	-1.05
CBU_0892	truA	tRNA pseudouridine synthase A	1.05	-1.15	-1.25	***-1.62***
CBU_0893	accD	acetyl-coenzyme A carboxylase carboxyl transferase subunit beta	1.21	***1.38***	***2.16***	***2.91***
CBU_0894	folC	dihydrofolate synthase	***-3.11***	***-2.47***	***-3.43***	***-5.84***
CBU_0895	dedD	sporulation and cell division repeat protein	-1.26	-1.17	-1.18	-1.20
CBU_0896	dedE	colicin V production protein	-1.42	-1.31	-1.33	***-1.52***
CBU_0897	purF	amidophosphoribosyltransferase	-1.43	-1.40	-1.41	-1.59
CBU_0898	-	thyroglobulin type-1 repeat-containing protein	-1.13	-1.15	1.04	-1.03
CBU_0904	-	tRNA-dihydrouridine synthase A	1.08	1.27	1.37	1.08
CBU_0905	rpmE	50S ribosomal protein L31	***-2.11***	-1.42	***-2.42***	***-3.17***
CBU_0906	-	MFS superfamily transporter	1.08	1.18	1.02	1.10
CBU_0907	yciL	BolA	***-1.55***	***-1.57***	***-1.78***	***-1.96***
CBU_0908	ispZ	intracellular septation protein A	***-1.44***	-1.39	***-1.61***	***-2.10***
CBU_0909	-	sua5/YciO/YrdC/YwlC family protein	1.45	1.41	***1.86***	***2.30***
CBU_0910	-	hypothetical protein	1.06	-1.13	-1.15	-1.19
CBU_0911	-	hypothetical protein	-1.35	-1.22	***-2.12***	***-1.79***
CBU_0912	prpD	2-methylcitrate dehydratase	***-1.83***	-1.31	***-1.90***	***-2.55***

NCBI RSA 493 mapping	NCBI syn	Description	5	7	14	21
CBU_0913	-	thioesterase	***-2.57***	-1.33	***-2.32***	***-3.95***
CBU_0914	-	hypothetical protein	1.56	1.48	***1.75***	***2.16***
CBU_0915	enhB.1	enhanced entry protein	1.43	1.23	1.37	***1.75***
CBU_0916	-	hypothetical protein	-1.01	1.03	1.15	***1.59***
CBU_0918	-	hypothetical protein	-1.34	-1.33	-1.28	-1.25
CBU_0920	-	fatty acid desaturase	***1.46***	***1.53***	***2.25***	***3.23***
CBU_0921	-	hypothetical protein	***2.75***	***2.69***	***3.62***	***4.86***
CBU_0922	-	MFS superfamily transporter	-1.13	-1.05	1.01	-1.29
CBU_0923	-	hypothetical protein	1.04	-1.17	-1.27	-1.26
CBU_0924	-	O-methyltransferase	***2.41***	***2.23***	***2.66***	***3.71***
CBU_0925	-	lytic murein transglycosylase	***5.21***	***6.03***	***7.79***	***10.14***
CBU_0926	mmsB	3-hydroxyisobutyrate dehydrogenase	-1.11	1.07	-1.06	-1.02
CBU_0927	mmsA	malonate-semialdehyde dehydrogenase (acetylating)	-1.01	-1.04	***-1.45***	***-1.49***
CBU_0928	pdxH	pyridoxamine 5'-phosphate oxidase	***-1.46***	1.08	1.19	1.04
CBU_0929	-	short chain dehydrogenase/reductase oxidoreductase	1.09	-1.10	-1.14	***-1.64***
CBU_0930	-	3-methyladenine DNA glycosylase	-1.11	-1.00	1.17	1.19
CBU_0931	glpD	glycerol-3-phosphate dehydrogenase	-1.04	1.05	1.05	-1.18
CBU_0932	gplK	glycerol kinase	1.10	1.69	***1.75***	***1.84***
CBU_0933	-	ABC transporter permease	1.00	-1.29	-1.08	1.08
CBU_0934	-	ABC transporter ATP-binding protein	-1.34	-1.27	-1.11	-1.26
CBU_0935	-	RNA binding protein	-1.34	-1.12	-1.24	***-1.58***
CBU_0936	-	OmpA-like transmembrane domain-containing protein	1.19	1.06	1.16	***1.24***
CBU_0937	-	hypothetical protein	-1.18	1.09	1.15	1.01
CBU_0939	-	AsmA family protein	***-1.86***	-1.32	***-1.49***	***-2.17***
CBU_0940	mutY	A/G-specific adenine glycosylase	-1.44	-1.44	-1.36	***-1.59***
CBU_0941	-	hypothetical protein	***-1.64***	-1.04	***-1.86***	***-2.63***
CBU_0942	-	hypothetical protein	-1.15	1.02	1.17	-1.06
CBU_0943	-	rhodanese-like domain-containing protein	***1.91***	***2.63***	***2.94***	***3.68***
CBU_0944	-	hypothetical protein	-1.51	-1.28	1.46	1.21
CBU_0945	-	hypothetical protein	***-1.36***	-1.10	1.13	***1.28***
CBU_0946	rhuM	death-on-curing family protein	***-1.73***	-1.48	***-1.66***	***-2.15***
CBU_0948	-	hypothetical protein	1.01	-1.03	1.10	-1.05
CBU_0949	-	hypothetical protein	-1.26	-1.18	1.29	1.18
CBU_0952	-	hypothetical protein	1.38	1.15	1.17	1.28
CBU_0953	-	amino acid permease	1.13	-1.10	1.15	1.37
CBU_0954	-	phosphohydrolase	1.15	-1.16	1.07	1.17
CBU_0955	gacA.3	LuxR family transcriptional regulator	1.08	1.05	1.17	***1.27***
CBU_0956	-	hypothetical protein	***2.88***	***4.62***	***6.45***	***8.75***
CBU_0957	-	hypothetical protein	***5.58***	***8.73***	***18.02***	***28.45***
CBU_0959	-	Bcr/CflA subfamily drug resistance transporter	1.02	1.25	***1.38***	***1.44***
CBU_0960	-	DNA-binding protein/cupin domain protein	1.49	***2.93***	***5.99***	***7.90***
CBU_0961	-	hypothetical protein	1.61	***2.10***	***3.57***	***5.22***
CBU_0962	-	short chain dehydrogenase/reductase oxidoreductase	1.02	1.07	1.04	-1.03
CBU_0963	bcp	antioxidant, AhpC/TSA family	-1.32	1.07	-1.32	-1.85
CBU_0964	-	hypothetical protein	-1.04	1.12	1.31	1.13
CBU_0964a	-	hypothetical protein	-1.23	-1.59	***-2.01***	***-2.96***
CBU_0965	cydA-2	cytochrome d ubiquinol oxidase subunit I	***-1.37***	-1.25	***-1.49***	***-1.91***
CBU_0966	cydB	cytochrome d ubiquinol oxidase subunit II	-1.15	-1.26	-1.36	-1.54
CBU_0967	ybgT	cyd operon protein YbgT	-1.22	-1.35	-1.41	-1.36
CBU_0968	-	phospholipase D	-1.26	-1.09	1.04	-1.01
CBU_0970	-	hypothetical protein	***-2.18***	-1.49	***-1.66***	***-2.26***
CBU_0971	pyrD	dihydroorotate dehydrogenase 2	1.12	-1.04	-1.06	-1.09
CBU_0972	-	hypothetical protein	1.08	1.11	1.33	1.23
CBU_0973	-	isovaleryl-CoA dehydrogenase	-1.18	-1.16	-1.21	-1.69
CBU_0974	-	acetyl-CoA acetyltransferase	-1.28	-1.47	-1.68	***-1.81***
CBU_0975	-	acyl-CoA biotin-dependent carboxyltransferase	-1.05	-1.16	-1.20	-1.42
CBU_0976	-	methylglutaconyl-CoA hydratase	-1.06	-1.18	-1.32	-1.37
CBU_0977	-	biotin carboxylase	-1.07	-1.23	-1.27	***-1.46***
CBU_0978	-	hypothetical protein	-1.13	-1.16	-1.10	***-1.27***
CBU_0979	-	lipoprotein	1.60	1.57	***1.88***	***2.48***
CBU_0980	-	lipoprotein	1.58	1.22	1.21	1.49
CBU_0981	-	hypothetical protein	-1.06	-1.18	-1.14	-1.26
CBU_0982	-	phospho-2-dehydro-3-deoxyheptonate aldolase	***1.60***	***1.71***	1.27	1.21
CBU_0984	-	prephenate dehydrogenase	-1.07	-1.15	***-1.32***	***-1.51***
CBU_0985	-	hypothetical protein	1.29	-1.11	-1.11	-1.01
CBU_0986	-	RNA methyltransferase	-1.37	-1.03	1.04	-1.31
CBU_0987	-	rare lipoprotein A	-1.29	1.06	-1.04	-1.72
CBU_0988	ung	uracil-DNA glycosylase	***-1.33***	-1.17	***-1.40***	***-1.88***
CBU_0993	def	peptide deformylase	***-2.82***	***-1.83***	***-1.94***	***-2.48***
CBU_0994	-	hypothetical protein	1.23	-1.23	1.29	2.51

NCBI RSA 493 mapping	NCBI syn	Description	5	7	14	21
CBU_0995	-	cytochrome c oxidase, membrane subunit	***-4.10***	***-2.40***	***-3.28***	***-7.40***
CBU_0996	ctaB	protoheme IX farnesyltransferase	***-2.95***	***-1.74***	***-2.86***	***-4.81***
CBU_0997	-	lipoprotein	-1.84	-1.83	***-2.95***	***-4.69***
CBU_0998	purA	adenylosuccinate synthetase	-1.12	-1.34	***-1.66***	***-1.92***
CBU_0999	hflX	GTP-binding protein HflX	-1.29	***-1.70***	***-1.85***	***-2.08***
CBU_1000	lolD	lipoprotein releasing system, ATP-binding protein	-1.02	-1.38	***-1.55***	***-1.76***
CBU_1001	lolC	lipoprotein releasing system transmembrane protein LolC/E family	-1.06	-1.34	-1.09	-1.24
CBU_1002	birA	bifunctional biotin operon repressor/biotin synthetase BirA	***-1.53***	-1.43	***-1.97***	***-2.47***
CBU_1003	bioD	dethiobiotin synthetase	2.03	1.20	1.55	1.70
CBU_1004	bioC.2	biotin biosynthesis protein BioC	1.45	1.26	1.36	1.24
CBU_1005	bioH	carboxylesterase BioH	1.05	-1.06	-1.24	-1.14
CBU_1006	bioF	8-amino-7-oxononanoate synthase	-1.31	1.04	-1.26	-1.00
CBU_1007	bioB	biotin synthase	-1.28	1.23	1.07	-1.02
CBU_1008	bioA	adenosylmethionine-8-amino-7-oxononanoate aminotransferase	-1.27	-1.40	-1.39	1.13
CBU_1010	-	hypothetical protein	1.18	-1.09	-1.26	-1.35
CBU_1011	-	lignostilbene-alpha,beta-dioxygenase	***2.25***	1.29	***1.61***	***2.10***
CBU_1014	-	hypothetical protein	-1.09	-1.36	-1.18	***-1.96***
CBU_1015	-	hypothetical protein	-1.29	-1.57	-1.24	1.05
CBU_1016	-	hypothetical protein	-1.24	1.02	-1.10	-1.49
CBU_1017	-	ATPase	-1.26	1.02	-1.08	***-1.43***
CBU_1018	-	hypothetical protein	***-2.52***	-1.63	***-1.85***	***-3.45***
CBU_1019	-	hypothetical protein	-2.18	-1.56	-2.12	***-3.28***
CBU_1020	-	hypothetical protein	1.09	1.27	1.22	1.95
CBU_1021	-	nucleotidyltransferase	-1.23	-1.32	***-1.57***	***-1.94***
CBU_1022	-	nucleotidyltransferase domain-containing protein	***-1.62***	***-1.68***	-1.33	***-1.55***
CBU_1023	-	quinone oxidoreductase	-1.24	-1.19	-1.26	-1.06
CBU_1023a	-	hypothetical protein	-1.01	-1.19	1.10	1.37
CBU_1027	-	ATPase	1.25	1.24	1.28	1.23
CBU_1028	-	hypothetical protein	1.27	1.22	1.52	***1.79***
CBU_1031	-	phage lysozyme	1.81	1.49	***1.97***	***2.52***
CBU_1034	pgsA	CDP-diacylglycerol--glycerol-3-phosphate 3-phosphatidyltransferase	-1.33	-1.41	-1.40	***-2.53***
CBU_1035	-	nicotinate phosphoribosyltransferase	1.13	1.05	1.27	1.06
CBU_1035d	cyoD	cytochrome O ubiquinol oxidase	1.02	-1.16	-1.29	-1.32
CBU_1038	cyoC	cytochrome c oxidase polypeptide III	-1.31	-1.38	-1.57	***-1.84***
CBU_1039	cyoB	cytochrome c oxidase polypeptide I	-1.44	-1.21	-1.45	***-2.03***
CBU_1040	cyoA	cytochrome c oxidase polypeptide II	***-2.09***	-1.42	***-1.52***	***-2.36***
CBU_1041	-	hypothetical protein	-1.17	-1.00	-1.11	-1.10
CBU_1042	-	hypothetical protein	1.18	1.08	1.17	1.05
CBU_1043	gacA.4	LuxR family transcriptional regulator	-1.27	-1.33	-1.22	-1.07
CBU_1050	csrA-2	carbon storage regulator	***2.06***	1.87	***2.10***	***2.73***
CBU_1051	-	aspartokinase	-1.12	-1.02	1.10	1.08
CBU_1052	alaS	alanyl-tRNA synthetase	-1.06	-1.04	1.11	1.15
CBU_1053	recX	regulatory protein RecX	1.11	-1.00	1.19	***1.34***
CBU_1054	recA	recombinase A	1.15	1.21	1.32	1.23
CBU_1055	-	competence/damage inducible protein CinA	1.32	1.14	1.26	***1.30***
CBU_1056	mutS	DNA mismatch repair protein MutS	-1.06	-1.10	-1.09	-1.13
CBU_1057	-	cell wall biosynthesis glycosyltransferase	1.15	1.11	1.24	1.12
CBU_1058	-	hypothetical protein	1.02	-1.11	-1.18	-1.30
CBU_1059	-	RNA pseudouridine synthase	***1.32***	-1.02	1.16	1.04
CBU_1060	scpB	segregation and condensation protein	***1.43***	1.22	***1.24***	***1.27***
CBU_1061	scpA	segregation and condensation protein	1.26	1.06	1.08	1.18
CBU_1061a	-	hypothetical protein	-1.13	-1.23	-1.22	-1.52
CBU_1063	-	hypothetical protein	1.01	1.22	1.40	***1.76***
CBU_1064	-	hypothetical protein	1.05	-1.18	***-1.51***	***-1.87***
CBU_1065	-	2'-5' RNA ligase	1.25	1.11	1.03	-1.01
CBU_1066	-	transporter, divalent anion:sodium symporter family	1.14	-1.48	***-1.95***	***-2.22***
CBU_1067	ttcA	C32 tRNA thiolase	***-2.58***	***-1.83***	***-2.22***	***-5.28***
CBU_1067a	-	adenylate cyclase	1.10	1.06	-1.11	-1.41
CBU_1071	-	DoxX family protein	1.37	1.10	1.02	1.02
CBU_1072	tgt	queuine tRNA-ribosyltransferase	***-2.42***	-1.54	***-1.50***	***-2.35***
CBU_1073	-	hypothetical protein	1.20	1.21	***1.67***	***1.85***
CBU_1074	-	hypoxanthine-guanine phosphoribosyltransferase	***1.55***	1.19	***1.46***	***1.54***
CBU_1075	-	transporter, small conductance mechanosensitive ion channel (MscS) family	1.34	1.07	1.17	1.23
CBU_1077	nagZ	beta-hexosaminidase	-1.04	***-1.50***	***-1.58***	***-1.96***
CBU_1078	-	hypothetical protein	-1.05	-1.08	-1.14	-1.15
CBU_1079	-	hypothetical protein	-1.03	-1.03	-1.09	-1.05
CBU_1080	-	ErfK/YbiS/YcfS/YnhG family protein	-1.25	-1.25	***-1.59***	***-1.75***
CBU_1081	-	hypothetical protein	-1.08	-1.37	***-1.57***	***-2.30***
CBU_1082	miaA	tRNA delta(2)-isopentenylpyrophosphate transferase	1.04	-1.23	-1.23	-1.61
CBU_1083	mutL	DNA mismatch repair protein	-1.08	-1.13	-1.04	-1.22

NCBI RSA 493 mapping	NCBI syn	Description	5	7	14	21
CBU_1084	-	hypothetical protein	-1.03	-1.14	-1.10	*-1.46*
CBU_1085	-	N-acetylmuramoyl-L-alanine amidase	-1.09	*-1.46*	*-1.30*	*-1.48*
CBU_1087	-	hypothetical protein	-1.20	*-1.36*	*-1.53*	*-1.60*
CBU_1088	-	carbohydrate kinase	1.00	-1.17	*-1.48*	*-1.90*
CBU_1089	-	hypothetical protein	1.83	*1.89*	*2.49*	*2.76*
CBU_1091	vacB	ribonuclease R	1.26	-1.08	-1.12	*-1.26*
CBU_1092	-	lipoprotein	*2.34*	*2.50*	*2.68*	*2.79*
CBU_1093	-	AcrB/AcrD/AcrF family transporter	1.16	-1.29	1.05	-1.00
CBU_1094	-	RND family efflux transporter MFP subunit	1.75	1.18	1.14	1.05
CBU_1095	-	hypothetical protein	1.93	1.90	*2.15*	*2.57*
CBU_1096	fumC	fumarate hydratase	*2.41*	*2.56*	*3.59*	*4.72*
CBU_1097	aldC	alpha-acetolactate decarboxylase	*1.41*	1.35	*1.39*	*1.69*
CBU_1098	-	hypothetical protein	-1.28	1.21	1.38	1.37
CBU_1099	lepB-1	signal peptidase I	-1.19	-1.31	-1.06	-1.39
CBU_1100	-	hypothetical protein	1.22	-1.02	*-1.32*	*-1.58*
CBU_1100a	-	hypothetical protein	1.41	1.49	2.40	2.13
CBU_1103	-	Slt family transglycosylase	-1.02	1.27	1.45	1.33
CBU_1111	-	membrane-bound lytic murein transglycosylase A	-1.07	-1.27	-1.05	-1.22
CBU_1112	-	GIY-YIG catalytic domain-containing protein	*-2.53*	-1.44	-1.35	*-2.06*
CBU_1115	-	hypothetical protein	-1.10	-1.35	*-1.52*	*-1.55*
CBU_1116	ald	alanine dehydrogenase	-1.41	-1.79	-1.64	-1.72
CBU_1117	etfA	electron transfer flavoprotein subunit alpha	*-2.09*	*-1.67*	*-1.89*	*-2.40*
CBU_1118	etfB	electron transfer flavoprotein subunit beta	*-2.11*	*-1.50*	*-1.89*	*-2.53*
CBU_1119	-	carboxymethylenebutenolidase	*-1.85*	-1.28	-1.34	*-2.09*
CBU_1120	-	electron-transferring-flavoprotein dehydrogenase	-1.28	-1.53	-1.07	-1.26
CBU_1121	-	hypothetical protein	-1.91	-1.49	-1.20	-1.19
CBU_1122	enhA.3	enhanced entry protein	*-4.81*	*-2.31*	*-2.54*	*-3.99*
CBU_1123	cbpA	curved DNA-binding protein	-1.19	-1.02	-1.00	-1.35
CBU_1124	-	chaperone-modulator protein CbpM	-1.51	-1.48	*-2.04*	*-3.26*
CBU_1127	-	hypothetical protein	-1.11	-1.21	*-1.55*	*-2.13*
CBU_1128	iscU.1	NifU-like N terminal domain protein	-1.09	-1.12	-1.17	-1.34
CBU_1129	nifS	cysteine desulfurase	-1.31	-1.30	-1.20	*-1.56*
CBU_1130	-	OPT family oligopeptide transporter	1.09	-1.20	1.01	1.08
CBU_1131	-	RNA methyltransferase	-1.21	-1.06	-1.05	-1.30
CBU_1132	-	hypothetical protein	1.37	1.03	-1.00	-1.03
CBU_1133	suhB	myo-inositol-1(or 4)-monophosphatase	*-3.97*	*-2.14*	*-3.26*	*-5.41*
CBU_1134	-	hypothetical protein	-1.10	-1.11	-1.17	*-1.60*
CBU_1135	-	hypothetical protein	*1.97*	*1.83*	*2.37*	*2.90*
CBU_1136	-	enhanced entry protein enhC, tetratricopeptide repeat family	*5.36*	*6.93*	*11.58*	*13.82*
CBU_1137	enhB.2	enhanced entry protein	*4.00*	*4.83*	*5.78*	*6.68*
CBU_1138	enhA.4	enhanced entry protein	*3.25*	*4.63*	*6.43*	*8.03*
CBU_1138a	-	hypothetical protein	1.08	1.03	1.03	-1.51
CBU_1139	-	hypothetical protein	*3.94*	*2.79*	*2.47*	*2.61*
CBU_1141	secF	preprotein translocase subunit SecF	-1.08	-1.39	-1.31	-1.40
CBU_1142	secD	protein translocase subunit	-1.28	-1.00	1.23	1.11
CBU_1143	yajC	protein translocase subunit	1.04	1.01	-1.08	-1.04
CBU_1144	-	hypothetical protein	1.14	1.19	*1.28*	1.14
CBU_1145	-	hypothetical protein	1.31	1.05	-1.06	1.29
CBU_1147	mnmA	tRNA-specific 2-thiouridylase MnmA	*-1.91*	-1.36	*-1.37*	*-1.56*
CBU_1148	mfd	transcription-repair coupling factor	1.05	-1.33	*-1.48*	-1.27
CBU_1151	-	HAD superfamily hydrolase	-1.23	-1.03	-1.19	*-1.54*
CBU_1152	trpE	anthranilate synthase component I	1.09	-1.38	1.45	-1.11
CBU_1154	trpC	indole-3-glycerol phosphate synthase	-1.07	1.37	*2.34*	*2.55*
CBU_1155	trpBF	bifunctional phosphoribosylanthranilate isomerase/tryptophan synthase subunit beta	1.35	1.34	*2.31*	*3.00*
CBU_1156	trpA	tryptophan synthase subunit alpha	-1.01	1.16	*1.92*	*2.69*
CBU_1157	-	lipoprotein	*-2.05*	-1.44	-1.24	-1.34
CBU_1158	-	sterol delta-7-reductase	1.25	*2.13*	*2.81*	*2.85*
CBU_1159	-	hypothetical protein	1.29	1.25	*1.89*	*2.16*
CBU_1160	-	hypothetical protein	*1.38*	1.27	*1.48*	*1.62*
CBU_1161	-	hypothetical protein	1.26	-1.36	-1.20	1.00
CBU_1162	-	MFS superfamily transporter	-1.47	-1.27	-1.52	*-1.89*
CBU_1169	-	heat shock protein 20	1.37	*2.37*	*3.18*	*4.07*
CBU_1170	-	hypothetical protein	1.80	1.50	1.38	1.64
CBU_1171	-	hypothetical protein	*1.43*	1.09	1.08	*1.52*
CBU_1173	-	hypothetical protein	*2.25*	*2.15*	*2.41*	*3.25*
CBU_1175	-	hypothetical protein	1.41	1.38	*1.98*	*2.25*
CBU_1176	phrB	deoxyribodipyrimidine photolyase	*-1.47*	*-1.48*	-1.10	-1.29
CBU_1178	-	hypothetical protein	-1.32	*-1.50*	-1.30	*-1.74*
CBU_1179	-	Bcr/CflA subfamily drug resistance transporter	*-1.87*	-1.36	*-1.54*	*-2.37*
CBU_1180	-	hypothetical protein	3.99	2.76	1.01	3.03

NCBI RSA 493 mapping	NCBI syn	Description	5	7	14	21
CBU_1181	thiI	thiamine biosynthesis protein ThiI	-1.23	***-1.63***	-1.40	-1.40
CBU_1182	iscS	class V aminotransferase	-1.08	-1.43	-1.40	***-1.74***
CBU_1183	-	nucleic acid binding domain-containing protein	1.31	-1.05	-1.08	1.21
CBU_1184	-	acyltransferase	***-1.97***	-1.47	***-1.57***	***-2.39***
CBU_1185	uvrC	excinuclease ABC subunit C	-1.18	-1.00	1.03	-1.12
CBU_1186b	-	hypothetical protein	-1.59	-1.84	-1.38	-1.73
CBU_1187	-	hypothetical protein	-1.29	-1.16	-1.28	-1.10
CBU_1188	serS	seryl-tRNA synthetase	-1.10	-1.36	-1.10	-1.07
CBU_1189	-	recombination factor protein RarA	-1.16	-1.23	***-1.45***	***-2.02***
CBU_1190	lolA	outer-membrane lipoproteins carrier protein	-1.10	1.11	-1.05	***-1.46***
CBU_1191	ftsK	DNA translocase FtsK	1.05	1.04	1.15	1.10
CBU_1193	trxB	thioredoxin-disulfide reductase	***-1.72***	-1.44	***-1.74***	***-2.59***
CBU_1194	-	hypothetical protein	***-2.10***	-1.60	***-2.05***	***-3.05***
CBU_1195	infA	translation initiation factor IF-1	1.02	-1.04	-1.23	***-1.53***
CBU_1196	clpA	ATP-dependent Clp protease ATP-binding subunit ClpA	***5.20***	***6.89***	***11.38***	***14.98***
CBU_1197	-	hypothetical protein	1.12	-1.25	-1.32	-1.25
CBU_1198	-	hypothetical protein	1.12	-1.14	***-1.32***	***-1.48***
CBU_1199	-	hypothetical protein	***2.15***	1.51	1.11	-1.13
CBU_1200	icd	isocitrate dehydrogenase	***-1.93***	-1.30	***-1.63***	***-2.75***
CBU_1201	queA	S-adenosylmethionine--tRNA ribosyltransferase-isomerase	***-2.37***	***-1.80***	***-1.86***	***-3.05***
CBU_1202	-	hypothetical protein	***2.40***	***1.84***	***2.16***	***2.92***
CBU_1203	-	glutamate synthase domain protein	2.07	1.44	1.67	1.59
CBU_1204	-	succinate-semialdehyde dehydrogenase	***1.79***	1.30	***1.60***	***1.55***
CBU_1206	-	delta(24(24(1)))-sterol reductase	-1.06	-1.43	-1.69	***-2.65***
CBU_1207	-	hypothetical protein	-1.11	-1.39	-1.93	-2.00
CBU_1208	-	major facilitator transporter	1.57	1.14	1.41	1.31
CBU_1209	-	hypothetical protein	1.30	1.06	-1.17	-1.14
CBU_1212a	-	hypothetical protein	1.62	1.23	-1.01	-1.27
CBU_1213	-	ankyrin repeat-containing protein	1.33	1.24	1.48	***1.68***
CBU_1214	-	hypothetical protein	***2.32***	***2.17***	***2.45***	***3.43***
CBU_1217	-	hypothetical protein	2.65	2.46	2.40	***3.21***
CBU_1219	-	hypothetical protein	-1.12	***-1.61***	***-1.65***	***-1.85***
CBU_1220	purC	phosphoribosylaminoimidazole-succinocarboxamide synthase	-1.12	-1.16	-1.23	-1.57
CBU_1221	-	lipoprotein	1.01	-1.02	-1.02	-1.20
CBU_1222	dapA	dihydrodipicolinate synthase	1.15	-1.06	-1.05	-1.21
CBU_1223	kdgK	2-dehydro-3-deoxygluconokinase	-1.13	1.12	1.17	-1.14
CBU_1224	-	RmuC family protein	1.01	1.11	1.22	1.01
CBU_1225	-	haloalkane dehalogenase	-1.18	-1.10	-1.03	-1.24
CBU_1226	-	glutamate dehydrogenase	-1.23	-1.09	-1.00	1.02
CBU_1227	qseB	transcriptional regulatory protein	1.26	1.14	1.31	***1.54***
CBU_1228	qseC	sensor protein qseC	***1.63***	1.17	1.22	1.35
CBU_1229	-	ATP-dependent nuclease subunit B	-1.48	-1.36	-1.59	-1.78
CBU_1230	-	ATP-dependent nuclease subunit A	-1.34	-1.37	-1.99	***-4.77***
CBU_1231	-	hypothetical protein	-1.13	1.04	1.20	1.38
CBU_1233	-	5'-nucleotidase	***1.92***	1.49	***2.15***	***2.89***
CBU_1234	-	ATPase	1.79	1.70	***2.45***	***3.06***
CBU_1235	orn	oligoribonuclease	1.43	1.20	1.04	1.01
CBU_1236	-	hypothetical protein	1.11	1.21	1.20	1.37
CBU_1237	-	twitching motility protein PilT	-1.17	-1.20	***-1.57***	***-2.39***
CBU_1238	-	CopG family transcriptional regulator	-1.30	-1.36	***-2.04***	***-2.68***
CBU_1239	-	hypothetical protein	***1.37***	1.28	1.18	1.09
CBU_1240	gcp	DNA-binding/iron metalloprotein/AP endonuclease	-1.41	-1.21	***-1.69***	***-2.22***
CBU_1241	mdh	malate dehydrogenase	-1.44	-1.11	-1.59	***-2.07***
CBU_1242	-	long-chain fatty acid transport protein	1.47	1.21	1.20	1.01
CBU_1243	xseA	exodeoxyribonuclease VII large subunit	-1.10	-1.04	-1.14	-1.44
CBU_1244	-	EmrB/QacA family drug resistance transporter	-1.31	-1.23	-1.68	***-2.64***
CBU_1245	engA	GTP-binding protein EngA	-1.02	-1.21	-1.35	-1.37
CBU_1247	-	hypothetical protein	-1.21	-1.29	-1.25	***-1.47***
CBU_1248	hisS	histidyl-tRNA synthetase	1.27	-1.14	-1.04	-1.15
CBU_1249	-	DNA-binding protein	1.30	1.04	1.21	-1.07
CBU_1250	-	hypothetical protein	1.74	1.57	1.77	1.23
CBU_1252	-	radical SAM protein	1.03	1.28	***1.84***	***2.18***
CBU_1255	-	polyprenyl synthetase	1.01	1.09	1.07	-1.07
CBU_1256	-	hypothetical protein	-1.24	-1.15	-1.33	***-1.72***
CBU_1257	-	hypothetical protein	1.11	1.02	-1.25	***-1.45***
CBU_1258	ndk	nucleoside diphosphate kinase	***-3.04***	***-2.23***	***-2.69***	***-4.35***
CBU_1259	nhaP.1	Na+/H+ antiporter	1.36	1.20	1.14	1.08
CBU_1260	-	OmpA-like transmembrane domain-containing protein	-1.36	1.17	1.17	***1.56***
CBU_1261	-	D-alanyl-D-alanine serine-type carboxypeptidase	***-1.42***	-1.12	***-1.55***	***-2.29***
CBU_1262	-	hypothetical protein	***-1.94***	***-1.81***	-1.60	***-2.26***

NCBI RSA 493 mapping	NCBI syn	Description	5	7	14	21
CBU_1263	-	hypothetical protein	-1.36	-1.26	*-1.57*	*-2.23*
CBU_1265	lipB	lipoate-protein ligase B	*-1.48*	-1.25	*-1.53*	*-2.35*
CBU_1266	lipA	lipoyl synthase	-1.03	-1.00	-1.08	-1.15
CBU_1267	-	DedA/PAP2 domain-containing protein	-1.11	-1.35	-1.21	-1.35
CBU_1268	-	pyridine nucleotide-disulfide oxidoreductase	*-1.33*	*-1.38*	*-1.44*	*-1.27*
CBU_1269	-	thioesterase	1.17	1.14	1.50	1.16
CBU_0006a	-	hypothetical protein	1.67	1.05	-1.34	-1.75
CBU_1272	-	hypothetical protein	*2.49*	2.11	*2.15*	*2.57*
CBU_1273	-	6-phosphofructokinase	*-2.65*	*-1.63*	*-2.03*	*-4.01*
CBU_1274	-	hypothetical protein	1.20	1.12	-1.25	*-1.43*
CBU_1275	RspA	mandelate racemase	-1.28	-1.02	-1.25	*-1.63*
CBU_1276	-	short chain dehydrogenase/reductase oxidoreductase	-1.26	-1.09	*-1.65*	*-2.14*
CBU_1277	eda	2-dehydro-3-deoxyphosphooctonate aldolase	-1.32	-1.25	-1.73	*-1.99*
CBU_1278	-	antioxidant, AhpC/TSA family	*-1.32*	*-1.38*	*-1.73*	*-2.32*
CBU_1279	exoD	exopolysaccharide transporter	-1.05	-1.15	-1.10	-1.09
CBU_1279a	-	hypothetical protein	-1.01	-1.14	-1.93	-1.86
CBU_1280	greA	transcription elongation factor	1.18	1.02	1.10	1.12
CBU_1280a	-	hypothetical protein	*2.03*	1.71	1.83	*2.54*
CBU_1281	carB	carbamoyl-phosphate synthase large subunit	*-1.93*	*-1.69*	*-1.98*	*-2.15*
CBU_1282	carA	carbamoyl phosphate synthase small subunit	*-2.20*	*-1.74*	*-2.48*	*-3.41*
CBU_1284	-	ATPase	1.21	-1.16	-1.03	1.17
CBU_1285	-	multidrug resistance protein B	1.11	-1.46	-1.18	-1.35
CBU_1286	-	ATPase	-1.00	-1.17	-1.34	*-1.83*
CBU_1289	dnaJ	molecular chaperone DnaJ	*-2.14*	*-1.54*	*-1.55*	*-1.77*
CBU_1290	dnaK	molecular chaperone DnaK	*-2.30*	1.08	-1.05	-1.04
CBU_1291	-	biotin-requiring enzyme subunit	-1.08	1.13	1.29	1.05
CBU_1292	-	ankyrin repeat-containing protein	-1.27	-1.39	-1.33	-1.34
CBU_1293	grpE	heat shock protein GrpE	*-1.88*	*-1.29*	*-1.31*	-1.13
CBU_1294	-	twitching motility protein PilT	1.35	*1.55*	*2.52*	*4.21*
CBU_1295	-	hypothetical protein	*1.94*	*2.49*	*4.11*	*5.96*
CBU_1296	ppnK	inorganic polyphosphate/ATP-NAD kinase	*1.44*	1.06	1.11	1.13
CBU_1297	recN	DNA repair protein	-1.18	-1.23	-1.21	*-1.53*
CBU_1300	-	hypothetical protein	1.16	1.66	*2.35*	*2.99*
CBU_1301	fur	ferric uptake regulation protein	1.20	1.26	1.30	1.25
CBU_1302	omlA	hypothetical protein	*-1.32*	-1.11	*-1.43*	*-1.74*
CBU_1303	-	hypothetical protein	-1.01	-1.08	-1.25	*-1.31*
CBU_1304	-	polyketide cyclase/dehydrase	-1.08	-1.23	*-1.37*	*-1.70*
CBU_1305	smpB	SsrA-binding protein	*-2.94*	*-2.14*	*-3.06*	*-4.90*
CBU_1306	msrA	bifunctional methionine sulfoxide reductase B/A protein	*-3.35*	*-2.18*	*-3.12*	*-6.12*
CBU_1307	-	hypothetical protein	-1.45	-1.86	1.09	1.26
CBU_1308	-	phosphohydrolase	*-2.56*	-1.29	*-1.99*	*-2.98*
CBU_1310	-	hypothetical protein	1.16	1.30	2.21	*2.80*
CBU_1311	-	hypothetical protein	1.28	-1.21	-1.07	1.70
CBU_1312	-	hypothetical protein	1.63	1.29	1.53	1.52
CBU_1313	-	hypothetical protein	-1.29	*-1.53*	*-1.63*	*-2.60*
CBU_1314	-	hypothetical protein	1.34	1.14	1.62	*2.86*
CBU_1316	-	hypothetical protein	1.20	-1.11	1.23	1.25
CBU_1316a	-	hypothetical protein	-1.00	-1.19	-1.17	1.06
CBU_1317	-	hypothetical protein	1.22	1.08	-1.28	-1.84
CBU_1318	-	hypothetical protein	*-2.21*	*-1.89*	*-2.54*	*-3.02*
CBU_1319	-	transcriptional regulator domain-containing protein	-1.00	-1.11	-1.17	*-1.23*
CBU_1320	ihfA	integration host factor subunit alpha	1.09	1.01	1.01	-1.00
CBU_1321	pheT	phenylalanyl-tRNA synthetase subunit beta	1.01	-1.20	-1.23	*-1.38*
CBU_1322	pheS	phenylalanyl-tRNA synthetase subunit alpha	-1.15	-1.16	*-1.46*	*-1.68*
CBU_1323	rplT	50S ribosomal protein L20	-1.20	-1.34	*-1.75*	*-1.98*
CBU_1323a	-	hypothetical protein	1.23	1.02	1.22	*1.86*
CBU_1324	rpmI	50S ribosomal protein L35	-1.29	-1.22	-1.42	-1.31
CBU_1325	infC	translation initiation factor IF-3	*-1.60*	-1.30	*-1.61*	*-1.75*
CBU_1326	thrS	threonyl-tRNA synthetase	-1.45	-1.25	-1.32	*-1.45*
CBU_1328	-	hypothetical protein	*-2.02*	*-1.92*	*-2.86*	*-3.18*
CBU_1329	-	radical SAM superfamily protein	-1.62	-1.02	-1.11	1.03
CBU_1331	-	hypothetical protein	*3.42*	*3.71*	*5.60*	*8.26*
CBU_1332	-	hypothetical protein	*2.69*	*2.90*	*3.89*	*5.97*
CBU_1333	-	hypothetical protein	-1.30	1.01	-1.03	1.35
CBU_1334	-	hypothetical protein	-1.23	-1.35	-1.24	*-1.65*
CBU_1335	-	cyclin	-1.16	-1.18	-1.16	*-1.45*
CBU_1336	-	transporter, drug/metabolite exporter family	1.24	1.08	1.32	1.34
CBU_1337	dnaE	DNA polymerase III subunit alpha	-1.24	-1.42	*-1.74*	*-2.17*
CBU_1338	ddl	D-alanyl-alanine synthetase A	-1.17	-1.01	-1.15	*-1.57*
CBU_1339	rnhB	ribonuclease HII	-1.58	-1.47	*-1.99*	*-2.28*

NCBI RSA 493 mapping	NCBI syn	Description	5	7	14	21
CBU_1340	-	hypothetical protein	-1.01	-1.26	***-1.48***	***-1.90***
CBU_1341	guaA	GMP synthase	-1.48	-1.28	***-1.92***	***-2.14***
CBU_1342	guaB	inosine-5'-monophosphate dehydrogenase	-1.29	-1.10	***-1.68***	***-2.19***
CBU_1345	-	MFS superfamily transporter	1.58	1.10	-1.14	-1.19
CBU_1346	-	trehalase	-1.29	-1.18	-1.15	***-1.86***
CBU_1347	-	amino acid permease	***1.52***	1.34	***1.99***	***2.23***
CBU_1348	-	hypothetical protein	-1.13	1.66	***2.28***	***3.96***
CBU_1349	-	hypothetical protein	1.04	-1.13	-1.34	***-1.69***
CBU_1350	glmM	phosphoglucosamine mutase	-1.06	-1.16	-1.26	***-1.64***
CBU_1351	folP	dihydropteroate synthase	-1.20	-1.33	-1.34	***-1.74***
CBU_1352	ftsH	ATP-dependent metallopeptidase HflB	1.04	1.02	1.10	-1.07
CBU_1353	rrmJ	23S rRNA Um2552 2'-O-methyltransferase	-1.15	-1.18	-1.37	-1.11
CBU_1354	-	hypothetical protein	-1.15	-1.18	-1.09	1.05
CBU_1355	-	FeS assembly scaffold SufA	***-1.57***	-1.41	***-1.39***	-1.28
CBU_1356	iscU.2	NifU family SUF system FeS assembly protein	-1.02	-1.12	1.02	1.02
CBU_1357	csdB	cysteine desulfurase	-1.21	-1.10	1.19	1.37
CBU_1358	sufD	FeS assembly protein SufD	-1.18	-1.26	1.16	***1.46***
CBU_1359	sufC	FeS assembly ATPase SufC	***-1.68***	-1.25	-1.02	1.10
CBU_1360	sufB	cysteine desulfurase	***-1.49***	1.07	***1.94***	***2.04***
CBU_1361	-	FeS assembly SUF system regulator	-1.45	-1.03	1.27	1.37
CBU_1362	czcD.2	cation diffusion facilitator family transporter	***-2.19***	-1.53	-1.42	-1.01
CBU_1363	-	exonuclease	-1.20	-1.30	-1.25	1.23
CBU_1366	-	hypothetical protein	-1.24	1.15	1.21	1.21
CBU_1369	-	hypothetical protein	1.08	-1.06	1.07	1.12
CBU_1370	-	hypothetical protein	1.09	1.27	1.99	1.97
CBU_1371	-	non-proteolytic protein, peptidase M22	-1.18	-1.03	-1.16	***-1.45***
CBU_1372	-	hypothetical protein	-1.18	-1.07	-1.00	-1.26
CBU_1373	pabB	para-aminobenzoate synthetase component I	***-1.65***	-1.30	-1.49	***-2.08***
CBU_1374	-	cytidine/deoxycytidylate deaminase	-1.38	-1.31	***-1.75***	***-2.25***
CBU_1375	relA	RelA/SpoT family protein	-1.17	-1.28	***-1.79***	***-1.79***
CBU_1376	-	membrane endopeptidase, M50 family	-1.38	1.37	1.14	1.06
CBU_1378	-	hypothetical protein	1.31	-2.90	-1.40	***-3.03***
CBU_1379a	-	hypothetical protein	-1.49	-1.48	***-1.73***	***-3.74***
CBU_1380	-	DoxD family protein	***1.29***	1.04	1.20	***1.26***
CBU_1381	cdsA	phosphatidate cytidylyltransferase	1.28	-1.17	-1.18	-1.29
CBU_1382	uppS	undecaprenyl pyrophosphate synthetase	-1.20	-1.11	-1.27	-1.17
CBU_1383	frr	ribosome recycling factor	***-1.40***	-1.14	-1.30	-1.34
CBU_1384	pyrH	uridylate kinase	***-1.59***	-1.19	***-1.55***	***-1.94***
CBU_1385	tsf	elongation factor Ts	-1.46	-1.05	***-1.57***	***-1.90***
CBU_1386	rpsB	30S ribosomal protein S2	***-2.08***	-1.19	***-1.73***	***-2.25***
CBU_1387	-	hypothetical protein	1.15	***1.47***	***1.72***	***2.24***
CBU_1388	map	methionine aminopeptidase	***-2.81***	***-1.90***	***-2.34***	***-3.91***
CBU_1388d	-	hypothetical protein	1.66	1.54	1.86	1.99
CBU_1394	enhA.5	enhanced entry protein	***7.50***	***7.72***	***7.85***	***9.63***
CBU_1395	-	hypothetical protein	-1.05	-1.30	-1.35	-1.16
CBU_1396	sucD	succinate-CoA ligase (ADP-forming) subunit alpha	-1.23	-1.30	-1.42	***-1.62***
CBU_1397	sucC	succinyl-CoA synthetase subunit beta	-1.23	***-1.59***	***-2.11***	***-2.65***
CBU_1398	sucB	dihydrolipoyllysine-residue succinyltransferase, E2 component of oxoglutarate dehydrogenase	-1.22	-1.42	***-1.60***	***-1.70***
CBU_1399	sucA	2-oxoglutarate dehydrogenase E1	***-1.58***	***-1.62***	***-2.08***	***-2.27***
CBU_1400	sdhB	succinate dehydrogenase iron-sulfur subunit	***-1.90***	***-1.67***	***-2.08***	***-2.59***
CBU_1401	sdhA	succinate dehydrogenase flavoprotein subunit	***-2.13***	***-1.81***	***-1.93***	***-2.46***
CBU_1402	sdhD	succinate dehydrogenase membrane anchor subunit	***-1.87***	***-1.42***	***-2.11***	***-3.24***
CBU_1403	sdhC	succinate dehydrogenase cytochrome b556 subunit	***-1.89***	***-1.36***	***-1.97***	***-2.77***
CBU_1404	-	hypothetical protein	***2.10***	***2.29***	***2.46***	***3.19***
CBU_1405	-	hypothetical protein	-1.12	-1.37	-1.22	-1.43
CBU_1408	-	hypothetical protein	-1.12	-1.19	-1.07	-1.07
CBU_1409	-	hypothetical protein	-1.26	1.15	1.16	-1.11
CBU_1410	gltA	citrate (Si)-synthase	1.03	***1.26***	***1.46***	***1.37***
CBU_1411	-	hypothetical protein	1.05	1.11	1.28	***1.30***
CBU_1412	-	hypothetical protein	-1.01	-1.20	***-2.17***	***-2.79***
CBU_1413	-	hypothetical protein	1.13	-1.01	-1.30	***-1.98***
CBU_1414	-	hypothetical protein	1.18	1.21	-1.14	***-2.34***
CBU_1415	thiL	thiamine-monophosphate kinase	-1.03	1.02	1.24	-1.10
CBU_1416	-	S24 family peptidase	-1.03	1.10	1.13	1.07
CBU_1417	nusB	transcription antitermination protein NusB	-1.37	-1.43	***-1.76***	***-2.05***
CBU_1418	nrdR	transcriptional regulator NrdR	-1.33	-1.07	-1.15	-1.30
CBU_1419	glyA	serine hydroxymethyltransferase	-1.20	-1.21	-1.40	***-1.85***
CBU_1422	radA	DNA repair protein RadA	***-1.81***	-1.48	***-1.77***	***-2.82***
CBU_1422a	-	hypothetical protein	-1.52	-1.78	-1.33	-1.51
CBU_1424	hemB	delta-aminolevulinic acid dehydratase	-1.02	-1.18	-1.08	-1.34

NCBI RSA 493 mapping	NCBI syn	Description	5	7	14	21
CBU_1425	-	hypothetical protein	1.24	1.15	1.18	1.19
CBU_1426	-	hypothetical protein	1.66	1.16	1.09	1.14
CBU_1427a	-	hypothetical protein	***3.82***	***3.03***	***4.40***	***4.76***
CBU_1429	-	hypothetical protein	1.34	1.25	1.30	1.19
CBU_1429a	-	hypothetical protein	***2.20***	***2.43***	***3.68***	***4.84***
CBU_1430	truB	tRNA pseudouridine synthase B	-1.14	-1.14	-1.16	-1.28
CBU_1431	rbfA	ribosome-binding factor A	-1.58	***-1.89***	***-2.30***	***-3.07***
CBU_1432	infB	translation initiation factor IF-2	-1.03	-1.18	***-1.50***	***-1.76***
CBU_1433	nusA	transcription elongation factor NusA	-1.31	-1.16	-1.34	***-1.67***
CBU_1434	-	hypothetical protein	-1.08	-1.06	***-1.47***	***-1.73***
CBU_1435	nuoN	NADH dehydrogenase subunit N	-1.29	-1.45	***-1.61***	***-2.65***
CBU_1436	nuoM	NADH dehydrogenase subunit M	***-1.74***	***-2.02***	***-1.97***	***-2.87***
CBU_1437	nuoL	NADH dehydrogenase subunit L	-1.58	-1.52	***-1.58***	***-1.93***
CBU_1438	nuoK	NADH dehydrogenase subunit K	-1.44	-1.44	-1.38	***-1.78***
CBU_1439	nuoJ	NADH dehydrogenase subunit J	-1.17	-1.59	-1.56	***-1.77***
CBU_1440	nuoI	NADH dehydrogenase subunit I	***-1.47***	***-1.47***	***-1.48***	***-1.93***
CBU_1441	nuoH	NADH dehydrogenase subunit H	-1.38	-1.59	***-1.82***	***-2.56***
CBU_1442	nuoG	NADH dehydrogenase subunit G	-1.04	-1.35	-1.39	-1.48
CBU_1443	nuoF	NADH dehydrogenase subunit F	***-1.64***	-1.36	***-1.48***	***-1.69***
CBU_1444	nuoE	NADH dehydrogenase subunit E	***-1.75***	***-1.63***	***-1.49***	***-1.72***
CBU_1445	nuoD	NADH dehydrogenase subunit D	***-1.69***	***-1.67***	***-1.86***	***-2.49***
CBU_1446	nuoC	NADH dehydrogenase subunit C	-1.34	-1.26	***-1.56***	***-2.23***
CBU_1447	nuoB	NADH dehydrogenase subunit B	***-1.43***	-1.23	***-1.52***	***-1.84***
CBU_1448	nuoA	NADH dehydrogenase subunit A	-1.02	-1.04	***-1.21***	***-1.42***
CBU_1449	secG	protein translocase subunit	***-1.54***	-1.11	-1.19	***-1.51***
CBU_1450	tpiA	triosephosphate isomerase	***-1.38***	-1.23	***-1.33***	***-1.58***
CBU_1451	-	hypothetical protein	***-2.05***	-1.66	***-2.54***	***-3.96***
CBU_1457	-	hypothetical protein	-1.24	1.02	1.07	1.18
CBU_1458	-	ATPase	-1.69	-1.57	-1.61	***-2.43***
CBU_1458a	-	hypothetical protein	-1.01	-1.05	-1.53	***-1.85***
CBU_1459	-	hypothetical protein	-1.01	-1.45	-1.03	-1.00
CBU_1460	-	hypothetical protein	1.64	1.80	***2.51***	***3.43***
CBU_1269a	-	hypothetical protein	***2.23***	1.41	1.40	1.41
CBU_1463	-	hypothetical protein	1.01	-1.16	-1.30	***-1.54***
CBU_1464	hupB	DNA-binding protein HU	1.16	-1.04	-1.12	-1.16
CBU_1465	-	hypothetical protein	-1.37	-1.27	***-1.48***	***-1.93***
CBU_1466	-	hypothetical protein	***-1.55***	***-1.35***	***-1.33***	***-1.74***
CBU_1467	tldD	protease TldD	-1.27	-1.17	-1.37	***-1.77***
CBU_1468	-	hypothetical protein	1.37	-1.17	1.23	1.28
CBU_1469	mreD	rod shape-determining protein	-1.12	-1.30	-1.30	***-1.56***
CBU_1470	mreC	rod shape-determining protein MreC	1.06	-1.16	-1.30	***-1.65***
CBU_1471	mreB	rod shape-determining protein MreB	-1.02	-1.03	-1.07	-1.28
CBU_1472	-	hypothetical protein	1.10	-1.14	-1.22	1.28
CBU_1473	gatC	glutamyl-tRNA(Gln) and/or aspartyl-tRNA(Asn) amidotransferase subunit C	***-1.84***	-1.20	***-1.50***	***-2.04***
CBU_1474	gatA	aspartyl/glutamyl-tRNA amidotransferase subunit A	-1.23	-1.33	-1.50	***-1.60***
CBU_1475	gatB	aspartyl/glutamyl-tRNA amidotransferase subunit B	-1.32	-1.38	***-1.72***	***-1.88***
CBU_1476	oxyR	hydrogen peroxide-inducible genes activator	-1.40	-1.47	***-1.53***	-1.19
CBU_1477	-	anti-oxidant AhpCTSA family protein	1.58	-1.09	1.14	***7.37***
CBU_1478	ahpD	alkylhydroperoxidase	1.00	-1.18	-1.33	1.99
CBU_1479	-	hypothetical protein	-1.35	-1.57	-1.41	-1.28
CBU_1480	-	hypothetical protein	-1.26	-1.36	***-1.68***	***-1.83***
CBU_1481	-	hypothetical protein	-1.31	-1.33	-1.38	***-1.97***
CBU_1482	-	hypothetical protein	-1.07	-1.05	1.01	-1.12
CBU_1483	-	nfeD family protein	1.49	1.44	1.61	1.36
CBU_1484	-	MFS superfamily transporter	1.23	1.12	1.26	1.10
CBU_1485	-	cyclin	1.23	1.11	-1.21	***-1.73***
CBU_1486	-	hypothetical protein	1.09	-1.10	1.06	1.38
CBU_1487	cysS	cysteinyl-tRNA synthetase	-1.02	-1.14	***-1.53***	***-1.59***
CBU_1488	gltX-2	glutamyl-tRNA synthetase	***-2.10***	-1.20	-1.54	***-2.89***
CBU_1489	lpxH	UDP-2,3-diacylglucosamine hydrolase	-1.04	1.05	-1.18	-1.62
CBU_1490	higA	addiction module antidote protein	1.10	1.10	1.12	***1.32***
CBU_1491	-	plasmid maintenance system killer protein	1.05	-1.04	-1.17	1.03
CBU_1493	-	hypothetical protein	1.09	1.17	1.18	1.45
CBU_1494	pdxJ	pyridoxine 5'-phosphate synthase	***1.66***	***1.76***	***2.13***	***2.35***
CBU_1497	-	hypothetical protein	1.25	-1.42	***-1.70***	***-1.46***
CBU_1501	recO	DNA repair protein	-1.22	***-1.90***	-1.44	***-1.60***
CBU_1502	era	GTP-binding protein Era	1.14	-1.39	-1.48	***-1.90***
CBU_1503	rnc	ribonuclease III	-1.07	-1.29	-1.37	-1.29
CBU_1504	lepB-2	signal peptidase I	1.14	-1.05	-1.01	-1.01
CBU_1505	lepA	GTP-binding protein LepA	***-1.46***	***-1.38***	***-1.56***	***-2.36***

NCBI RSA 493 mapping	NCBI syn	Description	5	7	14	21
CBU_1506	-	thioesterase	-1.13	-1.15	-1.14	-1.30
CBU_1507	-	copper-exporting ATPase	1.17	1.06	1.19	1.23
CBU_1508	-	ATPase	-1.00	-1.01	-1.10	***-1.38***
CBU_1509	tilS	tRNA(Ile)-lysidine synthase	1.05	***-1.62***	***-1.69***	***-2.08***
CBU_1510	accA	acetyl-CoA carboxylase carboxyltransferase subunit alpha	-1.29	-1.26	***-1.49***	***-1.98***
CBU_1511	-	ZIP family protein	***-1.83***	-1.09	-1.16	***-1.77***
CBU_1512	-	rhodanese domain-containing protein	1.44	***1.59***	***2.64***	***3.36***
CBU_1513	-	short chain dehydrogenase/reductase oxidoreductase	***1.75***	***1.81***	***1.99***	***2.26***
CBU_1514	-	short chain dehydrogenase/reductase oxidoreductase	-1.16	1.11	-1.23	-1.53
CBU_1515	-	hypothetical protein	1.32	***1.81***	***1.89***	1.51
CBU_1516	-	hypothetical protein	***2.68***	***3.09***	***3.41***	***4.79***
CBU_1517	-	RNA methyltransferase	***2.67***	***2.71***	***3.63***	***5.47***
CBU_1518	gpsA	NAD(P)H-dependent glycerol-3-phosphate dehydrogenase	1.16	-1.01	-1.03	-1.10
CBU_1519	secB	preprotein translocase subunit SecB	1.15	-1.06	-1.05	-1.01
CBU_1520	grxC	glutaredoxin	1.00	1.03	-1.03	-1.06
CBU_1521	-	rhodanese domain-containing protein	1.06	-1.07	-1.14	-1.25
CBU_1522	-	hypothetical protein	-1.80	***-2.29***	-1.52	-1.94
CBU_1529	-	peptidase catalytic domain protein of S9A/B/C families	-1.40	1.09	1.01	-1.30
CBU_1530	-	hypothetical protein	1.25	***1.79***	***2.21***	***2.15***
CBU_1530a	-	hypothetical protein	-1.00	-1.42	-1.53	-1.69
CBU_1536	yibO	phosphoglyceromutase	-1.53	-1.22	-1.44	***-1.95***
CBU_1537	-	peptidase M23/M37 domain-containing protein	1.04	-1.08	-1.42	***-1.65***
CBU_1538	-	peptidase	1.38	1.23	1.36	***1.42***
CBU_1539	-	tyrosine-specific transport protein	1.01	-1.17	1.01	1.03
CBU_1540	-	hypothetical protein	1.14	-1.35	-1.24	1.01
CBU_1541	-	hypothetical protein	-1.05	-1.50	-1.61	-1.83
CBU_1542	-	hypothetical protein	1.13	1.14	1.30	1.30
CBU_1543	-	hypothetical protein	1.29	1.47	***1.64***	***1.95***
CBU_1547	thyA	thymidylate synthase	-1.41	-1.11	-1.15	***-1.87***
CBU_1548	-	hypothetical protein	-1.08	1.08	-1.11	***-1.32***
CBU_1549	lgt	prolipoprotein diacylglyceryl transferase	***-1.55***	***-1.40***	***-1.68***	***-2.61***
CBU_1550	ptsP	phosphoenolpyruvate-protein phosphotransferase	1.17	1.10	1.20	1.11
CBU_1551	-	dinucleoside polyphosphate hydrolase	1.07	1.04	1.02	1.12
CBU_1552	-	hypothetical protein	1.72	1.49	1.17	1.45
CBU_1552a	-	hypothetical protein	1.29	1.51	1.12	-1.32
CBU_1553	nrdA	ribonucleotide-diphosphate reductase subunit alpha	1.17	1.38	1.39	1.06
CBU_1554	nrdB	ribonucleotide-diphosphate reductase subunit beta	1.32	1.35	1.13	1.05
CBU_1556	-	hypothetical protein	-2.78	-1.24	-1.13	***-4.02***
CBU_1558	-	FmdB transcriptional regulator family	-1.14	1.09	1.04	-1.08
CBU_1559	-	hypothetical protein	-1.33	-1.20	-1.31	***-1.40***
CBU_1560	-	hypothetical protein	***1.74***	1.44	***1.77***	***2.14***
CBU_1561	-	hypothetical protein	***2.89***	***2.83***	***4.12***	***5.17***
CBU_1565	aspS	aspartyl-tRNA synthetase	***-1.47***	-1.22	***-1.46***	***-1.87***
CBU_1566	-	hypothetical protein	-1.50	-1.56	***-1.83***	***-1.89***
CBU_1567	ruvC	Holliday junction resolvase	-1.20	-1.16	***-1.59***	***-1.81***
CBU_1568	ruvA	Holliday junction DNA helicase RuvA	1.05	1.03	1.02	1.17
CBU_1569	-	hypothetical protein	1.17	-1.21	1.03	1.04
CBU_1570	ruvB	Holliday junction DNA helicase RuvB	-1.16	-1.04	-1.17	***-1.73***
CBU_1573	-	MFS superfamily transporter	-1.00	-1.04	-1.02	-1.02
CBU_1574	tolQ	TolQ	1.25	1.17	1.22	1.02
CBU_1575	tolR	protein TolR	1.07	-1.10	-1.08	-1.14
CBU_1576	-	hypothetical protein	***-1.57***	-1.07	-1.43	***-1.68***
CBU_1577	-	LysM domain-containing protein	-1.06	***-1.48***	-1.17	***-1.32***
CBU_1578	-	hypothetical protein	-1.02	-1.31	-1.40	***-1.58***
CBU_1579	-	NAD(P)H:quinone oxidoreductase, type IV	***-1.73***	1.02	***-1.37***	***-2.39***
CBU_1580	-	ATPase	-1.39	-1.47	***-1.63***	***-1.68***
CBU_1581	-	hypothetical protein	-1.01	-1.13	1.44	1.56
CBU_1582	shaG	Na(+)/H(+) antiporter subunit G	-1.36	-1.63	-1.39	***-1.69***
CBU_1583	shaF	Na(+)/H(+) antiporter subunit F	1.02	-1.03	1.02	-1.19
CBU_1584	shaE	Na(+)/H(+) antiporter subunit E	-1.02	-1.21	-1.35	***-1.71***
CBU_1585	shaD	Na(+)/H(+) antiporter subunit D	-1.18	-1.32	-1.38	***-1.87***
CBU_1586	-	Na(+)/H(+) antiporter subunit C	-1.12	-1.06	-1.16	-1.54
CBU_1587	shaB	monovalent cation/H+ antiporter subunit B	-1.08	1.06	-1.23	***-1.60***
CBU_1588	shaA	monovalent cation/H+ antiporter subunit A	-1.13	1.00	1.06	-1.32
CBU_1589	-	hypothetical protein	1.38	1.13	1.02	1.06
CBU_1590	nhaP.2	Na(+)/H(+) antiporter NhaP	-1.47	-1.43	-1.47	-1.99
CBU_1593	rpsU	30S ribosomal protein S21	1.02	-1.01	-1.17	-1.19
CBU_1594	-	GatB/Yqey domain-containing protein	***-1.56***	-1.28	***-1.63***	***-2.37***
CBU_1595	dnaG	DNA primase	-1.22	1.01	-1.41	***-1.77***
CBU_1596	rpoD	RNA polymerase sigma factor	***1.46***	***1.46***	***1.67***	***2.63***

NCBI RSA 493 mapping	NCBI syn	Description	5	7	14	21
CBU_1597	-	lipoprotein	-1.89	-1.83	***-2.65***	***-3.86***
CBU_1600	-	opacity family porin protein	-1.40	-1.18	-1.40	***-1.99***
CBU_1601	-	hypothetical protein	-1.09	1.14	1.01	-1.40
CBU_1602	rimK	ribosomal protein S6 modification protein	1.16	1.01	-1.12	-1.46
CBU_1603	-	succinylglutamate desuccinylase/aspartoacylase	-1.27	-1.48	-1.74	***-2.13***
CBU_1607	-	hypothetical protein	1.32	1.21	1.22	1.47
CBU_1612	-	hypothetical protein	1.82	2.00	1.79	1.72
CBU_1613	-	hypothetical protein	1.22	-1.11	1.54	1.40
CBU_1614	-	hypothetical protein	-2.26	-1.65	-1.15	-1.35
CBU_1618	-	hypothetical protein	***-2.40***	-1.83	-1.56	-1.55
CBU_1621	-	hypothetical protein	***-1.47***	***-1.76***	***-1.56***	***-1.70***
CBU_1622	icmB	IcmB	-1.04	-1.01	***1.73***	***1.82***
CBU_1623	icmJ	IcmJ	1.06	1.20	***1.50***	***2.07***
CBU_1624	icmD	hypothetical protein	1.28	1.30	***1.69***	***1.96***
CBU_1625	icmC	hypothetical protein	1.08	1.01	1.39	***1.60***
CBU_1626	icmG	hypothetical protein	1.10	1.30	***2.13***	***2.57***
CBU_1627	icmE	IcmE	-1.15	1.06	1.73	***2.07***
CBU_1628	icmK	IcmK	1.02	1.23	***1.95***	***2.21***
CBU_1629	icmL.1	IcmL	-1.12	-1.05	1.42	1.48
CBU_1630	icmL.2	IcmL	-1.01	1.01	1.45	***1.58***
CBU_1631	icmN	ompA family protein	1.06	1.11	1.60	1.52
CBU_1632	icmO	IcmO	-1.01	1.20	***2.11***	***2.33***
CBU_1633	icmP	IcmP	-1.05	1.16	***1.85***	***1.88***
CBU_1634	icmQ	hypothetical protein	1.14	***1.59***	***2.16***	***2.47***
CBU_1635	-	hypothetical protein	1.72	1.61	-1.02	1.75
CBU_1636	-	hypothetical protein	1.16	1.30	-1.03	1.04
CBU_1638	-	ATPase	-1.02	-1.01	1.12	1.12
CBU_1639	-	hypothetical protein	1.25	1.59	1.24	1.20
CBU_1641	icmT	IcmT protein	1.32	1.31	***1.53***	***1.60***
CBU_1642	icmS	IcmS protein	1.23	-1.01	1.08	1.29
CBU_1643	dotD	DotD	1.25	***1.60***	***1.84***	***1.98***
CBU_1644	dotC	DotC	-1.10	1.23	***1.81***	***2.16***
CBU_1645	dotB	Dot/Icm secretion system ATPase DotB	1.08	-1.14	1.12	1.19
CBU_1646	-	transporter	1.18	-1.22	-1.01	1.05
CBU_1647	-	hypothetical protein	1.24	1.28	***1.58***	***1.88***
CBU_1648	dotaA	DotA protein	1.16	1.46	***1.88***	***2.16***
CBU_1649	icmV	hypothetical protein	-1.07	1.42	***1.82***	***2.00***
CBU_1650	icmW	IcmW	-1.17	1.16	***1.46***	1.31
CBU_1651	-	hypothetical protein	1.09	1.22	1.21	1.23
CBU_1652	icmX	IcmX	1.23	***1.58***	***1.64***	***1.63***
CBU_1653a	-	virulence-associated protein C	-1.43	-1.77	-1.90	***-3.62***
CBU_1655	rfaE	bifunctional heptose 7-phosphate kinase/heptose 1-phosphate adenyltransferase	-1.43	-1.07	***-1.78***	***-2.71***
CBU_1656	-	hypothetical protein	-1.55	-1.60	***-2.75***	***-2.35***
CBU_1657	-	alpha-L-glycero-D-manno-heptose beta-1,4-glucosyltransferase	***-1.48***	-1.19	***-1.35***	***-1.50***
CBU_1658	-	hypothetical protein	-1.46	-1.35	-1.78	***-2.90***
CBU_1659	-	O-antigen ligase	-1.13	-1.17	***-1.84***	***-2.67***
CBU_1660	-	hypothetical protein	1.02	-1.19	-1.62	-1.97
CBU_1661	rfaF	lipopolysaccharide heptosyltransferase II	-1.04	-1.01	1.09	-1.05
CBU_1662	-	hypothetical protein	-1.07	-1.10	-1.24	***-1.56***
CBU_1663	-	hypothetical protein	1.43	1.36	1.57	1.55
CBU_1664	-	hypothetical protein	-1.09	-1.08	-1.11	***-1.43***
CBU_1665	-	hypothetical protein	1.22	1.07	-1.17	-1.07
CBU_1669	rpoS	RNA polymerase sigma factor	***2.14***	***2.17***	***2.69***	***2.82***
CBU_1670	-	lipoprotein NlpD	-1.23	-1.18	***-1.71***	***-1.52***
CBU_1671	surE	stationary phase survival protein SurE	-1.28	-1.07	-1.11	***-1.54***
CBU_1673	ftsB	cell division protein ftsB	***-1.59***	-1.33	-1.28	***-1.60***
CBU_1674	eno	phosphopyruvate hydratase	-1.14	-1.09	1.00	1.20
CBU_1675	kdsA	2-dehydro-3-deoxyphosphooctonate aldolase	***-1.82***	-1.41	***-1.55***	***-2.34***
CBU_1676	-	hypothetical protein	***1.46***	1.29	***1.43***	***1.64***
CBU_1677	-	hemerythrin HHE cation binding domain protein	***2.09***	***2.68***	***4.01***	***5.52***
CBU_1678	speG	diamine N-acetyltransferase	***1.87***	***1.85***	***2.87***	***3.51***
CBU_1681	-	hypothetical protein	***4.23***	***5.29***	***7.02***	***9.73***
CBU_1682	pyrG	CTP synthetase	1.08	-1.01	1.00	-1.20
CBU_1683	-	hypothetical protein	-2.22	-1.51	-1.86	-1.03
CBU_1685	-	hypothetical protein	1.32	-1.19	1.38	1.69
CBU_1686	-	hypothetical protein	1.22	-1.09	1.33	1.35
CBU_1688	dcd	deoxycytidine triphosphate deaminase	***-1.47***	-1.05	-1.29	***-1.53***
CBU_1689	apbC	NifH/FrxC domain protein	1.08	1.14	1.21	-1.01
CBU_1690	-	hypothetical protein	1.82	1.20	1.28	1.40
CBU_1691	-	DNA-binding protein	1.20	1.16	-1.03	1.18

NCBI RSA 493 mapping	NCBI syn	Description	5	7	14	21
CBU_1692	-	phage derived Gp49-like protein	-1.03	1.17	1.26	1.19
CBU_1695	metG	methionyl-tRNA synthetase	-1.29	1.08	-1.33	-1.67
CBU_1696	rnfB	4Fe-4S ferredoxin	-1.12	-1.06	-1.50	-1.32
CBU_1697	nth	endonuclease III	-1.08	-1.09	-1.05	-1.33
CBU_1698	-	ATPase	-1.56	-1.11	-1.37	-1.83
CBU_1699	-	hypothetical protein	-1.16	1.29	-1.72	-2.18
CBU_1701	-	hypothetical protein	-1.85	-1.48	-1.89	-2.66
CBU_1702	-	hypothetical protein	-1.24	-1.36	-1.67	-1.58
CBU_1703	pyrC	dihydroorotase	-1.12	-1.10	-1.34	-1.54
CBU_1704	rnt	ribonuclease T	-1.57	-1.48	-2.20	-3.18
CBU_1705	-	hypothetical protein	-1.35	1.09	-1.40	-1.63
CBU_1706	-	anti-oxidant AhpCTSA family protein	-1.19	1.05	1.12	1.64
CBU_1707	-	hypothetical protein	-1.21	-1.19	-1.81	-2.65
CBU_1708	sodB	superoxide dismutase	-1.19	1.08	-1.42	-1.89
CBU_1709	dapB	dihydrodipicolinate reductase	-1.14	1.05	-1.40	-1.63
CBU_1710	-	hypothetical protein	-1.57	-1.56	-1.78	-2.10
CBU_1711	-	hypothetical protein	-1.72	-1.73	-2.05	-2.95
CBU_1712	-	GIY-YIG catalytic domain-containing protein	-1.98	-1.38	-1.50	-2.20
CBU_1713	-	glycine dehydrogenase subunit 2	-1.46	-1.51	-1.75	-2.28
CBU_1714	-	glycine dehydrogenase subunit 1	-2.08	-1.68	-2.11	-3.00
CBU_1715	gcvH	glycine cleavage system protein H	-2.22	-1.51	-2.42	-4.73
CBU_1716	gcvT	glycine cleavage system aminomethyltransferase T	-2.20	-1.73	-2.28	-4.18
CBU_1718	groEL	molecular chaperone GroEL	-2.21	1.03	-1.24	-2.05
CBU_1719	groES	co-chaperonin GroES	1.28	1.02	-1.05	-1.22
CBU_1720	acnA	aconitate hydratase	-1.86	-1.37	-1.69	-2.74
CBU_1721	-	hypothetical protein	-1.15	-1.42	-1.31	-1.55
CBU_1723	dsbD	thiol:disulfide interchange protein	1.27	1.12	1.58	1.46
CBU_1724	-	hypothetical protein	1.57	1.92	2.44	3.06
CBU_1724a	-	hypothetical protein	-1.43	-1.01	-1.44	-1.85
CBU_1725	accB	biotin carboxyl carrier protein of acetyl-CoA carboxylase	-1.15	1.16	-1.02	-1.12
CBU_1726	accC	biotin carboxylase	-1.22	-1.29	-1.73	-1.71
CBU_1727	arcB	ornithine cyclodeaminase	-3.09	-1.38	-2.80	-3.56
CBU_1728	-	nicotinamide mononucleotide transporter	-3.51	-2.15	-3.29	-6.13
CBU_1729	hemF	coproporphyrinogen III oxidase	-1.19	-1.18	-1.41	-1.85
CBU_1730	-	HAD hydrolase	-1.98	-1.29	-1.70	-2.17
CBU_1732	-	D-3-phosphoglycerate dehydrogenase	-2.84	-1.78	-2.62	-5.17
CBU_1733	-	hypothetical protein	-1.03	-1.07	-1.21	-1.14
CBU_1734	-	chromosome replication initiator DnaA	-1.15	-1.14	-1.18	-1.33
CBU_1735	-	hypothetical protein	1.05	1.01	1.00	1.01
CBU_1736	purM	phosphoribosylaminoimidazole synthetase	-1.96	-1.72	-2.47	-3.38
CBU_1737	purN	phosphoribosylglycinamide formyltransferase	-1.13	-1.37	-1.48	-1.88
CBU_1738	hipB	integration host factor beta-subunit	1.50	1.39	1.50	1.99
CBU_1739	-	tetrapyrrole methylase domain-containing protein	1.01	-1.15	-1.12	-1.24
CBU_1740	-	hypothetical protein	-1.11	-1.30	-1.46	-1.73
CBU_1741	-	lipoprotein	-1.26	-1.07	-1.11	-1.57
CBU_1742	-	hypothetical protein	-2.07	-1.82	-2.43	-4.19
CBU_1743	gmhA	phosphoheptose isomerase	-1.23	-1.04	-1.13	-1.55
CBU_1744	-	phospholipid-binding liporotein	-1.11	-1.17	-1.43	-1.89
CBU_1745	-	peptidase, M50 family protein	1.39	1.10	1.45	1.57
CBU_1746	sspB	ClpXP protease specificity-enhancing factor	-1.07	-1.15	-1.45	-1.38
CBU_1747	sspA	stringent starvation protein A	-1.29	-1.12	-1.38	-1.35
CBU_1748	rpsI	30S ribosomal protein S9	1.03	-1.09	1.02	1.13
CBU_1749	rplM	50S ribosomal protein L13	-1.53	-1.23	-1.56	-1.73
CBU_1749a	-	hypothetical protein	1.19	1.06	-1.08	-1.16
CBU_1751	-	hypothetical protein	1.01	1.75	1.92	1.51
CBU_1752	-	hypothetical protein	-1.24	1.23	1.28	1.41
CBU_1753	-	hypothetical protein	-1.03	-1.03	-1.30	-1.58
CBU_1754	-	hypothetical protein	1.15	1.36	1.52	1.43
CBU_1755	-	hypothetical protein	1.47	1.33	1.44	2.12
CBU_1756	-	Bcr/CflA subfamily drug resistance transporter	-1.31	-1.03	-1.16	-1.36
CBU_1760	-	hypothetical protein	-1.13	-1.05	-1.12	-1.54
CBU_1761	-	two component system histidine kinase	-1.21	-1.13	-1.32	-1.29
CBU_1762	-	FAD dependent oxidoreductase	1.10	1.21	1.24	1.36
CBU_1763	-	ATPase	-1.38	-1.64	-1.31	-2.22
CBU_1764	-	hypothetical protein	1.38	1.07	1.19	1.31
CBU_1764a	-	hypothetical protein	1.18	1.19	1.10	1.00
CBU_1765	-	hypothetical protein	1.28	1.55	1.94	2.14
CBU_1766	feoB	ferrous iron transport protein B	-1.15	1.40	1.68	1.98
CBU_1767	feoA	ferrous iron transport protein A	1.27	1.37	1.68	1.72
CBU_1768	-	hypothetical protein	1.50	2.41	2.63	3.16

NCBI RSA 493 mapping	NCBI syn	Description	5	7	14	21
CBU_1769	-	alpha/beta hydrolase	-1.12	1.04	-1.22	-1.44
CBU_1770	-	ABC transporter ATP-binding protein	-1.59	1.00	-1.27	-1.50
CBU_1771	-	ABC transporter permease	-1.32	-1.14	-1.20	***-1.64***
CBU_1772	engB	ribosome biogenesis GTP-binding protein YsxC	***-1.72***	-1.20	***-1.78***	***-2.50***
CBU_1777	-	hypothetical protein	-1.26	-1.45	***-2.42***	***-2.39***
CBU_1778	fbaA	fructose-1,6-bisphosphate aldolase	-1.35	-1.29	***-1.60***	***-2.69***
CBU_1780	-	hypothetical protein	-1.32	-1.14	-1.01	-1.80
CBU_1781	pyk	pyruvate kinase	***-1.78***	-1.20	-1.19	***-1.47***
CBU_1782	pgk	phosphoglycerate kinase	-1.18	-1.12	***-1.37***	***-1.32***
CBU_1783	gap	glyceraldehyde 3-phosphate dehydrogenase	***-1.49***	-1.16	***-1.45***	***-1.50***
CBU_1784	tkt	transketolase	***-1.91***	-1.29	***-1.75***	***-2.51***
CBU_1786	-	ATPase	***1.46***	1.35	***1.54***	***1.77***
CBU_1787	glmS	glutamine-fructose-6-phosphate transaminase (isomerizing)	-1.41	-1.29	***-1.83***	***-2.05***
CBU_1788	-	hypothetical protein	-1.32	-1.50	***-1.89***	***-2.31***
CBU_1789	-	SMP-30/CGR1 family protein	***1.94***	***2.41***	***3.66***	***4.87***
CBU_1790	-	hypothetical protein	***-2.10***	***-1.62***	***-2.07***	***-2.26***
CBU_1794	-	hypothetical protein	1.09	***3.42***	***3.76***	***2.68***
CBU_1795	polI	DNA polymerase I	-1.18	-1.44	-1.31	-1.51
CBU_1796	-	amino acid permease	1.01	-1.36	-1.21	-1.56
CBU_1797	-	thioesterase	1.37	-1.36	-1.61	-1.60
CBU_1798	-	hypothetical protein	-1.27	-1.29	***-1.49***	***-2.21***
CBU_1799	-	acetyltransferase	-1.05	1.04	1.07	1.16
CBU_1800	-	hypothetical protein	1.20	1.16	1.21	1.30
CBU_1801	-	hypothetical protein	1.06	1.27	1.28	1.55
CBU_1802	-	hypothetical protein	1.14	1.02	***1.21***	***1.35***
CBU_1803	-	hypothetical protein	1.00	1.14	-1.07	1.06
CBU_1804	-	LuxR family transcriptional regulator	1.32	***1.59***	-1.41	-1.16
CBU_1805	-	LuxR family transcriptional regulator	***1.64***	***1.90***	1.13	1.24
CBU_1806	-	DNA polymerase domain-containing protein	1.06	1.04	1.24	1.52
CBU_1808	-	export ABC transporter permease	1.02	-1.25	-1.54	-1.57
CBU_1809	-	ABC transporter ATP-binding protein	-1.31	-1.43	-1.58	***-2.20***
CBU_1810	macA	ABC transporter substrate-binding protein	1.04	1.18	-1.18	-1.49
CBU_1811	-	type I secretion outer membrane protein	-1.09	1.06	-1.02	-1.15
CBU_1812	-	erythronate-4-phosphate dehydrogenase	-1.19	1.39	***1.96***	***1.47***
CBU_1813	-	hypothetical protein	1.04	-1.07	-1.06	-1.30
CBU_1814	-	hypothetical protein	1.09	1.01	***-1.47***	***-3.39***
CBU_1815	priA	primosomal protein N'	1.00	-1.13	1.12	-1.01
CBU_1816	efp	elongation factor P	-1.08	1.09	-1.21	***-1.50***
CBU_1817	-	lysyl-tRNA synthetase	-1.02	-1.24	-1.36	***-1.75***
CBU_1818	-	hypothetical protein	***-2.37***	-1.79	-1.65	***-1.79***
CBU_1819	-	hypothetical protein	-1.32	-1.14	-1.35	-1.19
CBU_1820	-	hypothetical protein	-1.28	***-1.42***	***-1.47***	***-1.86***
CBU_1821	-	hypothetical protein	1.05	-1.02	1.05	***-1.26***
CBU_1822	sodC	superoxide dismutase	***1.77***	***1.72***	***2.12***	***2.47***
CBU_1823	-	hypothetical protein	1.01	1.06	1.27	1.45
CBU_1824	-	hypothetical protein	1.10	-1.53	-1.56	-1.57
CBU_1825	-	hypothetical protein	1.14	-1.28	-1.22	1.22
CBU_1826	psd	phosphatidylserine decarboxylase	***-2.47***	-1.65	***-2.18***	***-3.33***
CBU_1827	-	multifunctional CCA protein	-1.32	-1.15	***-1.56***	***-2.04***
CBU_1828	-	hypothetical protein	-1.49	-1.47	***-1.73***	***-2.22***
CBU_1829	lolB	hypothetical protein	1.01	1.10	1.13	-1.08
CBU_1830	prsA	ribose-phosphate pyrophosphokinase	***-1.94***	-1.44	***-2.38***	***-3.86***
CBU_1831	-	hypothetical protein	-1.62	-1.39	***-1.94***	***-2.76***
CBU_1833	-	hypothetical protein	1.09	-1.11	-1.17	-1.36
CBU_1834	rmlA	glucose-1-phosphate thymidylyltransferase	-1.32	***-1.61***	***-2.20***	***-3.25***
CBU_1835	-	amine oxidase	-1.47	***-1.64***	***-1.96***	***-2.28***
CBU_1836	-	homoserine dehydrogenase, NAD binding domain	***-1.72***	-1.31	***-1.70***	***-2.33***
CBU_1837	-	NAD-dependent epimerase/dehydratase	-1.45	***-1.56***	***-1.94***	***-2.92***
CBU_1838	rfbC	dTDP-4-dehydrorhamnose 3,5-epimerase	***-2.43***	***-1.87***	***-2.18***	***-4.33***
CBU_1839	-	aminobutyraldehyde dehydrogenase	***-2.43***	***-1.71***	***-2.40***	***-5.34***
CBU_1840	-	50S ribosomal protein L25	***-1.55***	-1.00	***-2.12***	***-4.04***
CBU_1841	pth	peptidyl-tRNA hydrolase	-2.09	-1.36	***-2.19***	***-3.59***
CBU_1842	-	GTP-dependent nucleic acid-binding protein EngD	-1.23	-1.69	-1.83	***-2.42***
CBU_1843	-	hypothetical protein	***-1.80***	-1.57	***-1.98***	***-2.52***
CBU_1843a	-	hypothetical protein	-1.35	-1.21	-2.81	-1.49
CBU_1845	-	hypothetical protein	1.08	1.13	-1.23	-1.47
CBU_1847	-	hypothetical protein	***-2.61***	-1.29	-1.44	***-1.74***
CBU_1847b	-	hypothetical protein	***2.05***	***2.01***	***2.22***	***2.70***
CBU_1849	-	hypothetical protein	1.24	-1.02	-1.22	-1.35
CBU_1850	-	hypothetical protein	-1.04	-1.15	-1.30	***-1.32***

NCBI RSA 493 mapping	NCBI syn	Description	5	7	14	21
CBU_1851	-	hypothetical protein	***-1.47***	-1.41	***-1.54***	***-1.48***
CBU_1852	-	hypothetical protein	-1.44	-1.53	***-1.60***	***-1.92***
CBU_1853	-	GtrA family protein	-1.32	-1.26	-1.32	-1.41
CBU_1854	-	hypothetical protein	1.01	1.00	***-1.34***	***-1.45***
CBU_1855	pilF	fimbrial biogenesis domain-containing protein	-1.09	-1.10	-1.19	-1.17
CBU_1856	-	3-hydroxyisobutyryl-CoA hydrolase	1.09	1.24	1.22	1.34
CBU_1857	oppD	oligopeptide transport ATP-binding protein	1.34	-1.01	-1.19	1.03
CBU_1858	oppC	oligopeptide transport system permease	1.55	-1.73	-1.11	1.30
CBU_1859	oppB	oligopeptide ABC transporter permease	-2.11	-1.48	-1.32	-1.48
CBU_1862	-	radical SAM domain-containing protein	-1.37	-1.29	***-1.76***	***-1.85***
CBU_1863	-	hypothetical protein	***-2.10***	***-1.59***	***-1.78***	***-2.19***
CBU_1864	-	hypothetical protein	-1.00	-1.25	1.06	1.12
CBU_1865	-	hypothetical protein	1.38	1.48	***1.73***	***1.81***
CBU_1866	parC	DNA topoisomerase IV subunit A	-1.14	-1.06	1.02	-1.01
CBU_1867	-	hypothetical protein	1.06	-2.19	-1.32	1.51
CBU_1867a	-	hypothetical protein	-1.16	-1.05	1.11	1.47
CBU_1869	-	hypothetical protein	***1.48***	***1.35***	***1.39***	***1.32***
CBU_1870	-	2-polyprenyl-3-methyl-5-hydroxy-6-metoxy-1,4-benzoquinol methylase	1.24	1.16	1.39	***1.80***
CBU_1871	pabA	anthranilate synthase component II	1.27	1.02	-1.22	-1.01
CBU_1872	rpe	ribulose-phosphate 3-epimerase	-1.24	***-1.51***	***-1.55***	***-2.01***
CBU_1873	djlA	Dna-J like membrane chaperone protein	-1.34	-1.39	-1.25	-1.40
CBU_1874	-	glutamate--cysteine ligase	1.19	1.49	***1.85***	***2.18***
CBU_1875	gshB	glutathione synthetase	-1.01	1.28	1.58	1.83
CBU_1876	-	SAM-dependent methyltransferase	-1.08	-1.17	-1.27	-1.22
CBU_1877	-	ATPase	-1.16	1.01	-1.01	1.09
CBU_1878	-	iron-sulfur cluster insertion protein ErpA	***1.38***	1.22	***1.24***	***1.29***
CBU_1879	-	peptide deformylase	-1.26	1.02	-1.08	-1.21
CBU_1880	-	hypothetical protein	-1.14	-1.07	-1.10	-1.28
CBU_1881	-	rubredoxin	***-1.82***	-1.22	***-2.26***	***-3.63***
CBU_1882	hemL	glutamate-1-semialdehyde aminotransferase	1.08	1.10	-1.02	-1.31
CBU_1883	queD	queuosine biosynthesis protein	-1.33	1.06	1.01	-1.32
CBU_1884	-	hypothetical protein	-1.19	-1.13	-1.17	***-1.34***
CBU_1886	-	hypothetical protein	1.28	1.08	-1.41	-1.46
CBU_1887	ponA	multimodular transpeptidase-transglycosylase PBP 1A	-1.02	1.06	1.05	-1.32
CBU_1888	-	hypothetical protein	***-3.41***	-2.03	***-4.82***	***-5.72***
CBU_1890	-	hypothetical protein	-2.79	-2.98	-2.42	-1.75
CBU_1891	pilQ	type II and III secretion system family protein	-1.39	-1.03	-1.07	-1.47
CBU_1892	aroK	shikimate kinase	1.09	1.04	-1.15	-1.08
CBU_1893	aroB	3-dehydroquinate synthase	1.04	-1.04	-1.13	-1.48
CBU_1894	-	hypothetical protein	1.24	1.13	1.25	1.41
CBU_1895	-	hypothetical protein	1.21	1.01	1.11	1.18
CBU_1896	-	major facilitator family transporter	***-1.81***	-1.15	-1.27	***-1.55***
CBU_1899	-	methyltransferase	1.22	-1.04	-1.34	-1.22
CBU_1901	-	M16 family peptidase	-1.29	-1.16	-1.18	-1.69
CBU_1902	-	M16 family peptidase	***-1.53***	-1.08	-1.08	***-1.49***
CBU_1903	ftsY	cell division protein	1.39	1.21	1.32	1.38
CBU_1904	ftsE	cell division ATP-binding protein	-1.10	-1.41	-1.52	***-1.94***
CBU_1905	ftsX	ABC transporter permease	1.31	1.11	1.23	1.12
CBU_1906	-	hypothetical protein	1.72	1.37	-1.53	-1.14
CBU_1907	-	acetyltransferase	1.10	1.20	1.32	1.17
CBU_1908	-	hypothetical protein	1.02	-1.16	-1.35	-1.18
CBU_1909	rpoH	RNA polymerase factor sigma-32	***-1.54***	-1.24	-1.36	***-1.78***
CBU_1910	com1	hypothetical protein	-1.06	-1.07	***-1.49***	***-1.42***
CBU_1911	-	hypothetical protein	1.41	1.10	1.29	***2.33***
CBU_1913	glyQ	glycyl-tRNA synthetase subunit alpha	-1.39	-1.49	-1.50	***-3.06***
CBU_1914	glyS	glycyl-tRNA synthetase subunit beta	-1.14	-1.31	***-1.60***	***-1.84***
CBU_1915	sun	ribosomal RNA small subunit methyltransferase B	-1.04	-1.40	-1.40	-1.31
CBU_1916	-	universal stress protein family protein	1.29	1.09	-1.03	-1.04
CBU_1917	rpmH	50S ribosomal protein L34	***-1.46***	-1.12	***-1.33***	***-1.39***
CBU_1918	rnpA	ribonuclease P protein component	***-1.43***	-1.12	***-1.31***	-1.18
CBU_1919	-	hypothetical protein	1.20	1.06	-1.17	-1.16
CBU_1920	yidC	inner membrane protein oxaA	***-1.78***	-1.44	***-1.94***	***-1.69***
CBU_1921	-	hypothetical protein	-1.25	***-1.83***	***-2.97***	***-2.67***
CBU_1922	trmE	tRNA modification GTPase TrmE	-1.43	-1.06	***-1.51***	***-1.92***
CBU_1924	gidA	tRNA uridine 5-carboxymethylaminomethyl modification protein GidA	-1.20	1.22	1.06	-1.12
CBU_1925	gidB	16S rRNA methyltransferase GidB	-1.17	1.07	-1.14	-1.48
CBU_1926	soj	CobQ/CobB/MinD/ParA nucleotide binding domain-containing protein	-1.00	-1.07	-1.10	***-1.43***
CBU_1927	parB	chromosome partitioning protein	-1.27	-1.29	***-1.38***	***-1.87***
CBU_1928	-	acyl-CoA synthetase	-1.38	-1.36	-1.67	***-2.73***
CBU_1929	-	major facilitator transporter	1.15	-1.55	-1.36	***-2.05***

NCBI RSA 493 mapping	NCBI syn	Description	5	7	14	21
CBU_1930	-	hypothetical protein	1.24	1.06	1.05	-1.02
CBU_1930a	-	hypothetical protein	-1.09	-1.13	***-1.36***	***-1.76***
CBU_1931	-	hypothetical protein	-1.87	***-5.62***	***-10.64***	***-9.66***
CBU_1932	-	hypothetical protein	***3.78***	***4.83***	***5.83***	***8.64***
CBU_1933	-	Ku protein	***4.99***	***5.30***	***7.53***	***9.30***
CBU_1934	-	DNA ligase D	***4.09***	***2.91***	***4.55***	***5.35***
CBU_1936	-	hypothetical protein	-2.49	-2.60	-1.30	-1.27
CBU_1938	atpI	ATP synthase F0 subunit I	-1.20	-1.49	-1.60	-1.76
CBU_1939	atpB	ATP synthase F0F1 subunit A	-1.32	-1.25	-1.60	***-2.68***
CBU_1940	atpE	ATP synthase subunit C	***-1.56***	***-1.47***	***-1.66***	***-2.04***
CBU_1941	atpF	ATP synthase F0F1 subunit B	***-1.73***	-1.32	-1.24	***-1.37***
CBU_1942	atpH	ATP synthase subunit delta	***-2.01***	-1.62	-1.56	***-1.69***
CBU_1943	atpA	ATP synthase F0F1 subunit alpha	-1.42	-1.32	-1.37	***-1.54***
CBU_1944	atpG	ATP synthase F0F1 subunit gamma	-1.62	***-1.82***	***-1.73***	***-2.24***
CBU_1945	atpD	ATP synthase F0F1 subunit beta	-1.31	-1.25	-1.25	-1.18
CBU_1946	atpC	ATP synthase F0F1 subunit epsilon	-1.14	-1.16	-1.07	-1.13
CBU_1947	glmU	UDP-N-acetylglucosamine pyrophosphorylase	-1.05	-1.10	-1.38	***-1.89***
CBU_1950	-	hypothetical protein	***1.91***	1.68	***1.68***	***2.11***
CBU_1951	-	hypothetical protein	-1.31	-1.16	***-2.14***	***-2.84***
CBU_1952	-	ABC transporter ATP-binding protein	-1.15	1.56	1.72	1.40
CBU_1953	-	hypothetical protein	-1.50	-1.38	***-2.74***	***-5.82***
CBU_1954	-	(2R)-phospho-3-sulfolactate synthase	-1.19	-1.24	***-1.70***	***-3.56***
CBU_1955	pntAA	NAD(P) transhydrogenase subunit alpha	-1.32	-1.35	***-1.66***	***-2.35***
CBU_1956	pntAB	NAD(P) transhydrogenase subunit alpha	-1.17	***-1.52***	***-2.01***	***-2.51***
CBU_1957	pntB	NAD(P) transhydrogenase subunit alpha	-1.13	-1.68	-1.76	-1.51
CBU_1960	-	16S ribosomal RNA methyltransferase RsmE	-1.88	-1.30	-2.00	-2.52
CBU_1964	hemK	peptide release factor-glutamine N5-methyltransferase	-1.01	-1.21	-1.18	-1.38
CBU_1965	prfA	peptide chain release factor 1	-1.01	-1.14	***-1.84***	***-1.88***
CBU_1966	hemA	glutamyl-tRNA reductase	***-2.36***	-1.44	***-2.16***	***-3.74***
CBU_1967	-	Bcr/CflA subfamily drug resistance transporter	1.20	1.19	-1.16	-1.02
CBU_1968	folB	dihydroneopterin aldolase	***-1.55***	-1.39	***-2.12***	***-2.95***
CBU_1969	dksA	RNA polymerase-binding protein DksA	***-1.88***	***-1.73***	***-2.29***	***-3.02***
CBU_1970	dapF	diaminopimelate epimerase	1.06	-1.11	***-1.48***	***-1.86***
CBU_1971	-	hypothetical protein	1.27	1.18	1.19	1.10
CBU_1972	-	LuxR family transcriptional regulator	-1.66	-1.17	-1.38	-1.61
CBU_1973	-	hypothetical protein	***-1.85***	-1.52	***-2.74***	***-3.60***
CBU_1975	-	phospholipase/carboxylesterase	-1.23	1.03	-1.16	***-1.47***
CBU_1976	-	nucleotidyl transferase	-1.40	-1.30	***-1.54***	***-1.97***
CBU_1977	-	phosphotransferase enzyme family protein	***-1.51***	-1.17	***-1.52***	***-2.16***
CBU_1978	ostA	organic solvent tolerance protein	-1.16	-1.20	***-1.91***	***-2.57***
CBU_1980	-	peptidyl-prolyl cis-trans isomerase surA	1.01	1.33	1.19	1.01
CBU_1981	pdxA	4-hydroxythreonine-4-phosphate dehydrogenase	1.16	-1.04	-1.09	1.06
CBU_1982	ksgA	dimethyladenosine transferase	1.06	-1.10	-1.10	-1.15
CBU_1983	uspA	universal stress protein family protein	1.07	1.05	***-1.25***	***-2.00***
CBU_1984	-	hypothetical protein	1.17	-1.00	-1.13	***-1.37***
CBU_1985	-	hypothetical protein	1.12	-1.22	***-1.74***	***-2.44***
CBU_1986	sppA	U7 family peptidase	1.16	-1.03	-1.03	-1.21
CBU_1987	apaH	diadenosine tetraphosphatase	1.24	-1.14	-1.58	***-1.98***
CBU_1987a	-	hypothetical protein	1.19	-1.17	1.23	1.62
CBU_1991	relB	RelB	-1.08	-1.07	-1.18	-1.33
CBU_1992	relE	RelE	1.00	1.10	-1.16	-1.11
CBU_1993	folA	dihydrofolate reductase	1.14	1.06	1.14	1.19
CBU_1994	-	hypothetical protein	1.24	1.21	1.34	1.14
CBU_1995	-	acylphosphatase	1.23	1.00	***1.44***	***2.20***
CBU_1996	-	D,D-heptose 1,7-bisphosphate phosphatase	1.08	1.09	1.02	-1.16
CBU_1997	fmt	methionyl-tRNA formyltransferase	1.20	1.02	1.18	1.24
CBU_1998	-	DNA protecting protein DprA	1.68	1.45	1.62	1.25
CBU_1999	smg	Smg protein	-1.06	-1.29	***-1.47***	***-1.72***
CBU_2000	topA	DNA topoisomerase I	1.21	***-1.57***	***-1.35***	-1.25
CBU_2001	-	hypothetical protein	1.18	1.06	1.25	1.32
CBU_2002	purE	phosphoribosylaminoimidazole carboxylase carboxyltransferase subunit	1.43	-1.21	-1.44	-1.45
CBU_2003	-	sua5/YciO/YrdC/YwlC family protein	1.22	-1.32	-1.58	***-1.96***
CBU_2003a	-	hypothetical protein	2.01	-1.24	2.40	1.16
CBU_2004	-	heptosyl transferase glycosyltransferase 9 protein	-1.03	-1.16	-1.49	-1.39
CBU_2005	rstB	signal transduction histidine kinase-like protein	-1.69	-2.00	-1.73	***-2.70***
CBU_2006	-	transcriptional regulatory protein	***-1.50***	***-1.55***	***-1.50***	***-1.68***
CBU_2007	-	hypothetical protein	-1.14	***-1.43***	***-1.45***	***-1.46***
CBU_2008	argS	arginyl-tRNA synthetase	***-1.72***	-1.43	***-1.76***	***-2.39***
CBU_2009	-	hypothetical protein	***-1.54***	-1.10	-1.01	-1.08
CBU_2010	-	hypothetical protein	1.04	1.01	1.13	-1.12

NCBI RSA 493 mapping	NCBI syn	Description	5	7	14	21
CBU_2011	hslV	ATP-dependent protease peptidase subunit	1.16	-1.14	-1.04	-1.03
CBU_2012	hslU	ATP-dependent protease ATP-binding subunit HslU	***-2.50***	-1.22	***-1.68***	***-2.12***
CBU_2013	-	hypothetical protein	-1.07	-1.15	-1.15	-1.22
CBU_2014	-	hypothetical protein	-1.24	-1.14	-1.02	-1.11
CBU_2016	-	hypothetical protein	1.36	***1.61***	***2.35***	***2.44***
CBU_2017	ubiE	ubiquinone/menaquinone biosynthesis methyltransferase ubiE	-1.13	-1.22	***-1.41***	***-1.85***
CBU_2018	-	hypothetical protein	-1.14	-1.27	***-1.50***	***-1.72***
CBU_2019	ubiB	2-polyprenylphenol 6-hydroxylase	-1.15	***-1.49***	***-1.72***	***-1.96***
CBU_2020	-	glutamate/gamma-aminobutyrate antiporter	-1.55	-1.16	1.16	-1.11
CBU_2021	-	ATPase	-1.17	1.10	1.15	-1.20
CBU_2023	-	hypothetical protein	***1.67***	***2.00***	***2.39***	***2.83***
CBU_2024	-	cystathionine beta-synthase	***-2.05***	***-1.46***	***-1.69***	***-2.56***
CBU_2025	metC	O-succinylhomoserine (thiol)-lyase	***-1.42***	***-1.72***	***-2.28***	***-2.74***
CBU_2027	-	hypothetical protein	***-1.24***	-1.11	-1.03	-1.17
CBU_2028	-	hypothetical protein	-1.58	1.04	-1.18	1.05
CBU_2029	-	hypothetical protein	-1.22	1.17	1.17	-1.19
CBU_2030	metK	S-adenosylmethionine synthetase	***-1.75***	-1.27	***-2.03***	***-2.86***
CBU_2031	ahcY	S-adenosyl-L-homocysteine hydrolase	***-1.66***	***-1.71***	***-2.00***	***-3.14***
CBU_2032	-	GAF domain-containing protein	-1.14	-1.32	***-1.54***	***-1.63***
CBU_2033	-	hypothetical protein	1.44	1.69	1.36	1.41
CBU_2036	-	hypothetical protein	-1.14	-1.24	1.06	1.66
CBU_2040	-	HlyD family type I secretion membrane fusion protein	1.16	-1.19	-1.03	1.85
CBU_2041	-	PAS domain-containing protein	-1.16	-1.25	-1.15	1.09
CBU_2046	-	hypothetical protein	1.21	-1.02	-1.03	1.21
CBU_2047	metF	methylenetetrahydrofolate reductase	1.33	-1.07	-1.33	-1.46
CBU_2048	metE	5-methyltetrahydropteroyltriglutamate--homocysteine S-methyltransferase	-1.00	-1.18	***-1.78***	***-1.64***
CBU_2049	trpS	tryptophanyl-tRNA synthetase	***-1.83***	-1.59	***-2.19***	***-3.99***
CBU_2051a	-	hypothetical protein	-1.22	-1.06	-1.71	-1.10
CBU_2052	-	hypothetical protein	1.18	1.15	1.14	***1.70***
CBU_2054	uvrD	DNA-dependent helicase II	***-2.03***	-1.29	***-1.81***	***-2.99***
CBU_2055	-	zinc uptake transporter	-1.26	-1.34	-1.77	***-2.45***
CBU_2056	-	hypothetical protein	-1.14	-1.10	-1.45	-1.48
CBU_2057	-	hypothetical protein	-1.35	-1.23	-1.33	-1.37
CBU_2058	-	proline/betaine transporter	1.38	1.54	***1.89***	***1.96***
CBU_2061	-	hypothetical protein	1.11	-1.05	-1.34	-1.37
CBU_2065	-	hypothetical protein	-1.28	-1.27	-1.25	-1.50
CBU_2067	-	MFS superfamily transporter	1.01	-1.11	-1.01	-1.04
CBU_2068	-	MFS superfamily transporter	-1.07	-1.30	-1.29	***-2.54***
CBU_2069	-	thymidine kinase	-1.06	-1.10	1.05	-1.16
CBU_2070	-	chorismate mutase	-1.35	-1.36	***-1.69***	***-2.24***
CBU_2071	-	hypothetical protein	***2.23***	***2.39***	***2.81***	***2.42***
CBU_2072	-	hypothetical protein	-1.21	-1.22	***-1.35***	***-1.60***
CBU_2073	-	acyltransferase	-1.24	1.05	-1.02	***-1.37***
CBU_2074	hemC	porphobilinogen deaminase	-1.17	-1.10	-1.12	***-1.39***
CBU_2075	aroD	3-dehydroquinate dehydratase	-1.26	-1.33	-1.31	***-1.51***
CBU_2076	-	hypothetical protein	-1.21	-1.38	-1.47	***-1.73***
CBU_2077	hemD	uroporphyrinogen-III synthase	-1.20	-1.56	***-1.57***	***-1.98***
CBU_2078	-	Fic family protein	-1.23	1.26	***1.77***	***1.99***
CBU_2079	-	hypothetical protein	***2.36***	***2.85***	***4.15***	***5.88***
CBU_2080	-	uroporphyrin-III C-methyltransferase	1.13	1.12	***1.71***	***1.86***
CBU_2081	hemY	protein HemY	1.06	-1.12	-1.09	-1.21
CBU_2082	-	hypothetical protein	1.13	-1.15	-1.13	-1.11
CBU_2083	queC	queuosine biosynthesis protein QueC	***-1.84***	***-1.56***	***-1.97***	***-3.10***
CBU_2084	-	hypothetical protein	1.08	1.06	-1.06	1.03
CBU_2085	-	hypothetical protein	-1.01	-1.02	-1.03	1.04
CBU_2086	rho	transcription termination factor Rho	***-1.74***	-1.24	***-1.46***	***-1.65***
CBU_2087	trx	thioredoxin	1.01	-1.11	***-1.32***	***-1.32***
CBU_2088	-	sodium:neurotransmitter symporter family protein	***-2.02***	***-1.71***	***-1.97***	***-3.54***
CBU_2089	-	hypothetical protein	-1.09	-1.37	-1.18	-1.26
CBU_2090	proC	pyrroline-5-carboxylate reductase	1.44	1.10	1.04	1.00
CBU_2091	-	hypothetical protein	-1.11	-1.15	-1.28	***-1.38***
CBU_2092	pckA	phosphoenolpyruvate carboxykinase	1.09	1.15	-1.04	-1.23
CBU_2093	algH	transcriptional regulator	-1.18	-1.32	***-1.50***	***-1.82***
CBU_2094	-	Holliday junction resolvase-like protein	1.07	***-1.41***	-1.04	1.04
CBU_2095	pyrB	aspartate carbamoyltransferase	1.04	-1.57	-1.35	-1.55
CBU_2095a	-	hypothetical protein	-1.28	-1.37	-1.33	***-1.47***
CBUA0001	-	DNA-binding protein	-1.60	1.05	1.14	1.08
CBUA0003	-	hypothetical protein	-1.13	-1.16	-1.24	-1.21
CBUA0006	-	hypothetical protein	1.26	1.25	1.63	1.20
CBUA0007	-	hypothetical protein	1.45	1.23	***3.12***	***4.34***

NCBI RSA 493 mapping	NCBI syn	Description	5	7	14	21
CBUA0010	-	phage integrase family site specific recombinase	-1.38	-1.02	-1.13	-1.16
CBUA0011	-	hypothetical protein	-3.21	-2.77	-1.43	-1.74
CBUA0012	-	hypothetical protein	***-1.74***	1.13	***1.66***	***1.52***
CBUA0013	-	hypothetical protein	-1.34	-1.16	-1.03	-1.06
CBUA0014	-	hypothetical protein	-1.94	-1.47	-1.57	-1.61
CBUA0015	-	hypothetical protein	1.09	-1.12	-1.22	1.05
CBUA0016	-	CbhE protein	-2.26	-1.90	1.02	1.51
CBUA0017	-	hypothetical protein	1.42	1.17	-1.37	1.32
CBUA0020	-	hypothetical protein	-1.02	-1.12	1.16	1.17
CBUA0021	-	hypothetical protein	1.24	1.10	1.36	1.39
CBUA0022	-	hypothetical protein	1.01	-1.58	-1.28	-1.27
CBUA0023	-	hypothetical protein	-1.09	-1.10	-1.01	1.01
CBUA0024	-	hypothetical protein	-1.04	-1.01	1.05	-1.02
CBUA0025	-	hypothetical protein	1.19	1.13	1.25	***1.45***
CBUA0026	-	hypothetical protein	-1.21	-1.00	-1.32	***-1.64***
CBUA0027	-	DNA-binding protein	1.35	1.15	1.20	***1.42***
CBUA0028	-	hypothetical protein	***1.67***	1.29	***1.86***	***2.35***
CBUA0031	-	hypothetical protein	1.11	-1.09	***1.65***	***3.33***
CBUA0032	-	Ser/Thr protein phosphatase family protein	-1.27	-1.01	1.11	1.10
CBUA0033	-	hypothetical protein	1.03	-1.09	1.45	***2.03***
CBUA0034	-	hypothetical protein	1.01	-1.03	-1.11	-1.05
CBUA0036	-	parB protein, putative	***-1.42***	-1.04	-1.25	***-1.54***
CBUA0037	-	parA protein, putative	-1.02	1.38	***1.75***	***1.97***
CBUA0038	-	repB protein, putative	-1.08	1.29	1.22	***1.42***
CBUA0039	-	repA protein, putative	-1.07	1.16	***1.34***	1.28
CBUA0040	-	hypothetical protein	1.48	1.11	-1.02	-1.13
