# Supplementary material for: Transcriptional Profiling of Coxiella burnetii Reveals Extensive Cell Wall Remodeling in the Small Cell Variant Developmental Form
Source: PLoS One. 2016 Feb 24;11(2):e0149957. doi: 10.1371/journal.pone.0149957 (PMC4766238; doi:10.1371/journal.pone.0149957)
Supplement: S2 Table — (PDF) [file pone.0149957.s002.pdf]

S2 Table. Predicted amino acid transporter proteins in *Coxiella burnetii* RSA493

**ORF**	**Family ID**	**Family Name**	**Transporter Type**	**Substrate**	
CBU0922	MFS	The Major Facilitator Superfamily (MFS)	Secondary Transporter	valine	
CBU0566	MFS	The Major Facilitator Superfamily (MFS)	Secondary Transporter	asparagine	
CBU0515	MFS	The Major Facilitator Superfamily (MFS)	Secondary Transporter	valine	
CBU2058	MFS	The Major Facilitator Superfamily (MFS)	Secondary Transporter	proline/betaine	
CBU0539	POT	The Proton-dependent Oligopeptide Transporter (POT) Family	Secondary Transporter	proton/peptide	
CBU0504	POT	The Proton-dependent Oligopeptide Transporter (POT) Family	Secondary Transporter	proton/peptide	
CBU1347	APC	The Amino Acid-Polyamine-Organocation (APC) Family	Secondary Transporter	arginine	
CBU0953	APC	The Amino Acid-Polyamine-Organocation (APC) Family	Secondary Transporter	cationic amino acid	
CBU0570	APC	The Amino Acid-Polyamine-Organocation (APC) Family	Secondary Transporter	L-asparagine	
CBU0426	APC	The Amino Acid-Polyamine-Organocation (APC) Family	Secondary Transporter	amino acid	
CBU0354	APC	The Amino Acid-Polyamine-Organocation (APC) Family	Secondary Transporter	amino acid	
CBU2020	APC	The Amino Acid-Polyamine-Organocation (APC) Family	Secondary Transporter	L-glutamate/gamma-aminobutyrate	
CBU1798	APC	The Amino Acid-Polyamine-Organocation (APC) Family	Secondary Transporter	cationic amino acid	
CBU1796	APC	The Amino Acid-Polyamine-Organocation (APC) Family	Secondary Transporter	L-asparagine	
CBU1539	HAAAP	The Hydroxy/Aromatic Amino Acid Permease (HAAAP) Family	Secondary Transporter	tyrosine	
CBU1130	OPT	The Oligopeptide Transporter (OPT) Family	Secondary Transporter	oligopeptide	
					**Protein**
CBU0728	ABC	The ATP-binding Cassette (ABC) Superfamily	ATP-Dependent	amino acid	ABC
CBU0727	ABC	The ATP-binding Cassette (ABC) Superfamily	ATP-Dependent	amino acid	membrane
CBU0729	ABC	The ATP-binding Cassette (ABC) Superfamily	ATP-Dependent	amino acid	binding protein
CBU0481	ABC	The ATP-binding Cassette (ABC) Superfamily	ATP-Dependent	arginine	ABC
CBU0484	ABC	The ATP-binding Cassette (ABC) Superfamily	ATP-Dependent	arginine	membrane
CBU0483	ABC	The ATP-binding Cassette (ABC) Superfamily	ATP-Dependent	arginine	membrane
CBU0482	ABC	The ATP-binding Cassette (ABC) Superfamily	ATP-Dependent	arginine	binding protein
CBU0107	ABC	The ATP-binding Cassette (ABC) Superfamily	ATP-Dependent	amino acid	ABC
CBU0108	ABC	The ATP-binding Cassette (ABC) Superfamily	ATP-Dependent	amino acid	membrane
CBU0109	ABC	The ATP-binding Cassette (ABC) Superfamily	ATP-Dependent	amino acid	binding protein
CBU1857	ABC	The ATP-binding Cassette (ABC) Superfamily	ATP-Dependent	oligopeptide	ABC
CBU1859	ABC	The ATP-binding Cassette (ABC) Superfamily	ATP-Dependent	oligopeptide	membrane
CBU1858	ABC	The ATP-binding Cassette (ABC) Superfamily	ATP-Dependent	oligopeptide	membrane
CBU1860	ABC	The ATP-binding Cassette (ABC) Superfamily	ATP-Dependent	oligopeptide	binding protein

The substrate specificity of CBU0515 and CBU0922 is based on homology to PhtJ of *Legionella pneumophila* (67). The substrate specificity of CBU1347 is based on homology to ArgP of *Franscisella novicida* (68)
