# Supplementary material for: Transcriptional Profiling of Coxiella burnetii Reveals Extensive Cell Wall Remodeling in the Small Cell Variant Developmental Form
Source: PLoS One. 2016 Feb 24;11(2):e0149957. doi: 10.1371/journal.pone.0149957 (PMC4766238; doi:10.1371/journal.pone.0149957)
Supplement: S3 Table — (PDF) [file pone.0149957.s003.pdf]

S3 Table. Molecular weights of hypothetical proteins encoded by genes upregulated > 5-fold in 21 day SCV

Gene	Molecular weight (kDa)	Protein pI	NCBI RSA 493 mapping	Control=Vero-3 day signal	Test=Vero-21 day signal	Test=Vero-21 day signal / Control=Vero-3 day signal Unfiltered Fold
CBU_0957	47.6	9.75	CBU_0957	139	3,966	28.45
CBU_0536	4.4	4.85	CBU_0536	457	4,945	16.22
CBU_0019	3.8	12.51	CBU_0019	1,083	15,165	14.00
CBU_1681	10.7	10.41	CBU_1681	1,451	14,116	9.73
CBU_0193	47	10.19	CBU_0193	890	8,512	9.56
CBU_0711	5.4	10.41	CBU_0711	455	4,305	9.46
CBU_0538	3.2	12.02	CBU_0538	215	1,883	8.78
CBU_0956	25.4	10.61	CBU_0956	562	4,916	8.75
CBU_1932	7.5	12.28	CBU_1932	1,660	14,344	8.64
CBU_1331	14.1	5.72	CBU_1331	1,664	13,747	8.26
CBU_1332	5.7	8.48	CBU_1332	950	5,675	5.97
CBU_1295	12.6	9.71	CBU_1295	2,231	13,291	5.96
CBU_0181a	9.9	9.89	CBU_0181a	162	749	5.93
CBU_2079	14.3	8.62	CBU_2079	1,634	9,599	5.88
CBU_0477	10.6	11.50	CBU_0477	353	2,076	5.87
CBU_0877	4.6	11.21	CBU_0877	1,717	9,536	5.55
CBU_0469	7.4	4.53	CBU_0469	1,330	7,350	5.52
CBU_0961	6.3	8.66	CBU_0961	1,549	8,088	5.22
CBU_0555	6.6	4.02	CBU_0555	1,454	7,528	5.18
CBU_1561	29.6	10.03	CBU_1561	1,699	8,790	5.17
CBU_0731	17.7	9.62	CBU_0731	1,302	6,737	5.17
