# Supplementary material for: Transcriptional Profiling of Coxiella burnetii Reveals Extensive Cell Wall Remodeling in the Small Cell Variant Developmental Form
Source: PLoS One. 2016 Feb 24;11(2):e0149957. doi: 10.1371/journal.pone.0149957 (PMC4766238; doi:10.1371/journal.pone.0149957)
Supplement: S4 Table — (PDF) [file pone.0149957.s004.pdf]

Sequence Id	Sequence Name	Protein Length (AA)	Protein Weight (kDa)	Protein Isoelectric Point (pI)	D3 Normalized SC (N-SC)	D14 Normalized SC (N-SC)	D21 Normalized SC (N-SC)	D14 N-SC Log2 Relative Expression	D21 N-SC Log2 Relative Expression	Uniprot Accession
CBU_0002	Q83FD7_COXBU DNA polymerase III subunit beta OS=Coxiella burnetii (strain RSA 493 / Nine Mile phase I) GN=dnaN PE=3 SV=1	369	41.719256	0.0056685	498.26	134.71	30.88	-1.89	-4.01	Q83FD7
CBU_0004	Q83FD5_COXBU DNA gyrase subunit B OS=Coxiella burnetii (strain RSA 493 / Nine Mile phase I) GN=gyrB PE=3 SV=2	810	90.769981	0.0064937	31.21	7.31	12.86	-2.09	-1.28	Q83FD5
CBU_0007	Q83FD3_COXBU Uncharacterized protein OS=Coxiella burnetii (strain RSA 493 / Nine Mile phase I) GN=CBU_0007 PE=4 SV=1	70	8.4015218	0.0101694	116.07	105.67	97.68	-0.14	-0.25	Q83FD3
CBU_0010	AROE_COXBU Shikimate dehydrogenase OS=Coxiella burnetii (strain RSA 493 / Nine Mile phase I) GN=aroE PE=3 SV=1	272	29.757264	0.0062778	17.7	15.23	33.52	-0.22	0.92	Q83FD0
CBU_0016	Q83FC4_COXBU Purine nucleoside phosphorylase OS=Coxiella burnetii (strain RSA 493 / Nine Mile phase I) GN=xapA PE=3 SV=1	273	30.12275	0.0063921	81.57	111.63	124.04	0.45	0.6	Q83FC4
CBU_0017	Q83FC3_COXBU Deoxyribose-phosphate aldolase OS=Coxiella burnetii (strain RSA 493 / Nine Mile phase I) GN=deoC PE=3 SV=1	241	26.22381	0.0063667	63.68	105.58	117.54	0.73	0.88	Q83FC3
CBU_0020	Q83FC0_COXBU Ribosome-associated factor Y OS=Coxiella burnetii (strain RSA 493 / Nine Mile phase I) GN=CBU_0020 PE=4 SV=2	183	21.11086	0.0065073	0	324.98	252.66	10	10	Q83FC0
CBU_0021	Q83FB9_COXBU Uncharacterized protein OS=Coxiella burnetii (strain RSA 493 / Nine Mile phase I) GN=CBU_0021 PE=4 SV=1	809	93.004371	0.0083237	138.37	0	0	-10	-10	Q83FB9
CBU_0024	CSRA1_COXBU Carbon storage regulator homolog 1 OS=Coxiella burnetii (strain RSA 493 / Nine Mile phase I) GN=csrA1 PE=3 SV=1	68	7.6641091	0.0055669	159.31	0	0	-10	-10	Q83FB6
CBU_0026	RPIA_COXBU Ribose-5-phosphate isomerase A OS=Coxiella burnetii (strain RSA 493 / Nine Mile phase I) GN=rpiA PE=3 SV=1	220	23.81566	0.0060874	12.31	43.04	102.12	1.81	3.05	Q83FB4
CBU_0031	Q83FB1_COXBU 3-oxoacyl-[acyl-carrier protein] reductase OS=Coxiella burnetii (strain RSA 493 / Nine Mile phase I) GN=CBU_0031 PE=4 SV=1	312	33.812634	0.0067271	42.44	0	1.04	-10	-5.35	Q83FB1
CBU_0034	Q83FA8_COXBU Acyl carrier protein OS=Coxiella burnetii (strain RSA 493 / Nine Mile phase I) GN=acpP PE=3 SV=1	86	9.4799638	0.0040435	153.96	44.73	15.14	-1.78	-3.35	Q83FA8
CBU_0035	Q83FA7_COXBU 3-oxoacyl-[acyl-carrier-protein] synthase OS=Coxiella burnetii (strain RSA 493 / Nine Mile phase I) GN=CBU_0035 PE=3 SV=1	424	44.645181	0.0058081	24.84	4.19	9.98	-2.57	-1.32	Q83FA7
CBU_0036	Q820X6_COXBU (3R)-hydroxymyristoyl-[acyl carrier protein] dehydratase OS=Coxiella burnetii (strain RSA 493 / Nine Mile phase I) GN=CBU_0036 PE=4 SV=1	159	17.763367	0.006522	113.55	7.44	8.19	-3.93	-3.79	Q820X6
CBU_0037	Q820X5_COXBU 3-hydroxydecanoyl-[acyl-carrier-protein] dehydratase OS=Coxiella burnetii (strain RSA 493 / Nine Mile phase I) GN=fabA PE=4 SV=2	205	23.016863	0.0068296	24.95	0	0	-10	-10	Q820X5
CBU_0038	Q820X4_COXBU 3-oxoacyl-[acyl-carrier-protein] synthase III OS=Coxiella burnetii (strain RSA 493 / Nine Mile phase I) GN=CBU_0038 PE=3 SV=1	327	35.983959	0.0063159	129.75	0	0	-10	-10	Q820X4
CBU_0039	Q83FA6_COXBU Oligopeptidase A OS=Coxiella burnetii (strain RSA 493 / Nine Mile phase I) GN=prlC PE=3 SV=1	677	78.146567	0.0060112	369.36	304.18	230.38	-0.28	-0.68	Q83FA6
CBU_0043	NTPA_COXBU Non-canonical purine NTP pyrophosphatase OS=Coxiella burnetii (strain RSA 493 / Nine Mile phase I) GN=CBU_0043 PE=1 SV=1	200	21.745953	0.0045513	54.16	72.49	86.29	0.42	0.67	Q83FA3
CBU_0044	Q83FA2_COXBU Hypothetical exported protein OS=Coxiella burnetii (strain RSA 493 / Nine Mile phase I) GN=CBU_0044 PE=4 SV=2	251	27.993895	0.0108579	43.16	60.12	88.21	0.48	1.03	Q83FA2
CBU_0045	Q83FA1_COXBU Outer membrane protein OS=Coxiella burnetii (strain RSA 493 / Nine Mile phase I) GN=CBU_0045 PE=4 SV=1	186	21.119392	0.0112534	825.09	1073.74	1150.13	0.38	0.48	Q83FA1
CBU_0053	Q83F95_COXBU Enhanced entry protein OS=Coxiella burnetii (strain RSA 493 / Nine Mile phase I) GN=enhA.1 PE=4 SV=1	249	28.089279	0.0089536	0	19.01	30.08	10	10	Q83F95
CBU_0054	UBIC_COXBU Probable chorismate--pyruvate lyase OS=Coxiella burnetii (strain RSA 493 / Nine Mile phase I) GN=ubiC PE=3 SV=1	185	21.505096	0.0057192	112.23	76.77	110.88	-0.55	-0.02	Q83F94
CBU_0056	Q83F92_COXBU Type I secretion outer membrane protein OS=Coxiella burnetii (strain RSA 493 / Nine Mile phase I) GN=CBU_0056 PE=4 SV=1	616	68.616756	0.0099351	84.51	159.46	207.73	0.92	1.3	Q83F92
CBU_0064	Q83F84_COXBU Topoisomerase IV subunit B OS=Coxiella burnetii (strain RSA 493 / Nine Mile phase I) GN=parE PE=3 SV=2	641	71.49187	0.0069175	7.51	28.62	88.39	1.93	3.56	Q83F84
CBU_0065	Q83F83_COXBU Rhodanese-related sulfurtransferase OS=Coxiella burnetii (strain RSA 493 / Nine Mile phase I) GN=CBU_0065 PE=4 SV=1	124	14.087077	0.0053003	63.09	59.65	118.16	-0.08	0.91	Q83F83
CBU_0067	Q83F81_COXBU Uncharacterized protein OS=Coxiella burnetii (strain RSA 493 / Nine Mile phase I) GN=CBU_0067 PE=3 SV=1	107	12.508293	0.0052622	126.55	0	0	-10	-10	Q83F81
CBU_0073	Q83F75_COXBU Xaa-Pro aminopeptidase OS=Coxiella burnetii (strain RSA 493 / Nine Mile phase I) GN=CBU_0073 PE=3 SV=2	607	68.124853	0.0054907	101.13	104.8	111.04	0.05	0.13	Q83F75
CBU_0075	Q83F73_COXBU 2-polyprenyl-6-methoxyphenol hydroxylase OS=Coxiella burnetii (strain RSA 493 / Nine Mile phase I) GN=ubiH PE=4 SV=1	410	45.18533	0.0100083	1.47	12.99	28.59	3.15	4.28	Q83F73
CBU_0076	Q83F72_COXBU Monooxygenase OS=Coxiella burnetii (strain RSA 493 / Nine Mile phase I) GN=visC PE=4 SV=2	412	46.150196	0.009188	0	10.77	18.97	10	10	Q83F72
CBU_0081	SYP_COXBU Proline--tRNA ligase OS=Coxiella burnetii (strain RSA 493 / Nine Mile phase I) GN=proS PE=3 SV=1	566	63.530687	0.0056938	24.99	28.23	35.67	0.18	0.51	Q83F67
CBU_0084	Q83F64_COXBU Phosphoglycerol transferase MdoB and related protein-like protein, alkaline phosphatase superfamily OS=Coxiella burnetii (strain RSA 493 / Nine Mile phase I) GN=CBU_0084 PE=4 SV=1	638	72.692641	0.009437	26.88	11.59	15.31	-1.21	-0.81	Q83F64
CBU_0085	Q83F63_COXBU Entericidin A OS=Coxiella burnetii (strain RSA 493 / Nine Mile phase I) GN=CBU_0085 PE=4 SV=1	51	5.4659468	0.0103745	330.42	127.63	114.92	-1.37	-1.52	Q83F63
CBU_0089a	B5QS73_COXBU Uncharacterized protein OS=Coxiella burnetii (strain RSA 493 / Nine Mile phase I) GN=CBU_0089.1 PE=4 SV=1	113	12.460396	0.0102866	10.65	552.47	524.43	5.7	5.62	B5QS73
CBU_0090	TOLB_COXBU Protein TolB OS=Coxiella burnetii (strain RSA 493 / Nine Mile phase I) GN=tolB PE=1 SV=1	437	47.744686	0.0089829	37.18	10.16	9.69	-1.87	-1.94	Q83F59
CBU_0091	Q83F58_COXBU Peptidoglycan-associated lipoprotein OS=Coxiella burnetii (strain RSA 493 / Nine Mile phase I) GN=CBU_0091 PE=3 SV=2	195	21.325964	0.0098618	334.86	389.95	659.57	0.22	0.98	Q83F58
CBU_0092	Q83F57_COXBU Tol system periplasmic component OS=Coxiella burnetii (strain RSA 493 / Nine Mile phase I) GN=ybgF PE=4 SV=2	305	34.221545	0.0065513	56.24	35.89	40.57	-0.65	-0.47	Q83F57
CBU_0094	CLPB_COXBU Chaperone protein ClpB OS=Coxiella burnetii (strain RSA 493 / Nine Mile phase I) GN=clpB PE=3 SV=1	859	96.690883	0.0054146	165.7	334.8	314.24	1.01	0.92	Q83F55
CBU_0099	Q83F50_COXBU Phosphohistidine phosphatase SixA homolog OS=Coxiella burnetii (strain RSA 493 / Nine Mile phase I) GN=sixA PE=4 SV=2	159	18.077136	0.005605	43.53	72.57	81.91	0.74	0.91	Q83F50
CBU_0103	Q83F46_COXBU Peptidase, M20A family OS=Coxiella burnetii (strain RSA 493 / Nine Mile phase I) GN=CBU_0103 PE=4 SV=1	480	52.843935	0.0052368	23.82	33.9	30.53	0.51	0.36	Q83F46
CBU_0109	Q83F42_COXBU Lipoprotein OS=Coxiella burnetii (strain RSA 493 / Nine Mile phase I) GN=CBU_0109 PE=1 SV=1	267	28.858477	0.0097153	31.56	72.03	51.22	1.19	0.7	Q83F42
CBU_0110	Q83F41_COXBU Hypothetical exported protein OS=Coxiella burnetii (strain RSA 493 / Nine Mile phase I) GN=CBU_0110 PE=4 SV=2	161	18.264358	0.0093198	22.43	27.57	10.11	0.3	-1.15	Q83F41

CBU_0111	Q83F40_COXBU 2-amino-3-ketobutyrate coenzyme A ligase OS=Coxiella burnetii (strain RSA 493 / Nine Mile phase I) GN=kbl PE=1 SV=1	396	43.288307	0.0057954	42.55	88.91	116.76	1.06	1.46	Q83F40
CBU_0112	TDH_COXBU L-threonine 3-dehydrogenase OS=Coxiella burnetii (strain RSA 493 / Nine Mile phase I) GN=tdh PE=3 SV=1	342	37.152288	0.0077524	30.8	51.04	68.55	0.73	1.15	Q83F39
CBU_0114	Y114_COXBU UPF0234 protein CBU_0114 OS=Coxiella burnetii (strain RSA 493 / Nine Mile phase I) GN=CBU_0114 PE=3 SV=1	160	18.154593	0.0090562	122.25	173.83	134.31	0.51	0.14	Q83F37
CBU_0115	MRAZ_COXBU Protein MraZ OS=Coxiella burnetii (strain RSA 493 / Nine Mile phase I) GN=mraZ PE=3 SV=1	152	17.310101	0.0051733	21.78	245.26	246.35	3.49	3.5	Q83F36
CBU_0123	MURE_COXBU UDP-N-acetylmuramoyl-L-alanyl-D-glutamate--2,6-diaminopimelate ligase OS=Coxiella burnetii (strain RSA 493 / Nine Mile phase I) GN=murE PE=3 SV=1	489	53.775745	0.0064175	0	15.13	5.33	10	10	Q83F28
CBU_0124	Q83F27_COXBU UDP-N-acetylmuramoyl-tripeptide--D-alanyl-D-alanine ligase OS=Coxiella burnetii (strain RSA 493 / Nine Mile phase I) GN=murF PE=3 SV=1	446	47.64809	0.0070054	173.4	145.28	100.75	-0.26	-0.78	Q83F27
CBU_0131	MURD_COXBU UDP-N-acetylmuramoylalanine--D-glutamate ligase OS=Coxiella burnetii (strain RSA 493 / Nine Mile phase I) GN=murD PE=3 SV=1	442	48.724414	0.0068589	4.77	18.07	0.74	1.92	-2.69	Q83F20
CBU_0140	Q83F13_COXBU Cell division protein ftsA OS=Coxiella burnetii (strain RSA 493 / Nine Mile phase I) GN=ftsA PE=3 SV=2	413	44.626367	0.00577	16.76	1.43	0	-3.55	-10	Q83F13
CBU_0141	FTSZ_COXBU Cell division protein FtsZ OS=Coxiella burnetii (strain RSA 493 / Nine Mile phase I) GN=ftsZ PE=1 SV=1	386	40.702789	0.0044624	512.18	156.37	75.08	-1.71	-2.77	Q83F12
CBU_0147	SECA_COXBU Protein translocase subunit SecA OS=Coxiella burnetii (strain RSA 493 / Nine Mile phase I) GN=secA PE=3 SV=1	913	103.79638	0.0056304	151.94	119.26	126.61	-0.35	-0.26	Q83F06
CBU_0151	QUEF_COXBU NADPH-dependent 7-cyano-7-deazaguanine reductase OS=Coxiella burnetii (strain RSA 493 / Nine Mile phase I) GN=queF PE=3 SV=1	278	31.980291	0.0069614	10.82	0	0	-10	-10	Q83F02
CBU_0177	Q83EY1_COXBU Glycine betaine transport system permease protein OS=Coxiella burnetii (strain RSA 493 / Nine Mile phase I) GN=CBU_0177 PE=3 SV=2	526	58.263202	0.009979	2.86	21.37	5.57	2.9	0.96	Q83EY1
CBU_0178	Q83EY0_COXBU Glycine betaine transport ATP-binding protein OS=Coxiella burnetii (strain RSA 493 / Nine Mile phase I) GN=CBU_0178 PE=3 SV=1	252	28.545169	0.0094224	32.24	108.02	140.84	1.74	2.13	Q83EY0
CBU_0179	ANMK_COXBU Anhydro-N-acetylmuramic acid kinase OS=Coxiella burnetii (strain RSA 493 / Nine Mile phase I) GN=anmK PE=3 SV=1	372	40.634938	0.0068003	1.62	23.86	38.51	3.88	4.57	Q83EX9
CBU_0181	SYY_COXBU Tyrosine--tRNA ligase OS=Coxiella burnetii (strain RSA 493 / Nine Mile phase I) GN=tyrS PE=3 SV=1	399	45.783047	0.0062271	45.25	121.61	110.17	1.43	1.28	Q83EX7
CBU_0194	Q83EW4_COXBU L-serine dehydratase OS=Coxiella burnetii (strain RSA 493 / Nine Mile phase I) GN=sda PE=4 SV=1	455	48.840347	0.0052114	3.31	1.95	15.74	-0.76	2.25	Q83EW4
CBU_0199	COAA_COXBU Pantothenate kinase OS=Coxiella burnetii (strain RSA 493 / Nine Mile phase I) GN=coaA PE=1 SV=1	318	36.880505	0.0095249	10.41	38.15	45.05	1.87	2.11	Q83EV9
CBU_0205	SYE1_COXBU Glutamate--tRNA ligase 1 OS=Coxiella burnetii (strain RSA 493 / Nine Mile phase I) GN=gltX1 PE=3 SV=1	469	53.645949	0.0079722	1.28	1.26	13.89	-0.02	3.44	Q83EV3
CBU_0215	Q83EU4_COXBU Peptidase, C40 family OS=Coxiella burnetii (strain RSA 493 / Nine Mile phase I) GN=CBU_0215 PE=4 SV=2	532	60.104308	0.0061763	77.49	159.06	203.81	1.04	1.4	Q83EU4
CBU_0221.2	EFTU_COXBU Elongation factor Tu OS=Coxiella burnetii (strain RSA 493 / Nine Mile phase I) GN=tufA PE=1 SV=1	397	43.480485	0.0051987	3996.02	3900.03	3835.95	-0.04	-0.06	Q83ES6
CBU_0224	Q83ET6_COXBU Protein translocase subunit OS=Coxiella burnetii (strain RSA 493 / Nine Mile phase I) GN=secE PE=4 SV=1	127	14.205863	0.0108579	30.8	4.66	10.26	-2.72	-1.59	Q83ET6
CBU_0225	Q83ET5_COXBU Transcription termination/antitermination protein nusG OS=Coxiella burnetii (strain RSA 493 / Nine Mile phase I) GN=nusG PE=3 SV=2	188	21.66657	0.0089683	238.49	280.14	259.79	0.23	0.12	Q83ET5
CBU_0226	RL11_COXBU 50S ribosomal protein L11 OS=Coxiella burnetii (strain RSA 493 / Nine Mile phase I) GN=rplK PE=3 SV=1	145	15.495331	0.0101255	334.12	208.13	314.38	-0.68	-0.09	Q83ET4
CBU_0227	RL1_COXBU 50S ribosomal protein L1 OS=Coxiella burnetii (strain RSA 493 / Nine Mile phase I) GN=rplA PE=3 SV=1	232	24.691377	0.0100962	540.87	452.74	357.89	-0.26	-0.6	Q83ET3
CBU_0228	RL10_COXBU 50S ribosomal protein L10 OS=Coxiella burnetii (strain RSA 493 / Nine Mile phase I) GN=rplJ PE=3 SV=1	174	19.092366	0.0097153	883.72	840.01	668.06	-0.07	-0.4	Q83ET2
CBU_0229	RL7_COXBU 50S ribosomal protein L7/L12 OS=Coxiella burnetii (strain RSA 493 / Nine Mile phase I) GN=rplL PE=1 SV=1	126	13.221935	0.0045132	8346.78	3254.62	3178.56	-1.36	-1.39	P0C8S3
CBU_0231	RPOB_COXBU DNA-directed RNA polymerase subunit beta OS=Coxiella burnetii (strain RSA 493 / Nine Mile phase I) GN=rpoB PE=3 SV=1	1375	153.54318	0.0057573	823.74	837.49	765.36	0.02	-0.11	P0C8S4
CBU_0232	RPOC_COXBU DNA-directed RNA polymerase subunit beta' OS=Coxiella burnetii (strain RSA 493 / Nine Mile phase I) GN=rpoC PE=3 SV=1	1414	156.98796	0.0075474	882.1	786.35	732.5	-0.17	-0.27	Q83ET0
CBU_0233	RS12_COXBU 30S ribosomal protein S12 OS=Coxiella burnetii (strain RSA 493 / Nine Mile phase I) GN=rpsL PE=3 SV=1	124	13.726567	0.011854	303.34	214.75	160.18	-0.5	-0.92	Q83ES9
CBU_0234	RS7_COXBU 30S ribosomal protein S7 OS=Coxiella burnetii (strain RSA 493 / Nine Mile phase I) GN=rpsG PE=3 SV=3	191	21.259404	0.0107261	324.55	158.01	136.38	-1.04	-1.25	Q83ES8
CBU_0235	EFG_COXBU Elongation factor G OS=Coxiella burnetii (strain RSA 493 / Nine Mile phase I) GN=fusA PE=3 SV=1	699	77.666317	0.0049194	963.44	743.28	722.95	-0.37	-0.41	Q83ES7
CBU_0237	RS10_COXBU 30S ribosomal protein S10 OS=Coxiella burnetii (strain RSA 493 / Nine Mile phase I) GN=rpsJ PE=3 SV=1	110	12.565888	0.0100815	199.7	78	68.08	-1.36	-1.55	Q83ES5
CBU_0238	RL3_COXBU 50S ribosomal protein L3 OS=Coxiella burnetii (strain RSA 493 / Nine Mile phase I) GN=rplC PE=3 SV=1	217	23.475465	0.0102866	1110.75	839.9	897.3	-0.4	-0.31	Q83ES4
CBU_0239	RL4_COXBU 50S ribosomal protein L4 OS=Coxiella burnetii (strain RSA 493 / Nine Mile phase I) GN=rplD PE=3 SV=1	205	22.341932	0.0103892	1622	598.97	212.84	-1.44	-2.93	Q83ES3
CBU_0240	RL23_COXBU 50S ribosomal protein L23 OS=Coxiella burnetii (strain RSA 493 / Nine Mile phase I) GN=rplW PE=3 SV=1	95	10.784736	0.0103013	440.28	174.41	181.66	-1.34	-1.28	Q83ES2
CBU_0241	RL2_COXBU 50S ribosomal protein L2 OS=Coxiella burnetii (strain RSA 493 / Nine Mile phase I) GN=rplB PE=3 SV=1	275	30.37453	0.0116929	246.2	129.11	124.32	-0.93	-0.99	Q83ES1
CBU_0242	RS19_COXBU 30S ribosomal protein S19 OS=Coxiella burnetii (strain RSA 493 / Nine Mile phase I) GN=rpsS PE=3 SV=1	95	10.79267	0.0109165	95.03	40.49	34.27	-1.23	-1.47	Q83ES0
CBU_0243	RL22_COXBU 50S ribosomal protein L22 OS=Coxiella burnetii (strain RSA 493 / Nine Mile phase I) GN=rplV PE=3 SV=1	115	12.556668	0.0102427	510.25	367.91	328.44	-0.47	-0.64	O85387
CBU_0244	RS3_COXBU 30S ribosomal protein S3 OS=Coxiella burnetii (strain RSA 493 / Nine Mile phase I) GN=rpsC PE=3 SV=2	227	25.592835	0.0103892	616.41	371.47	331.35	-0.73	-0.9	O85388
CBU_0245	RL16_COXBU 50S ribosomal protein L16 OS=Coxiella burnetii (strain RSA 493 / Nine Mile phase I) GN=rplP PE=3 SV=1	137	15.47153	0.0116196	232.82	203.01	223.41	-0.2	-0.06	Q83ER9
CBU_0246	RL29_COXBU 50S ribosomal protein L29 OS=Coxiella burnetii (strain RSA 493 / Nine Mile phase I) GN=rpmC PE=3 SV=1	65	7.676238	0.0107847	263.88	4.55	0	-5.86	-10	Q83ER8
CBU_0247	RS17_COXBU 30S ribosomal protein S17 OS=Coxiella burnetii (strain RSA 493 / Nine Mile phase I) GN=rpsQ PE=3 SV=1	89	10.27954	0.0107261	284.01	132.98	120.73	-1.09	-1.23	Q83ER7
CBU_0248	RL14_COXBU 50S ribosomal protein L14 OS=Coxiella burnetii (strain RSA 493 / Nine Mile phase I) GN=rplN PE=3 SV=1	122	13.513559	0.0114438	670.89	417.13	424.36	-0.69	-0.66	Q83ER6
CBU_0249	RL24_COXBU 50S ribosomal protein L24 OS=Coxiella burnetii (strain RSA 493 / Nine Mile phase I) GN=rplX PE=3 SV=1	107	11.765674	0.0105356	534.33	210.15	164.33	-1.35	-1.7	Q83ER5
CBU_0250	RL5_COXBU 50S ribosomal protein L5 OS=Coxiella burnetii (strain RSA 493 / Nine Mile phase I) GN=rplE PE=3 SV=1	182	20.717127	0.0101694	388.54	277.99	218.27	-0.48	-0.83	Q83ER4
CBU_0251	Q83ER3_COXBU 30S ribosomal protein S14 OS=Coxiella burnetii (strain RSA 493 / Nine Mile phase I) GN=rpsN PE=3 SV=1	99	11.54247	0.0115171	231	92.65	121.69	-1.32	-0.92	Q83ER3
CBU_0252	RS8_COXBU 30S ribosomal protein S8 OS=Coxiella burnetii (strain RSA 493 / Nine Mile phase I) GN=rpsH PE=3 SV=1	130	14.541725	0.0097593	481.46	216.22	172.82	-1.15	-1.48	Q83ER2
CBU_0253	RL6_COXBU 50S ribosomal protein L6 OS=Coxiella burnetii (strain RSA 493 / Nine Mile phase I) GN=rplF PE=3 SV=1	178	19.166523	0.0105649	338.11	202.79	219.51	-0.74	-0.62	Q83ER1

CBU_0254	RL18_COXBU 50S ribosomal protein L18 OS=Coxiella burnetii (strain RSA 493 / Nine Mile phase I) GN=rplR PE=3 SV=1	117	13.080266	0.0112974	195.47	73.34	108.54	-1.41	-0.85	Q83ER0
CBU_0255	RS5_COXBU 30S ribosomal protein S5 OS=Coxiella burnetii (strain RSA 493 / Nine Mile phase I) GN=rpsE PE=3 SV=1	168	17.457288	0.0102866	480.03	274.74	255.84	-0.81	-0.91	Q83EQ9
CBU_0256	RL30_COXBU 50S ribosomal protein L30 OS=Coxiella burnetii (strain RSA 493 / Nine Mile phase I) GN=rpmD PE=3 SV=1	63	7.2758447	0.00973	315.24	23.48	15.51	-3.75	-4.35	Q83EQ8
CBU_0257	RL15_COXBU 50S ribosomal protein L15 OS=Coxiella burnetii (strain RSA 493 / Nine Mile phase I) GN=rplO PE=3 SV=1	143	15.257589	0.0117515	288.29	215.18	179.88	-0.42	-0.68	Q83EQ7
CBU_0258	Q83EQ6_COXBU Protein translocase subunit SecY OS=Coxiella burnetii (strain RSA 493 / Nine Mile phase I) GN=secY PE=3 SV=1	442	48.643413	0.0106235	225.35	272.45	302.77	0.27	0.43	Q83EQ6
CBU_0260	RS13_COXBU 30S ribosomal protein S13 OS=Coxiella burnetii (strain RSA 493 / Nine Mile phase I) GN=rpsM PE=3 SV=1	119	13.363346	0.0113999	515.85	186.48	194.27	-1.47	-1.41	P59753
CBU_0261	RS11_COXBU 30S ribosomal protein S11 OS=Coxiella burnetii (strain RSA 493 / Nine Mile phase I) GN=rpsK PE=3 SV=1	123	13.479109	0.0117515	611.61	245.36	248.84	-1.32	-1.3	Q83EQ4
CBU_0262	RS4_COXBU 30S ribosomal protein S4 OS=Coxiella burnetii (strain RSA 493 / Nine Mile phase I) GN=rpsD PE=3 SV=1	206	23.696674	0.0103159	670.48	509.88	406.22	-0.4	-0.72	Q83EQ3
CBU_0263	RPOA_COXBU DNA-directed RNA polymerase subunit alpha OS=Coxiella burnetii (strain RSA 493 / Nine Mile phase I) GN=rpoA PE=3 SV=1	327	35.515806	0.0055288	1671.13	1072.21	959.9	-0.64	-0.8	Q83EQ2
CBU_0264	RL17_COXBU 50S ribosomal protein L17 OS=Coxiella burnetii (strain RSA 493 / Nine Mile phase I) GN=rplQ PE=3 SV=1	126	14.515736	0.0105796	35.82	65.75	67.19	0.88	0.91	Q83EQ1
CBU_0270	Y270_COXBU Putative reductase CBU_0270 OS=Coxiella burnetii (strain RSA 493 / Nine Mile phase I) GN=CBU_0270 PE=3 SV=1	406	44.829054	0.0057319	178.62	277.66	220.55	0.64	0.3	Q83EP5
CBU_0271	SSB_COXBU Single-stranded DNA-binding protein OS=Coxiella burnetii (strain RSA 493 / Nine Mile phase I) GN=ssb PE=1 SV=1	158	17.408522	0.0055415	714.19	245.31	241.12	-1.54	-1.57	Q83EP4
CBU_0272	Q83EP3_COXBU Transporter, MFS superfamily OS=Coxiella burnetii (strain RSA 493 / Nine Mile phase I) GN=CBU_0272 PE=4 SV=1	458	50.72247	0.0098179	99.21	158.92	86.73	0.68	-0.19	Q83EP3
CBU_0274	Q83EP1_COXBU UvrABC system protein A OS=Coxiella burnetii (strain RSA 493 / Nine Mile phase I) GN=uvrA PE=3 SV=1	954	106.05351	0.0087046	10.09	48.69	63.48	2.27	2.65	Q83EP1
CBU_0275	DCUP_COXBU Uroporphyrinogen decarboxylase OS=Coxiella burnetii (strain RSA 493 / Nine Mile phase I) GN=hemE PE=3 SV=1	361	40.160072	0.0090562	22.51	2.46	13.53	-3.19	-0.73	Q83EP0
CBU_0286	Q83EM9_COXBU Poly(A) polymerase OS=Coxiella burnetii (strain RSA 493 / Nine Mile phase I) GN=pcnB PE=3 SV=1	439	52.397894	0.0105649	5.48	4.04	6.68	-0.44	0.28	Q83EM9
CBU_0288	COAD_COXBU Phosphopantetheine adenylyltransferase OS=Coxiella burnetii (strain RSA 493 / Nine Mile phase I) GN=coaD PE=1 SV=1	159	17.937541	0.0062397	355.8	695.96	579.54	0.97	0.7	Q83EM7
CBU_0291	RL28_COXBU 50S ribosomal protein L28 OS=Coxiella burnetii (strain RSA 493 / Nine Mile phase I) GN=rpmB PE=3 SV=1	79	9.2201303	0.0118247	232.35	56.18	41.22	-2.05	-2.5	Q83EM4
CBU_0293	DUT_COXBU Deoxyuridine 5'-triphosphate nucleotidohydrolase OS=Coxiella burnetii (strain RSA 493 / Nine Mile phase I) GN=dut PE=1 SV=1	152	16.1854	0.0054272	132.64	0	0	-10	-10	Q45920
CBU_0296	PYRE_COXBU Orotate phosphoribosyltransferase OS=Coxiella burnetii (strain RSA 493 / Nine Mile phase I) GN=pyrE PE=3 SV=1	209	23.43226	0.0067563	162.7	256.24	300.68	0.66	0.89	Q45918
CBU_0297	Q83EM1_COXBU Exodeoxyribonuclease III OS=Coxiella burnetii (strain RSA 493 / Nine Mile phase I) GN=xth PE=4 SV=1	259	30.415343	0.0093052	26.72	39.98	37.72	0.58	0.5	Q83EM1
CBU_0299	RNPH_COXBU Ribonuclease PH OS=Coxiella burnetii (strain RSA 493 / Nine Mile phase I) GN=rph PE=3 SV=1	237	25.936477	0.0079722	72.37	67.41	81.06	-0.1	0.16	Q83EL9
CBU_0300	Q83EL8_COXBU Stress-induced protein, putative OS=Coxiella burnetii (strain RSA 493 / Nine Mile phase I) GN=yicC PE=4 SV=1	287	32.99356	0.0059224	0	6.19	14.75	10	10	Q83EL8
CBU_0301	KGUA_COXBU Guanylate kinase OS=Coxiella burnetii (strain RSA 493 / Nine Mile phase I) GN=gmk PE=1 SV=1	206	23.776717	0.007855	77.42	51.71	82.19	-0.58	0.09	Q83EL7
CBU_0302	RPOZ_COXBU DNA-directed RNA polymerase subunit omega OS=Coxiella burnetii (strain RSA 493 / Nine Mile phase I) GN=rpoZ PE=3 SV=1	97	10.734434	0.0045767	322.63	189.12	161.13	-0.77	-1	Q83EL6
CBU_0303	Q820X2_COXBU GTP pyrophosphokinase OS=Coxiella burnetii (strain RSA 493 / Nine Mile phase I) GN=spoT PE=3 SV=1	707	80.053957	0.0099058	18.3	69.05	96.25	1.92	2.39	Q820X2
CBU_0304	Q83EL5_COXBU Translation initiation inhibitor OS=Coxiella burnetii (strain RSA 493 / Nine Mile phase I) GN=CBU_0304 PE=4 SV=2	148	16.391616	0.0089976	677.06	261.89	195.81	-1.37	-1.79	Q83EL5
CBU_0307	Q83EL2_COXBU Outer membrane protein OS=Coxiella burnetii (strain RSA 493 / Nine Mile phase I) GN=CBU_0307 PE=4 SV=1	231	24.867705	0.0101694	1758.59	262.57	638.53	-2.74	-1.46	Q83EL2
CBU_0309	HTPG_COXBU Chaperone protein HtpG OS=Coxiella burnetii (strain RSA 493 / Nine Mile phase I) GN=htpG PE=1 SV=1	633	72.710706	0.0050718	832.39	288.86	268.51	-1.53	-1.63	Q83EL0
CBU_0312	FOLD_COXBU Bifunctional protein FolD OS=Coxiella burnetii (strain RSA 493 / Nine Mile phase I) GN=folD PE=3 SV=1	283	30.782392	0.0082651	11.7	12.55	17.26	0.1	0.56	Q83EK7
CBU_0314	Q83EK5_COXBU Hydroxyacylglutathione hydrolase OS=Coxiella burnetii (strain RSA 493 / Nine Mile phase I) GN=gloB PE=3 SV=1	257	28.887684	0.0061001	0	4.61	2.53	10	10	Q83EK5
CBU_0326	Q83EJ4_COXBU Phosphoribosylamine--glycine ligase OS=Coxiella burnetii (strain RSA 493 / Nine Mile phase I) GN=purD PE=3 SV=2	437	47.582687	0.0060874	229.3	356.13	284.63	0.64	0.31	Q83EJ4
CBU_0336	Q83EI4_COXBU Bifunctional purine biosynthesis protein PurH OS=Coxiella burnetii (strain RSA 493 / Nine Mile phase I) GN=purH PE=3 SV=2	527	57.542003	0.0065073	7.42	23.58	16.06	1.67	1.11	Q83EI4
CBU_0337	Q83EI3_COXBU DNA-binding protein fis OS=Coxiella burnetii (strain RSA 493 / Nine Mile phase I) GN=fis PE=3 SV=1	103	11.568019	0.0072251	461.6	0	0	-10	-10	Q83EI3
CBU_0338	Q83EI2_COXBU Membrane alanine aminopeptidase OS=Coxiella burnetii (strain RSA 493 / Nine Mile phase I) GN=pepN PE=4 SV=2	901	102.9401	0.0060366	166.32	194.08	225.5	0.22	0.44	Q83EI2
CBU_0341	K6PF_COXBU 6-phosphofructokinase OS=Coxiella burnetii (strain RSA 493 / Nine Mile phase I) GN=pfkA PE=3 SV=1	326	34.972253	0.0091147	1.85	2.72	10.99	0.56	2.57	Q83EH9
CBU_0347	Q83EH4_COXBU D-xylose-proton symporter OS=Coxiella burnetii (strain RSA 493 / Nine Mile phase I) GN=CBU_0347 PE=3 SV=2	463	51.111588	0.0097593	16.9	37.06	30.94	1.13	0.87	Q83EH4
CBU_0350	UBIG_COXBU 3-demethylubiquinone-9 3-methyltransferase OS=Coxiella burnetii (strain RSA 493 / Nine Mile phase I) GN=ubiG PE=3 SV=1	234	26.450515	0.0058208	59.15	15.17	27.83	-1.96	-1.09	Q820B5
CBU_0353	Q83EG9_COXBU Non-proteolytic protein, peptidase family S49 OS=Coxiella burnetii (strain RSA 493 / Nine Mile phase I) GN=CBU_0353 PE=4 SV=1	338	38.319648	0.0098911	30.27	172.45	170.51	2.51	2.49	Q83EG9
CBU_0364	Q83EF9_COXBU Phosphate transporter OS=Coxiella burnetii (strain RSA 493 / Nine Mile phase I) GN=CBU_0364 PE=4 SV=2	443	47.797889	0.0100229	36	0	0	-10	-10	Q83EF9
CBU_0366	Q83EF7_COXBU Phosphate regulon sensor protein OS=Coxiella burnetii (strain RSA 493 / Nine Mile phase I) GN=phoR PE=4 SV=1	252	28.891306	0.0094224	7.16	30.53	21.97	2.09	1.62	Q83EF7
CBU_0367	Q83EF6_COXBU Phosphate regulon transcriptional regulatory protein OS=Coxiella burnetii (strain RSA 493 / Nine Mile phase I) GN=phoB PE=4 SV=1	248	28.803394	0.0078403	3.64	3.58	11.82	-0.02	1.7	Q83EF6
CBU_0368	Q83EF5_COXBU Hypothetical exported protein OS=Coxiella burnetii (strain RSA 493 / Nine Mile phase I) GN=CBU_0368 PE=4 SV=2	394	44.025397	0.0098911	9.16	13.52	3.31	0.56	-1.47	Q83EF5
CBU_0370	Q83EF3_COXBU Hypothetical membrane associated protein OS=Coxiella burnetii (strain RSA 493 / Nine Mile phase I) GN=CBU_0370 PE=4 SV=1	75	7.3429303	0.0113853	36.11	741.66	464.53	4.36	3.69	Q83EF3
CBU_0378	Q83EE6_COXBU Hypothetical membrane associated protein OS=Coxiella burnetii (strain RSA 493 / Nine Mile phase I) GN=CBU_0378 PE=4 SV=1	140	15.032989	0.0103892	15.05	0	0	-10	-10	Q83EE6

CBU_0379	Q83EE5_COXBU Anhydro-N-acetylmuramyl-tripeptide amidase OS=Coxiella burnetii (strain RSA 493 / Nine Mile phase I) GN=ampD PE=4 SV=1	257	30.116332	0.0060112	50.35	137	177.37	1.44	1.82	Q83EE5
CBU_0385	RL21_COXBU 50S ribosomal protein L21 OS=Coxiella burnetii (strain RSA 493 / Nine Mile phase I) GN=rplU PE=3 SV=2	115	12.733863	0.0101694	368.95	131.21	127.41	-1.49	-1.53	Q83EE0
CBU_0386	RL27_COXBU 50S ribosomal protein L27 OS=Coxiella burnetii (strain RSA 493 / Nine Mile phase I) GN=rpmA PE=3 SV=1	90	10.023287	0.0112388	407.91	220.26	264.1	-0.89	-0.63	Q83ED9
CBU_0387	OBG_COXBU GTPase obg OS=Coxiella burnetii (strain RSA 493 / Nine Mile phase I) GN=obg PE=3 SV=1	339	37.125463	0.0078257	24.85	49.75	47.06	1	0.92	Q83ED8
CBU_0391	Q83ED4_COXBU Riboflavin kinase OS=Coxiella burnetii (strain RSA 493 / Nine Mile phase I) GN=ribF PE=4 SV=2	345	39.09668	0.009481	0	1.72	9.44	10	10	Q83ED4
CBU_0395	Q83ED0_COXBU Hypothetical exported protein OS=Coxiella burnetii (strain RSA 493 / Nine Mile phase I) GN=CBU_0395 PE=4 SV=1	228	24.461187	0.0093931	147.82	1036.86	811.17	2.81	2.46	Q83ED0
CBU_0396	SYI_COXBU Isoleucine--tRNA ligase OS=Coxiella burnetii (strain RSA 493 / Nine Mile phase I) GN=ileS PE=3 SV=1	936	106.08343	0.005897	52.72	86.61	94.27	0.72	0.84	Q83EC9
CBU_0418	Q83EA8_COXBU Branched-chain amino acid aminotransferase OS=Coxiella burnetii (strain RSA 493 / Nine Mile phase I) GN=CBU_0418 PE=3 SV=1	281	31.660161	0.0089536	1.07	9.48	16.22	3.15	3.92	Q83EA8
CBU_0419	Q83EA7_COXBU Polysaccharide deacetylase family OS=Coxiella burnetii (strain RSA 493 / Nine Mile phase I) GN=CBU_0419 PE=4 SV=1	276	32.540144	0.0099937	0	57.89	70.78	10	10	Q83EA7
CBU_0424	PANB_COXBU 3-methyl-2-oxobutanoate hydroxymethyltransferase OS=Coxiella burnetii (strain RSA 493 / Nine Mile phase I) GN=panB PE=3 SV=1	266	28.95973	0.0072104	19.23	0	0	-10	-10	Q83EA2
CBU_0430	SYK_COXBU Lysine--tRNA ligase OS=Coxiella burnetii (strain RSA 493 / Nine Mile phase I) GN=lysS PE=3 SV=1	498	57.684513	0.0060112	61.03	33.87	66.04	-0.85	0.11	Q83E97
CBU_0431	Q83E96_COXBU Aminomethyltransferase family protein OS=Coxiella burnetii (strain RSA 493 / Nine Mile phase I) GN=CBU_0431 PE=3 SV=1	258	28.825988	0.0070933	18.66	49.31	49.22	1.4	1.4	Q83E96
CBU_0433	Q83E94_COXBU Mechanosensitive ion channel OS=Coxiella burnetii (strain RSA 493 / Nine Mile phase I) GN=CBU_0433 PE=4 SV=1	173	19.19574	0.010521	34.79	73.54	22.59	1.08	-0.62	Q83E94
CBU_0442	RL19_COXBU 50S ribosomal protein L19 OS=Coxiella burnetii (strain RSA 493 / Nine Mile phase I) GN=rplS PE=3 SV=1	115	13.249332	0.0112388	1418.22	666.36	792.79	-1.09	-0.84	Q83E85
CBU_0445	RS16_COXBU 30S ribosomal protein S16 OS=Coxiella burnetii (strain RSA 493 / Nine Mile phase I) GN=rpsP PE=3 SV=1	137	15.532289	0.0103599	560.1	233.24	206.77	-1.26	-1.44	Q83E83
CBU_0450	Q83E79_COXBU Signal recognition particle protein OS=Coxiella burnetii (strain RSA 493 / Nine Mile phase I) GN=ffh PE=3 SV=1	461	50.892324	0.0102134	3.26	1.28	0	-1.35	-10	Q83E79
CBU_0454	KAD_COXBU Adenylate kinase OS=Coxiella burnetii (strain RSA 493 / Nine Mile phase I) GN=adk PE=3 SV=1	231	25.83158	0.0063667	49.5	87.1	93.03	0.82	0.91	Q83E75
CBU_0455	Q83E74_COXBU Thioredoxin OS=Coxiella burnetii (strain RSA 493 / Nine Mile phase I) GN=CBU_0455 PE=3 SV=1	121	13.910229	0.0047163	17.41	44.01	29.6	1.34	0.77	Q83E74
CBU_0456	HQ1_COXBU Histone-like protein Hq1 OS=Coxiella burnetii (strain RSA 493 / Nine Mile phase I) GN=hcbA PE=1 SV=1	117	13.171939	0.0130845	72.01	1244.19	1338.61	4.11	4.22	Q45881
CBU_0461	Q83E69_COXBU Pyruvate dehydrogenase E1 component OS=Coxiella burnetii (strain RSA 493 / Nine Mile phase I) GN=pdhA PE=3 SV=1	884	99.696954	0.0058462	291.04	237.97	225.05	-0.29	-0.37	Q83E69
CBU_0462	Q83E68_COXBU Dihydrolipoamide acetyltransferase component of pyruvate dehydrogenase complex OS=Coxiella burnetii (strain RSA 493 / Nine Mile phase I) GN=pdhC PE=3 SV=1	436	47.925436	0.0058716	194.63	158.8	174.01	-0.29	-0.16	Q83E68
CBU_0463	Q83E67_COXBU Dihydrolipoyl dehydrogenase OS=Coxiella burnetii (strain RSA 493 / Nine Mile phase I) GN=lpdA PE=3 SV=1	474	51.134068	0.0072837	523.74	518.09	509.02	-0.02	-0.04	Q83E67
CBU_0467	BIOC1_COXBU Malonyl-[acyl-carrier protein] O-methyltransferase 1 OS=Coxiella burnetii (strain RSA 493 / Nine Mile phase I) GN=bioC1 PE=3 SV=1	282	31.671139	0.0063413	8.54	0	0	-10	-10	Q83E64
CBU_0469	Q83E62_COXBU Uncharacterized protein OS=Coxiella burnetii (strain RSA 493 / Nine Mile phase I) GN=CBU_0469 PE=4 SV=1	67	7.3407163	0.0046401	0	145.73	72.9	10	10	Q83E62
CBU_0470	Q83E61_COXBU Purine/pyrimidine phosphoribosyl transferase OS=Coxiella burnetii (strain RSA 493 / Nine Mile phase I) GN=CBU_0470 PE=4 SV=1	218	23.963897	0.0081772	22.09	297.23	262.88	3.75	3.57	Q83E61
CBU_0473	Q83E58_COXBU Bacterial DNA-binding protein OS=Coxiella burnetii (strain RSA 493 / Nine Mile phase I) GN=CBU_0473 PE=4 SV=2	129	14.278013	0.0112681	1856.8	472.48	532.58	-1.97	-1.8	Q83E58
CBU_0479	KDSB_COXBU 3-deoxy-manno-octulosonate cytidylyltransferase OS=Coxiella burnetii (strain RSA 493 / Nine Mile phase I) GN=kdsB PE=1 SV=3	249	27.989052	0.0048813	164.35	152.1	197.46	-0.11	0.26	Q83E52
CBU_0481	Q83E50_COXBU Arginine transport ATP-binding protein OS=Coxiella burnetii (strain RSA 493 / Nine Mile phase I) GN=artP PE=3 SV=1	250	27.776587	0.0084409	18.05	0	0	-10	-10	Q83E50
CBU_0482	Q83E49_COXBU Arginine-binding protein OS=Coxiella burnetii (strain RSA 493 / Nine Mile phase I) GN=CBU_0482 PE=1 SV=1	269	29.620295	0.0097153	7.83	15.4	12.1	0.98	0.63	Q83E49
CBU_0486	Q83E45_COXBU Ribonuclease E OS=Coxiella burnetii (strain RSA 493 / Nine Mile phase I) GN=CBU_0486 PE=4 SV=1	720	80.331074	0.0096274	465.58	424.5	481.63	-0.13	0.05	Q83E45
CBU_0488	Q83E44_COXBU Bis(5'-nucleosyl)-tetraphosphatase (Symmetrical) OS=Coxiella burnetii (strain RSA 493 / Nine Mile phase I) GN=CBU_0488 PE=4 SV=1	235	26.783908	0.0062778	0	25.18	54.04	10	10	Q83E44
CBU_0491	RL32_COXBU 50S ribosomal protein L32 OS=Coxiella burnetii (strain RSA 493 / Nine Mile phase I) GN=rpmF PE=3 SV=1	64	7.4457661	0.0103745	37.61	0	0	-10	-10	Q83E41
CBU_0492	PLSX_COXBU Phosphate acyltransferase OS=Coxiella burnetii (strain RSA 493 / Nine Mile phase I) GN=plsX PE=3 SV=1	343	37.409156	0.0093784	297.4	469.26	349.34	0.66	0.23	Q83E40
CBU_0493	FABH_COXBU 3-oxoacyl-[acyl-carrier-protein] synthase 3 OS=Coxiella burnetii (strain RSA 493 / Nine Mile phase I) GN=fabH PE=3 SV=1	319	34.705249	0.0058462	532.97	446.13	467.49	-0.26	-0.19	Q820X0
CBU_0494	Q83E39_COXBU Malonyl CoA-acyl carrier protein transacylase OS=Coxiella burnetii (strain RSA 493 / Nine Mile phase I) GN=fabD PE=1 SV=1	313	33.948716	0.0054653	315.33	404.58	390.11	0.36	0.31	Q83E39
CBU_0495	Q820W9_COXBU 3-oxoacyl-[acyl-carrier protein] reductase OS=Coxiella burnetii (strain RSA 493 / Nine Mile phase I) GN=fabG PE=3 SV=1	249	26.280453	0.007811	343.21	356.48	303.38	0.05	-0.18	Q820W9
CBU_0496	Q83E38_COXBU Acyl carrier protein OS=Coxiella burnetii (strain RSA 493 / Nine Mile phase I) GN=acpP PE=3 SV=1	84	9.2724648	0.0038022	257.93	341.66	360.5	0.41	0.48	Q83E38
CBU_0497	FABF_COXBU 3-oxoacyl-[acyl-carrier-protein] synthase 2 OS=Coxiella burnetii (strain RSA 493 / Nine Mile phase I) GN=fabF PE=1 SV=1	414	44.029147	0.0053892	157.73	274.43	261.9	0.8	0.73	Q83E37
CBU_0499	KTHY_COXBU Thymidylate kinase OS=Coxiella burnetii (strain RSA 493 / Nine Mile phase I) GN=tmk PE=3 SV=2	227	25.12619	0.006522	26.51	16.94	38.73	-0.65	0.55	Q83E35
CBU_0502	Q83E32_COXBU DNase, TatD family OS=Coxiella burnetii (strain RSA 493 / Nine Mile phase I) GN=CBU_0502 PE=4 SV=2	255	28.59049	0.0059351	24.78	38.29	43.41	0.63	0.81	Q83E32
CBU_0503	Q83E31_COXBU Glutamine synthetase OS=Coxiella burnetii (strain RSA 493 / Nine Mile phase I) GN=glnA PE=3 SV=1	359	39.572678	0.0052241	508.79	129.39	104.3	-1.98	-2.29	Q83E31
CBU_0516a	B5QS96_COXBU Uncharacterized protein OS=Coxiella burnetii (strain RSA 493 / Nine Mile phase I) GN=CBU_0516.1 PE=4 SV=1	118	12.841227	0.0083677	5.1	5.01	19.32	-0.02	1.92	B5QS96
CBU_0517	Q83E19_COXBU Aspartate aminotransferase OS=Coxiella burnetii (strain RSA 493 / Nine Mile phase I) GN=aspB PE=3 SV=2	423	46.372028	0.0064048	336.48	265.1	227.08	-0.34	-0.57	Q83E19
CBU_0518	UVRB_COXBU UvrABC system protein B OS=Coxiella burnetii (strain RSA 493 / Nine Mile phase I) GN=uvrB PE=3 SV=1	672	77.270354	0.0054399	8.96	24.66	44.09	1.46	2.3	Q83E18

CBU_0520	Q83E16_COXBU Hydroxymethylglutaryl-CoA lyase OS=Coxiella burnetii (strain RSA 493 / Nine Mile phase I) GN=leuA PE=4 SV=2		299	32.305791	0.0063413	1.01	10.89	19.6	3.44	4.28	Q83E16
CBU_0521	Q83E15_COXBU 5-methylthioadenosine/S-adenosylhomocysteine deaminase OS=Coxiella burnetii (strain RSA 493 / Nine Mile phase I) GN=mtaD PE=3 SV=2		484	53.230844	0.0058208	129.32	24.45	14.8	-2.4	-3.13	Q83E15
CBU_0524	Q83E13_COXBU DNA gyrase subunit A OS=Coxiella burnetii (strain RSA 493 / Nine Mile phase I) GN=gyrA PE=3 SV=2		850	94.176145	0.0088218	121.07	33.76	13.41	-1.84	-3.17	Q83E13
CBU_0525	SERC_COXBU Phosphoserine aminotransferase OS=Coxiella burnetii (strain RSA 493 / Nine Mile phase I) GN=serC PE=3 SV=1		360	40.645562	0.0057319	213.15	136.43	149.24	-0.64	-0.51	Q83E12
CBU_0526	AROA_COXBU 3-phosphoshikimate 1-carboxyvinyltransferase OS=Coxiella burnetii (strain RSA 493 / Nine Mile phase I) GN=aroA PE=1 SV=1		438	46.384201	0.0062778	218.47	316.82	362.78	0.54	0.73	Q83E11
CBU_0527	KCY_COXBU Cytidylate kinase OS=Coxiella burnetii (strain RSA 493 / Nine Mile phase I) GN=cmk PE=3 SV=1		237	26.424885	0.0093491	74.91	77.4	103.04	0.05	0.46	Q83E10
CBU_0528	RS1_COXBU 30S ribosomal protein S1 OS=Coxiella burnetii (strain RSA 493 / Nine Mile phase I) GN=rpsA PE=1 SV=1		551	62.064283	0.0051353	705.59	257.21	244.65	-1.46	-1.53	Q83E09
CBU_0531	PYRF_COXBU Orotidine 5'-phosphate decarboxylase OS=Coxiella burnetii (strain RSA 493 / Nine Mile phase I) GN=pyrF PE=1 SV=1		236	25.814737	0.007811	31.88	66.45	59.33	1.06	0.9	Q83E06
CBU_0532	Q83E05_COXBU COME operon protein 1 OS=Coxiella burnetii (strain RSA 493 / Nine Mile phase I) GN=CBU_0532 PE=4 SV=2		123	13.466618	0.0109458	75.84	62.54	45	-0.28	-0.75	Q83E05
CBU_0535	Q83E02_COXBU Uncharacterized protein OS=Coxiella burnetii (strain RSA 493 / Nine Mile phase I) GN=CBU_0535 PE=4 SV=1		323	35.969755	0.010228	27.02	261.98	326.62	3.28	3.6	Q83E02
CBU_0540	SMC_COXBU Chromosome partition protein Smc OS=Coxiella burnetii (strain RSA 493 / Nine Mile phase I) GN=smc PE=3 SV=1		1169	133.45149	0.0051353	40.93	3.29	4.74	-3.64	-3.11	Q81ZL2
CBU_0541	Q83DZ7_COXBU Cell division protein ZipA OS=Coxiella burnetii (strain RSA 493 / Nine Mile phase I) GN=zipA PE=3 SV=2		230	26.663725	0.0053257	43.17	2.57	0	-4.07	-10	Q83DZ7
CBU_0542	DNLJ_COXBU DNA ligase OS=Coxiella burnetii (strain RSA 493 / Nine Mile phase I) GN=ligA PE=3 SV=2		673	75.481366	0.0094224	2.68	17.59	28.06	2.71	3.39	Q83DZ6
CBU_0545	Q83DZ3_COXBU LemA OS=Coxiella burnetii (strain RSA 493 / Nine Mile phase I) GN=lemA PE=4 SV=2		215	24.716065	0.0094224	95.17	557.35	566.41	2.55	2.57	Q83DZ3
CBU_0546	Q83DZ2_COXBU Endopeptidase OS=Coxiella burnetii (strain RSA 493 / Nine Mile phase I) GN=htpX PE=3 SV=1		348	39.316816	0.0095396	23.35	56.96	101.05	1.29	2.11	Q83DZ2
CBU_0548	Q83DZ0_COXBU Membrane-bound lytic murein transglycosylase B OS=Coxiella burnetii (strain RSA 493 / Nine Mile phase I) GN=CBU_0548 PE=4 SV=1		334	38.683345	0.0098472	30.63	77.96	76.04	1.35	1.31	Q83DZ0
CBU_0550	Q83DY8_COXBU Cell elongation specific D,D-transpeptidase OS=Coxiella burnetii (strain RSA 493 / Nine Mile phase I) GN=pbpA PE=4 SV=2		615	69.950664	0.0094517	1.96	0.48	0	-2.02	-10	Q83DY8
CBU_0552	Q83DY7_COXBU Ribosomal silencing factor RsfS OS=Coxiella burnetii (strain RSA 493 / Nine Mile phase I) GN=rsfS PE=3 SV=1		116	13.172802	0.004729	85.6	2.55	25.26	-5.07	-1.76	Q83DY7
CBU_0558	Q83DY2_COXBU Rare lipoprotein B OS=Coxiella burnetii (strain RSA 493 / Nine Mile phase I) GN=CBU_0558 PE=4 SV=1		183	20.548021	0.0103306	36.18	61.44	67.61	0.76	0.9	Q83DY2
CBU_0559	SYL_COXBU Leucine--tRNA ligase OS=Coxiella burnetii (strain RSA 493 / Nine Mile phase I) GN=leuS PE=3 SV=1		820	94.181594	0.007562	69.72	60.98	69.89	-0.19	0	Q83DY1
CBU_0560	Q83DY0_COXBU Hypothetical cytosolic protein OS=Coxiella burnetii (strain RSA 493 / Nine Mile phase I) GN=CBU_0560 PE=1 SV=1		410	46.378182	0.0066392	65.32	65.67	68.3	0.01	0.06	Q83DY0
CBU_0562a	B5QS99_COXBU Uncharacterized protein OS=Coxiella burnetii (strain RSA 493 / Nine Mile phase I) GN=CBU_0562.1 PE=4 SV=1		140	15.289393	0.0068735	15.05	29.59	9.3	0.98	-0.69	B5QS99
CBU_0565	Q83DX7_COXBU Magnesium and cobalt efflux protein OS=Coxiella burnetii (strain RSA 493 / Nine Mile phase I) GN=corC PE=4 SV=2		283	32.325021	0.0050972	24.46	17.77	21.86	-0.46	-0.16	Q83DX7
CBU_0567	YBEY_COXBU Endoribonuclease YbeY OS=Coxiella burnetii (strain RSA 493 / Nine Mile phase I) GN=ybeY PE=3 SV=1		152	17.267467	0.0044624	3.96	23.36	21.42	2.56	2.44	Q83DX5
CBU_0568	Q83DX4_COXBU PhoH-like protein OS=Coxiella burnetii (strain RSA 493 / Nine Mile phase I) GN=CBU_0568 PE=4 SV=1		323	36.664185	0.0061255	1.86	83.36	92.74	5.48	5.64	Q83DX4
CBU_0570	Q83DX2_COXBU Amino acid permease OS=Coxiella burnetii (strain RSA 493 / Nine Mile phase I) GN=CBU_0570 PE=4 SV=1		560	62.390699	0.0093052	23.11	0	0	-10	-10	Q83DX2
CBU_0572	Q83DX0_COXBU Cytosol aminopeptidase OS=Coxiella burnetii (strain RSA 493 / Nine Mile phase I) GN=CBU_0572 PE=1 SV=1		458	50.838364	0.0054653	111.69	410.22	378.93	1.88	1.76	Q83DX0
CBU_0573	Q83DW9_COXBU Acyl-CoA dehydrogenase, medium-chain specific OS=Coxiella burnetii (strain RSA 493 / Nine Mile phase I) GN=CBU_0573 PE=4 SV=1		715	79.919657	0.008353	0	21.52	38.71	10	10	Q83DW9
CBU_0574	Q83DW8_COXBU 3-ketoacyl-CoA thiolase OS=Coxiella burnetii (strain RSA 493 / Nine Mile phase I) GN=CBU_0574 PE=3 SV=1		431	47.023516	0.0083384	16.76	24.71	47.59	0.56	1.51	Q83DW8
CBU_0576	Q83DW6_COXBU Enoyl-CoA hydratase OS=Coxiella burnetii (strain RSA 493 / Nine Mile phase I) GN=yfcX PE=4 SV=2		683	75.706257	0.0095835	29.52	129.53	182.11	2.13	2.63	Q83DW6
CBU_0582	Q83DW0_COXBU BolA OS=Coxiella burnetii (strain RSA 493 / Nine Mile phase I) GN=bolA PE=1 SV=1		79	8.8603293	0.0055161	57.14	0	0	-10	-10	Q83DW0
CBU_0583	Q83DV9_COXBU Glutaredoxin OS=Coxiella burnetii (strain RSA 493 / Nine Mile phase I) GN=CBU_0583 PE=3 SV=1		99	11.239726	0.0049067	705.17	23.91	0	-4.88	-10	Q83DV9
CBU_0586	Q83DV6_COXBU Pyridine nucleotide-disulfide oxidoreductase family OS=Coxiella burnetii (strain RSA 493 / Nine Mile phase I) GN=CBU_0586 PE=4 SV=1		1171	131.91664	0.006522	0.26	39.16	34.76	7.25	7.08	Q83DV6
CBU_0587	GLPE_COXBU Thiosulfate sulfurtransferase GlpE OS=Coxiella burnetii (strain RSA 493 / Nine Mile phase I) GN=glpE PE=3 SV=1		107	12.336114	0.0057319	78.74	16.59	3.04	-2.25	-4.69	Q83DV5
CBU_0589	Q83DV3_COXBU Ferredoxin, 2Fe-2s OS=Coxiella burnetii (strain RSA 493 / Nine Mile phase I) GN=CBU_0589 PE=4 SV=1		132	14.98752	0.0065513	6.84	44.83	56.73	2.71	3.05	Q83DV3
CBU_0598	Q83DU4_COXBU Bis(5'-adenosyl)-triphosphatase OS=Coxiella burnetii (strain RSA 493 / Nine Mile phase I) GN=CBU_0598 PE=3 SV=2		187	21.229855	0.0053003	28.96	30.06	17.41	0.05	-0.73	Q83DU4
CBU_0607	Q83DT5_COXBU Diphosphomevalonate decarboxylase OS=Coxiella burnetii (strain RSA 493 / Nine Mile phase I) GN=mvaD PE=3 SV=1		503	56.810392	0.0087339	6.58	7.06	12.95	0.1	0.98	Q83DT5
CBU_0610	Q820W8_COXBU 3-hydroxy-3-methylglutaryl-coenzyme A reductase OS=Coxiella burnetii (strain RSA 493 / Nine Mile phase I) GN=CBU_0610 PE=3 SV=1		357	38.899177	0.0085581	31.19	0	0	-10	-10	Q820W8
CBU_0611	Q83DT2_COXBU Outer membrane protein assembly factor BamA OS=Coxiella burnetii (strain RSA 493 / Nine Mile phase I) GN=yaeT PE=3 SV=1		803	90.681187	0.0097886	325.27	357.78	318.72	0.14	-0.03	Q83DT2
CBU_0612	Q83DT1_COXBU Outer membrane protein OS=Coxiella burnetii (strain RSA 493 / Nine Mile phase I) GN=ompH PE=4 SV=1		165	18.793979	0.0101987	266.26	229.53	219.05	-0.21	-0.28	Q83DT1
CBU_0613	LPXD_COXBU UDP-3-O-acylglucosamine N-acyltransferase OS=Coxiella burnetii (strain RSA 493 / Nine Mile phase I) GN=lpxD PE=3 SV=1		342	36.230991	0.0082651	61.59	3.46	0	-4.15	-10	Q83DT0
CBU_0614	FABZ_COXBU 3-hydroxyacyl-[acyl-carrier-protein] dehydratase FabZ OS=Coxiella burnetii (strain RSA 493 / Nine Mile phase I) GN=fabZ PE=3 SV=1		145	16.340941	0.0097886	132.82	59.17	76.35	-1.17	-0.8	Q820W7
CBU_0615	Q820W6_COXBU Acyl-[acyl-carrier-protein]--UDP-N-acetylglucosamine O-acyltransferase OS=Coxiella burnetii (strain RSA 493 / Nine Mile phase I) GN=lpxA PE=3 SV=1		259	28.13642	0.0083823	44.15	28.56	20.11	-0.63	-1.13	Q820W6
CBU_0621	Q83DS4_COXBU NAD-dependent oxidoreductase OS=Coxiella burnetii (strain RSA 493 / Nine Mile phase I) GN=CBU_0621 PE=4 SV=1		327	36.19325	0.0065806	196.93	30.76	47.8	-2.68	-2.04	Q83DS4
CBU_0628	Q83DR7_COXBU Inorganic pyrophosphatase OS=Coxiella burnetii (strain RSA 493 / Nine Mile phase I) GN=ppa PE=1 SV=1		175	19.624125	0.0050591	718.75	547.79	524.69	-0.39	-0.45	Q83DR7
CBU_0629	Q83DR6_COXBU Proline dehydrogenase OS=Coxiella burnetii (strain RSA 493 / Nine Mile phase I) GN=putA PE=3 SV=1		1046	116.33143	0.0062905	190.16	218.37	250.59	0.2	0.4	Q83DR6

CBU_0630	MIP_COXBU Peptidyl-prolyl cis-trans isomerase Mip OS=Coxiella burnetii (strain RSA 493 / Nine Mile phase I) GN=mip PE=1 SV=2	230	25.483408	0.0102134	430.44	424.52	404.89	-0.02	-0.09	P51752
CBU_0631	Q83DR5_COXBU Phosphoribosylformylglycinamidine synthase OS=Coxiella burnetii (strain RSA 493 / Nine Mile phase I) GN=purL PE=4 SV=2	1324	146.44107	0.0063286	10	29.27	22.38	1.55	1.16	Q83DR5
CBU_0632	Q83DR4_COXBU Uncharacterized protein OS=Coxiella burnetii (strain RSA 493 / Nine Mile phase I) GN=CBU_0632 PE=4 SV=1	99	11.859611	0.0045132	1109.43	1007.17	1266.26	-0.14	0.19	Q83DR4
CBU_0637	Q83DQ9_COXBU Coenzyme PQQ synthesis protein C OS=Coxiella burnetii (strain RSA 493 / Nine Mile phase I) GN=CBU_0637 PE=4 SV=1	253	29.383874	0.0060747	53.52	0	0	-10	-10	Q83DQ9
CBU_0638	Q83DQ8_COXBU Dihydrolipoamide acetyltransferase component of pyruvate dehydrogenase complex OS=Coxiella burnetii (strain RSA 493 / Nine Mile phase I) GN=CBU_0638 PE=3 SV=1	378	40.802511	0.005021	159.21	174.55	180.89	0.13	0.18	Q83DQ8
CBU_0639	Q83DQ7_COXBU Pyruvate dehydrogenase E1 component beta subunit OS=Coxiella burnetii (strain RSA 493 / Nine Mile phase I) GN=CBU_0639 PE=4 SV=1	326	35.436683	0.0060112	104.3	124.34	185.78	0.25	0.83	Q83DQ7
CBU_0640	Q83DQ6_COXBU Pyruvate dehydrogenase E1 component alpha subunit OS=Coxiella burnetii (strain RSA 493 / Nine Mile phase I) GN=CBU_0640 PE=4 SV=1	368	41.093501	0.0051226	165.99	243.61	273.4	0.55	0.72	Q83DQ6
CBU_0641	Q83DQ5_COXBU Leucine dehydrogenase OS=Coxiella burnetii (strain RSA 493 / Nine Mile phase I) GN=CBU_0641 PE=3 SV=2	382	42.259769	0.0062905	86.65	48.8	69.9	-0.83	-0.31	Q83DQ5
CBU_0643	Q83DQ3_COXBU Riboflavin biosynthesis protein RibD OS=Coxiella burnetii (strain RSA 493 / Nine Mile phase I) GN=ribD PE=3 SV=1	354	39.613777	0.0082358	0	1.67	18.4	10	10	Q83DQ3
CBU_0646	Q83DQ0_COXBU Riboflavin synthase alpha chain OS=Coxiella burnetii (strain RSA 493 / Nine Mile phase I) GN=ribE PE=4 SV=2	207	23.070861	0.0062524	69.78	2.86	7.86	-4.61	-3.15	Q83DQ0
CBU_0647	Q83DP9_COXBU GTP cyclohydrolase II OS=Coxiella burnetii (strain RSA 493 / Nine Mile phase I) GN=ribA PE=3 SV=2	387	42.643083	0.0057446	15.55	0	0	-10	-10	Q83DP9
CBU_0648	RISB_COXBU 6,7-dimethyl-8-ribityllumazine synthase OS=Coxiella burnetii (strain RSA 493 / Nine Mile phase I) GN=ribH PE=1 SV=1	151	16.572667	0.0070786	709.44	619.18	526.15	-0.2	-0.43	Q83DP8
CBU_0656	Y656_COXBU Nucleoid-associated protein CBU_0656 OS=Coxiella burnetii (strain RSA 493 / Nine Mile phase I) GN=CBU_0656 PE=3 SV=1	110	12.077077	0.0047671	90.27	107.59	71.04	0.25	-0.35	Q83DP1
CBU_0658	Y658_COXBU Uncharacterized protein CBU_0658 OS=Coxiella burnetii (strain RSA 493 / Nine Mile phase I) GN=CBU_0658 PE=1 SV=1	143	15.953934	0.0046655	1933.85	0	0	-10	-10	Q83DN9
CBU_0659	Q83DN8_COXBU DNA polymerase III subunit gamma/tau OS=Coxiella burnetii (strain RSA 493 / Nine Mile phase I) GN=dnaZX PE=4 SV=1	509	56.786592	0.0059858	8.28	0	0	-10	-10	Q83DN8
CBU_0666	DAPE_COXBU Succinyl-diaminopimelate desuccinylase OS=Coxiella burnetii (strain RSA 493 / Nine Mile phase I) GN=dapE PE=3 SV=1	374	41.59977	0.0058716	1.61	12.66	14.8	2.98	3.2	Q83DN2
CBU_0667	DAPD_COXBU 2,3,4,5-tetrahydropyridine-2,6-dicarboxylate N-succinyltransferase OS=Coxiella burnetii (strain RSA 493 / Nine Mile phase I) GN=dapD PE=3 SV=1	271	29.811394	0.0073862	53.3	115.73	91.31	1.12	0.78	Q83DN1
CBU_0674	Q83DM5_COXBU Phosphoheptose isomerase OS=Coxiella burnetii (strain RSA 493 / Nine Mile phase I) GN=CBU_0674 PE=4 SV=2	196	21.766972	0.0067417	150.46	0	0	-10	-10	Q83DM5
CBU_0675	Q83DM4_COXBU Transaldolase OS=Coxiella burnetii (strain RSA 493 / Nine Mile phase I) GN=CBU_0675 PE=4 SV=1	247	27.448033	0.0054019	283.86	0	0	-10	-10	Q83DM4
CBU_0676	H7C7E7_COXBU UDP-glucose 4-epimerase OS=Coxiella burnetii (strain RSA 493 / Nine Mile phase I) GN=CBU_0676 PE=4 SV=1	337	37.715287	0.0054272	305.38	6.15	1.93	-5.63	-7.3	H7C7E7
CBU_0677	B5U8Q0_COXBU NAD dependent epimerase/dehydratase family OS=Coxiella burnetii (strain RSA 493 / Nine Mile phase I) GN=CBU_0677 PE=4 SV=1	345	38.363592	0.0068003	66.29	0	0	-10	-10	B5U8Q0
CBU_0701	H7C7D8_COXBU 3'(2'),5'-bisphosphate nucleotidase OS=Coxiella burnetii (strain RSA 493 / Nine Mile phase I) GN=cysQ-2 PE=4 SV=1	277	30.626143	0.0076938	11.95	0	0	-10	-10	H7C7D8
CBU_0704	H7C7G2_COXBU Polysaccharide export ATP-binding protein OS=Coxiella burnetii (strain RSA 493 / Nine Mile phase I) GN=rfbI PE=3 SV=1	258	28.8681	0.0090122	5.83	0	0	-10	-10	H7C7G2
CBU_0712	Q83DL2_COXBU Response regulator OS=Coxiella burnetii (strain RSA 493 / Nine Mile phase I) GN=gacA.1 PE=4 SV=1	211	23.586979	0.0095103	0	14.02	3.09	10	10	Q83DL2
CBU_0714	Q83DL0_COXBU Uncharacterized protein OS=Coxiella burnetii (strain RSA 493 / Nine Mile phase I) GN=CBU_0714 PE=4 SV=2	136	15.65056	0.0042212	0	23.93	126.89	10	10	Q83DL0
CBU_0718	Q83DK8_COXBU Hypothetical membrane associated protein OS=Coxiella burnetii (strain RSA 493 / Nine Mile phase I) GN=CBU_0718 PE=4 SV=1	94	10.394581	0.0105356	326.52	22662.75	16917.79	6.12	5.7	Q83DK8
CBU_0719	Q83DK7_COXBU Uncharacterized protein OS=Coxiella burnetii (strain RSA 493 / Nine Mile phase I) GN=CBU_0719 PE=4 SV=1	141	15.474635	0.009686	0	323.15	344.08	10	10	Q83DK7
CBU_0720	Q83DK6_COXBU Agmatinase OS=Coxiella burnetii (strain RSA 493 / Nine Mile phase I) GN=CBU_0720 PE=3 SV=1	293	32.290271	0.0045513	8.22	16.16	25.56	0.98	1.64	Q83DK6
CBU_0721	DHSL_COXBU Deoxyhypusine synthase-like protein OS=Coxiella burnetii (strain RSA 493 / Nine Mile phase I) GN=CBU_0721 PE=3 SV=1	352	39.526628	0.0054146	174.39	59.68	81.4	-1.55	-1.1	P59650
CBU_0722	Q83DK5_COXBU Ornithine decarboxylase OS=Coxiella burnetii (strain RSA 493 / Nine Mile phase I) GN=CBU_0722 PE=3 SV=2	400	45.004825	0.0058081	6.77	0	0	-10	-10	Q83DK5
CBU_0727	Q83DK3_COXBU ABC transporter permease protein OS=Coxiella burnetii (strain RSA 493 / Nine Mile phase I) GN=CBU_0727 PE=4 SV=1	377	40.838132	0.0057954	4.79	62	88.1	3.69	4.2	Q83DK3
CBU_0729	Q83DK1_COXBU Hypothetical exported protein OS=Coxiella burnetii (strain RSA 493 / Nine Mile phase I) GN=CBU_0729 PE=4 SV=1	308	33.930964	0.0096567	2.93	53.8	57.09	4.2	4.28	Q83DK1
CBU_0730	Q83DK0_COXBU Uncharacterized protein OS=Coxiella burnetii (strain RSA 493 / Nine Mile phase I) GN=CBU_0730 PE=4 SV=1	199	22.378882	0.0100229	25.71	72.85	109.63	1.5	2.09	Q83DK0
CBU_0736	Q83DJ4_COXBU Hypothetical exported protein OS=Coxiella burnetii (strain RSA 493 / Nine Mile phase I) GN=CBU_0736 PE=4 SV=1	217	23.986361	0.0103159	22.19	0	0	-10	-10	Q83DJ4
CBU_0737	TIG_COXBU Trigger factor OS=Coxiella burnetii (strain RSA 493 / Nine Mile phase I) GN=tig PE=1 SV=1	442	50.197234	0.0051733	195.39	87.02	85.45	-1.17	-1.19	Q83DJ3
CBU_0738	CLPP_COXBU ATP-dependent Clp protease proteolytic subunit OS=Coxiella burnetii (strain RSA 493 / Nine Mile phase I) GN=clpP PE=1 SV=1	195	21.570039	0.0060747	455.23	364.15	364.01	-0.32	-0.32	Q83DJ2
CBU_0739	CLPX_COXBU ATP-dependent Clp protease ATP-binding subunit ClpX OS=Coxiella burnetii (strain RSA 493 / Nine Mile phase I) GN=clpX PE=3 SV=1	422	46.720768	0.0063032	70.59	0	0	-10	-10	Q83DJ1
CBU_0740	Q83DJ0_COXBU Lon protease OS=Coxiella burnetii (strain RSA 493 / Nine Mile phase I) GN=lon PE=3 SV=1	817	91.566508	0.0091587	289.5	56.13	68.55	-2.37	-2.08	Q83DJ0
CBU_0743	Q83DI7_COXBU Phosphocarrier protein HPr OS=Coxiella burnetii (strain RSA 493 / Nine Mile phase I) GN=ptsH PE=4 SV=1	89	10.024278	0.0086899	27.05	13.3	3.66	-1.02	-2.89	Q83DI7
CBU_0744	Q820W5_COXBU Hpr(Ser) kinase OS=Coxiella burnetii (strain RSA 493 / Nine Mile phase I) GN=CBU_0744 PE=1 SV=1	192	21.463334	0.0064429	14.11	29.28	35.61	1.05	1.34	Q820W5
CBU_0745	Q83DI6_COXBU Ribosome-associated factor Y OS=Coxiella burnetii (strain RSA 493 / Nine Mile phase I) GN=CBU_0745 PE=1 SV=1	96	10.990829	0.0097593	257.03	70.89	67.84	-1.86	-1.92	Q83DI6
CBU_0746	Q83DI5_COXBU LPS ABC transporter ATP-binding protein OS=Coxiella burnetii (strain RSA 493 / Nine Mile phase I) GN=CBU_0746 PE=3 SV=1	249	27.804774	0.0091294	44.71	19.01	43.15	-1.23	-0.05	Q83DI5

CBU_0750	Q83DI1_COXBU Arabinose 5-phosphate isomerase OS=Coxiella burnetii (strain RSA 493 / Nine Mile phase I) GN=CBU_0750 PE=3 SV=1	324	34.501203	0.0069028	9.29	24.66	23.11	1.41	1.32	Q83DI1
CBU_0753	Q83DH8_COXBU Acriflavin resistance plasma membrane protein OS=Coxiella burnetii (strain RSA 493 / Nine Mile phase I) GN=CBU_0753 PE=4 SV=1	1022	110.90268	0.0077964	0.88	0.87	0.32	-0.02	-1.47	Q83DH8
CBU_0754	Q83DH7_COXBU Acriflavin resistance periplasmic protein OS=Coxiella burnetii (strain RSA 493 / Nine Mile phase I) GN=CBU_0754 PE=4 SV=1	348	37.627324	0.0099204	5.19	17	10.29	1.71	0.99	Q83DH7
CBU_0755	Q83DH6_COXBU Endopeptidase OS=Coxiella burnetii (strain RSA 493 / Nine Mile phase I) GN=degP.2 PE=4 SV=1	451	47.723929	0.0091294	211.51	494	425.96	1.22	1.01	Q83DH6
CBU_0758	Q83DH4_COXBU Outer membrane protein assembly factor BamD OS=Coxiella burnetii (strain RSA 493 / Nine Mile phase I) GN=bamD PE=3 SV=2	272	30.860955	0.0098911	356.23	366.58	384.27	0.04	0.11	Q83DH4
CBU_0762	Q83DH1_COXBU Uncharacterized protein OS=Coxiella burnetii (strain RSA 493 / Nine Mile phase I) GN=CBU_0762 PE=4 SV=1	208	23.548298	0.0086606	4.34	18.49	36	2.09	3.05	Q83DH1
CBU_0770	Q83DG6_COXBU Hypothetical ATPase OS=Coxiella burnetii (strain RSA 493 / Nine Mile phase I) GN=CBU_0770 PE=4 SV=2	421	47.9677	0.0094956	0	12.65	13.92	10	10	Q83DG6
CBU_0771	Q83DG5_COXBU Methylisocitrate lyase OS=Coxiella burnetii (strain RSA 493 / Nine Mile phase I) GN=prpB PE=4 SV=1	290	31.721244	0.0057446	35.28	80.6	103.3	1.19	1.55	Q83DG5
CBU_0772	Q83DG4_COXBU Citrate synthase OS=Coxiella burnetii (strain RSA 493 / Nine Mile phase I) GN=prpC PE=1 SV=1	372	41.508193	0.0073276	65.52	149.53	188.19	1.19	1.52	Q83DG4
CBU_0780	Q83DF7_COXBU Response regulator OS=Coxiella burnetii (strain RSA 493 / Nine Mile phase I) GN=gacA.2 PE=4 SV=1	216	24.215173	0.0096128	52.94	104.1	143.21	0.98	1.44	Q83DF7
CBU_0795	GCH1_COXBU GTP cyclohydrolase 1 OS=Coxiella burnetii (strain RSA 493 / Nine Mile phase I) GN=folE PE=3 SV=1	184	20.745989	0.0093784	40.89	120.6	111.49	1.56	1.45	Q83DE3
CBU_0796	Q83DE2_COXBU Adenosine 5'-monophosphoramidase OS=Coxiella burnetii (strain RSA 493 / Nine Mile phase I) GN=CBU_0796 PE=4 SV=1	113	12.455278	0.0063159	21.3	89.02	95.09	2.06	2.16	Q83DE2
CBU_0798	Q83DE0_COXBU Multidrug resistance protein A OS=Coxiella burnetii (strain RSA 493 / Nine Mile phase I) GN=CBU_0798 PE=4 SV=1	331	37.053616	0.0098325	37.27	153.75	220.35	2.04	2.56	Q83DE0
CBU_0804	Q83DD4_COXBU Acriflavin resistance plasma membrane protein OS=Coxiella burnetii (strain RSA 493 / Nine Mile phase I) GN=CBU_0804 PE=4 SV=1	1012	109.92643	0.0084849	0	2.34	2.25	10	10	Q83DD4
CBU_0808	SYV_COXBU Valine--tRNA ligase OS=Coxiella burnetii (strain RSA 493 / Nine Mile phase I) GN=valS PE=3 SV=1	920	106.5625	0.0086606	50.7	45.02	36.81	-0.17	-0.46	Q83DD0
CBU_0811	RF3_COXBU Peptide chain release factor 3 OS=Coxiella burnetii (strain RSA 493 / Nine Mile phase I) GN=prfC PE=1 SV=1	525	59.421718	0.0063286	6.3	5.07	1.86	-0.31	-1.76	Q83DC7
CBU_0818	Q83DC0_COXBU Transcriptional regulator, TetR family OS=Coxiella burnetii (strain RSA 493 / Nine Mile phase I) GN=CBU_0818 PE=4 SV=1	193	22.136061	0.0097153	307.15	366.39	377.91	0.25	0.3	Q83DC0
CBU_0819	Q83DB9_COXBU Glutathione S-transferase OS=Coxiella burnetii (strain RSA 493 / Nine Mile phase I) GN=CBU_0819 PE=3 SV=1	224	25.374411	0.0093345	55.08	0	0	-10	-10	Q83DB9
CBU_0823	Q83DB5_COXBU NAD-dependent malic enzyme OS=Coxiella burnetii (strain RSA 493 / Nine Mile phase I) GN=sfcA PE=3 SV=1	565	63.392007	0.0067563	18.64	6.81	4.03	-1.45	-2.21	Q83DB5
CBU_0824	Q83DB4_COXBU Adenylosuccinate lyase OS=Coxiella burnetii (strain RSA 493 / Nine Mile phase I) GN=purB PE=4 SV=1	461	52.455512	0.0074155	16.32	33.37	32.49	1.03	0.99	Q83DB4
CBU_0825	Q83DB3_COXBU Nucleotide-sugar aminotransferase OS=Coxiella burnetii (strain RSA 493 / Nine Mile phase I) GN=CBU_0825 PE=3 SV=1	369	40.568641	0.0058335	27.73	0	0	-10	-10	Q83DB3
CBU_0829	Q83DA9_COXBU NAD dependent epimerase/dehydratase family OS=Coxiella burnetii (strain RSA 493 / Nine Mile phase I) GN=CBU_0829 PE=4 SV=2	331	36.791601	0.0061128	37.27	0	0	-10	-10	Q83DA9
CBU_0844	Q83D94_COXBU UDP-N-acetylglucosamine 4-epimerase OS=Coxiella burnetii (strain RSA 493 / Nine Mile phase I) GN=CBU_0844 PE=4 SV=2	339	38.50262	0.0079575	80.78	66.33	93.17	-0.28	0.21	Q83D94
CBU_0845	Q83D93_COXBU UDP-N-acetyl-D-galactosamine 6-dehydrogenase OS=Coxiella burnetii (strain RSA 493 / Nine Mile phase I) GN=CBU_0845 PE=3 SV=1	429	47.611472	0.0053257	9.12	17.93	13.66	0.98	0.58	Q83D93
CBU_0847	Q820W4_COXBU 3-hydroxyacyl CoA dehydrogenase OS=Coxiella burnetii (strain RSA 493 / Nine Mile phase I) GN=CBU_0847 PE=3 SV=1	254	26.905184	0.0088364	2.37	5.82	7.69	1.3	1.7	Q820W4
CBU_0849	Q83D90_COXBU UTP--glucose-1-phosphate uridylyltransferase OS=Coxiella burnetii (strain RSA 493 / Nine Mile phase I) GN=galU PE=4 SV=1	295	32.622274	0.0064302	286.63	89.26	73.95	-1.68	-1.95	Q83D90
CBU_0851	RS15_COXBU 30S ribosomal protein S15 OS=Coxiella burnetii (strain RSA 493 / Nine Mile phase I) GN=rpsO PE=3 SV=1	89	10.291711	0.0108872	300.91	136.3	113.41	-1.14	-1.41	Q83D88
CBU_0852	PNP_COXBU Polyribonucleotide nucleotidyltransferase OS=Coxiella burnetii (strain RSA 493 / Nine Mile phase I) GN=pnp PE=3 SV=1	696	76.265877	0.0052622	465.64	431.06	400.46	-0.11	-0.22	Q83D87
CBU_0856	MSBA_COXBU Lipid A export ATP-binding/permease protein MsbA OS=Coxiella burnetii (strain RSA 493 / Nine Mile phase I) GN=msbA PE=3 SV=1	589	65.896276	0.0097739	3.07	0	0	-10	-10	Q83D84
CBU_0858	Q83D82_COXBU Glutamine-dependent NAD(+) synthetase OS=Coxiella burnetii (strain RSA 493 / Nine Mile phase I) GN=nadE PE=3 SV=1	542	60.247589	0.0055288	22.76	48.04	37.85	1.08	0.73	Q83D82
CBU_0864	RS6_COXBU 30S ribosomal protein S6 OS=Coxiella burnetii (strain RSA 493 / Nine Mile phase I) GN=rpsF PE=3 SV=1	127	14.550501	0.0078989	831.66	316.84	310.23	-1.39	-1.42	Q83D76
CBU_0865	RS18_COXBU 30S ribosomal protein S18 OS=Coxiella burnetii (strain RSA 493 / Nine Mile phase I) GN=rpsR PE=3 SV=1	73	8.5294178	0.009979	239.08	68.9	57.99	-1.79	-2.04	Q83D75
CBU_0867	RL9_COXBU 50S ribosomal protein L9 OS=Coxiella burnetii (strain RSA 493 / Nine Mile phase I) GN=rplI PE=1 SV=1	152	16.551931	0.0062144	289.04	179.08	160.66	-0.69	-0.85	Q83D73
CBU_0868	Q83D72_COXBU Replicative DNA helicase OS=Coxiella burnetii (strain RSA 493 / Nine Mile phase I) GN=dnaB PE=4 SV=1	610	67.746515	0.0062905	1.97	6.31	9.07	1.68	2.2	Q83D72
CBU_0869	ALR_COXBU Alanine racemase OS=Coxiella burnetii (strain RSA 493 / Nine Mile phase I) GN=alr PE=3 SV=1	364	39.918912	0.0092173	0	14.63	11.63	10	10	Q83D71
CBU_0874	AROC_COXBU Chorismate synthase OS=Coxiella burnetii (strain RSA 493 / Nine Mile phase I) GN=aroC PE=3 SV=1	352	37.907831	0.0094956	13.68	101.71	73.08	2.89	2.42	Q83D67
CBU_0875	Q83D66_COXBU Aspartate-semialdehyde dehydrogenase OS=Coxiella burnetii (strain RSA 493 / Nine Mile phase I) GN=asd PE=3 SV=1	340	37.253307	0.0053765	13.28	178.4	245.16	3.75	4.21	Q83D66
CBU_0884	Q83D58_COXBU GTP-binding protein OS=Coxiella burnetii (strain RSA 493 / Nine Mile phase I) GN=bipA PE=4 SV=1	602	67.211284	0.0058589	49.49	11.8	22.18	-2.07	-1.16	Q83D58
CBU_0889	Q83D54_COXBU Thiol:disulfide interchange protein OS=Coxiella burnetii (strain RSA 493 / Nine Mile phase I) GN=dsbA PE=4 SV=2	199	21.773594	0.0101987	42.34	80.29	76.9	0.92	0.86	Q83D54
CBU_0890	Q83D53_COXBU Zn-dependent hydrolase, glyoxalase II family OS=Coxiella burnetii (strain RSA 493 / Nine Mile phase I) GN=CBU_0890 PE=4 SV=1	233	26.267431	0.0059731	14.21	0	0	-10	-10	Q83D53
CBU_0891	Q83D52_COXBU Hypothetical exported membrane associated protein OS=Coxiella burnetii (strain RSA 493 / Nine Mile phase I) GN=CBU_0891 PE=4 SV=1	312	34.330436	0.0051479	21.22	0	0	-10	-10	Q83D52
CBU_0893	ACCD_COXBU Acetyl-coenzyme A carboxylase carboxyl transferase subunit beta OS=Coxiella burnetii (strain RSA 493 / Nine Mile phase I) GN=accD PE=3 SV=1	291	32.192725	0.006522	14.48	95.57	134.27	2.72	3.21	Q83D51
CBU_0894	Q83D50_COXBU Folylpolyglutamate synthase OS=Coxiella burnetii (strain RSA 493 / Nine Mile phase I) GN=folC PE=4 SV=1	416	46.166739	0.0052622	22.42	0	0	-10	-10	Q83D50

CBU_0895	Q83D49_COXBU Cell division related protein OS=Coxiella burnetii (strain RSA 493 / Nine Mile phase I) GN=dedD PE=4 SV=2	205	22.907736	0.0104771	220.18	11.55	0	-4.25	-10	Q83D49
CBU_0897	Q83D47_COXBU Amidophosphoribosyltransferase OS=Coxiella burnetii (strain RSA 493 / Nine Mile phase I) GN=purF PE=3 SV=1	506	55.882612	0.0059858	29.14	49.12	63.71	0.75	1.13	Q83D47
CBU_0905	RL31_COXBU 50S ribosomal protein L31 OS=Coxiella burnetii (strain RSA 493 / Nine Mile phase I) GN=rpmE PE=3 SV=1	79	9.0544762	0.0095835	533.27	273.4	317.37	-0.96	-0.75	Q83D39
CBU_0907	Q83D37_COXBU YciL OS=Coxiella burnetii (strain RSA 493 / Nine Mile phase I) GN=yciL PE=3 SV=1	192	21.902318	0.0069175	70.53	29.28	16.96	-1.27	-2.06	Q83D37
CBU_0910	Q83D34_COXBU Hypothetical membrane associated protein OS=Coxiella burnetii (strain RSA 493 / Nine Mile phase I) GN=CBU_0910 PE=4 SV=1	324	36.871104	0.0054272	0	5.48	14.07	10	10	Q83D34
CBU_0915	Q83D29_COXBU Enhanced entry protein OS=Coxiella burnetii (strain RSA 493 / Nine Mile phase I) GN=enhB.1 PE=4 SV=1	169	19.338772	0.0086606	587.58	1078.45	949.85	0.88	0.69	Q83D29
CBU_0924	Q83D22_COXBU O-methyltransferase OS=Coxiella burnetii (strain RSA 493 / Nine Mile phase I) GN=CBU_0924 PE=1 SV=1	222	24.837045	0.0047671	73.2	151.94	178.94	1.05	1.29	Q83D22
CBU_0925	Q83D21_COXBU Membrane-bound lytic murein transglycosylase B OS=Coxiella burnetii (strain RSA 493 / Nine Mile phase I) GN=CBU_0925 PE=4 SV=1	333	38.44687	0.0102866	0	178.59	112.45	10	10	Q83D21
CBU_0928	PDXH_COXBU Pyridoxine/pyridoxamine 5'-phosphate oxidase OS=Coxiella burnetii (strain RSA 493 / Nine Mile phase I) GN=pdxH PE=3 SV=1	196	23.602817	0.0063667	208.8	306.44	277.43	0.55	0.41	Q83D18
CBU_0929	Q83D17_COXBU Short chain dehydrogenase OS=Coxiella burnetii (strain RSA 493 / Nine Mile phase I) GN=CBU_0929 PE=3 SV=1	281	31.602056	0.0102866	9.64	0	0	-10	-10	Q83D17
CBU_0932	GLPK_COXBU Glycerol kinase OS=Coxiella burnetii (strain RSA 493 / Nine Mile phase I) GN=glpK PE=3 SV=1	501	55.3341	0.0053638	0.6	5.32	0.65	3.15	0.11	Q83D14
CBU_0933	Q83D13_COXBU ABC transporter permease protein OS=Coxiella burnetii (strain RSA 493 / Nine Mile phase I) GN=CBU_0933 PE=4 SV=1	257	28.832739	0.0092905	12.88	0	0	-10	-10	Q83D13
CBU_0934	Q83D12_COXBU ABC transporter ATP-binding protein OS=Coxiella burnetii (strain RSA 493 / Nine Mile phase I) GN=CBU_0934 PE=4 SV=1	304	34.510299	0.0089683	0	30.17	42.84	10	10	Q83D12
CBU_0937	Y937_COXBU UPF0422 protein CBU_0937 OS=Coxiella burnetii (strain RSA 493 / Nine Mile phase I) GN=CBU_0937 PE=3 SV=1	465	51.334751	0.0091294	2158.81	2059.67	1767.39	-0.07	-0.29	Q83D09
CBU_0939	Q83D08_COXBU ASMA OS=Coxiella burnetii (strain RSA 493 / Nine Mile phase I) GN=CBU_0939 PE=4 SV=2	508	55.582737	0.0104917	26.66	0	0	-10	-10	Q83D08
CBU_0943	Q83D04_COXBU Sulfurtransferase OS=Coxiella burnetii (strain RSA 493 / Nine Mile phase I) GN=CBU_0943 PE=4 SV=2	146	16.564481	0.0088218	0	129.7	84.75	10	10	Q83D04
CBU_0946	Q83D01_COXBU RhuM OS=Coxiella burnetii (strain RSA 493 / Nine Mile phase I) GN=rhuM PE=4 SV=1	328	37.058814	0.0099351	49.54	169.59	194.57	1.78	1.97	Q83D01
CBU_0952	Y952_COXBU Uncharacterized protein CBU_0952 OS=Coxiella burnetii (strain RSA 493 / Nine Mile phase I) GN=CBU_0952 PE=1 SV=1	227	25.91745	0.0092759	177.63	510.94	492	1.52	1.47	Q83CZ8
CBU_0955	Q83CZ5_COXBU Response regulator OS=Coxiella burnetii (strain RSA 493 / Nine Mile phase I) GN=gacA.3 PE=4 SV=1	217	24.104207	0.0099644	36.05	68.17	78.03	0.92	1.11	Q83CZ5
CBU_0962	Q83CY9_COXBU Short chain dehydrogenase OS=Coxiella burnetii (strain RSA 493 / Nine Mile phase I) GN=CBU_0962 PE=3 SV=1	232	25.533262	0.007313	1066.17	1717.86	1469.45	0.69	0.46	Q83CY9
CBU_0963	BCP_COXBU Putative peroxiredoxin bcp OS=Coxiella burnetii (strain RSA 493 / Nine Mile phase I) GN=bcp PE=1 SV=1	151	16.821712	0.0079429	4999.95	6289.79	5487.9	0.33	0.13	Q83CY8
CBU_0965	Q83CY6_COXBU Cytochrome d ubiquinol oxidase subunit I OS=Coxiella burnetii (strain RSA 493 / Nine Mile phase I) GN=cydA-2 PE=4 SV=1	521	58.475504	0.0093784	75.66	69.28	30.62	-0.13	-1.3	Q83CY6
CBU_0968	Q83CY3_COXBU Phospholipase D OS=Coxiella burnetii (strain RSA 493 / Nine Mile phase I) GN=CBU_0968 PE=4 SV=1	176	20.204507	0.009979	41.03	70.61	70.3	0.78	0.78	Q83CY3
CBU_0973	Q83CX8_COXBU Isovaleryl-CoA dehydrogenase OS=Coxiella burnetii (strain RSA 493 / Nine Mile phase I) GN=CBU_0973 PE=3 SV=1	387	42.327538	0.0057573	62.2	133.79	147.24	1.1	1.24	Q83CX8
CBU_0974	Q83CX7_COXBU Acetyl-CoA acetyltransferase OS=Coxiella burnetii (strain RSA 493 / Nine Mile phase I) GN=CBU_0974 PE=3 SV=1	394	42.197694	0.0076792	9.93	103.63	166.94	3.38	4.07	Q83CX7
CBU_0975	Q83CX6_COXBU Methylcrotonyl-CoA carboxylase carboxyl transferase subunit OS=Coxiella burnetii (strain RSA 493 / Nine Mile phase I) GN=CBU_0975 PE=4 SV=2	554	61.001268	0.0075327	18.47	58.21	84.63	1.66	2.2	Q83CX6
CBU_0976	Q83CX5_COXBU Methylglutaconyl-CoA hydratase OS=Coxiella burnetii (strain RSA 493 / Nine Mile phase I) GN=CBU_0976 PE=4 SV=1	256	28.199692	0.0059858	74.05	88.99	108.11	0.27	0.55	Q83CX5
CBU_0979	Q83CX2_COXBU Lipoprotein OS=Coxiella burnetii (strain RSA 493 / Nine Mile phase I) GN=CBU_0979 PE=4 SV=1	100	11.517857	0.0110923	132.4	319.54	296.3	1.27	1.16	Q83CX2
CBU_0982	Q83CW9_COXBU Phospho-2-dehydro-3-deoxyheptonate aldolase OS=Coxiella burnetii (strain RSA 493 / Nine Mile phase I) GN=CBU_0982 PE=3 SV=1	360	39.58377	0.0077817	73.56	186.57	216.17	1.34	1.56	Q83CW9
CBU_0985	Q83CW7_COXBU Hypothetical cytosolic protein OS=Coxiella burnetii (strain RSA 493 / Nine Mile phase I) GN=CBU_0985 PE=4 SV=1	252	29.346608	0.0088364	1.19	4.7	0	1.98	-10	Q83CW7
CBU_0986	RLMB_COXBU 23S rRNA (guanosine-2'-O-)-methyltransferase RlmB OS=Coxiella burnetii (strain RSA 493 / Nine Mile phase I) GN=rlmB PE=3 SV=1	250	27.105565	0.00702	3.61	17.75	41.68	2.3	3.53	Q83CW6
CBU_0988	UNG_COXBU Uracil-DNA glycosylase OS=Coxiella burnetii (strain RSA 493 / Nine Mile phase I) GN=ung PE=1 SV=1	229	25.632482	0.0093052	57.82	0	0	-10	-10	Q83CW4
CBU_0993	DEF1_COXBU Peptide deformylase 1 OS=Coxiella burnetii (strain RSA 493 / Nine Mile phase I) GN=def1 PE=3 SV=1	170	19.305911	0.0082358	60.18	6.96	7.66	-3.11	-2.97	Q83CV9
CBU_0998	PURA_COXBU Adenylosuccinate synthetase OS=Coxiella burnetii (strain RSA 493 / Nine Mile phase I) GN=purA PE=3 SV=1	435	47.940816	0.0061001	29.05	21.09	16.47	-0.46	-0.82	Q83CV4
CBU_1000	LOLD_COXBU Lipoprotein-releasing system ATP-binding protein LolD OS=Coxiella burnetii (strain RSA 493 / Nine Mile phase I) GN=lolD PE=3 SV=1	233	25.797635	0.0089243	2.58	2.54	6.99	-0.02	1.44	Q83CV2
CBU_1001	Q83CV1_COXBU Lipoprotein releasing system transmembrane protein OS=Coxiella burnetii (strain RSA 493 / Nine Mile phase I) GN=lolC PE=4 SV=1	414	44.985503	0.010228	15.26	29.3	3.93	0.94	-1.96	Q83CV1
CBU_1018	Q83CT5_COXBU Uncharacterized protein OS=Coxiella burnetii (strain RSA 493 / Nine Mile phase I) GN=CBU_1018 PE=4 SV=1	118	13.813416	0.0090415	25.5	42.63	16.56	0.74	-0.62	Q83CT5
CBU_1021	Q83CT2_COXBU Nucleotidyltransferase OS=Coxiella burnetii (strain RSA 493 / Nine Mile phase I) GN=CBU_1021 PE=4 SV=1	147	17.05687	0.0054526	6.14	40.26	59.81	2.71	3.28	Q83CT2
CBU_1027	Q83CS6_COXBU ATPase OS=Coxiella burnetii (strain RSA 493 / Nine Mile phase I) GN=CBU_1027 PE=4 SV=1	463	50.652244	0.0098325	0	54.96	56.96	10	10	Q83CS6
CBU_1035	Q83CS0_COXBU Nicotinate phosphoribosyltransferase OS=Coxiella burnetii (strain RSA 493 / Nine Mile phase I) GN=CBU_1035 PE=3 SV=1	468	52.843807	0.0094956	0	19.6	24.35	10	10	Q83CS0
CBU_1039	Q83CR8_COXBU Cytochrome c oxidase polypeptide I OS=Coxiella burnetii (strain RSA 493 / Nine Mile phase I) GN=cyoB PE=3 SV=1	668	75.209403	0.009188	66.67	48.72	35.58	-0.45	-0.91	Q83CR8
CBU_1040	Q83CR7_COXBU Cytochrome c oxidase polypeptide II OS=Coxiella burnetii (strain RSA 493 / Nine Mile phase I) GN=cyoA PE=4 SV=1	298	34.182864	0.0089536	184.79	147.94	123.47	-0.32	-0.58	Q83CR7
CBU_1050	CSRA2_COXBU Carbon storage regulator homolog 2 OS=Coxiella burnetii (strain RSA 493 / Nine Mile phase I) GN=csrA2 PE=3 SV=1	70	7.9421906	0.0045894	77.38	557.94	97.68	2.85	0.34	Q83CQ8

CBU_1051	Q83CQ7_COXBU Aspartokinase OS=Coxiella burnetii (strain RSA 493 / Nine Mile phase I) GN=CBU_1051 PE=3 SV=1	409	44.060953	0.0056431	22.81	31.11	19.11	0.45	-0.26	Q83CQ7
CBU_1052	SYA_COXBU Alanine--tRNA ligase OS=Coxiella burnetii (strain RSA 493 / Nine Mile phase I) GN=alaS PE=3 SV=1	865	97.577425	0.0062905	113.76	199.76	202.52	0.81	0.83	Q83CQ6
CBU_1054	RECA_COXBU Protein RecA OS=Coxiella burnetii (strain RSA 493 / Nine Mile phase I) GN=recA PE=3 SV=1	343	37.086607	0.0057827	612.36	80.22	85.44	-2.93	-2.84	Q83CQ4
CBU_1055	Q83CQ3_COXBU Putative competence-damage protein OS=Coxiella burnetii (strain RSA 493 / Nine Mile phase I) GN=CBU_1055 PE=4 SV=1	166	17.64023	0.0098325	0	23.17	45.11	10	10	Q83CQ3
CBU_1060	SCPBL_COXBU Segregation and condensation protein B homolog OS=Coxiella burnetii (strain RSA 493 / Nine Mile phase I) GN=scpB PE=1 SV=1	209	23.685514	0.0045894	113.74	19.82	34.27	-2.52	-1.73	Q83CP9
CBU_1064	Q83CP5_COXBU Hypothetical cytosolic protein OS=Coxiella burnetii (strain RSA 493 / Nine Mile phase I) GN=CBU_1064 PE=4 SV=1	94	11.018652	0.0091587	601.83	358.83	204.37	-0.75	-1.56	Q83CP5
CBU_1065	Q83CP4_COXBU 2'-5' RNA ligase OS=Coxiella burnetii (strain RSA 493 / Nine Mile phase I) GN=CBU_1065 PE=4 SV=1	184	21.637693	0.0096714	0	19.3	24.77	10	10	Q83CP4
CBU_1067	TTCA_COXBU tRNA 2-thiocytidine biosynthesis protein TtcA OS=Coxiella burnetii (strain RSA 493 / Nine Mile phase I) GN=ttcA PE=3 SV=1	256	29.871529	0.0095542	5.88	0	0	-10	-10	Q83CP2
CBU_1073	Q83CN7_COXBU Hypothetical exported protein OS=Coxiella burnetii (strain RSA 493 / Nine Mile phase I) GN=CBU_1073 PE=4 SV=1	168	19.374187	0.0098765	46.57	88.06	75.59	0.92	0.7	Q83CN7
CBU_1074	Q83CN6_COXBU Hypoxanthine-guanine phosphoribosyltransferase OS=Coxiella burnetii (strain RSA 493 / Nine Mile phase I) GN=CBU_1074 PE=4 SV=1	186	20.424363	0.0055669	55.01	0	5.25	-10	-3.39	Q83CN6
CBU_1077	Q83CN4_COXBU Anhydromuramoyl-peptide exo-beta-N-acetylglucosaminidase OS=Coxiella burnetii (strain RSA 493 / Nine Mile phase I) GN=nagZ PE=4 SV=1	313	34.994984	0.0058716	10.58	9.45	16.64	-0.16	0.65	Q83CN4
CBU_1079	Q83CN2_COXBU Uncharacterized protein OS=Coxiella burnetii (strain RSA 493 / Nine Mile phase I) GN=CBU_1079 PE=4 SV=1	198	23.515838	0.0056177	50.15	11.95	11.51	-2.07	-2.12	Q83CN2
CBU_1080	Q83CN1_COXBU ErfK/YbiS/YcfS/YnhG family protein OS=Coxiella burnetii (strain RSA 493 / Nine Mile phase I) GN=CBU_1080 PE=4 SV=1	160	18.20139	0.0096567	11.28	9.25	24.42	-0.29	1.11	Q83CN1
CBU_1083	MUTL_COXBU DNA mismatch repair protein MutL OS=Coxiella burnetii (strain RSA 493 / Nine Mile phase I) GN=mutL PE=3 SV=1	574	64.527979	0.007313	6.82	0	1.7	-10	-2	Q83CM9
CBU_1088	NNR_COXBU Bifunctional NAD(P)H-hydrate repair enzyme Nnr OS=Coxiella burnetii (strain RSA 493 / Nine Mile phase I) GN=nnr PE=3 SV=1	488	51.64896	0.0056304	19.73	33.95	32.03	0.78	0.7	Q83CM5
CBU_1091	Q83CM3_COXBU Ribonuclease R OS=Coxiella burnetii (strain RSA 493 / Nine Mile phase I) GN=vacB PE=3 SV=2	749	86.636522	0.0093931	16.47	56.09	61.3	1.77	1.9	Q83CM3
CBU_1093	Q83CM1_COXBU Acriflavin resistance plasma membrane protein OS=Coxiella burnetii (strain RSA 493 / Nine Mile phase I) GN=CBU_1093 PE=4 SV=1	1019	112.25444	0.0068882	0	10.45	3.51	10	10	Q83CM1
CBU_1094	Q83CM0_COXBU Acriflavin resistance periplasmic protein OS=Coxiella burnetii (strain RSA 493 / Nine Mile phase I) GN=CBU_1094 PE=4 SV=1	380	41.019117	0.0097739	34.05	267.84	258.77	2.98	2.93	Q83CM0
CBU_1095	Q83CL9_COXBU Hypothetical exported protein OS=Coxiella burnetii (strain RSA 493 / Nine Mile phase I) GN=CBU_1095 PE=4 SV=2	187	20.223356	0.0071079	9.65	80.69	67.91	3.06	2.81	Q83CL9
CBU_1096	FUMC_COXBU Fumarate hydratase class II OS=Coxiella burnetii (strain RSA 493 / Nine Mile phase I) GN=fumC PE=3 SV=2	459	49.800653	0.0062397	34.09	165.66	210.69	2.28	2.63	Q83CL8
CBU_1097	Q83CL7_COXBU Alpha-acetolactate decarboxylase OS=Coxiella burnetii (strain RSA 493 / Nine Mile phase I) GN=aldC PE=4 SV=1	265	29.128345	0.00973	77.22	104.95	66.35	0.44	-0.22	Q83CL7
CBU_1098	Q83CL6_COXBU Hypothetical cytosolic protein OS=Coxiella burnetii (strain RSA 493 / Nine Mile phase I) GN=CBU_1098 PE=4 SV=1	274	31.57365	0.009686	10.98	0	0	-10	-10	Q83CL6
CBU_1099	Q83CL5_COXBU Signal peptidase I OS=Coxiella burnetii (strain RSA 493 / Nine Mile phase I) GN=lepB-1 PE=3 SV=1	259	30.070217	0.0099937	8.13	33.13	54.06	2.03	2.73	Q83CL5
CBU_1100	Q83CL4_COXBU Hypothetical exported protein OS=Coxiella burnetii (strain RSA 493 / Nine Mile phase I) GN=CBU_1100 PE=4 SV=2	142	15.366132	0.0103013	182.24	164.61	245.35	-0.15	0.43	Q83CL4
CBU_1111	Q83CK4_COXBU Membrane-bound lytic murein transglycosylase A OS=Coxiella burnetii (strain RSA 493 / Nine Mile phase I) GN=CBU_1111 PE=4 SV=2	404	45.262902	0.0097007	0.74	10.99	16.93	3.88	4.51	Q83CK4
CBU_1116	Q83CJ9_COXBU Alanine dehydrogenase OS=Coxiella burnetii (strain RSA 493 / Nine Mile phase I) GN=ald PE=3 SV=1	369	39.428521	0.0060747	63.61	68.96	111.18	0.12	0.81	Q83CJ9
CBU_1117	Q83CJ8_COXBU Electron transfer flavoprotein alpha-subunit OS=Coxiella burnetii (strain RSA 493 / Nine Mile phase I) GN=etfA PE=4 SV=1	314	34.085515	0.0050337	69	5.65	5.18	-3.61	-3.73	Q83CJ8
CBU_1118	Q83CJ7_COXBU Electron transfer flavoprotein beta-subunit OS=Coxiella burnetii (strain RSA 493 / Nine Mile phase I) GN=etfB PE=4 SV=1	250	27.424032	0.0064048	526	310.08	325.61	-0.76	-0.69	Q83CJ7
CBU_1119	Q83CJ6_COXBU Carboxymethylenebutenolidase OS=Coxiella burnetii (strain RSA 493 / Nine Mile phase I) GN=CBU_1119 PE=4 SV=2	273	30.719743	0.006354	46.29	95.37	115.69	1.04	1.32	Q83CJ6
CBU_1122	Q83CJ3_COXBU Enhanced entry protein OS=Coxiella burnetii (strain RSA 493 / Nine Mile phase I) GN=enhA.3 PE=4 SV=2	253	28.674544	0.0094224	593.5	429.19	263.83	-0.47	-1.17	Q83CJ3
CBU_1123	CBPA_COXBU Curved DNA-binding protein OS=Coxiella burnetii (strain RSA 493 / Nine Mile phase I) GN=cbpA PE=3 SV=1	313	34.895996	0.0076792	74.99	103.04	131.08	0.46	0.81	Q83CJ2
CBU_1124	CBPM_COXBU Chaperone modulatory protein CbpM OS=Coxiella burnetii (strain RSA 493 / Nine Mile phase I) GN=cbpM PE=3 SV=1	106	12.354622	0.0059097	130.59	131.19	61.44	0.01	-1.09	Q83CJ1
CBU_1128	Q83CI7_COXBU IscU OS=Coxiella burnetii (strain RSA 493 / Nine Mile phase I) GN=iscU.1 PE=4 SV=1	119	13.687764	0.0055796	12.64	0	0	-10	-10	Q83CI7
CBU_1129	Q83CI6_COXBU Cysteine desulfurase OS=Coxiella burnetii (strain RSA 493 / Nine Mile phase I) GN=nifS PE=3 SV=1	396	43.040399	0.0075474	85.11	0	0	-10	-10	Q83CI6
CBU_1130	Q83CI5_COXBU Oligopeptide transporter OS=Coxiella burnetii (strain RSA 493 / Nine Mile phase I) GN=CBU_1130 PE=4 SV=2	689	73.766727	0.0101841	13.1	24.05	21.27	0.88	0.7	Q83CI5
CBU_1131	Q83CI4_COXBU rRNA methylase, SpoU family OS=Coxiella burnetii (strain RSA 493 / Nine Mile phase I) GN=CBU_1131 PE=4 SV=1	240	26.766885	0.0087046	13.79	11.1	12.21	-0.31	-0.18	Q83CI4
CBU_1133	Q83CI2_COXBU Myo-inositol-1(Or 4)-monophosphatase OS=Coxiella burnetii (strain RSA 493 / Nine Mile phase I) GN=suhB PE=4 SV=2	267	29.774567	0.0086753	60.86	17.73	41.46	-1.78	-0.55	Q83CI2
CBU_1136	Q83CH9_COXBU Enhanced entry protein enhC, tetratricopeptide repeat family OS=Coxiella burnetii (strain RSA 493 / Nine Mile phase I) GN=CBU_1136 PE=4 SV=1	1044	117.64823	0.0096421	0	41.38	9.67	10	10	Q83CH9
CBU_1137	Q83CH8_COXBU Enhanced entry protein OS=Coxiella burnetii (strain RSA 493 / Nine Mile phase I) GN=enhB.2 PE=4 SV=1	184	20.877827	0.0092173	0	98.09	70.78	10	10	Q83CH8
CBU_1138	Q83CH7_COXBU Enhanced entry protein OS=Coxiella burnetii (strain RSA 493 / Nine Mile phase I) GN=enhA.4 PE=4 SV=1	248	27.712995	0.0090854	0	36.98	36.76	10	10	Q83CH7
CBU_1141	Q83CH4_COXBU Protein-export membrane protein SecF OS=Coxiella burnetii (strain RSA 493 / Nine Mile phase I) GN=secF PE=3 SV=1	304	33.550419	0.0101255	6.93	40.88	38.56	2.56	2.48	Q83CH4
CBU_1142	Q83CH3_COXBU Protein translocase subunit SecD OS=Coxiella burnetii (strain RSA 493 / Nine Mile phase I) GN=secD PE=3 SV=1	622	67.699387	0.009188	100.14	191.22	124.07	0.93	0.31	Q83CH3

CBU_1143	Q83CH2_COXBU Protein translocase subunit OS=Coxiella burnetii (strain RSA 493 / Nine Mile phase I) GN=yajC PE=4 SV=1	116	12.498967	0.009979	9421.72	6215.92	6910.76	-0.6	-0.45	Q83CH2
CBU_1148	Q83CG9_COXBU Transcription-repair coupling factor OS=Coxiella burnetii (strain RSA 493 / Nine Mile phase I) GN=mfd PE=4 SV=1	1157	131.50565	0.0064302	0	3.58	3.66	10	10	Q83CG9
CBU_1169	Q83CE9_COXBU Small heat shock protein OS=Coxiella burnetii (strain RSA 493 / Nine Mile phase I) GN=CBU_1169 PE=3 SV=1	151	17.177817	0.0055669	388.6	505.53	498.12	0.38	0.36	Q83CE9
CBU_1183	Q83CD7_COXBU Glycine-rich RNA-binding protein OS=Coxiella burnetii (strain RSA 493 / Nine Mile phase I) GN=CBU_1183 PE=4 SV=2	117	13.122544	0.0099937	7710.6	606.92	169.76	-3.67	-5.51	Q83CD7
CBU_1185	UVRC_COXBU UvrABC system protein C OS=Coxiella burnetii (strain RSA 493 / Nine Mile phase I) GN=uvrC PE=3 SV=1	609	69.00351	0.0099937	0	0.49	7.49	10	10	Q83CD5
CBU_1188	SYS_COXBU Serine--tRNA ligase OS=Coxiella burnetii (strain RSA 493 / Nine Mile phase I) GN=serS PE=3 SV=1	423	48.086465	0.0057446	9.96	37.07	27.71	1.9	1.48	P39919
CBU_1190	LOLA_COXBU Outer-membrane lipoprotein carrier protein OS=Coxiella burnetii (strain RSA 493 / Nine Mile phase I) GN=lolA PE=1 SV=1	211	23.767668	0.0105063	125.5	180.89	131.17	0.53	0.06	P39917
CBU_1193	TRXB_COXBU Thioredoxin reductase OS=Coxiella burnetii (strain RSA 493 / Nine Mile phase I) GN=trxB PE=3 SV=2	320	34.580485	0.0059351	63.94	72.12	112.95	0.17	0.82	P39916
CBU_1195	IF1_COXBU Translation initiation factor IF-1 OS=Coxiella burnetii (strain RSA 493 / Nine Mile phase I) GN=infA PE=3 SV=1	83	9.3018712	0.0096274	333.54	206.76	215.76	-0.69	-0.63	Q83CD1
CBU_1200	IDH_COXBU Isocitrate dehydrogenase [NADP] OS=Coxiella burnetii (strain RSA 493 / Nine Mile phase I) GN=icd PE=1 SV=1	427	46.588542	0.006062	658.91	435.15	372.89	-0.6	-0.82	Q9ZH99
CBU_1203	Q83CC4_COXBU Ferredoxin-dependent glutamate synthase OS=Coxiella burnetii (strain RSA 493 / Nine Mile phase I) GN=CBU_1203 PE=3 SV=1	550	61.750549	0.0077524	0	6.99	8.88	10	10	Q83CC4
CBU_1204	Q83CC3_COXBU Succinate-semialdehyde dehydrogenase (NADP+) OS=Coxiella burnetii (strain RSA 493 / Nine Mile phase I) GN=CBU_1204 PE=3 SV=2	458	50.038832	0.006522	3.94	131.79	153.56	5.06	5.28	Q83CC3
CBU_1220	PUR7_COXBU Phosphoribosylaminoimidazole-succinocarboxamide synthase OS=Coxiella burnetii (strain RSA 493 / Nine Mile phase I) GN=purC PE=3 SV=1	240	27.443113	0.0063667	86.51	173.83	147.88	1.01	0.77	Q83CA8
CBU_1221	Q83CA7_COXBU Hypothetical membrane spanning protein OS=Coxiella burnetii (strain RSA 493 / Nine Mile phase I) GN=CBU_1221 PE=4 SV=1	197	21.796578	0.0105503	278	405.51	429.74	0.54	0.63	Q83CA7
CBU_1222	DAPA_COXBU 4-hydroxy-tetrahydrodipicolinate synthase OS=Coxiella burnetii (strain RSA 493 / Nine Mile phase I) GN=dapA PE=3 SV=1	289	31.575486	0.0052241	5.21	63.47	83.37	3.61	4	Q83CA6
CBU_1223	Q83CA5_COXBU 2-dehydro-3-deoxygluconokinase OS=Coxiella burnetii (strain RSA 493 / Nine Mile phase I) GN=kdgK PE=4 SV=1	309	34.419622	0.0061255	22.4	76.6	96.94	1.77	2.11	Q83CA5
CBU_1224a	B5QSB9_COXBU Uncharacterized protein OS=Coxiella burnetii (strain RSA 493 / Nine Mile phase I) GN=CBU_1224.1 PE=4 SV=1	65	7.219576	0.0095688	2573.97	2872.26	2469.62	0.16	-0.06	B5QSB9
CBU_1225	Q83CA3_COXBU 1,3,4,6-tetrachloro-1,4-cyclohexadiene hydrolase OS=Coxiella burnetii (strain RSA 493 / Nine Mile phase I) GN=CBU_1225 PE=4 SV=2	302	34.723877	0.0087046	0	28.41	33.42	10	10	Q83CA3
CBU_1226	Q83CA2_COXBU NAD-specific glutamate dehydrogenase OS=Coxiella burnetii (strain RSA 493 / Nine Mile phase I) GN=CBU_1226 PE=4 SV=2	1626	186.46201	0.0066392	108.26	166.86	132.57	0.62	0.29	Q83CA2
CBU_1227	Q83CA1_COXBU Transcriptional regulatory protein OS=Coxiella burnetii (strain RSA 493 / Nine Mile phase I) GN=qseB PE=4 SV=2	232	26.245956	0.0093491	141.38	12.75	21.05	-3.47	-2.75	Q83CA1
CBU_1233	Q83C95_COXBU 5'-nucleotidase OS=Coxiella burnetii (strain RSA 493 / Nine Mile phase I) GN=CBU_1233 PE=4 SV=2	300	33.185037	0.0059351	75.23	35.5	60.78	-1.08	-0.31	Q83C95
CBU_1234	Q83C94_COXBU Hypothetical ATPase OS=Coxiella burnetii (strain RSA 493 / Nine Mile phase I) GN=CBU_1234 PE=4 SV=2	428	50.692593	0.0063794	3.52	0	22.82	-10	2.7	Q83C94
CBU_1235	ORN_COXBU Oligoribonuclease OS=Coxiella burnetii (strain RSA 493 / Nine Mile phase I) GN=orn PE=1 SV=1	183	20.980697	0.0056431	6.58	8.08	10.68	0.3	0.7	Q83C93
CBU_1239	PLSY_COXBU Glycerol-3-phosphate acyltransferase OS=Coxiella burnetii (strain RSA 493 / Nine Mile phase I) GN=plsY PE=3 SV=1	193	20.829728	0.0107847	20.27	242.22	285.12	3.58	3.81	Q83C89
CBU_1241	MDH_COXBU Malate dehydrogenase OS=Coxiella burnetii (strain RSA 493 / Nine Mile phase I) GN=mdh PE=3 SV=1	328	35.429091	0.0049321	369.72	884.02	838.84	1.26	1.18	Q83C87
CBU_1245	DER_COXBU GTPase Der OS=Coxiella burnetii (strain RSA 493 / Nine Mile phase I) GN=der PE=3 SV=1	443	49.658603	0.0095981	23.09	16.7	19.85	-0.47	-0.22	Q83C83
CBU_1247	Q83C81_COXBU Uncharacterized protein OS=Coxiella burnetii (strain RSA 493 / Nine Mile phase I) GN=CBU_1247 PE=4 SV=1	206	23.694378	0.0096714	30.68	0	7.9	-10	-1.96	Q83C81
CBU_1248	SYH_COXBU Histidine--tRNA ligase OS=Coxiella burnetii (strain RSA 493 / Nine Mile phase I) GN=hisS PE=3 SV=1	421	47.761738	0.0057446	5.72	10.54	17.79	0.88	1.64	Q83C80
CBU_1249	Q83C79_COXBU Integral membrane protein OS=Coxiella burnetii (strain RSA 493 / Nine Mile phase I) GN=CBU_1249 PE=4 SV=1	203	22.968232	0.0098911	91.9	8.75	12.83	-3.39	-2.84	Q83C79
CBU_1255	Q83C74_COXBU Dimethylallyltransferase OS=Coxiella burnetii (strain RSA 493 / Nine Mile phase I) GN=CBU_1255 PE=3 SV=1	267	29.657291	0.005186	100.3	29.92	35.37	-1.75	-1.5	Q83C74
CBU_1258	NDK_COXBU Nucleoside diphosphate kinase OS=Coxiella burnetii (strain RSA 493 / Nine Mile phase I) GN=ndk PE=3 SV=1	144	15.826169	0.0063667	137.92	16.44	11.31	-3.07	-3.61	Q83C71
CBU_1260	Y1260_COXBU Uncharacterized protein CBU_1260 OS=Coxiella burnetii (strain RSA 493 / Nine Mile phase I) GN=CBU_1260 PE=4 SV=1	248	26.219533	0.0099058	505.97	374.62	464.78	-0.43	-0.12	Q83C69
CBU_1261	Q83C68_COXBU D-alanyl-D-alanine serine-type carboxypeptidase OS=Coxiella burnetii (strain RSA 493 / Nine Mile phase I) GN=CBU_1261 PE=3 SV=1	418	46.32091	0.0096714	163.41	234.29	153.46	0.52	-0.09	Q83C68
CBU_1266	LIPA_COXBU Lipoyl synthase OS=Coxiella burnetii (strain RSA 493 / Nine Mile phase I) GN=lipA PE=3 SV=1	315	35.563109	0.0081333	9.55	0	0	-10	-10	Q83C63
CBU_1268	Q83C61_COXBU Uncharacterized protein OS=Coxiella burnetii (strain RSA 493 / Nine Mile phase I) GN=CBU_1268 PE=4 SV=2	357	40.527965	0.0084409	39.62	14.92	5.47	-1.41	-2.86	Q83C61
CBU_1269	Q83C60_COXBU Acyl-CoA hydrolase OS=Coxiella burnetii (strain RSA 493 / Nine Mile phase I) GN=CBU_1269 PE=4 SV=1	163	18.597811	0.0100815	0	34.49	91.89	10	10	Q83C60
CBU_1273	Q83C58_COXBU Pyrophosphate--fructose 6-phosphate 1-phosphotransferase OS=Coxiella burnetii (strain RSA 493 / Nine Mile phase I) GN=CBU_1273 PE=4 SV=1	420	46.035482	0.007269	94.57	11.27	1.55	-3.07	-5.93	Q83C58
CBU_1275	Q83C56_COXBU Starvation sensing protein OS=Coxiella burnetii (strain RSA 493 / Nine Mile phase I) GN=rspA PE=4 SV=1	402	45.383853	0.0057065	168.42	162.66	149.84	-0.05	-0.17	Q83C56
CBU_1276	Q83C55_COXBU Short chain dehydrogenase OS=Coxiella burnetii (strain RSA 493 / Nine Mile phase I) GN=CBU_1276 PE=3 SV=2	268	29.112129	0.0065366	295.3	377.57	311.03	0.35	0.07	Q83C55
CBU_1277	Q83C54_COXBU 4-Hydroxy-2-oxoglutarate aldolase OS=Coxiella burnetii (strain RSA 493 / Nine Mile phase I) GN=eda PE=4 SV=2	235	25.713838	0.0085728	28.17	71.77	73.44	1.35	1.38	Q83C54
CBU_1278	Q83C53_COXBU Thiol-disulfide isomerase and thioredoxin OS=Coxiella burnetii (strain RSA 493 / Nine Mile phase I) GN=CBU_1278 PE=4 SV=1	185	20.731256	0.0049702	47.17	54.38	38.72	0.21	-0.28	Q83C53
CBU_1280	Q83C51_COXBU Transcription elongation factor GreA OS=Coxiella burnetii (strain RSA 493 / Nine Mile phase I) GN=greA PE=3 SV=2	173	19.172918	0.0046021	52.18	6.84	3.76	-2.93	-3.79	Q83C51
CBU_1281	Q83C50_COXBU Carbamoyl-phosphate synthase large chain OS=Coxiella burnetii (strain RSA 493 / Nine Mile phase I) GN=carB PE=3 SV=1	1073	118.44551	0.005313	46.27	62.04	64.03	0.42	0.47	Q83C50
CBU_1282	Q83C49_COXBU Carbamoyl-phosphate synthase small chain OS=Coxiella burnetii (strain RSA 493 / Nine Mile phase I) GN=carA PE=3 SV=2	402	43.746511	0.0069321	5.99	13.98	17.01	1.22	1.51	Q83C49

CBU_1289	DNAJ_COXBU Chaperone protein DnaJ OS=Coxiella burnetii (strain RSA 493 / Nine Mile phase I) GN=dnaJ PE=3 SV=2	374	40.812558	0.0082505	96.55	10.28	11.32	-3.23	-3.09	P42381
CBU_1290	DNAK_COXBU Chaperone protein DnaK OS=Coxiella burnetii (strain RSA 493 / Nine Mile phase I) GN=dnaK PE=1 SV=1	656	70.693737	0.0049956	2366.49	1678.73	2494.18	-0.5	0.08	O87712
CBU_1291	Q83C43_COXBU Uncharacterized protein OS=Coxiella burnetii (strain RSA 493 / Nine Mile phase I) GN=CBU_1291 PE=4 SV=1	197	21.565101	0.0095103	10.69	0	0	-10	-10	Q83C43
CBU_1292	Q83C42_COXBU Ankyrin repeat protein OS=Coxiella burnetii (strain RSA 493 / Nine Mile phase I) GN=CBU_1292 PE=4 SV=1	465	52.829776	0.0059731	18.77	0	2.1	-10	-3.16	Q83C42
CBU_1293	GRPE_COXBU Protein GrpE OS=Coxiella burnetii (strain RSA 493 / Nine Mile phase I) GN=grpE PE=3 SV=1	204	22.919666	0.0052622	339.27	127.63	162.8	-1.41	-1.06	Q83C41
CBU_1296	PPNK_COXBU Probable inorganic polyphosphate/ATP-NAD kinase OS=Coxiella burnetii (strain RSA 493 / Nine Mile phase I) GN=ppnK PE=3 SV=1	299	32.853238	0.0051987	18.12	80.15	70.78	2.15	1.97	Q83C38
CBU_1297	Q83C37_COXBU DNA repair protein RecN OS=Coxiella burnetii (strain RSA 493 / Nine Mile phase I) GN=recN PE=3 SV=1	556	63.02782	0.0060493	4.33	0	0	-10	-10	Q83C37
CBU_1302	Q83C32_COXBU Outer membrane lipoprotein OS=Coxiella burnetii (strain RSA 493 / Nine Mile phase I) GN=omlA PE=4 SV=1	126	14.155377	0.0100229	23.88	51.66	41.35	1.11	0.79	Q83C32
CBU_1306	Q83C28_COXBU Peptide methionine sulfoxide reductase MsrA OS=Coxiella burnetii (strain RSA 493 / Nine Mile phase I) GN=msrA PE=3 SV=1	284	32.804022	0.0069028	9.54	16.67	10.32	0.81	0.11	Q83C28
CBU_1320	IHFA_COXBU Integration host factor subunit alpha OS=Coxiella burnetii (strain RSA 493 / Nine Mile phase I) GN=ihfA PE=3 SV=1	103	11.592261	0.010228	742.06	405.03	290.83	-0.87	-1.35	Q83C16
CBU_1321	SYFB_COXBU Phenylalanine--tRNA ligase beta subunit OS=Coxiella burnetii (strain RSA 493 / Nine Mile phase I) GN=pheT PE=3 SV=1	792	88.408299	0.0055796	53.57	104.98	117.99	0.97	1.14	Q83C15
CBU_1322	SYFA_COXBU Phenylalanine--tRNA ligase alpha subunit OS=Coxiella burnetii (strain RSA 493 / Nine Mile phase I) GN=pheS PE=3 SV=1	328	37.808709	0.0074302	22.02	53.22	53.61	1.27	1.28	Q83C14
CBU_1323	RL20_COXBU 50S ribosomal protein L20 OS=Coxiella burnetii (strain RSA 493 / Nine Mile phase I) GN=rplT PE=3 SV=1	119	13.416594	0.0119565	91.03	39.78	38.31	-1.19	-1.25	Q83C13
CBU_1324	RL35_COXBU 50S ribosomal protein L35 OS=Coxiella burnetii (strain RSA 493 / Nine Mile phase I) GN=rpmI PE=3 SV=1	64	7.3701688	0.0124253	333.83	60.1	35.61	-2.47	-3.23	Q83C12
CBU_1325	IF3_COXBU Translation initiation factor IF-3 OS=Coxiella burnetii (strain RSA 493 / Nine Mile phase I) GN=infC PE=3 SV=2	185	21.358562	0.0106382	297.66	185.52	135.52	-0.68	-1.14	Q83C11
CBU_1326	SYT_COXBU Threonine--tRNA ligase OS=Coxiella burnetii (strain RSA 493 / Nine Mile phase I) GN=thrS PE=3 SV=1	651	73.720133	0.0059604	314.32	286.33	295.1	-0.13	-0.09	Q83C10
CBU_1334	Q83C03_COXBU Hypothetical membrane spanning protein OS=Coxiella burnetii (strain RSA 493 / Nine Mile phase I) GN=CBU_1334 PE=4 SV=1	377	42.719492	0.0095981	0.8	1.57	6.91	0.98	3.11	Q83C03
CBU_1337	Q83C00_COXBU DNA polymerase III alpha subunit OS=Coxiella burnetii (strain RSA 493 / Nine Mile phase I) GN=dnaE PE=4 SV=1	1143	128.38163	0.005605	5.27	0	0	-10	-10	Q83C00
CBU_1338	DDL_COXBU D-alanine--D-alanine ligase OS=Coxiella burnetii (strain RSA 493 / Nine Mile phase I) GN=ddl PE=1 SV=1	372	41.17395	0.0056431	23.46	61.24	48.14	1.38	1.04	Q83BZ9
CBU_1341	GUAA_COXBU GMP synthase [glutamine-hydrolyzing] OS=Coxiella burnetii (strain RSA 493 / Nine Mile phase I) GN=guaA PE=1 SV=1	524	58.626653	0.0062778	123.47	59.29	54.06	-1.06	-1.19	Q83BZ6
CBU_1342	Q83BZ5_COXBU Inosine-5'-monophosphate dehydrogenase OS=Coxiella burnetii (strain RSA 493 / Nine Mile phase I) GN=guaB PE=3 SV=1	489	52.721729	0.0081772	311.99	255.34	233.05	-0.29	-0.42	Q83BZ5
CBU_1350	GLMM_COXBU Phosphoglucosamine mutase OS=Coxiella burnetii (strain RSA 493 / Nine Mile phase I) GN=glmM PE=3 SV=1	446	48.110033	0.0064683	20.92	0	0	-10	-10	Q83BY7
CBU_1351	Q83BY6_COXBU Dihydropteroate synthase OS=Coxiella burnetii (strain RSA 493 / Nine Mile phase I) GN=folP PE=1 SV=1	297	33.141136	0.0064429	19.25	2.99	24.12	-2.69	0.33	Q83BY6
CBU_1352	Q83BY5_COXBU ATP-dependent zinc metalloprotease FtsH OS=Coxiella burnetii (strain RSA 493 / Nine Mile phase I) GN=ftsH PE=3 SV=1	647	71.547046	0.0062271	355.33	425.29	390.53	0.26	0.14	Q83BY5
CBU_1372	Q83BW5_COXBU Hypothetical membrane associated protein OS=Coxiella burnetii (strain RSA 493 / Nine Mile phase I) GN=CBU_1372 PE=4 SV=1	193	21.713136	0.0092173	20.27	6.13	20.25	-1.72	0	Q83BW5
CBU_1380	Q83BV7_COXBU DoxD-like family OS=Coxiella burnetii (strain RSA 493 / Nine Mile phase I) GN=CBU_1380 PE=4 SV=1	281	31.161783	0.0088071	78.17	143.2	139.05	0.87	0.83	Q83BV7
CBU_1382	UPPS_COXBU Ditrans,polycis-undecaprenyl-diphosphate synthase ((2E,6E)-farnesyl-diphosphate specific) OS=Coxiella burnetii (strain RSA 493 / Nine Mile phase I) GN=uppS PE=3 SV=1	237	27.594343	0.0093491	92.69	0	0	-10	-10	Q83BV5
CBU_1383	RRF_COXBU Ribosome-recycling factor OS=Coxiella burnetii (strain RSA 493 / Nine Mile phase I) GN=frr PE=3 SV=1	185	20.914061	0.0064175	326.94	155.13	221.77	-1.08	-0.56	Q83BV4
CBU_1384	PYRH_COXBU Uridylate kinase OS=Coxiella burnetii (strain RSA 493 / Nine Mile phase I) GN=pyrH PE=3 SV=1	243	26.326714	0.0093491	170.89	191.16	200.99	0.16	0.23	Q83BV3
CBU_1385	EFTS_COXBU Elongation factor Ts OS=Coxiella burnetii (strain RSA 493 / Nine Mile phase I) GN=tsf PE=1 SV=1	296	31.781682	0.0057827	431.04	226.9	248.61	-0.93	-0.79	Q9X5U9
CBU_1386	RS2_COXBU 30S ribosomal protein S2 OS=Coxiella burnetii (strain RSA 493 / Nine Mile phase I) GN=rpsB PE=3 SV=1	313	35.277665	0.0092466	523.96	406.47	412.99	-0.37	-0.34	Q9X5U8
CBU_1388	Q83BV1_COXBU Methionine aminopeptidase OS=Coxiella burnetii (strain RSA 493 / Nine Mile phase I) GN=map PE=3 SV=2	270	29.933503	0.0073716	117.02	89.86	83.21	-0.38	-0.49	Q83BV1
CBU_1394	Q83BU9_COXBU Enhanced entry protein OS=Coxiella burnetii (strain RSA 493 / Nine Mile phase I) GN=enhA.5 PE=4 SV=1	175	19.348891	0.0104917	0	211.34	240.02	10	10	Q83BU9
CBU_1396	SUCD_COXBU Succinyl-CoA ligase [ADP-forming] subunit alpha OS=Coxiella burnetii (strain RSA 493 / Nine Mile phase I) GN=sucD PE=3 SV=2	294	30.629455	0.0053003	800.39	466.96	437.47	-0.78	-0.87	P53591
CBU_1397	SUCC_COXBU Succinyl-CoA ligase [ADP-forming] subunit beta OS=Coxiella burnetii (strain RSA 493 / Nine Mile phase I) GN=sucC PE=3 SV=2	390	42.288279	0.0053765	1460.59	864.11	863.28	-0.76	-0.76	P53592
CBU_1398	Q83BU7_COXBU Dihydrolipoamide succinyltransferase component (E2) of 2-oxoglutarate dehydrogenase complex OS=Coxiella burnetii (strain RSA 493 / Nine Mile phase I) GN=sucB PE=3 SV=1	405	45.833146	0.0054146	878.22	978.21	887.58	0.16	0.02	Q83BU7
CBU_1399	ODO1_COXBU 2-oxoglutarate dehydrogenase E1 component OS=Coxiella burnetii (strain RSA 493 / Nine Mile phase I) GN=sucA PE=3 SV=3	934	106.63743	0.0073716	413.68	172.01	139.1	-1.27	-1.57	P51056
CBU_1400	DHSB_COXBU Succinate dehydrogenase iron-sulfur subunit OS=Coxiella burnetii (strain RSA 493 / Nine Mile phase I) GN=sdhB PE=3 SV=1	235	26.811323	0.0066831	412.32	376.45	375.49	-0.13	-0.14	P51053
CBU_1401	DHSA_COXBU Succinate dehydrogenase flavoprotein subunit OS=Coxiella burnetii (strain RSA 493 / Nine Mile phase I) GN=sdhA PE=3 SV=1	587	65.37886	0.0067271	462.39	334.69	353.34	-0.47	-0.39	P51054
CBU_1403	DHSC_COXBU Succinate dehydrogenase cytochrome b556 subunit OS=Coxiella burnetii (strain RSA 493 / Nine Mile phase I) GN=sdhC PE=3 SV=1	125	14.283583	0.0097739	7.22	14.2	18.23	0.98	1.34	P51055
CBU_1404	Q83BU6_COXBU Hypothetical exported protein OS=Coxiella burnetii (strain RSA 493 / Nine Mile phase I) GN=CBU_1404 PE=4 SV=1	110	12.230027	0.0088218	54.71	69.93	26.64	0.35	-1.04	Q83BU6
CBU_1410	CISY_COXBU Citrate synthase OS=Coxiella burnetii (strain RSA 493 / Nine Mile phase I) GN=gltA PE=3 SV=1	430	48.58344	0.0073716	62.28	89.45	105.25	0.52	0.76	P18789
CBU_1415	Q83BT7_COXBU Thiamine-monophosphate kinase OS=Coxiella burnetii (strain RSA 493 / Nine Mile phase I) GN=thiL PE=3 SV=1	338	37.202387	0.0058208	2.67	42.02	92.48	3.98	5.11	Q83BT7

CBU_1417	NUSB_COXBU N utilization substance protein B homolog OS=Coxiella burnetii (strain RSA 493 / Nine Mile phase I) GN=nusB PE=3 SV=1	138	15.845299	0.0056177	148.28	107.2	153.37	-0.47	0.05	Q83BT5
CBU_1418	NRDR_COXBU Transcriptional repressor NrdR OS=Coxiella burnetii (strain RSA 493 / Nine Mile phase I) GN=nrdR PE=3 SV=1	157	18.54964	0.0090269	24.92	24.5	45.63	-0.02	0.87	Q83BT4
CBU_1419	GLYA_COXBU Serine hydroxymethyltransferase OS=Coxiella burnetii (strain RSA 493 / Nine Mile phase I) GN=glyA PE=3 SV=1	419	45.754246	0.0061636	39.5	67.79	74.6	0.78	0.92	Q83BT3
CBU_1422	Q83BT0_COXBU DNA repair protein radA OS=Coxiella burnetii (strain RSA 493 / Nine Mile phase I) GN=radA PE=3 SV=1	451	48.730655	0.0077524	258.21	26.24	90.97	-3.3	-1.51	Q83BT0
CBU_1424	Q83BS8_COXBU Delta-aminolevulinic acid dehydratase OS=Coxiella burnetii (strain RSA 493 / Nine Mile phase I) GN=hemB PE=3 SV=1	334	36.843108	0.0073423	104.51	22.15	26.32	-2.24	-1.99	Q83BS8
CBU_1425	Q83BS7_COXBU 17 kDa common-antigen OS=Coxiella burnetii (strain RSA 493 / Nine Mile phase I) GN=CBU_1425 PE=4 SV=1	150	15.91213	0.0101108	816.48	1262.4	1002.87	0.63	0.3	Q83BS7
CBU_1429	Q83BS3_COXBU Hypothetical membrane spanning protein OS=Coxiella burnetii (strain RSA 493 / Nine Mile phase I) GN=CBU_1429 PE=4 SV=1	181	20.239256	0.0095688	0	1.63	7.2	10	10	Q83BS3
CBU_1432	IF2_COXBU Translation initiation factor IF-2 OS=Coxiella burnetii (strain RSA 493 / Nine Mile phase I) GN=infB PE=3 SV=1	803	88.412887	0.0064937	108.3	103.54	139.49	-0.06	0.37	Q83BS1
CBU_1433	NUSA_COXBU Transcription termination/antitermination protein NusA OS=Coxiella burnetii (strain RSA 493 / Nine Mile phase I) GN=nusA PE=1 SV=1	503	56.221912	0.004437	938.04	644.69	682.94	-0.54	-0.46	Q83BS0
CBU_1440	NUOI_COXBU NADH-quinone oxidoreductase subunit I OS=Coxiella burnetii (strain RSA 493 / Nine Mile phase I) GN=nuoI PE=3 SV=1	163	18.966696	0.0085142	16.61	3.63	9.99	-2.19	-0.73	Q83BR3
CBU_1442	Q83BR1_COXBU NADH-quinone oxidoreductase chain G OS=Coxiella burnetii (strain RSA 493 / Nine Mile phase I) GN=nuoG PE=4 SV=1	787	87.62341	0.0054146	186.21	162.41	208.11	-0.2	0.16	Q83BR1
CBU_1443	Q83BR0_COXBU NADH-quinone oxidoreductase chain F OS=Coxiella burnetii (strain RSA 493 / Nine Mile phase I) GN=nuoF PE=4 SV=1	422	46.438238	0.0065513	17.83	34.36	30.09	0.95	0.76	Q83BR0
CBU_1444	Q83BQ9_COXBU NADH-quinone oxidoreductase chain E OS=Coxiella burnetii (strain RSA 493 / Nine Mile phase I) GN=nuoE PE=3 SV=1	174	19.830811	0.0046655	79.55	59.52	56.14	-0.42	-0.5	Q83BQ9
CBU_1445	NUOD_COXBU NADH-quinone oxidoreductase subunit D OS=Coxiella burnetii (strain RSA 493 / Nine Mile phase I) GN=nuoD PE=3 SV=1	417	48.070345	0.0062651	33.19	24.83	64.03	-0.42	0.95	Q83BQ8
CBU_1446	NUOC_COXBU NADH-quinone oxidoreductase subunit C OS=Coxiella burnetii (strain RSA 493 / Nine Mile phase I) GN=nuoC PE=3 SV=1	227	26.273438	0.0049067	174.98	72.99	110.45	-1.26	-0.66	Q83BQ7
CBU_1447	NUOB_COXBU NADH-quinone oxidoreductase subunit B OS=Coxiella burnetii (strain RSA 493 / Nine Mile phase I) GN=nuoB PE=3 SV=1	161	18.129867	0.0085728	114.01	88.21	58.65	-0.37	-0.96	Q83BQ6
CBU_1450	TPIS_COXBU Triosephosphate isomerase OS=Coxiella burnetii (strain RSA 493 / Nine Mile phase I) GN=tpiA PE=3 SV=1	255	28.136198	0.0062017	134.53	39.45	89.38	-1.77	-0.59	Q83BQ3
CBU_1451	Q83BQ2_COXBU Peptidyl-prolyl cis-trans isomerase OS=Coxiella burnetii (strain RSA 493 / Nine Mile phase I) GN=CBU_1451 PE=4 SV=1	522	58.701026	0.0096714	68.6	47.05	39.92	-0.54	-0.78	Q83BQ2
CBU_1464	Q83BN9_COXBU DNA-binding protein HU OS=Coxiella burnetii (strain RSA 493 / Nine Mile phase I) GN=hupB PE=3 SV=1	94	9.985234	0.0100229	1770.27	664.14	290.97	-1.41	-2.6	Q83BN9
CBU_1470	Q83BN3_COXBU Cell shape-determining protein MreC OS=Coxiella burnetii (strain RSA 493 / Nine Mile phase I) GN=mreC PE=3 SV=1	292	32.09963	0.0099204	35.04	1.01	3.35	-5.11	-3.39	Q83BN3
CBU_1471	Q83BN2_COXBU Rod shape-determining protein OS=Coxiella burnetii (strain RSA 493 / Nine Mile phase I) GN=mreB PE=4 SV=1	352	37.623679	0.0052749	796.74	552.24	509.69	-0.53	-0.64	Q83BN2
CBU_1474	GATA_COXBU Glutamyl-tRNA(Gln) amidotransferase subunit A OS=Coxiella burnetii (strain RSA 493 / Nine Mile phase I) GN=gatA PE=3 SV=1	483	52.510822	0.0061382	116.5	193.57	179.99	0.73	0.63	Q83BM9
CBU_1475	GATB_COXBU Aspartyl/glutamyl-tRNA(Asn/Gln) amidotransferase subunit B OS=Coxiella burnetii (strain RSA 493 / Nine Mile phase I) GN=gatB PE=3 SV=1	477	53.402502	0.0053257	39.11	102.97	130.38	1.4	1.74	Q83BM8
CBU_1476	Q83BM7_COXBU Hydrogen peroxide-inducible genes activator OS=Coxiella burnetii (strain RSA 493 / Nine Mile phase I) GN=oxyR PE=4 SV=1	311	34.584957	0.009437	37.74	19.03	8.38	-0.99	-2.17	Q83BM7
CBU_1477	Q83BM6_COXBU Peroxiredoxin OS=Coxiella burnetii (strain RSA 493 / Nine Mile phase I) GN=CBU_1477 PE=4 SV=1	179	20.487417	0.0046401	73.97	0	0	-10	-10	Q83BM6
CBU_1482	Q820W1_COXBU Membrane protease family, stomatin/prohibitin homolog OS=Coxiella burnetii (strain RSA 493 / Nine Mile phase I) GN=CBU_1482 PE=4 SV=1	249	28.351339	0.0054399	201.82	739.09	751.91	1.87	1.9	Q820W1
CBU_1487	SYC_COXBU Cysteine--tRNA ligase OS=Coxiella burnetii (strain RSA 493 / Nine Mile phase I) GN=cysS PE=1 SV=2	460	52.988172	0.0061128	0.65	1.29	3.54	0.98	2.44	Q83BL7
CBU_1488	SYE2_COXBU Glutamate--tRNA ligase 2 OS=Coxiella burnetii (strain RSA 493 / Nine Mile phase I) GN=gltX2 PE=3 SV=2	465	52.711832	0.0057827	20.06	80.81	52.52	2.01	1.39	Q83BL6
CBU_1494	PDXJ_COXBU Pyridoxine 5'-phosphate synthase OS=Coxiella burnetii (strain RSA 493 / Nine Mile phase I) GN=pdxJ PE=3 SV=1	240	25.978534	0.0054907	5.02	235.47	301.19	5.55	5.91	Q83BL1
CBU_1503	RNC_COXBU Ribonuclease 3 OS=Coxiella burnetii (strain RSA 493 / Nine Mile phase I) GN=rnc PE=3 SV=2	233	26.164528	0.0082358	10.33	11.43	19.56	0.15	0.92	P51837
CBU_1504	Q83BK4_COXBU Signal peptidase I OS=Coxiella burnetii (strain RSA 493 / Nine Mile phase I) GN=lepB-2 PE=3 SV=1	256	29.638914	0.0101255	8.23	0	0	-10	-10	Q83BK4
CBU_1505	LEPA_COXBU Elongation factor 4 OS=Coxiella burnetii (strain RSA 493 / Nine Mile phase I) GN=lepA PE=3 SV=2	602	66.421186	0.0066685	2.5	0.49	2.16	-2.35	-0.21	Q83BK3
CBU_1508	Q83BK0_COXBU Hypothetical ATPase OS=Coxiella burnetii (strain RSA 493 / Nine Mile phase I) GN=CBU_1508 PE=4 SV=1	543	62.725011	0.0092173	4.99	10.35	14.39	1.05	1.53	Q83BK0
CBU_1510	ACCA_COXBU Acetyl-coenzyme A carboxylase carboxyl transferase subunit alpha OS=Coxiella burnetii (strain RSA 493 / Nine Mile phase I) GN=accA PE=3 SV=1	316	35.337375	0.0062144	88.56	146.06	149.41	0.72	0.75	Q83BJ8
CBU_1513	Q83BJ5_COXBU Short chain dehydrogenase OS=Coxiella burnetii (strain RSA 493 / Nine Mile phase I) GN=CBU_1513 PE=3 SV=1	258	28.229808	0.0082212	14	47.02	47.96	1.75	1.78	Q83BJ5
CBU_1517	Q83BJ1_COXBU tRNA (cytidine(34)-2'-O)-methyltransferase OS=Coxiella burnetii (strain RSA 493 / Nine Mile phase I) GN=trmL PE=3 SV=1	152	17.39588	0.0074302	0	48.66	25.71	10	10	Q83BJ1
CBU_1518	GPDA_COXBU Glycerol-3-phosphate dehydrogenase [NAD(P)+] OS=Coxiella burnetii (strain RSA 493 / Nine Mile phase I) GN=gpsA PE=1 SV=1	332	36.026905	0.0065659	95.17	249.53	241.26	1.39	1.34	Q83BJ0
CBU_1519	SECB_COXBU Protein-export protein SecB OS=Coxiella burnetii (strain RSA 493 / Nine Mile phase I) GN=secB PE=3 SV=1	161	18.346964	0.0043862	295.31	391.44	400.44	0.41	0.44	Q83BI9
CBU_1520	Q83BI8_COXBU Glutaredoxin OS=Coxiella burnetii (strain RSA 493 / Nine Mile phase I) GN=grxC PE=4 SV=1	85	9.8821102	0.0066831	38.94	62.66	42.14	0.69	0.11	Q83BI8
CBU_1521	Q83BI7_COXBU Rhodanese-related sulfurtransferase OS=Coxiella burnetii (strain RSA 493 / Nine Mile phase I) GN=CBU_1521 PE=4 SV=1	144	16.172714	0.0102427	2.09	32.87	52.01	3.98	4.64	Q83BI7
CBU_1536	GPMI_COXBU 2,3-bisphosphoglycerate-independent phosphoglycerate mutase OS=Coxiella burnetii (strain RSA 493 / Nine Mile phase I) GN=gpmI PE=3 SV=1	519	57.456232	0.0055542	15.07	20.52	28.23	0.45	0.91	Q83BH2
CBU_1538	Q83BH0_COXBU Carboxy-terminal processing protease OS=Coxiella burnetii (strain RSA 493 / Nine Mile phase I) GN=CBU_1538 PE=3 SV=1	456	49.617492	0.0092612	69.95	76.56	59.27	0.13	-0.24	Q83BH0

CBU_1547	TYSY_COXBU Thymidylate synthase OS=Coxiella burnetii (strain RSA 493 / Nine Mile phase I) GN=thyA PE=3 SV=1	264	30.401396	0.0068442	20.52	0	0	-10	-10	Q83BG2
CBU_1550	Q83BF9_COXBU Phosphoenolpyruvate-protein phosphotransferase OS=Coxiella burnetii (strain RSA 493 / Nine Mile phase I) GN=ptsP PE=1 SV=2	766	84.857426	0.0049829	0.39	25.49	35.71	6.02	6.51	Q83BF9
CBU_1553	Q83BF6_COXBU Ribonucleoside-diphosphate reductase OS=Coxiella burnetii (strain RSA 493 / Nine Mile phase I) GN=nrdA PE=3 SV=1	941	106.36092	0.0061128	146.46	0	0	-10	-10	Q83BF6
CBU_1559	Q83BF1_COXBU Hypothetical membrane spanning protein OS=Coxiella burnetii (strain RSA 493 / Nine Mile phase I) GN=CBU_1559 PE=4 SV=1	209	23.823298	0.0105503	28.8	77.86	52.97	1.44	0.88	Q83BF1
CBU_1561	Q83BE9_COXBU Hypothetical membrane spanning protein OS=Coxiella burnetii (strain RSA 493 / Nine Mile phase I) GN=CBU_1561 PE=4 SV=2	262	29.591312	0.009979	0	4.52	9.94	10	10	Q83BE9
CBU_1565	SYD_COXBU Aspartate--tRNA ligase OS=Coxiella burnetii (strain RSA 493 / Nine Mile phase I) GN=aspS PE=3 SV=1	590	66.69469	0.0059604	156.07	177.02	176.6	0.18	0.18	Q83BE5
CBU_1566	Y1566_COXBU Probable transcriptional regulatory protein CBU_1566 OS=Coxiella burnetii (strain RSA 493 / Nine Mile phase I) GN=CBU_1566 PE=1 SV=1	244	26.537302	0.004729	44.4	33.95	50.71	-0.39	0.19	Q83BE4
CBU_1568	RUVA_COXBU Holliday junction ATP-dependent DNA helicase RuvA OS=Coxiella burnetii (strain RSA 493 / Nine Mile phase I) GN=ruvA PE=3 SV=1	200	22.192856	0.0065073	34.61	0	0	-10	-10	Q83BE2
CBU_1570	RUVB_COXBU Holliday junction ATP-dependent DNA helicase RuvB OS=Coxiella burnetii (strain RSA 493 / Nine Mile phase I) GN=ruvB PE=3 SV=1	351	39.1091	0.0056685	85.73	3.37	4.64	-4.67	-4.21	Q83BE0
CBU_1573	Q83BD8_COXBU Transporter, MFS superfamily OS=Coxiella burnetii (strain RSA 493 / Nine Mile phase I) GN=CBU_1573 PE=4 SV=1	411	44.684366	0.0089536	0	4.32	0.79	10	10	Q83BD8
CBU_1574	Q83BD7_COXBU TolQ OS=Coxiella burnetii (strain RSA 493 / Nine Mile phase I) GN=tolQ PE=3 SV=1	237	26.603168	0.0102134	187.91	98.62	97.55	-0.93	-0.95	Q83BD7
CBU_1575	Q83BD6_COXBU TolR OS=Coxiella burnetii (strain RSA 493 / Nine Mile phase I) GN=tolR PE=3 SV=1	147	16.19063	0.0096128	24.56	2.01	2.22	-3.61	-3.47	Q83BD6
CBU_1577	Q83BD4_COXBU Hypothetical exported protein OS=Coxiella burnetii (strain RSA 493 / Nine Mile phase I) GN=CBU_1577 PE=4 SV=1	314	35.194358	0.0064429	7.67	0	0	-10	-10	Q83BD4
CBU_1579	Q83BD2_COXBU Trp repressor binding protein OS=Coxiella burnetii (strain RSA 493 / Nine Mile phase I) GN=CBU_1579 PE=3 SV=1	197	21.124748	0.0073423	16.8	3	26.45	-2.48	0.65	Q83BD2
CBU_1589	Q83BC2_COXBU Hypothetical membrane associated protein OS=Coxiella burnetii (strain RSA 493 / Nine Mile phase I) GN=CBU_1589 PE=4 SV=1	102	11.046664	0.0082651	11.8	321.98	402.22	4.77	5.09	Q83BC2
CBU_1593	RS21_COXBU 30S ribosomal protein S21 OS=Coxiella burnetii (strain RSA 493 / Nine Mile phase I) GN=rpsU PE=3 SV=1	74	8.8817855	0.0106968	105.73	63.97	61.6	-0.72	-0.78	Q83BB9
CBU_1596	RPOD_COXBU RNA polymerase sigma factor RpoD OS=Coxiella burnetii (strain RSA 493 / Nine Mile phase I) GN=rpoD PE=1 SV=1	698	79.485796	0.0095396	93.12	168.28	186.13	0.85	1	Q83BB6
CBU_1602	RIMK_COXBU Ribosomal protein S6 modification protein OS=Coxiella burnetii (strain RSA 493 / Nine Mile phase I) GN=rimK PE=3 SV=1	301	33.152845	0.0101401	0	10.81	28.13	10	10	Q83BB0
CBU_1603	Q83BA9_COXBU Succinylglutamate desuccinylase/aspartoacylase family protein OS=Coxiella burnetii (strain RSA 493 / Nine Mile phase I) GN=CBU_1603 PE=3 SV=1	342	38.126837	0.0100522	0	7.79	8.57	10	10	Q83BA9
CBU_1622	Q83B91_COXBU IcmB OS=Coxiella burnetii (strain RSA 493 / Nine Mile phase I) GN=icmB PE=4 SV=1	1003	112.35424	0.0059478	112.51	82.3	99.01	-0.45	-0.18	Q83B91
CBU_1623	Q83B90_COXBU IcmJ OS=Coxiella burnetii (strain RSA 493 / Nine Mile phase I) GN=icmJ PE=4 SV=1	212	23.806963	0.0070054	12.77	0	10.75	-10	-0.25	Q83B90
CBU_1626	Q83B87_COXBU IcmG OS=Coxiella burnetii (strain RSA 493 / Nine Mile phase I) GN=icmG PE=4 SV=1	244	26.525563	0.0053638	87.56	2.43	10.68	-5.17	-3.04	Q83B87
CBU_1627	Q83B86_COXBU IcmE OS=Coxiella burnetii (strain RSA 493 / Nine Mile phase I) GN=icmE PE=4 SV=2	1039	106.43436	0.0093345	200.42	257.43	361.02	0.36	0.85	Q83B86
CBU_1628	Q83B85_COXBU IcmK OS=Coxiella burnetii (strain RSA 493 / Nine Mile phase I) GN=icmK PE=4 SV=2	351	37.389567	0.0093345	125.17	139.09	148.43	0.15	0.25	Q83B85
CBU_1629	Q83B84_COXBU IcmL OS=Coxiella burnetii (strain RSA 493 / Nine Mile phase I) GN=icmL.1 PE=4 SV=1	218	24.928832	0.0085288	117.33	65.15	41.82	-0.85	-1.49	Q83B84
CBU_1630	Q83B83_COXBU IcmL OS=Coxiella burnetii (strain RSA 493 / Nine Mile phase I) GN=icmL.2 PE=4 SV=1	207	23.081166	0.00973	219.51	138.65	117.97	-0.66	-0.9	Q83B83
CBU_1631	Q83B82_COXBU IcmN protein, OmpA family OS=Coxiella burnetii (strain RSA 493 / Nine Mile phase I) GN=icmN PE=4 SV=1	212	23.882443	0.0103159	58.2	94.9	105.98	0.71	0.86	Q83B82
CBU_1632	Q83B81_COXBU IcmO OS=Coxiella burnetii (strain RSA 493 / Nine Mile phase I) GN=icmO PE=4 SV=1	792	89.215124	0.0064683	41.79	0	0	-10	-10	Q83B81
CBU_1633	Q83B80_COXBU IcmP OS=Coxiella burnetii (strain RSA 493 / Nine Mile phase I) GN=icmP PE=4 SV=1	387	44.328786	0.0095981	22.55	0	0	-10	-10	Q83B80
CBU_1634	Q83B79_COXBU IcmQ OS=Coxiella burnetii (strain RSA 493 / Nine Mile phase I) GN=icmQ PE=4 SV=1	238	27.14207	0.0079429	50.57	0	0	-10	-10	Q83B79
CBU_1642	Q83B73_COXBU IcmS OS=Coxiella burnetii (strain RSA 493 / Nine Mile phase I) GN=icmS PE=4 SV=1	112	12.358379	0.004894	386.89	23.78	8.72	-4.02	-5.47	Q83B73
CBU_1643	Q83B72_COXBU DotD OS=Coxiella burnetii (strain RSA 493 / Nine Mile phase I) GN=dotD PE=4 SV=2	169	18.650139	0.0104185	717.56	521.72	418.09	-0.46	-0.78	Q83B72
CBU_1644	Q83B71_COXBU DotC OS=Coxiella burnetii (strain RSA 493 / Nine Mile phase I) GN=dotC PE=4 SV=1	274	30.236823	0.0089976	135.08	93.95	103.39	-0.52	-0.39	Q83B71
CBU_1645	Q83B70_COXBU ATP-binding protein OS=Coxiella burnetii (strain RSA 493 / Nine Mile phase I) GN=dotB PE=4 SV=1	372	41.29275	0.0060366	12.94	38.97	24.51	1.59	0.92	Q83B70
CBU_1648	Q83B67_COXBU DotA OS=Coxiella burnetii (strain RSA 493 / Nine Mile phase I) GN=dotaA PE=4 SV=2	814	86.793837	0.0052749	249.53	505.97	543.61	1.02	1.12	Q83B67
CBU_1650	Q83B65_COXBU IcmW OS=Coxiella burnetii (strain RSA 493 / Nine Mile phase I) GN=icmW PE=4 SV=1	149	16.942813	0.0052749	66.65	3.97	0	-4.07	-10	Q83B65
CBU_1651	Q83B64_COXBU Hypothetical membrane associated protein OS=Coxiella burnetii (strain RSA 493 / Nine Mile phase I) GN=CBU_1651 PE=4 SV=1	152	15.863697	0.0064556	47.51	27.25	2.14	-0.8	-4.47	Q83B64
CBU_1652	Q83B63_COXBU IcmX OS=Coxiella burnetii (strain RSA 493 / Nine Mile phase I) GN=icmX PE=4 SV=1	376	41.308437	0.0059985	44.82	92.85	77.94	1.05	0.8	Q83B63
CBU_1664	Q83B51_COXBU CBS domain containing protein OS=Coxiella burnetii (strain RSA 493 / Nine Mile phase I) GN=CBU_1664 PE=4 SV=1	197	22.28787	0.0085874	128.31	46.56	84.29	-1.46	-0.61	Q83B51
CBU_1671	SURE_COXBU 5'-nucleotidase SurE OS=Coxiella burnetii (strain RSA 493 / Nine Mile phase I) GN=surE PE=1 SV=1	258	28.030433	0.0052749	45.49	50.46	25.24	0.15	-0.85	Q9KI21
CBU_1674	ENO_COXBU Enolase OS=Coxiella burnetii (strain RSA 493 / Nine Mile phase I) GN=eno PE=1 SV=1	428	46.579683	0.0045386	35.86	120.98	120.2	1.75	1.75	Q83B44
CBU_1675	KDSA_COXBU 2-dehydro-3-deoxyphosphooctonate aldolase OS=Coxiella burnetii (strain RSA 493 / Nine Mile phase I) GN=kdsA PE=3 SV=1	280	30.469753	0.0059858	68.78	116.24	122.1	0.76	0.83	Q83B43
CBU_1677	Q83B41_COXBU Hypothetical cytosolic protein OS=Coxiella burnetii (strain RSA 493 / Nine Mile phase I) GN=CBU_1677 PE=4 SV=1	144	17.297966	0.0062144	0	384.23	368.57	10	10	Q83B41
CBU_1678	Q83B40_COXBU Spermidine N1-acetyltransferase OS=Coxiella burnetii (strain RSA 493 / Nine Mile phase I) GN=speG PE=1 SV=1	170	20.045406	0.0058081	12.39	10.44	55.54	-0.25	2.16	Q83B40
CBU_1682	PYRG_COXBU CTP synthase OS=Coxiella burnetii (strain RSA 493 / Nine Mile phase I) GN=pyrG PE=3 SV=1	555	61.168124	0.0058208	18.43	0	3.52	-10	-2.39	Q83B36

CBU_1689	Q83B30_COXBU Iron-sulfur cluster assembly/repair protein OS=Coxiella burnetii (strain RSA 493 / Nine Mile phase I) GN=apbC PE=4 SV=1	306	32.498449	0.0089976	39.34	0	0	-10	-10	Q83B30
CBU_1695	SYM_COXBU Methionine--tRNA ligase OS=Coxiella burnetii (strain RSA 493 / Nine Mile phase I) GN=metG PE=3 SV=1	546	62.185577	0.0062397	10.47	3.79	9.54	-1.46	-0.13	Q83B24
CBU_1703	Q83B17_COXBU Dihydroorotase OS=Coxiella burnetii (strain RSA 493 / Nine Mile phase I) GN=pyrC PE=3 SV=1	351	38.997261	0.0063794	2.57	12.64	17.63	2.3	2.78	Q83B17
CBU_1705	Q83B15_COXBU Uncharacterized protein OS=Coxiella burnetii (strain RSA 493 / Nine Mile phase I) GN=CBU_1705 PE=4 SV=2	129	14.561808	0.0099937	30.32	0	7.57	-10	-2	Q83B15
CBU_1706	Q83B14_COXBU Thioredoxin peroxidase OS=Coxiella burnetii (strain RSA 493 / Nine Mile phase I) GN=CBU_1706 PE=4 SV=1	200	21.818244	0.0049448	1513.6	1529.67	1196.61	0.02	-0.34	Q83B14
CBU_1708	SODF_COXBU Superoxide dismutase [Fe] OS=Coxiella burnetii (strain RSA 493 / Nine Mile phase I) GN=sodB PE=1 SV=1	193	22.241852	0.0062144	293.12	464.51	367.79	0.66	0.33	P19685
CBU_1709	DAPB_COXBU 4-hydroxy-tetrahydrodipicolinate reductase OS=Coxiella burnetii (strain RSA 493 / Nine Mile phase I) GN=dapB PE=3 SV=2	239	26.197505	0.0073716	11.33	32.19	24.52	1.51	1.11	P24703
CBU_1713	GCSPB_COXBU Probable glycine dehydrogenase [decarboxylating] subunit 2 OS=Coxiella burnetii (strain RSA 493 / Nine Mile phase I) GN=gcvPB PE=3 SV=1	491	54.566255	0.0085435	126.25	57.85	50.4	-1.13	-1.32	Q83B09
CBU_1714	GCSPA_COXBU Probable glycine dehydrogenase [decarboxylating] subunit 1 OS=Coxiella burnetii (strain RSA 493 / Nine Mile phase I) GN=gcvPA PE=3 SV=1	446	48.415948	0.0055542	112	12.6	57.68	-3.15	-0.96	Q83B08
CBU_1715	GCSH_COXBU Glycine cleavage system H protein OS=Coxiella burnetii (strain RSA 493 / Nine Mile phase I) GN=gcvH PE=3 SV=1	130	14.566993	0.0038784	203.7	229.87	180.34	0.17	-0.18	Q83B07
CBU_1716	GCST_COXBU Aminomethyltransferase OS=Coxiella burnetii (strain RSA 493 / Nine Mile phase I) GN=gcvT PE=3 SV=1	363	40.250961	0.0066685	84.55	123.89	128.27	0.55	0.6	Q83B06
CBU_1718	CH60_COXBU 60 kDa chaperonin OS=Coxiella burnetii (strain RSA 493 / Nine Mile phase I) GN=groL PE=1 SV=1	552	58.229346	0.0049956	20209.21	19980.66	20316.32	-0.02	0.01	P19421
CBU_1719	CH10_COXBU 10 kDa chaperonin OS=Coxiella burnetii (strain RSA 493 / Nine Mile phase I) GN=groS PE=3 SV=1	96	10.478531	0.0050337	1667.56	1729.02	1336.35	0.05	-0.32	P19422
CBU_1720	Q83B05_COXBU Aconitate hydratase OS=Coxiella burnetii (strain RSA 493 / Nine Mile phase I) GN=acnA PE=4 SV=2	917	101.30746	0.0057065	344.23	283.94	303.95	-0.28	-0.18	Q83B05
CBU_1725	Q83B00_COXBU Biotin carboxyl carrier protein of acetyl-CoA carboxylase OS=Coxiella burnetii (strain RSA 493 / Nine Mile phase I) GN=accB PE=4 SV=1	154	16.921792	0.0048179	201.26	391.94	336.18	0.96	0.74	Q83B00
CBU_1726	Q83AZ9_COXBU Biotin carboxylase OS=Coxiella burnetii (strain RSA 493 / Nine Mile phase I) GN=accC PE=4 SV=1	448	49.222998	0.0064048	14.78	25.1	30.53	0.76	1.05	Q83AZ9
CBU_1727	Q83AZ8_COXBU Ornithine cyclodeaminase OS=Coxiella burnetii (strain RSA 493 / Nine Mile phase I) GN=arcB PE=4 SV=1	336	37.889855	0.0054019	165.68	178.76	219.01	0.11	0.4	Q83AZ8
CBU_1729	HEM6_COXBU Coproporphyrinogen-III oxidase, aerobic OS=Coxiella burnetii (strain RSA 493 / Nine Mile phase I) GN=hemF PE=3 SV=1	310	36.411098	0.0076499	0	0.95	4.2	10	10	Q83AZ6
CBU_1730	Q83AZ5_COXBU Phosphoserine phosphatase OS=Coxiella burnetii (strain RSA 493 / Nine Mile phase I) GN=CBU_1730 PE=4 SV=1	258	29.216969	0.005186	60.65	26.38	36.6	-1.2	-0.73	Q83AZ5
CBU_1732	Q83AZ4_COXBU D-3-phosphoglycerate dehydrogenase OS=Coxiella burnetii (strain RSA 493 / Nine Mile phase I) GN=CBU_1732 PE=3 SV=1	388	42.818625	0.0066099	13.18	0	0.84	-10	-3.97	Q83AZ4
CBU_1734	Q83AZ2_COXBU DnaA-related protein OS=Coxiella burnetii (strain RSA 493 / Nine Mile phase I) GN=CBU_1734 PE=4 SV=1	237	26.797016	0.0057192	27.93	7.49	19.23	-1.9	-0.54	Q83AZ2
CBU_1735	Q83AZ1_COXBU Hypothetical exported protein OS=Coxiella burnetii (strain RSA 493 / Nine Mile phase I) GN=CBU_1735 PE=4 SV=2	381	41.209263	0.0060874	26.85	11.65	16.24	-1.2	-0.73	Q83AZ1
CBU_1736	PUR5_COXBU Phosphoribosylformylglycinamidine cyclo-ligase OS=Coxiella burnetii (strain RSA 493 / Nine Mile phase I) GN=purM PE=3 SV=1	352	38.174079	0.0067563	29.92	57.16	52.73	0.93	0.82	Q83AZ0
CBU_1737	Q83AY9_COXBU Phosphoribosylglycinamide formyltransferase OS=Coxiella burnetii (strain RSA 493 / Nine Mile phase I) GN=purN PE=1 SV=1	215	23.762485	0.0076792	23.79	2.75	1.51	-3.11	-3.97	Q83AY9
CBU_1739	Q820W0_COXBU Ribosomal RNA small subunit methyltransferase I OS=Coxiella burnetii (strain RSA 493 / Nine Mile phase I) GN=rsmI PE=3 SV=1	285	31.815343	0.0090415	23.23	40.49	41.13	0.8	0.82	Q820W0
CBU_1741	Q83AY6_COXBU Lipoprotein antigen OS=Coxiella burnetii (strain RSA 493 / Nine Mile phase I) GN=CBU_1741 PE=4 SV=1	396	44.244084	0.0100376	28.88	88.16	88.8	1.61	1.62	Q83AY6
CBU_1743	GMHA_COXBU Phosphoheptose isomerase OS=Coxiella burnetii (strain RSA 493 / Nine Mile phase I) GN=gmhA PE=3 SV=1	199	21.453898	0.0049321	89.22	44.6	65.45	-1	-0.45	Q83AY4
CBU_1744	Q83AY3_COXBU Lipoprotein OS=Coxiella burnetii (strain RSA 493 / Nine Mile phase I) GN=CBU_1744 PE=4 SV=1	191	20.663262	0.0105503	44.11	57.32	61.37	0.38	0.48	Q83AY3
CBU_1745	Q83AY2_COXBU Membrane endopeptidase, M50 family OS=Coxiella burnetii (strain RSA 493 / Nine Mile phase I) GN=CBU_1745 PE=4 SV=1	230	25.271274	0.0106821	3.92	20.58	25.48	2.39	2.7	Q83AY2
CBU_1747	SSPA_COXBU Stringent starvation protein A homolog OS=Coxiella burnetii (strain RSA 493 / Nine Mile phase I) GN=sspA PE=1 SV=1	209	24.381584	0.005478	190.05	171.3	168.26	-0.15	-0.18	Q83AY0
CBU_1748	RS9_COXBU 30S ribosomal protein S9 OS=Coxiella burnetii (strain RSA 493 / Nine Mile phase I) GN=rpsI PE=3 SV=1	139	15.277103	0.0117222	357.2	263.95	206.14	-0.44	-0.79	Q83AX9
CBU_1749	RL13_COXBU 50S ribosomal protein L13 OS=Coxiella burnetii (strain RSA 493 / Nine Mile phase I) GN=rplM PE=3 SV=1	142	15.783563	0.0104038	105.96	154.19	50.45	0.54	-1.07	Q83AX8
CBU_1751	Q83AX6_COXBU Uncharacterized protein OS=Coxiella burnetii (strain RSA 493 / Nine Mile phase I) GN=CBU_1751 PE=4 SV=1	420	48.787394	0.0079722	55.17	0	0	-10	-10	Q83AX6
CBU_1752	Q83AX5_COXBU Uncharacterized protein OS=Coxiella burnetii (strain RSA 493 / Nine Mile phase I) GN=CBU_1752 PE=4 SV=1	412	46.653143	0.0058081	91.3	0	0	-10	-10	Q83AX5
CBU_1754	Y1754_COXBU Uncharacterized protein CBU_1754 OS=Coxiella burnetii (strain RSA 493 / Nine Mile phase I) GN=CBU_1754 PE=1 SV=1	198	22.350176	0.0048306	97.27	5.98	24.67	-4.02	-1.98	Q83AX3
CBU_1761	Q83AW7_COXBU Two component system histidine kinase OS=Coxiella burnetii (strain RSA 493 / Nine Mile phase I) GN=CBU_1761 PE=4 SV=1	657	74.441338	0.0092466	0	2.25	14.87	10	10	Q83AW7
CBU_1770	Q83AV8_COXBU ABC transporter ATP-binding protein OS=Coxiella burnetii (strain RSA 493 / Nine Mile phase I) GN=CBU_1770 PE=3 SV=2	438	48.942377	0.0047036	172.44	90.52	153.14	-0.93	-0.17	Q83AV8
CBU_1772	ENGB_COXBU Probable GTP-binding protein EngB OS=Coxiella burnetii (strain RSA 493 / Nine Mile phase I) GN=engB PE=3 SV=1	205	23.040971	0.0084995	152.66	163.09	155.66	0.1	0.03	Q83AV6
CBU_1778	Q83AV0_COXBU Fructose-bisphosphate aldolase OS=Coxiella burnetii (strain RSA 493 / Nine Mile phase I) GN=fbaA PE=4 SV=1	363	39.489832	0.0053257	468.36	418.14	465.54	-0.16	-0.01	Q83AV0
CBU_1781	Q83AU7_COXBU Pyruvate kinase OS=Coxiella burnetii (strain RSA 493 / Nine Mile phase I) GN=pyk PE=3 SV=2	484	53.030027	0.0079575	205.17	172.39	163.48	-0.25	-0.33	Q83AU7
CBU_1782	PGK_COXBU Phosphoglycerate kinase OS=Coxiella burnetii (strain RSA 493 / Nine Mile phase I) GN=pgk PE=3 SV=1	391	42.318239	0.006189	160.85	262.58	235.67	0.71	0.55	Q83AU6
CBU_1783	Q83AU5_COXBU Glyceraldehyde 3-phosphate dehydrogenase OS=Coxiella burnetii (strain RSA 493 / Nine Mile phase I) GN=gap PE=3 SV=1	334	36.222646	0.0059097	1275.73	1447.48	1720.66	0.18	0.43	Q83AU5
CBU_1784	Q83AU4_COXBU Transketolase OS=Coxiella burnetii (strain RSA 493 / Nine Mile phase I) GN=tkt PE=4 SV=2	672	74.380164	0.0054399	251.21	175.68	152.63	-0.52	-0.72	Q83AU4
CBU_1787	Q83AU2_COXBU Glutamine--fructose-6-phosphate aminotransferase [isomerizing] OS=Coxiella burnetii (strain RSA 493 / Nine Mile phase I) GN=glmS PE=3 SV=1	611	67.293057	0.009686	28.07	31.96	29.31	0.19	0.06	Q83AU2

CBU_1789	Q83AU0_COXBU Gluconolactonase OS=Coxiella burnetii (strain RSA 493 / Nine Mile phase I) GN=CBU_1789 PE=4 SV=1	288	31.976009	0.0048052	10.45	30.82	39.57	1.56	1.92	Q83AU0
CBU_1795	Q83AT5_COXBU DNA polymerase I OS=Coxiella burnetii (strain RSA 493 / Nine Mile phase I) GN=polI PE=3 SV=2	902	101.74793	0.008939	4.34	13.12	25.27	1.6	2.54	Q83AT5
CBU_1810	Q83AS0_COXBU Macrolide-specific efflux protein OS=Coxiella burnetii (strain RSA 493 / Nine Mile phase I) GN=macA PE=4 SV=1	391	42.7286	0.0063667	0	8.32	19.99	10	10	Q83AS0
CBU_1812	PDXB_COXBU Erythronate-4-phosphate dehydrogenase OS=Coxiella burnetii (strain RSA 493 / Nine Mile phase I) GN=pdxB PE=3 SV=2	366	40.925405	0.0065366	0	8.89	18.68	10	10	Q83AR8
CBU_1816	EFP_COXBU Elongation factor P OS=Coxiella burnetii (strain RSA 493 / Nine Mile phase I) GN=efp PE=1 SV=1	188	21.013597	0.0056685	44.82	108.59	114.31	1.28	1.35	Q83AR4
CBU_1822	Q83AQ8_COXBU Superoxide dismutase [Cu-Zn] OS=Coxiella burnetii (strain RSA 493 / Nine Mile phase I) GN=sodC PE=3 SV=2	196	20.826036	0.0096567	35.31	212.85	264.14	2.59	2.9	Q83AQ8
CBU_1826	PSD_COXBU Phosphatidylserine decarboxylase proenzyme OS=Coxiella burnetii (strain RSA 493 / Nine Mile phase I) GN=psd PE=3 SV=1	282	31.902645	0.0094224	14.94	8.39	20.78	-0.83	0.48	Q83AQ4
CBU_1827	CCA_COXBU CCA-adding enzyme OS=Coxiella burnetii (strain RSA 493 / Nine Mile phase I) GN=cca PE=3 SV=1	376	42.393632	0.008939	0	3.93	3.46	10	10	Q820V9
CBU_1829	LOLB_COXBU Outer-membrane lipoprotein LolB OS=Coxiella burnetii (strain RSA 493 / Nine Mile phase I) GN=lolB PE=1 SV=1	210	24.105317	0.0098472	31.52	49.31	55.82	0.65	0.82	Q83AQ2
CBU_1830	KPRS_COXBU Ribose-phosphate pyrophosphokinase OS=Coxiella burnetii (strain RSA 493 / Nine Mile phase I) GN=prs PE=3 SV=1	319	35.181126	0.0057573	76.41	155.82	168.42	1.03	1.14	Q83AQ1
CBU_1835	Q83AP6_COXBU Protoporphyrinogen oxidase OS=Coxiella burnetii (strain RSA 493 / Nine Mile phase I) GN=CBU_1835 PE=4 SV=1	459	52.158797	0.0082358	62.94	2.58	1.42	-4.61	-5.47	Q83AP6
CBU_1836	Q83AP5_COXBU Homoserine dehydrogenase OS=Coxiella burnetii (strain RSA 493 / Nine Mile phase I) GN=CBU_1836 PE=4 SV=1	418	46.498065	0.0063032	219.57	0.71	0	-8.28	-10	Q83AP5
CBU_1837	Q83AP4_COXBU UDP-glucose 4-epimerase OS=Coxiella burnetii (strain RSA 493 / Nine Mile phase I) GN=CBU_1837 PE=4 SV=2	346	39.344243	0.0058335	79.14	3.42	0	-4.53	-10	Q83AP4
CBU_1838	Q83AP3_COXBU dTDP-4-dehydrorhamnose 3,5-epimerase OS=Coxiella burnetii (strain RSA 493 / Nine Mile phase I) GN=rfbC PE=4 SV=1	189	21.865262	0.0068442	44.58	1.57	0	-4.83	-10	Q83AP3
CBU_1840	RL25_COXBU 50S ribosomal protein L25 OS=Coxiella burnetii (strain RSA 493 / Nine Mile phase I) GN=rplY PE=3 SV=1	244	26.504616	0.0048433	531.53	383.18	293.58	-0.47	-0.86	Q83AP1
CBU_1842	Q83AN9_COXBU GTP-binding protein, probable translation factor OS=Coxiella burnetii (strain RSA 493 / Nine Mile phase I) GN=CBU_1842 PE=4 SV=2	367	40.233924	0.0051479	43.46	68.53	120.66	0.66	1.47	Q83AN9
CBU_1847	Q83AN5_COXBU Hypothetical exported protein OS=Coxiella burnetii (strain RSA 493 / Nine Mile phase I) GN=CBU_1847 PE=4 SV=1	81	8.9189227	0.0107993	48.29	87.67	4.02	0.86	-3.59	Q83AN5
CBU_1856	Q83AM7_COXBU 3-hydroxyisobutyryl-CoA hydrolase OS=Coxiella burnetii (strain RSA 493 / Nine Mile phase I) GN=CBU_1856 PE=4 SV=2	379	42.039477	0.0057573	0	6.25	8.59	10	10	Q83AM7
CBU_1863	Q83AM0_COXBU Hypothetical membrane spanning protein OS=Coxiella burnetii (strain RSA 493 / Nine Mile phase I) GN=CBU_1863 PE=4 SV=1	603	66.104146	0.0063667	11.48	0	0	-10	-10	Q83AM0
CBU_1865	Q83AL8_COXBU Hypothetical membrane associated protein OS=Coxiella burnetii (strain RSA 493 / Nine Mile phase I) GN=CBU_1865 PE=4 SV=2	180	20.962991	0.0097886	53.5	64.11	79.59	0.26	0.57	Q83AL8
CBU_1866	Q83AL7_COXBU Topoisomerase IV subunit A OS=Coxiella burnetii (strain RSA 493 / Nine Mile phase I) GN=parC PE=4 SV=1	753	84.300785	0.0095542	78.33	111.59	190.7	0.51	1.28	Q83AL7
CBU_1869	Q83AL4_COXBU Hypothetical exported protein OS=Coxiella burnetii (strain RSA 493 / Nine Mile phase I) GN=CBU_1869 PE=4 SV=1	217	24.716891	0.0094077	15.25	81.81	78.03	2.42	2.35	Q83AL4
CBU_1870	COQ7_COXBU 2-nonaprenyl-3-methyl-6-methoxy-1,4-benzoquinol hydroxylase OS=Coxiella burnetii (strain RSA 493 / Nine Mile phase I) GN=coq7 PE=3 SV=1	215	23.902146	0.0068296	7	6.88	21.2	-0.02	1.6	Q83AL3
CBU_1871	Q820B1_COXBU Anthranilate synthase component II OS=Coxiella burnetii (strain RSA 493 / Nine Mile phase I) GN=pabA PE=4 SV=1	201	22.231547	0.0075913	31.44	0	0	-10	-10	Q820B1
CBU_1872	Q83AL2_COXBU Ribulose-phosphate 3-epimerase OS=Coxiella burnetii (strain RSA 493 / Nine Mile phase I) GN=rpe PE=3 SV=2	231	25.18002	0.006189	32.57	65.32	95.85	1	1.56	Q83AL2
CBU_1874	Q83AL1_COXBU Glutamate--cysteine ligase OS=Coxiella burnetii (strain RSA 493 / Nine Mile phase I) GN=CBU_1874 PE=4 SV=1	435	48.909066	0.006189	35.97	19.04	34.43	-0.92	-0.06	Q83AL1
CBU_1875	GSHB_COXBU Glutathione synthetase OS=Coxiella burnetii (strain RSA 493 / Nine Mile phase I) GN=gshB PE=3 SV=1	321	36.278066	0.0057954	6.56	11.98	24.34	0.87	1.89	Q83AL0
CBU_1877	Q83AK8_COXBU Hypothetical ATPase OS=Coxiella burnetii (strain RSA 493 / Nine Mile phase I) GN=CBU_1877 PE=4 SV=1	427	49.760523	0.0091733	0	13.86	41.18	10	10	Q83AK8
CBU_1879	DEF2_COXBU Peptide deformylase 2 OS=Coxiella burnetii (strain RSA 493 / Nine Mile phase I) GN=def2 PE=3 SV=1	209	23.945468	0.0053638	210.21	144.4	218.11	-0.54	0.05	Q83AK6
CBU_1882	GSA_COXBU Glutamate-1-semialdehyde 2,1-aminomutase OS=Coxiella burnetii (strain RSA 493 / Nine Mile phase I) GN=hemL PE=3 SV=1	435	46.411731	0.0062905	23.52	3.4	4.49	-2.79	-2.39	Q83AK3
CBU_1883	Q83AK2_COXBU Queuosine biosynthesis protein OS=Coxiella burnetii (strain RSA 493 / Nine Mile phase I) GN=queD PE=4 SV=1	184	21.775872	0.0071958	22.9	12.86	14.16	-0.83	-0.69	Q83AK2
CBU_1887	Q83AJ8_COXBU Multimodular transpeptidase-transglycosylase PBP 1A OS=Coxiella burnetii (strain RSA 493 / Nine Mile phase I) GN=ponA PE=4 SV=1	793	88.221094	0.0095835	11	0	0	-10	-10	Q83AJ8
CBU_1893	AROB_COXBU 3-dehydroquinate synthase OS=Coxiella burnetii (strain RSA 493 / Nine Mile phase I) GN=aroB PE=3 SV=1	360	40.371173	0.0061763	2.51	35.34	50.65	3.82	4.34	Q83AJ2
CBU_1901	Q83AI5_COXBU Non-proteolytic protein, peptidase family M16 OS=Coxiella burnetii (strain RSA 493 / Nine Mile phase I) GN=CBU_1901 PE=3 SV=1	443	48.136298	0.0095249	16.3	108.87	94.82	2.74	2.54	Q83AI5
CBU_1902	Q83AI4_COXBU Peptidase, M16 family OS=Coxiella burnetii (strain RSA 493 / Nine Mile phase I) GN=CBU_1902 PE=3 SV=1	459	51.906407	0.0063032	47.86	157.93	120.6	1.72	1.33	Q83AI4
CBU_1903	Q83AI3_COXBU Signal recognition particle receptor FtsY OS=Coxiella burnetii (strain RSA 493 / Nine Mile phase I) GN=ftsY PE=3 SV=1	324	35.367946	0.0096567	1.86	2.74	7.03	0.56	1.92	Q83AI3
CBU_1910	H7C7D7_COXBU Outer membrane protein OS=Coxiella burnetii (strain RSA 493 / Nine Mile phase I) GN=com1 PE=4 SV=1	252	27.590456	0.0094517	7358.06	4038.93	3931.85	-0.87	-0.9	H7C7D7
CBU_1913	SYGA_COXBU Glycine--tRNA ligase alpha subunit OS=Coxiella burnetii (strain RSA 493 / Nine Mile phase I) GN=glyQ PE=3 SV=1	319	36.356022	0.0048052	6.6	14.84	24.5	1.17	1.89	P94616
CBU_1914	SYGB_COXBU Glycine--tRNA ligase beta subunit OS=Coxiella burnetii (strain RSA 493 / Nine Mile phase I) GN=glyS PE=3 SV=2	689	77.90749	0.0078843	27.08	24.05	41.11	-0.17	0.6	P45651
CBU_1916	USPA2_COXBU Universal stress protein A homolog 2 OS=Coxiella burnetii (strain RSA 493 / Nine Mile phase I) GN=uspA2 PE=1 SV=1	146	15.751365	0.0067124	391.6	1033.54	1074.95	1.4	1.46	P45680
CBU_1920	YIDC_COXBU Membrane protein insertase YidC OS=Coxiella burnetii (strain RSA 493 / Nine Mile phase I) GN=yidC PE=3 SV=2	566	64.46661	0.0097153	1.06	7.32	6.9	2.78	2.7	P45650
CBU_1924	MNMG_COXBU tRNA uridine 5-carboxymethylaminomethyl modification enzyme MnmG OS=Coxiella burnetii (strain RSA 493 / Nine Mile phase I) GN=mnmG PE=3 SV=2	627	69.907323	0.0080601	6.72	0	0	-10	-10	P94613
CBU_1926	Q83AH3_COXBU Chromosome partitioning protein OS=Coxiella burnetii (strain RSA 493 / Nine Mile phase I) GN=parA PE=4 SV=1	256	27.613513	0.0066099	8.23	21.96	19.08	1.42	1.21	Q83AH3

CBU_1927	PARB_COXBU Probable chromosome-partitioning protein ParB OS=Coxiella burnetii (strain RSA 493 / Nine Mile phase I) GN=parB PE=1 SV=2		290	32.184228	0.0093638	141.12	2.04	4.49	-6.11	-4.97	Q83AH2
CBU_1933	Q83AG6_COXBU Probable DNA repair protein CBU_1933 OS=Coxiella burnetii (strain RSA 493 / Nine Mile phase I) GN=CBU_1933 PE=3 SV=2		284	33.060635	0.0097886	0	11.46	13.76	10	10	Q83AG6
CBU_1941	ATPF_COXBU ATP synthase subunit b OS=Coxiella burnetii (strain RSA 493 / Nine Mile phase I) GN=atpF PE=3 SV=1		156	17.381334	0.0054019	94.52	34.14	48.01	-1.47	-0.98	Q83AF9
CBU_1942	ATPD_COXBU ATP synthase subunit delta OS=Coxiella burnetii (strain RSA 493 / Nine Mile phase I) GN=atpH PE=3 SV=1		185	21.157604	0.0098618	53.68	25.59	86.24	-1.07	0.68	Q83AF8
CBU_1943	ATPA_COXBU ATP synthase subunit alpha OS=Coxiella burnetii (strain RSA 493 / Nine Mile phase I) GN=atpA PE=1 SV=1		515	56.790777	0.0057319	417.19	151.1	148.58	-1.47	-1.49	Q83AF7
CBU_1944	ATPG_COXBU ATP synthase gamma chain OS=Coxiella burnetii (strain RSA 493 / Nine Mile phase I) GN=atpG PE=3 SV=1		289	32.574892	0.0079282	43.73	34.81	43.94	-0.33	0.01	Q83AF6
CBU_1945	ATPB_COXBU ATP synthase subunit beta OS=Coxiella burnetii (strain RSA 493 / Nine Mile phase I) GN=atpD PE=3 SV=1		461	50.385212	0.0048687	494.78	222.71	200.59	-1.15	-1.3	Q83AF5
CBU_1946	ATPE_COXBU ATP synthase epsilon chain OS=Coxiella burnetii (strain RSA 493 / Nine Mile phase I) GN=atpC PE=3 SV=1		142	15.259079	0.006189	125.03	122.93	240.77	-0.02	0.95	Q83AF4
CBU_1947	GLMU_COXBU Bifunctional protein GlmU OS=Coxiella burnetii (strain RSA 493 / Nine Mile phase I) GN=glmU PE=3 SV=1		455	49.263874	0.0072544	5.95	13.66	20.75	1.2	1.8	Q83AF3
CBU_1954	Q83AE7_COXBU (2R)-phospho-3-sulfolactate synthase OS=Coxiella burnetii (strain RSA 493 / Nine Mile phase I) GN=CBU_1954 PE=4 SV=1		290	33.308036	0.0052495	0	46.93	51.65	10	10	Q83AE7
CBU_1955	Q83AE6_COXBU NAD(P) transhydrogenase subunit alpha OS=Coxiella burnetii (strain RSA 493 / Nine Mile phase I) GN=pntAA PE=3 SV=2		419	45.135909	0.0089829	5.03	50.84	90.14	3.34	4.16	Q83AE6
CBU_1965	RF1_COXBU Peptide chain release factor 1 OS=Coxiella burnetii (strain RSA 493 / Nine Mile phase I) GN=prfA PE=3 SV=2		361	40.719986	0.0052368	4.17	4.92	3.61	0.24	-0.21	P47849
CBU_1969	Q83AD5_COXBU RNA polymerase-binding transcription factor DksA OS=Coxiella burnetii (strain RSA 493 / Nine Mile phase I) GN=dksA PE=3 SV=1		147	16.899398	0.0052749	24.56	38.24	59.81	0.64	1.28	Q83AD5
CBU_1970	DAPF_COXBU Diaminopimelate epimerase OS=Coxiella burnetii (strain RSA 493 / Nine Mile phase I) GN=dapF PE=1 SV=1		276	30.034333	0.0061255	243.13	325.89	384.6	0.42	0.66	Q83AD4
CBU_1975	Q83AC9_COXBU Carboxylesterase OS=Coxiella burnetii (strain RSA 493 / Nine Mile phase I) GN=CBU_1975 PE=4 SV=2		236	26.323765	0.0059731	179.78	102.8	122.79	-0.81	-0.55	Q83AC9
CBU_1978	LPTD_COXBU LPS-assembly protein LptD OS=Coxiella burnetii (strain RSA 493 / Nine Mile phase I) GN=lptD PE=3 SV=1		870	98.996008	0.0090708	1.38	4.08	4.49	1.56	1.7	Q83AC6
CBU_1980	Q83AC4_COXBU Peptidyl-prolyl cis-trans isomerase OS=Coxiella burnetii (strain RSA 493 / Nine Mile phase I) GN=CBU_1980 PE=4 SV=2		321	35.893355	0.0102573	336.54	82.96	84.19	-2.02	-2	Q83AC4
CBU_1981	Q83AC3_COXBU 4-hydroxythreonine-4-phosphate dehydrogenase OS=Coxiella burnetii (strain RSA 493 / Nine Mile phase I) GN=pdxA PE=3 SV=1		307	32.635512	0.0073569	0	42.41	54.09	10	10	Q83AC3
CBU_1982	RSMA_COXBU Ribosomal RNA small subunit methyltransferase A OS=Coxiella burnetii (strain RSA 493 / Nine Mile phase I) GN=rsmA PE=1 SV=2		258	29.74164	0.009437	9.33	1.15	8.83	-3.02	-0.08	Q83AC2
CBU_1983	USPA1_COXBU Universal stress protein A homolog 1 OS=Coxiella burnetii (strain RSA 493 / Nine Mile phase I) GN=uspA1 PE=3 SV=1		144	15.718228	0.0059731	198.52	189.03	228.38	-0.07	0.2	Q83AC1
CBU_1985	Q83AB9_COXBU Hypothetical exported protein OS=Coxiella burnetii (strain RSA 493 / Nine Mile phase I) GN=CBU_1985 PE=4 SV=1		232	26.252572	0.0096128	25.94	124.98	138.95	2.27	2.42	Q83AB9
CBU_1986	Q83AB8_COXBU Signal peptide peptidase OS=Coxiella burnetii (strain RSA 493 / Nine Mile phase I) GN=sppA PE=4 SV=1		313	35.070341	0.0088804	98.06	170.15	172.69	0.8	0.82	Q83AB8
CBU_1991	Q83AB4_COXBU RelB OS=Coxiella burnetii (strain RSA 493 / Nine Mile phase I) GN=relB PE=4 SV=1		84	9.6002384	0.0102427	32.24	0	0	-10	-10	Q83AB4
CBU_1993	Q83AB2_COXBU Dihydrofolate reductase OS=Coxiella burnetii (strain RSA 493 / Nine Mile phase I) GN=folA PE=1 SV=1		161	18.632946	0.009144	37.38	40.43	44.49	0.11	0.25	Q83AB2
CBU_1996	Q83AA9_COXBU D,D-heptose 1,7-bisphosphate phosphatase OS=Coxiella burnetii (strain RSA 493 / Nine Mile phase I) GN=CBU_1996 PE=3 SV=1		184	20.16839	0.0053003	179.89	165.63	122.1	-0.12	-0.56	Q83AA9
CBU_1997	FMT_COXBU Methionyl-tRNA formyltransferase OS=Coxiella burnetii (strain RSA 493 / Nine Mile phase I) GN=fmt PE=1 SV=1		314	34.248181	0.0079722	17.25	56.54	39.4	1.71	1.19	Q83AA8
CBU_2000	Q83AA5_COXBU DNA topoisomerase OS=Coxiella burnetii (strain RSA 493 / Nine Mile phase I) GN=topA PE=3 SV=1		765	87.132609	0.0084263	17.31	32.49	53.2	0.91	1.62	Q83AA5
CBU_2002	Q83AA3_COXBU N5-carboxyaminoimidazole ribonucleotide mutase OS=Coxiella burnetii (strain RSA 493 / Nine Mile phase I) GN=purE PE=1 SV=1		166	17.157125	0.0082358	16.31	35.65	52.96	1.13	1.7	Q83AA3
CBU_2006	Q83AA0_COXBU Two-component response regulator OS=Coxiella burnetii (strain RSA 493 / Nine Mile phase I) GN=CBU_2006 PE=4 SV=1		245	27.305486	0.0065366	77.38	0	0	-10	-10	Q83AA0
CBU_2008	SYR_COXBU Arginine--tRNA ligase OS=Coxiella burnetii (strain RSA 493 / Nine Mile phase I) GN=argS PE=3 SV=1		592	66.705235	0.0061509	20.84	27.49	30.8	0.4	0.56	Q83A98
CBU_2009	Q83A97_COXBU Uncharacterized protein OS=Coxiella burnetii (strain RSA 493 / Nine Mile phase I) GN=CBU_2009 PE=4 SV=2		444	50.119451	0.0094956	155.2	49.98	38.87	-1.63	-2	Q83A97
CBU_2011	HSLV_COXBU ATP-dependent protease subunit HslV OS=Coxiella burnetii (strain RSA 493 / Nine Mile phase I) GN=hslV PE=3 SV=1		181	19.520381	0.0097446	251.04	528	615.24	1.07	1.29	Q83A95
CBU_2012	HSLU_COXBU ATP-dependent protease ATPase subunit HslU OS=Coxiella burnetii (strain RSA 493 / Nine Mile phase I) GN=hslU PE=3 SV=1		447	50.132531	0.0054653	341.98	368.69	347.46	0.11	0.02	Q83A94
CBU_2017	UBIE_COXBU Ubiquinone/menaquinone biosynthesis methyltransferase ubiE OS=Coxiella burnetii (strain RSA 493 / Nine Mile phase I) GN=ubiE PE=3 SV=1		250	28.200593	0.0081626	99.9	57.99	57.31	-0.78	-0.8	Q83A90
CBU_2018	Q83A89_COXBU Uncharacterized protein OS=Coxiella burnetii (strain RSA 493 / Nine Mile phase I) GN=CBU_2018 PE=4 SV=2		204	22.594713	0.0067124	1.48	11.6	3.19	2.98	1.11	Q83A89
CBU_2019	Q83A88_COXBU 2-polyprenylphenol 6-hydroxylase accessory protein OS=Coxiella burnetii (strain RSA 493 / Nine Mile phase I) GN=ubiB PE=3 SV=1		541	62.400412	0.0095981	0	3.28	1.2	10	10	Q83A88
CBU_2023	Q83A85_COXBU Uncharacterized protein OS=Coxiella burnetii (strain RSA 493 / Nine Mile phase I) GN=CBU_2023 PE=4 SV=1		81	9.3410229	0.0093784	7.43	54.79	28.14	2.88	1.92	Q83A85
CBU_2024	Q83A84_COXBU Cysteine synthase OS=Coxiella burnetii (strain RSA 493 / Nine Mile phase I) GN=CBU_2024 PE=3 SV=1		316	34.275725	0.0048306	71.42	64.61	77.28	-0.14	0.11	Q83A84
CBU_2025	METC_COXBU Cystathionine beta-lyase OS=Coxiella burnetii (strain RSA 493 / Nine Mile phase I) GN=metC PE=1 SV=1		387	42.731084	0.0082065	30.32	50.46	129.57	0.73	2.1	Q83A83
CBU_2029	Q83A79_COXBU Hypothetical exported protein OS=Coxiella burnetii (strain RSA 493 / Nine Mile phase I) GN=CBU_2029 PE=4 SV=1		210	24.376516	0.0099937	108.9	46.49	44.96	-1.23	-1.28	Q83A79
CBU_2030	METK_COXBU S-adenosylmethionine synthase OS=Coxiella burnetii (strain RSA 493 / Nine Mile phase I) GN=metK PE=3 SV=1		393	42.732673	0.005478	430.31	48.18	14.91	-3.16	-4.85	Q83A78
CBU_2031	SAHH_COXBU Adenosylhomocysteinase OS=Coxiella burnetii (strain RSA 493 / Nine Mile phase I) GN=ahcY PE=3 SV=2		438	48.81845	0.0054399	276.87	33.78	52.78	-3.04	-2.39	Q83A77
CBU_2049	Q83A61_COXBU Tryptophanyl-tRNA synthetase OS=Coxiella burnetii (strain RSA 493 / Nine Mile phase I) GN=trpS PE=4 SV=1		356	40.617903	0.0062271	33.81	54.02	63.11	0.68	0.9	Q83A61
CBU_2054	Q83A56_COXBU DNA helicase II OS=Coxiella burnetii (strain RSA 493 / Nine Mile phase I) GN=uvrD PE=4 SV=2		723	82.373242	0.006481	5.83	0	0	-10	-10	Q83A56
CBU_2069	KITH_COXBU Thymidine kinase OS=Coxiella burnetii (strain RSA 493 / Nine Mile phase I) GN=tdk PE=3 SV=1		196	22.006271	0.0070493	26.1	155.49	154.5	2.57	2.57	Q83A42

CBU_2070	Q83A41_COXBU Chorismate mutase family protein OS=Coxiella burnetii (strain RSA 493 / Nine Mile phase I) GN=CBU_2070 PE=4 SV=1	136	15.58347	0.0102573	141.61	95.72	102.95	-0.56	-0.46	Q83A41
CBU_2074	HEM3_COXBU Porphobilinogen deaminase OS=Coxiella burnetii (strain RSA 493 / Nine Mile phase I) GN=hemC PE=3 SV=1	307	33.887923	0.0084556	0	2.89	14.85	10	10	Q83A37
CBU_2075	AROD_COXBU 3-dehydroquinate dehydratase OS=Coxiella burnetii (strain RSA 493 / Nine Mile phase I) GN=aroD PE=3 SV=1	233	26.472913	0.007269	23.25	39.37	58.69	0.76	1.34	Q83A36
CBU_2080	Q83A31_COXBU Uroporphyrin-III C-methyltransferase OS=Coxiella burnetii (strain RSA 493 / Nine Mile phase I) GN=CBU_2080 PE=4 SV=2	320	35.614883	0.0066245	124.13	311.59	271.68	1.33	1.13	Q83A31
CBU_2081	Q83A30_COXBU HemY OS=Coxiella burnetii (strain RSA 493 / Nine Mile phase I) GN=hemY PE=4 SV=1	392	45.405544	0.0100815	36.08	144.16	149.51	2	2.05	Q83A30
CBU_2082	Q83A29_COXBU Hypothetical exported protein OS=Coxiella burnetii (strain RSA 493 / Nine Mile phase I) GN=CBU_2082 PE=4 SV=2	255	28.08805	0.0103599	27.14	26.69	7.66	-0.02	-1.82	Q83A29
CBU_2086	Q83A25_COXBU Transcription termination factor Rho OS=Coxiella burnetii (strain RSA 493 / Nine Mile phase I) GN=rho PE=3 SV=1	418	46.766557	0.0062524	555.04	633.51	627.85	0.19	0.18	Q83A25
CBU_2087	Q83A24_COXBU Thioredoxin OS=Coxiella burnetii (strain RSA 493 / Nine Mile phase I) GN=trx PE=3 SV=1	112	12.587295	0.0047163	795.27	760.82	747.16	-0.06	-0.09	Q83A24
CBU_2090	Q83A21_COXBU Pyrroline-5-carboxylate reductase OS=Coxiella burnetii (strain RSA 493 / Nine Mile phase I) GN=proC PE=1 SV=1	274	29.571624	0.0057954	135.08	185.73	102.2	0.46	-0.4	Q83A21
CBU_2091	Q83A20_COXBU Hypothetical cytosolic protein OS=Coxiella burnetii (strain RSA 493 / Nine Mile phase I) GN=CBU_2091 PE=3 SV=1	228	25.611667	0.009979	26.4	35.04	47.13	0.41	0.84	Q83A20
CBU_2092	PCKA_COXBU Phosphoenolpyruvate carboxykinase [ATP] OS=Coxiella burnetii (strain RSA 493 / Nine Mile phase I) GN=pckA PE=3 SV=1	517	56.753765	0.0057954	326.52	281.57	306.71	-0.21	-0.09	Q83A19
CBU_2093	Y2093_COXBU UPF0301 protein CBU_2093 OS=Coxiella burnetii (strain RSA 493 / Nine Mile phase I) GN=CBU_2093 PE=3 SV=2	194	21.308276	0.0058208	46.53	12.2	0	-1.93	-10	Q83A18
CBU_2095	PYRB_COXBU Aspartate carbamoyltransferase OS=Coxiella burnetii (strain RSA 493 / Nine Mile phase I) GN=pyrB PE=3 SV=1	310	34.90434	0.0093638	15.53	58.22	97.68	1.91	2.65	Q83A16
CBUA0010	Q83A13_COXBU Phage integrase family protein OS=Coxiella burnetii (strain RSA 493 / Nine Mile phase I) GN=CBUA0010 PE=4 SV=2	417	48.648297	0.0096714	4.33	0	0	-10	-10	Q83A13
CBUA0013	H7C7F5_COXBU Uncharacterized protein OS=Coxiella burnetii (strain RSA 493 / Nine Mile phase I) GN=CBUA0013 PE=4 SV=1	243	27.935267	0.0087925	21.05	0	0	-10	-10	H7C7F5
CBUA0023	Q83A05_COXBU Uncharacterized protein OS=Coxiella burnetii (strain RSA 493 / Nine Mile phase I) GN=CBUA0023 PE=4 SV=1	233	26.671911	0.0094224	28.41	0	0	-10	-10	Q83A05
CBUA0027	H7C7G0_COXBU Putative DNA-binding protein OS=Coxiella burnetii (strain RSA 493 / Nine Mile phase I) GN=CBUA0027 PE=4 SV=1	114	12.873003	0.0103159	0	5.19	22.85	10	10	H7C7G0
CBUA0038	H7C7C9_COXBU DNA-binding protein OS=Coxiella burnetii (strain RSA 493 / Nine Mile phase I) GN=parB.2 PE=4 SV=1	334	37.56702	0.0095688	12.61	7.09	1.95	-0.83	-2.69	H7C7C9
